# The modulatory role of sulfated and non-sulfated small molecule heparan sulfate-glycomimetics in endothelial dysfunction: absolute structural clarification, molecular docking and simulated dynamics, SAR analyses and ADMET studies†

**DOI:** 10.1039/d0md00366b

**Published:** 2021-04-23

**Authors:** Daniel M. Gill, Ana Paula R. Povinelli, Gabriel Zazeri, Sabrina A. Shamir, Ayman M. Mahmoud, Fiona L. Wilkinson, M. Yvonne Alexander, Marinonio L. Cornelio, Alan M. Jones

**Affiliations:** School of Pharmacy, University of Birmingham Edgbaston B15 2TT UK a.m.jones.2@bham.ac.uk +44(0)121 414 7288; Departamento de Física – IBILCE Rua Cristovão Colombo 2265 CEP 15054-000 São José do Rio Preto São Paulo Brazil; Department of Natural Sciences, Manchester Metropolitan University M1 5GD UK; Physiology Division, Department of Zoology, Faculty of Science, Beni-Suef University Egypt; Department of Endocrinology, Diabetes & Nutrition, Center for Cardiovascular Research (CCR), Charité – Universitätsmedizin Berlin Berlin Germany; Centre for Biomedicine, Manchester Metropolitan University M1 5GD UK

## Abstract

The conceptual technology of small molecule glycomimetics, exemplified by compounds **C1–4**, has shown promising protective effects against lipid-induced endothelial dysfunction, restorative effects on diabetic endothelial colony forming cells, and preventative effects on downstream vascular calcification amongst other important *in vitro* and *ex vivo* studies. We report the optimised synthesis of an array of 17 small molecule glycomimetics, including the regio-, enantio- and diastereo-meric sulfated scaffolds of a hit structure along with novel desulfated examples. For the first time, the absolute stereochemical configurations of **C1–4** have been clarified based on an identified and consistent anomaly with the Sharpless asymmetric dihydroxylation reaction. We have investigated the role and importance of sulfation pattern, location, regioisomers, and spatial orientation of distal sulfate groups on the modulation of endothelial dysfunction through their interaction with hepatocyte growth factor (HGF). *In silico* studies demonstrated the key interactions the persulfated glycomimetics make with HGF and revealed the importance of both sulfate density and positioning (both point chirality and vector) to biological activity. *In vitro* biological data of the most efficient binding motifs, along with desulfated comparators, support the modulatory effects of sulfated small molecule glycomimetics in the downstream signaling cascade of endothelial dysfunction. *In vitro* absorption, distribution, metabolism, elimination and toxicity (ADMET) data demonstrate the glycomimetic approach to be a promising approach for hit-to-lead studies.

## Abstract 2

The conceptual technology of small molecule glycomimetics, exemplified by compounds **C1–4**, has shown promising protective effects against lipid-induced endothelial dysfunction.

## Graphical abstract

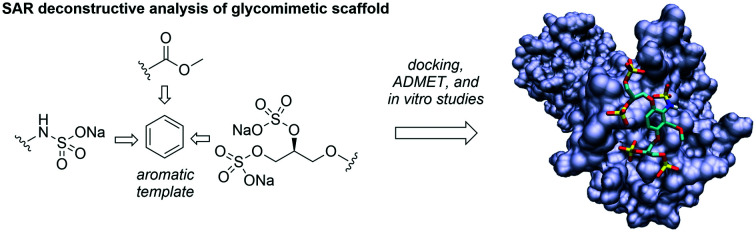

## Introduction

The endothelium plays a crucial role in the regulation of vascular haemostasis, vascular tone, thrombosis and thrombolysis.^1^ Endothelial dysfunction (ED) has been associated with the increased risk of atherosclerosis and cardiovascular complications,^2^ characterized by the reduced bioavailability of the vasodilator nitric oxide (NO) and increased levels of reactive oxygen species (ROS).^3^ Elevation in the level of free fatty acids (FFA) in the blood plasma has been implicated as an associated factor for the destruction of the endothelium, partly due to the enhanced production of ROS, which in turn reduces the availability of intracellular NO.^4^ Activation of NADPH oxidase by FFA leads to the deactivation of endothelial nitric oxide synthase (eNOS) and the down-regulation of the protective pathways.^5^

**Fig. 1:**
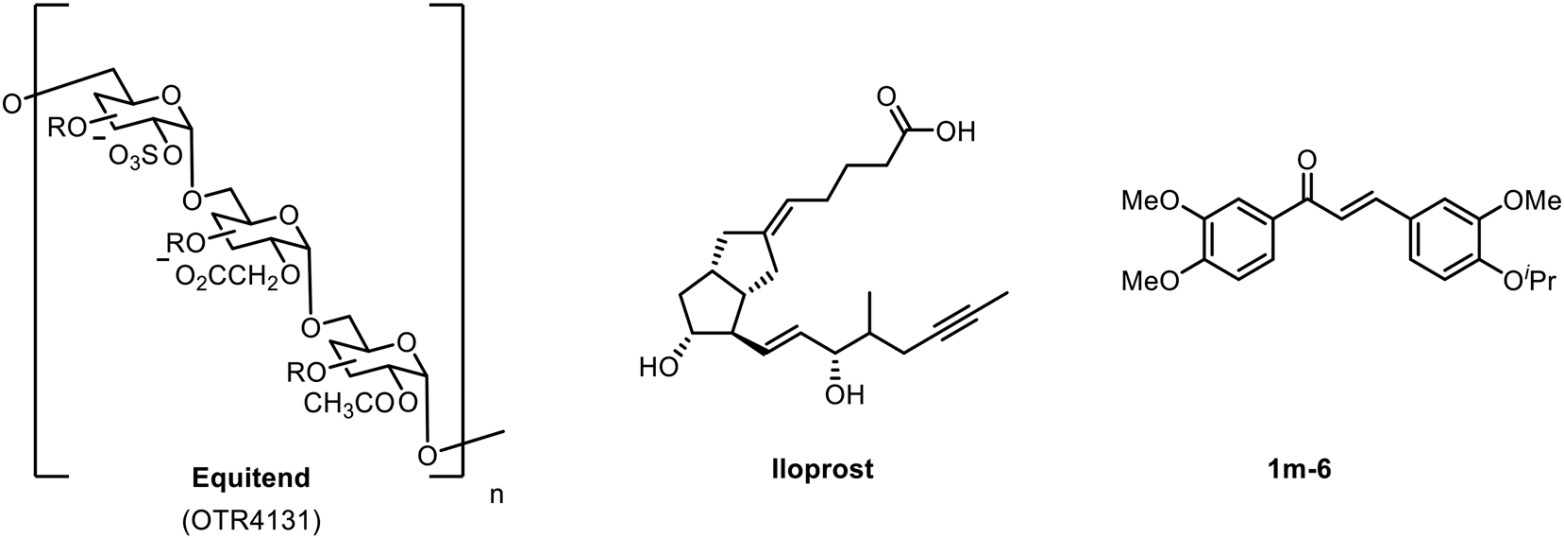
Molecular scaffold approaches to modulating endothelial dysfunction.

Examples of endothelial modulators are shown in Fig. 1, including the polysaccharide-based glycomimetic **Equitend**,^6^ an analogue of prostacyclin **Iloprost**^7^ and chalcone derivative **1m-6**.^8^ Our approach uses glycomimetics—small molecules that are designed to mimic the function of polysaccharide based biomolecules—and serve as an attractive therapeutic technology in a wide range of diseases.^9,10^ Recent advances of the glycomimetic approach have included the development of selective carbonic anhydrase inhibitors;^11^ and the use of highly sulfated glycomimetics as inhibitors of viral binding.^12^

**Fig. 2:**
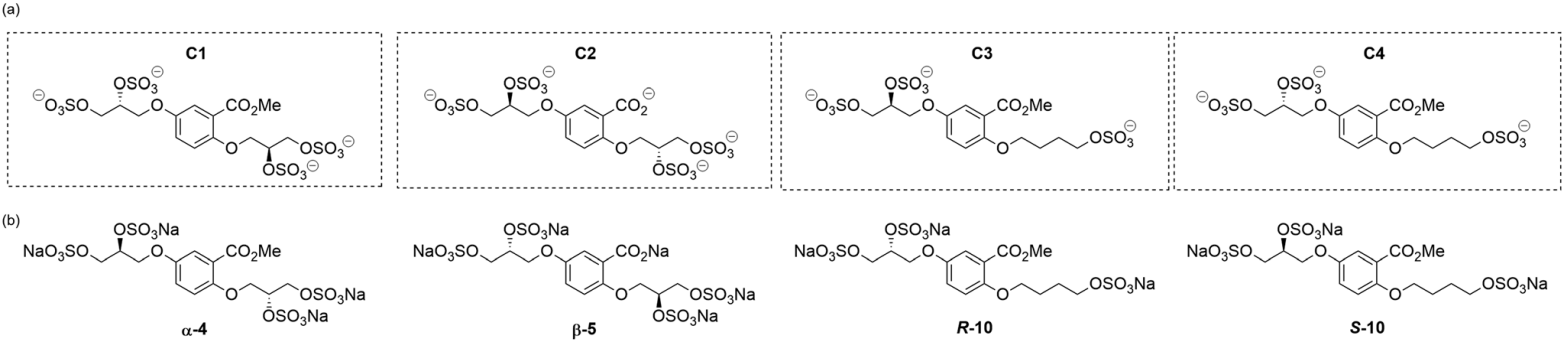
(a) Originally proposed stereoisomers of **C1–C4**;^13^ and (b) clarification of the stereochemical outcomes for **C1–C4** from *this work*.

The first generation glycomimetics (**C1–C4**, Fig. 2a), were originally designed as inhibitors of hepatocyte growth factor/scatter factor (HGF/SF)-induced hepatocyte growth factor receptor (c-Met) activation, affecting tumour cell growth and angiogenesis.^13^ It should be noted that the findings of the *present work* indicate that the original discovery experiments^13^ were likely performed with ambiguous mixtures of these glycomimetics.

It was subsequently found that **C1–C4** are superior to attenuate lipid-induced endothelial dysfunction and regenerating eNOS activity.^14^ We have also recently disclosed that our aryl templated, small molecule heparan sulfate-glycomimetics modulate vascular calcification^15^ and diabetic endothelial colony forming cells *in vitro*.^16^

Hepatocyte growth factor HGF/c-Met signalling has been reported to affect vascular calcification, an end point of endothelial dysfunction in the atherosclerotic spectrum.^17^ Heparan sulfate acts as a ligand for activation of the HGF/c-Met signalling pathway, thus one can attenuate this pathway using small molecule heparan sulfate-glycomimetics and reduce vascular calcification as a risk factor for cardiovascular diseases. The downstream signalling of HGF/c-Met modulates the phosphoinositide 3-kinase/protein kinase B (PI3K/Akt) pathway leading to the production of the biomarker nitric oxide (NO) *via* eNOS which has restorative effects on damaged endothelial cells.^14^

Herein, we report the structural clarification, and the optimised modular synthesis of small molecule heparan sulfate-glycomimetics, with molecular docking and dynamics of their interaction with HGF. The biological evaluation of small molecule glycomimetics as modulators for endothelial dysfunction combined with ADMET results demonstrate the concept of this new technology in glycomimetic design.

## Results and discussion

### An improved synthesis of **C1–C4**

**Scheme 1:**
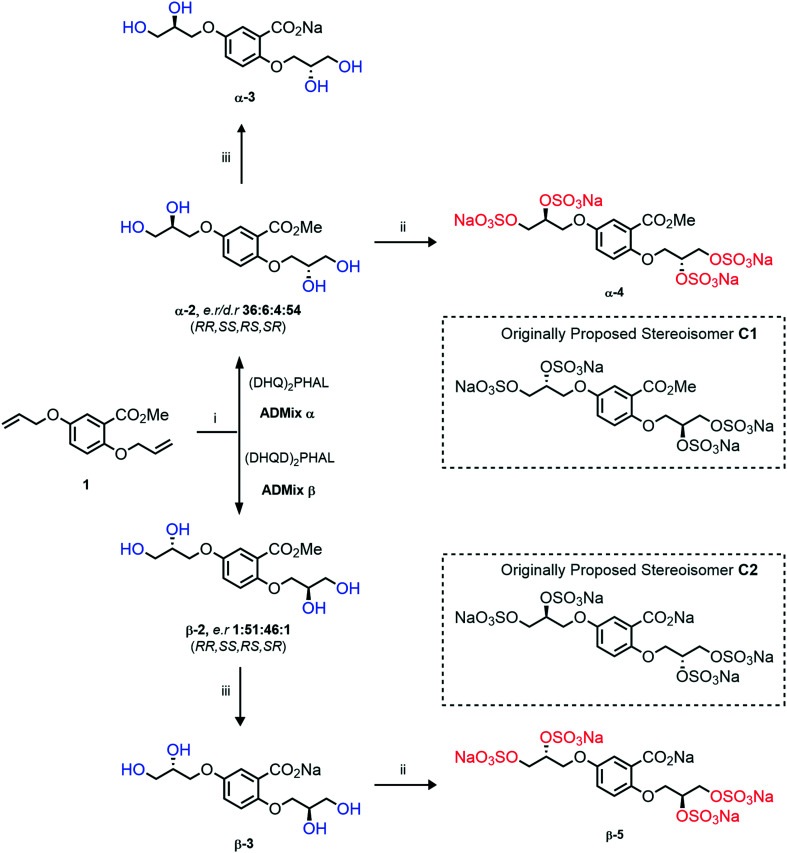
The synthesis of first-generation heparan sulfate-glycomimetics α-**4** (**C1**) and β-**5** (**C2**), including biologically relevant intermediates considered in this investigation. Conditions: i) K_2_CO_3_, K_3_Fe(CN)_6_, K_2_OsO_2_(OH)_4_, ^*t*^BuOH/H_2_O, 0 °C, 12 h, **99**% for both; ii) TBSAB, MeCN, 90 °C, 8 h **88**% and **86**% for α-**4** and β-**5**, respectively; iii) NaOH, MeOH, 70 °C, 2 h **99**% for both.

Initially, we sought to prepare the first generation glycomimetics (**C1–C4**, Fig. 2a) as a route to access both sulfated and non-sulfated analogues for a parallel biological evaluation. The knowledge gained from the results of our previously described chiral HPLC study, investigating the tandem Sharpless asymmetric dihydroxylation (AD) on diene **1**, provided evidence for the stereochemical configurations of glycomimetics **C1** and **C2** (Fig. 2b/Scheme 1).^18^ The chiral HPLC results highlighted that the tetraol precursors (α-**2** and β-**2**) contain mixtures of diastereo- and enantio-meric pairs, and importantly the stereochemistry of the major diastereomeric species is reversed in comparison to the original literature report.^13^ The stereochemistry of the originally assigned tetraols and subsequent glycomimetics from the original report^13^ were clarified (Fig. 2b and Scheme 1). The persulfation of all tetraol intermediates was carried out using the TBSAB-sulfation methodology^19–21^ (Scheme 1), specifically, the use of this reagent afforded the heparan sulfate-glycomimetics α-**4** (**C1**) and β-**5** (**C2**) in 88% and 86% yield, respectively.

**Scheme 2:**
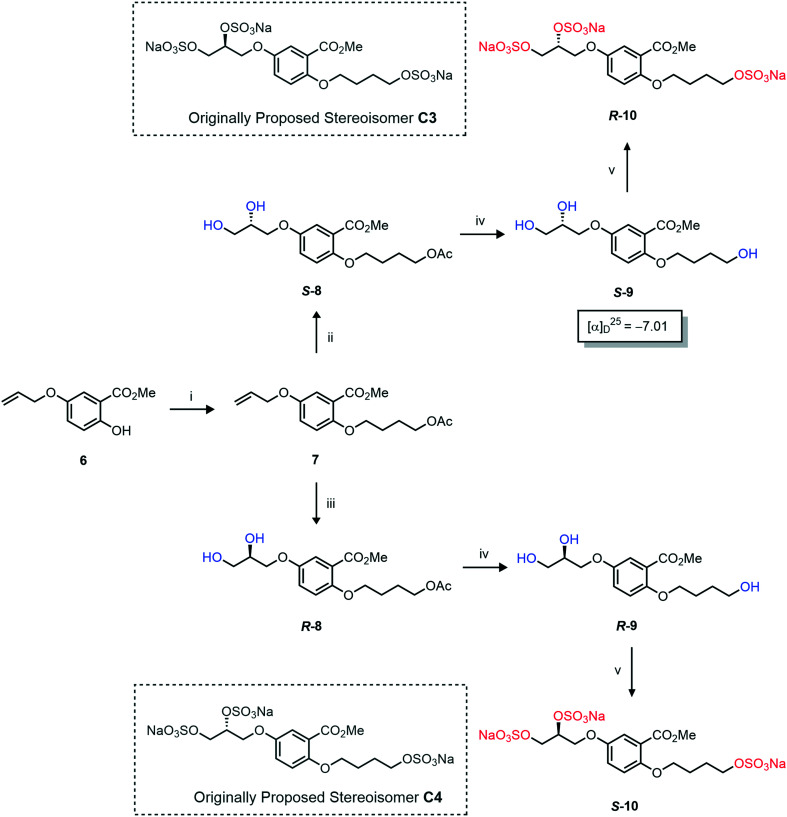
The synthesis of heparan sulfate-glycomimetics *S*-**10** (**C4**) and *R*-**10** (**C3**). Conditions: i) 4-bromobutylacetate, K_2_CO_3_, TBAI, acetone, reflux, 12 h, **90**%, ii) ADmix β, ^*t*^BuOH/H_2_O, 0 °C, 12 h, **99**% for both; iii) ADmix α, ^*t*^BuOH/H_2_O, 0 °C, 12 h, **99**% for both; iv) K_2_CO_3_, MeOH, rt, 12 h, **99**% for both; v) TBSAB, MeCN, 90 °C, 12 h, **72**% and **97**% for *R*-**10** and *S*-**10**, respectively.

The synthesis of diols *R*-**10** and *S*-**10** were adapted from the original report^13^ (Scheme 2). With alkene **7** in hand, AD reactions were performed with Sharpless formulations α and β under standard conditions,^22^ affording diols *S*-**8** and *R*-**8** in 99% yield (Scheme 2). A chemoselective hydrolysis of the acetate functionality using K_2_CO_3_ in methanol afforded triols *S*-**9** and *R*-**9** in 99% yield. Persulfation of the triols with TBSAB afforded the first-generation heparan sulfate-glycomimetics *R*-**10** (previously known as **C3**) and *S*-**10** (previously known as **C4**) in 72% and 97% yield, respectively.

**Scheme 3:**
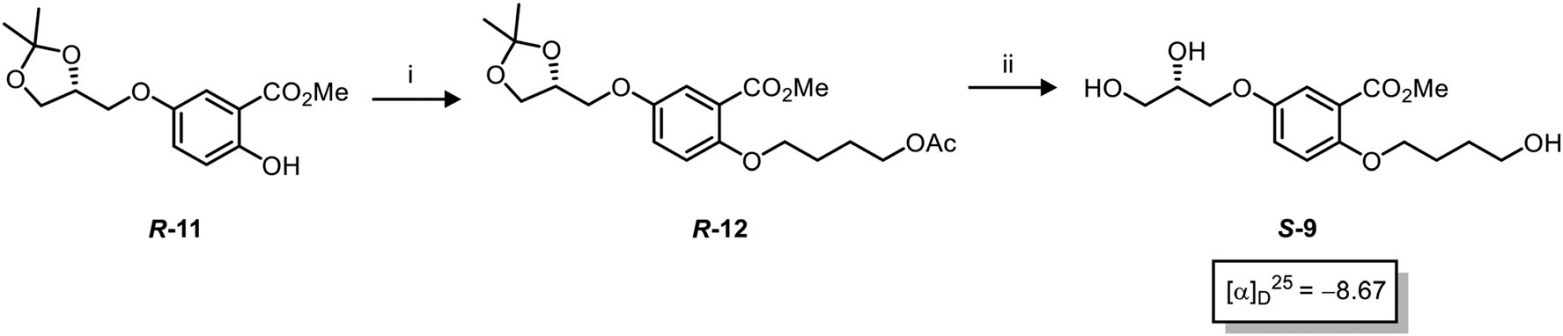
A chiral pool synthesis of *S*-**9** for the comparison of optical rotation. Conditions: i) 4-bromobutylacetate, K_2_CO_3_, TBAI, acetone, reflux, 12 h, **90**%, ii) 1) NaOMe, MeOH, 2) TFA, MeOH, 12 h, **99**%.

Asymmetric induction in the AD has been demonstrated to work effectively at the *meta* position alkene in **7**.^18^ Therefore this investigation was confident that the desired enantiomers were synthesized in a large enantiomeric excess. However, to further confirm the stereochemistry of these enantiomers, the synthesis of enantiomer *S*-**9** was prepared using a chiral pool method (Scheme 3), and the optical rotations of prepared *S*-**9** (Scheme 2) and authentic chiral pool *S*-**9** were compared.

The optical rotations correlated with the predicted stereochemical configuration, and the closely matching values (−7.01 *vs.* −8.67, c 1.0 in CHCl_3_) confirmed that the correct enantiomer was synthesised with a calculated 91 : 9 *e.r* (*S*/*R*) (Scheme 3). Furthermore, the stereochemical configurations of the triols **9** and resulting heparan sulfate-glycomimetics **10** also opposed the assigned stereochemistry from the original report^13^ (Fig. 2a).

Overall, this investigation has assigned the correct stereochemistry to all previously reported heparan sulfate-glycomimetics (**C1–C4**) and provided a practical and efficient synthesis to the final target structures.

### *In silico* studies

A computational approach was used to triage the key modified compounds based on α-**4** as the prototypical first-generation small molecule glycomimetic, to take forward for biological evaluation and *in vitro* ADMET studies.

The computational model was built based on the X-ray crystal structures of NK1–HGF (PDB: 1BHT, 2.0 Å resolution).^23^ Each heparan sulfate-glycomimetic and non-sulfated intermediate was docked individually against 1BHT under matching conditions and each minimum docking energy was calculated.^24^

Molecular docking ranked the glycomimetics according to their affinities toward the NK1–heparin binding site (Table S3†). MM/PBSA calculations were performed along with molecular dynamics to obtain a more accurate prediction of binding free energy of the active candidates. Such techniques incorporate conformational fluctuations, entropic contributions, and solvent molecules interactions, which are not considered by molecular docking that is based on simple energetic and geometric criteria. The glycomimetic binding energies were rescored based on MM/PBSA, as summarised in Table S3.† The exploration of detailed energy composition obtained from MM/PBSA, revealed that the predominant interaction of a negatively charged glycomimetic toward N-domain of the protein is electrostatic in nature. Not surprisingly, the microenvironment of interaction disclosed by docking is largely composed by positively charged amino acids residues of: Arg73 and Lys78, 58, 60, 62, 63, and 78 (Fig. S1†).

**Fig. 3:**
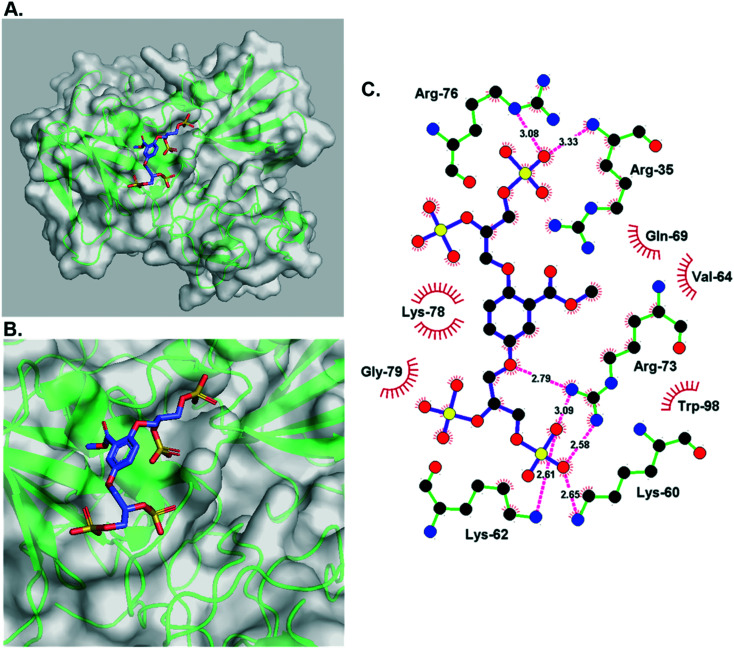
A) The minimum energy docked structure of heparan sulfate-glycomimetic α-**4** into the heparan sulfate-binding site of NK1 (HGF), Table 1, entry 3; B) zoomed in view of the docked structure. Key: amino acids are represented as green ribbons and polar surface area is opaque white; C) Ligplot representation of individual binding interactions displaying vicinal amino acid residues and hydrogen bonds.

Interestingly, the docked structure of the α-**4**–HGF complex (Fig. 3A) shows that the methyl ester is orientated directly into a hydrophobic pocket on the protein's accessible surface (Fig. 3B). Comparing this interaction to the β-**5**–HGF complex (Fig. S1†) indicates that this mode of binding is specific to the methyl ester group (present in heparan sulfate-glycomimetics α-**4**, *R*-**10**, and *S*-**10**), and the anionic carboxylate group (present in heparan sulfate-glycomimetic β-**5**) is orientated differently inside the heparan sulfate-binding site of HGF. This interaction is not gained through further electrostatic forces and is likely driven by enhanced hydrogen bonding and van der Waals forces. Consequently, the methyl ester is considered important to the design of a second generation of heparan sulfate-glycomimetics.

## Computational SAR around prototypical glycomimetic **4**

The first generation glycomimetics included a modified linear linker at the 2-position, and comparison with α-**4** showed a modest loss of affinity (*R*-**10** −41.0 or *S*-**10** −42.0 *vs.* α-**4** −47.4 kJ mol^−1^ (Table 1)). The molecules α-**4**, β-**5** and **26** presented the greatest binding energy values and therefore the highest affinity for the HGF/NK1 binding site. Moreover, the results showed that the binding sites are composed mainly by charged and polar amino acids and that electrostatic interaction and hydrogen bonding interactions dominate. Both α-**4** and β-**5** presented a considerable number of hydrogen bonds with the amino acids at Lys60, Lys62, and Arg73, (β-**5** also had further interactions with Gln69 and Thr61) (Fig. S1†). These results indicated that α-**4** and β-**5** are the most promising molecules to bind HGF–NK1, as they have both high affinity to the binding site and provided high stability to the resulting complex formed. In summary, it was shown that the sulfate groups present in α-**4**/β-**5** are essential to high affinity of the glycomimetic scaffold to the binding site from an energetic perspective.

**Table 1:** Docking scores for sulfated and non-sulfated glycomimetic analogues. Key: sulfate groups (red); alcohol groups (blue)

Entry	Code	Docked structure	Docking score (kJ mol^−1^)
1	*R-**10***	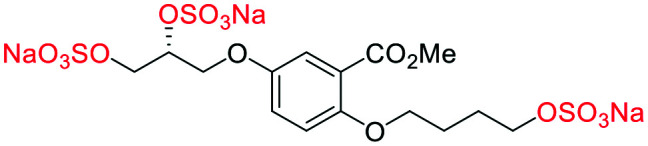	−41.0
2	*S-**10***	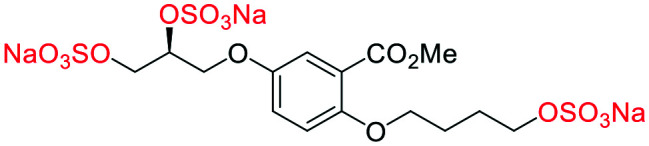	−42.0
3	α-**4**	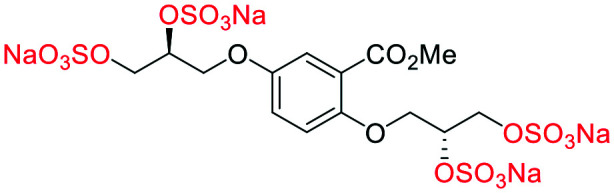	−47.4
4	β-**5**	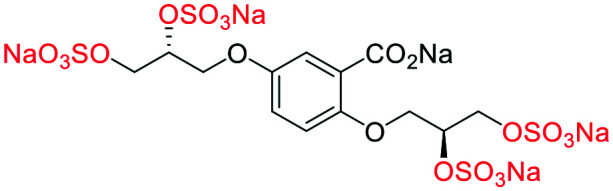	−51.4
5	**26**	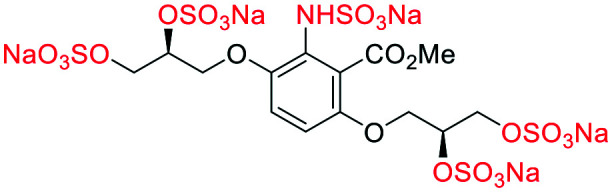	−47.7
6	α-**2**	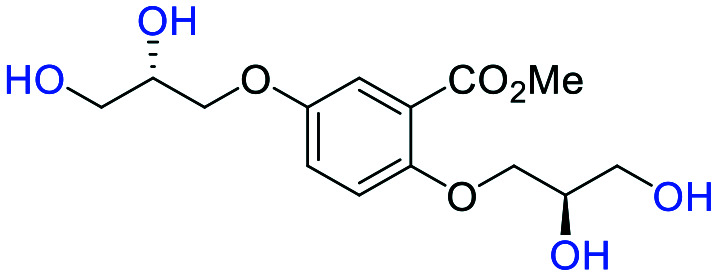	−9.2
7	**17f**	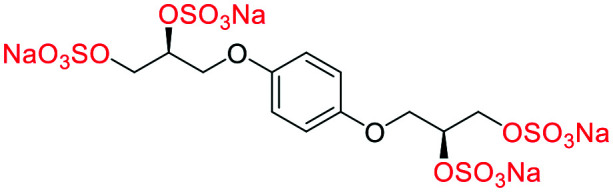	−45.8
8	β-**3**	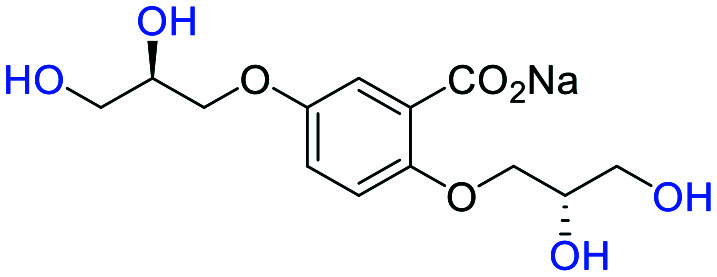	−19.2
9	α-**3**	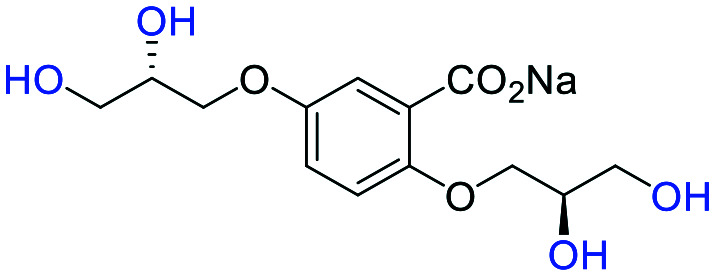	−15.8
10	**17g**	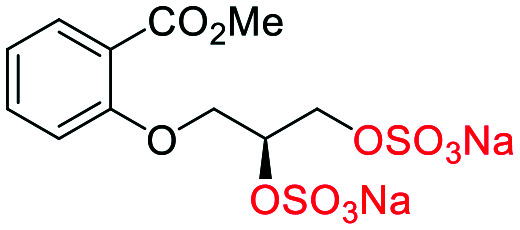	−36.9
11	**17h**	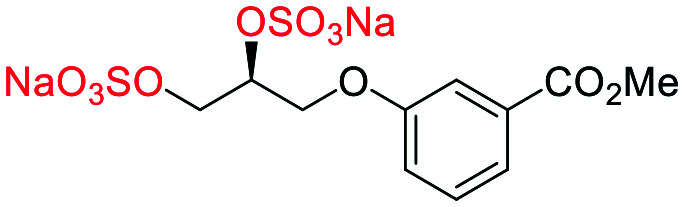	−43.6
12	**17i**	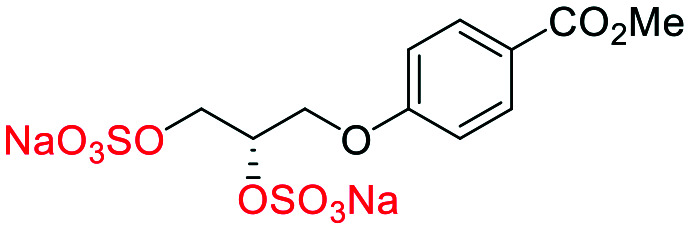	−38.3
13	**17a**	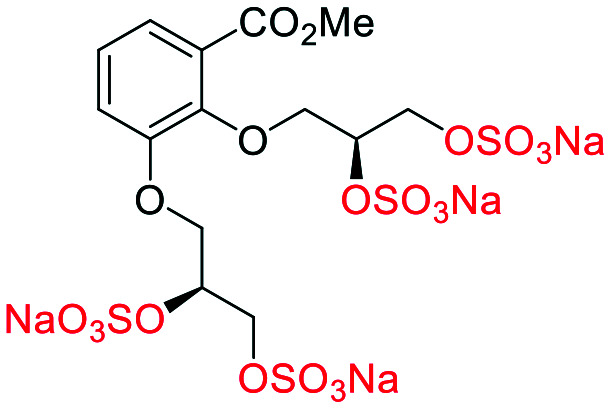	−51.2
14	**17b**	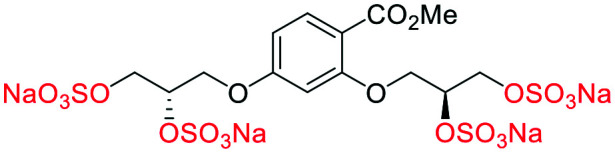	−47.3
15	**17c**	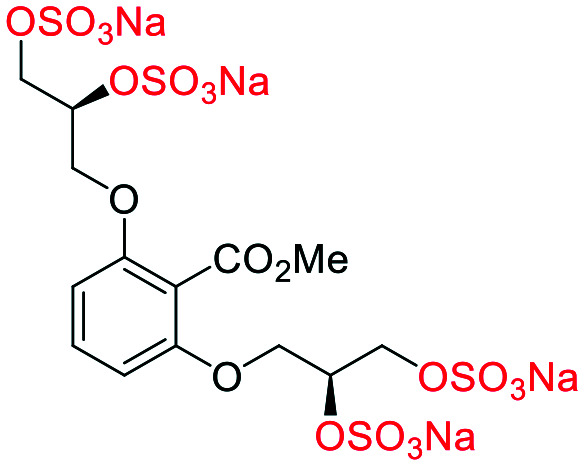	−42.8
16	**17d**	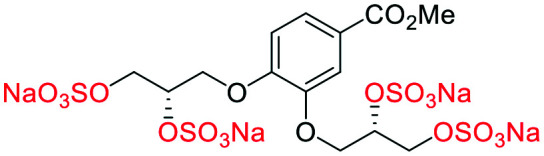	−47.4
17	**17e**	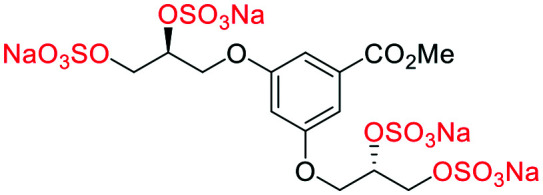	−45.4

In the case of the desulfated α-**2**, four hydrogen bonds were observed with Lys58 and Ala56, such interactions likely play an important role in the stabilization of the complex formed by α-**2** and unlike the other molecules α-**2** had a stronger influence through van der Waals interactions than electrostatic.

## Is the absolute stereochemistry critical to the binding of HGF?

As heparan sulfate is a larger, linear polysaccharide chain containing numerous chiral centres, with some degree of flexibility of iduronic acid motifs, we considered whether the stereochemical positioning of the sulfate vectors on our more rigid aromatic core influenced the binding of the heparan sulfate-glycomimetics to HGF *in silico*.

**Scheme 4:**
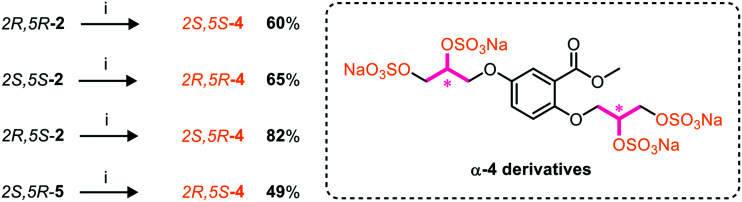
A chiral pool synthesis of α-**4**, synthesising each stereochemical combination. Conditions: i) 1) Bu_3_N·SO_3_, MeCN, 90 °C, 2) sodium 2-ethylhexanoate, ^*t*^BuOMe/EtOH.

A chiral pool method^18^ was employed to access all four possible stereochemical combinations of α-**4**. Each stereo-defined tetraol (Scheme 4) was in turn sulfated with TBSAB, affording the corresponding persulfated glycomimetic (α-**4**) in 49–82% isolated yield as their tetrakis sodium salts.

A computational docking study was performed on each potential stereoisomer of **4** to determine if the binding interactions of the heparan sulfate-glycomimetics are affected by single point changes in chirality. The results of the computational docking study suggested that each stereoisomer binds to HGF with similar energies (within 2.9 kJ mol^−1^). Docking results calculated that the enantiomer 2*S*,5*S*-**4** to have the strongest energy binding interaction with HGF. Furthermore, the enantiomer 2*R*,5*R*-**4** was calculated to have the weakest binding energy. A difference of ±2.9 kJ mol^−1^ suggests that non-electrostatic intermolecular forces, such as van der Waals interactions, have minimal effects on the free energy binding interaction. The diastereomers 2*S*,5*S*-**4**/2*R*,5*S*-**4** have closely matched energies (±1.6 kJ mol^−1^). A single point change in stereochemistry at the 2-position had an associated decrease in hydrogen bonding, suggesting a non-optimal orientation of the substituent.

Overall, the results gained by this docking study propose that the heparan sulfate-glycomimetic's binding to HGF are not substantially determined by their inherent asymmetry, which is in agreement with the near-equipotent biological results gained from the first generation of asymmetric heparan sulfate-glycomimetics, **C1–C4**.^14^

Furthermore, from the results of our initial *in silico* study, this investigation decided that a stereocontrolled synthesis was not imperative in the design of further generations of heparan sulfate-glycomimetics targeting HGF. Therefore, the design of a second generation of heparan sulfate-glycomimetics focussed on the regioisomeric orientation of anionic sulfate groups around an aromatic core structure.

## Computational SAR of second generation glycomimetics

For synthetic details of the preparation of racemic second generation regioisomeric glycomimetics see Schemes S1–S3.† To streamline the docking study of all the following second generation sulfated glycomimetics, the *nS*,*nS* or *S*-enantiomers from the racemates were docked against NK1 (HGF) using a globally parametrized training set of structures. A series of point changes were made to the core structure of **4**, as follows: converting the methyl ester to the carboxylic acid (β-**5**), resulted in an increased affinity (β-**5** −51.4 *vs.* α-**4** −47.4 kJ mol^−1^, Table 1); deletion of the methyl ester, led to a modest loss of affinity (**17f** −45.8 *vs.* α-**4** −47.4 kJ mol^−1^). Removal of the sulfate units from either the methyl ester (α-**4**) or the carboxylic acid (β-**5**), led to a dramatic loss of affinity for NK1 (α-**2** −9.2 *vs.* α-**4** −47.4 kJ mol^−1^ and β-**3** −19.2 or α-**3** −15.8 *vs.* β-**5** −51.4 kJ mol^−1^).

We next considered whether the 2,5-regioisomer of the sulfated glycols around the benzoate core was optimal (Table 1). The results of this study suggest that altering the orientation of sulfate groups *via* the investigation of regioisomers around an aromatic framework have little effect on the binding interactions with HGF and thus the 2,5-regioisomers were progressed to further experimental evaluation.

We next considered what is driving the interaction of α-**4** with HGF, is it the 2 or 5-sulfated glycol motif (Table 1). The removal of an entire sulfated glycol side chain afforded disulfates **17g–i** (Table 1). The structurally smaller disulfates **17g–i** (−40% by molecular mass compared to the tetrasulfates), however only an on average −16.5% drop in binding energy, suggesting the that the disulfates are likely to be more efficient at binding HGF than the tetrasulfates (**17a–f**). A rationale for this difference between the tetrasulfates and disulfates, is that an optimal interaction with the first disulfate chain may compromise an efficient binding event for the second disulfate chain at an orthosteric position.

Considering heparan sulfate has many non-sulfated regions in its macrostructure, and non-ionic interaction can account for up to 70% of the binding free energy,^25^ it is proposed that the interactions of endogenous heparan sulfate with HGF do not rely predominantly on anionic charge density from multiple sulfate functionalities. Taken together these results elucidate a valuable SAR profile for the glycomimetic manifold.

Overall, the results of the computational study propose that the heparan sulfate-glycomimetics bind HGF predominantly through electrostatic interactions. However, the results also highlight the potential significance of the methyl ester and point chirality to the binding interaction of HGF.

## Molecular dynamic binding simulation studies of key sulfated and desulfated glycomimetics

**Fig. 4:**
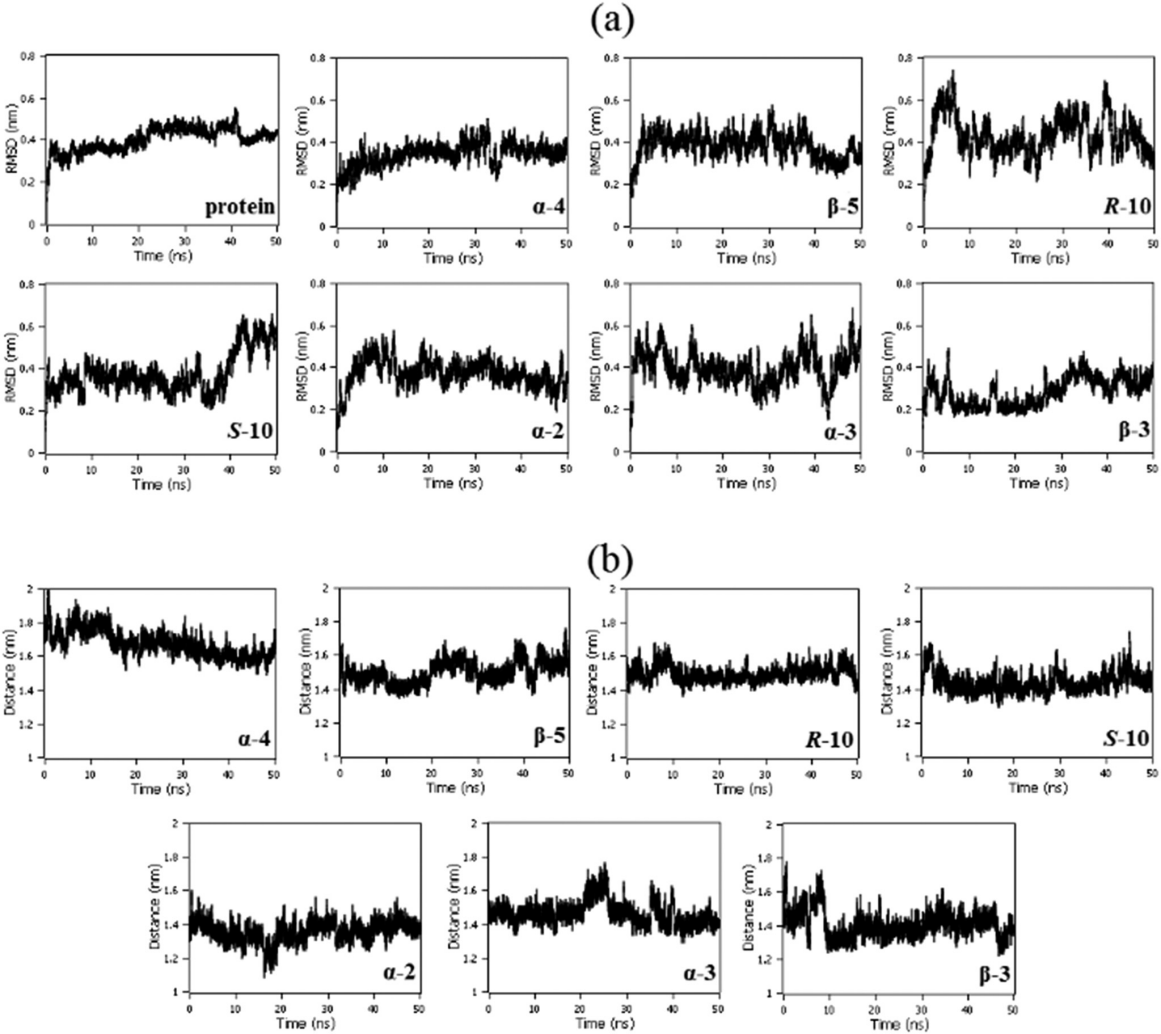
(a) RMSD of the protein backbone along 50 ns of molecular dynamics in the presence of ligands: α-**4**; β-**5**; *R*-**10**; *S*-**10**; α-**2**; α-**3**; and β-**3**; (b) distance between COG of domain 1 and COG of ligands: α-**4**; β-**5**; *R*-**10**; *S*-**10**; α-**2**; α-**3**; and β-**3**.

Root-mean-squared deviation (RMSD) analyses showed the stability of the protein structure along 50 ns of molecular dynamics in the presence of each triaged 2,5-regioisomeric glycomimetic scaffold investigated. It is notable that the glycomimetics did not cause any abrupt movements in the protein, indicating that NK1 remained stable in the presence of the glycomimetics. The protein remained stable after 10 ns of simulation in the presence of the molecules with small fluctuations (Fig. 4a). The distance between the centers of geometry (COG) of the N-domain with each glycomimetic structure remained stable with small fluctuations, indicating that the molecules remained in the binding site along 50 ns of molecular dynamics (Fig. 4b).

## Biological evaluation

The assays focused on the production of nitric oxide (NO) and the upregulation of nitric oxide synthase (eNOS), the production of reactive oxygen species (ROS) and the activity of nicotinamide adenine dinucleotide phosphate (NADPH) oxidase (Table 2). From the hypothesised model of heparan sulfate-glycomimetic activity,^17^ the glycomimetics are proposed to attenuate the binding of HGF to the cMET receptor. This disruption to MET-receptor activation is proposed to modulate intracellular phosphorylation leading to an upregulation of eNOS and increased output of extracellular NO. Furthermore, the mechanism of action has also been demonstrated to modulate the enzymes: superoxide dismutase (SOD), catalase (CAT) and glutathione peroxidase (GPx), which quench intracellular ROS using NADPH as cofactor. Overall, the cross examination of NO output and reduction of ROS was used to determine the therapeutic action of the triaged glycomimetics α-**4**, *R*-**10**, *S*-**10** and β-**5**, as well as 3 non-sulfated intermediates α-**2**, α-**3** and β-**3**, on free fatty acid (FFA) induced, oxidatively stressed endothelial cell cultures (Table 2).

**Table 2:** Biological data for eNOS and ROS levels. All data are averaged % of increase (+) or decrease (−) compared to control (FFA induced oxidatively stressed endothelial cells) (*n* = 3)

Entry	Compound number	NO production	eNOS phosphorylation	ROS production	NADPH oxidase activity
1	α-**4**	+110	+342	−28	−29
2	β-**5**	+62	+318	−29	−31
3	*S*-**10**	+63	+397	−39	−27
4	*R*-**10**	+52	+613	−23	−31
5	α-**3**	+41	+312	−23	−21
6	β-**3**	+52	+294	−19	−27
7	α-**2**	+46	+286	−36	−26

The results of the biological assays demonstrate similar trends across each compound (Table 2). Administration of the heparan sulfate-glycomimetics and non-sulfated intermediates generates an increase in eNOS phosphorylation with an associated output of NO. Furthermore, each compound demonstrated a correlation in the reduction of ROS and NADPH oxidase activity. Specifically, for the heparan sulfate-glycomimetic α-**4** (Table 2, entry 1), the concentration of NO increase by +110% compared to the control. However, the increased phosphorylation of eNOS is comparable to the other compounds tested, with the exception of *R*-**10**, which is shown to increase eNOS phosphorylation significantly greater than all the other compounds tested (+613%, Table 2, entry 4). All the remaining compounds demonstrated similar biological activity.

## ADMET data on hit glycomimetics

The triaged glycomimetics have been independently analysed for their ADMET properties (Table 3). All showed high kinetic solubility, no cytotoxic effect in Hep G2 cells, long half-life in mouse liver microsomes (MLMs) and rat liver microsomes (RLMs) and due to the compound stability, low levels of intrinsic clearance (CL_int_). Furthermore, no inhibition of the cardiac ion channel, hERG was observed. As expected, Caco-2 cell permeability levels were low or not detected due to the highly polar nature of β-**5** and *R*-**10** lending support to the cell receptor interaction proposed. A potential concern is CYP-mediated metabolic oxidation due to the *para*-arrangement of the phenolic groups in the 2,5-substituted glycomimetics, potentially leading to quinone-type reactive intermediates or loss of the sulfate groups, was not observed in the examples tested.

Physicochemical and ADMET properties of representative glycomimetics. NR = no response, ND = not determined, MEC = minimum effective concentration that significantly crosses vehicle control threshold, AC_50_ = the concentration at which 50% maximum effect is observed for each cell health parameter. Kinetic solubility measured with a turbidimetric assay (controls: nicardipine and pyrene). MTT (cytotoxicity) study using HepG2 cells (Cyprotex, Macclesfield, UK; controls: carbonyl cyanide 3-chlorophenylhydrazone and chlorpromazine). MLM assay (controls: diphenhydramine and diazepam). hERG assay (controls: quinidine and dimethylsulfoxide). Caco-2 assay (ATCC, Virginia, USA; controls: atenolol, propranolol, and talinolol)

**Table:** 

Compounds	α-**4**	β-**5**	*R*-**10**	*S*-**10**
Parameters				
Molecular weight (Da)	723.87	731.83	619.95	619.95
tPSA (Å^2^)	299	310	235	235
clog *P*	−1.44	−1.96	−0.13	−0.13
Number of HBAs	20	20	16	16
Number of HBDs	4	5	3	3
Chemical stability	Yes	Yes	Yes	Yes

**Table:** 

Assay results				
Kinetic solubility (μM)	>100	>100	>100	>100
HUVEC cell viability (1–500 μM)	NR	NR	NR	NR
MTT (MEC, μM)	NR	NR	NR	NR
MTT (AC_50_, μM)	NR	NR	NR	NR
MLM *t*_1/2_ (min)	1840	482	3450	4160
Mouse CL_int_ (μL min^−1^ mg^−1^)	0.755	2.88	0.402	0.333
RLM *t*_1/2_ (min)	145	2140	338	109
Rat CL_int_ (μL min^−1^ mg^−1^)	9.56	0.649	4.11	12.8
hERG	ND	>25 μM	>25 μM	ND
Caco-2 (B_2_A *P*_app_ 10^−6^ cm s^−1^)	ND	Not detected	Not detected	ND
Caco-2 (A_2_B *P*_app_ 10^−6^ cm s^−1^)	ND	Not detected	0.931	ND

## Conclusions

We have identified a promising series of persulfated and non-sulfated small molecule heparan sulfate-glycomimetics that elicit a protective effect in our simulated lipid-induced endothelial dysfunction model. All examples tested demonstrate an increase in NO production after oxidative stress through the eNOS phosphorylation biomarker, and significant decrease in reactive oxygen species (ROS) production through NADPH oxidase activity knock-down. Significantly higher protective effects were observed in the sulfated glycomimetic series compared to the desulfated precursors.

Therefore, coupled with these heparan sulfate-glycomimetics encouraging ADMET profiles (chemically stable, non-toxic against HUVECs, MTT and hERG; excellent water solubility; and long *t*_1/2_), modelled interaction profile through molecular docking to HGF and simulated dynamics studies demonstrate these heparan sulfate-glycomimetics as a new source of bespoke mimics for ongoing hit-to-lead studies targeting inflammation and cardiovascular disease.

## Experimental

### Selected compound characterisation

All general procedures can be located in the ESI.†

#### (α-**2**) methyl 5-((*R*)-2,3-dihydroxypropoxy)-2-((*S*)-2,3-dihydroxypropoxy)benzoate

##### Adapted from general procedure **D**^26^ using stock solutions

**I** (2 mL, 3.0 mmol), **II** (2 mL, 3.0 mmol), **III** (1.0 mL, 2.0 μmol) and **IV** (5.0 mL, 20.0 μmol).^27^ Methyl 2,5-bis(allyloxy)benzoate (**1**) (158 mg, 0.50 mmol) was added and the reaction mixture was stirred at 0 °C for 6 h with monitoring (EtOH/EtOAc 1 : 4, *R*_f_ = 0.20). The solvent was removed under reduced pressure and the crude mixture was directly purified by chromatography (SiO_2_, EtOAc/hexane 1 : 4) to afford the title compound as a clear oil (156 mg, 99%). **[*α*]**_**D**_^**25**^ −13.15 (c. 1.0, MeOH, 36 : 6 : 4 : 54 *e.r*/*d.r* (2*R*,5*R*; 2*S*,5*S*; 2*R*,5*S*; 2*S*,5*R*)); **IR***ν*_max_ cm^−1^ 3268 w, 2939 w, 2890 w, 1699 s (C=O), 1601 w, 1499 s, 1435 w; ^**1**^**H-NMR** (400 MHz, (CD_3_)_2_SO) *δ*_H_ 7.18 (d, *J* = 2.7 Hz, 1H, C6–H̲), 7.15–7.04 (m, 2H, C3–H̲ & C4–H̲), 4.94 (d, *J* = 5.0 Hz, 1H, CH–OH̲), 4.84 (d, *J* = 5.0 Hz, 1H, CH–OH̲), 4.66 (t, *J* = 5.7 Hz, 1H, CH_2_–OH̲), 4.59 (t, *J* = 5.7 Hz, 1H, CH_2_–OH̲), 4.01–3.85 (m, 3H), 3.86–3.69 (m, 6H, Me), 3.55–3.35 (m, 4H); ^**13**^**C-NMR** (101 MHz, (CD_3_)_2_SO) *δ*_C_ 166.0 (C̲O_2_Me), 152.2 (C5), 152.0 (C2), 121.0 (C1), 119.8 (C3/C4), 115.94 (C3/C4), 115.89 (C6), 71.3 (ArO–C̲H_2_), 70.3 (ArO–C̲H_2_), 69.9 (2C, C̲H–OH), 62.72 (CH̲_2_–OH), 62.66 (CH̲_2_–OH), 52.0 (Me); **LRMS***m*/*z* (ESI+) 339.12 (100%, [M + Na]^+^); **HRMS***m*/*z* (ESI+) C_14_H_20_O_8_Na requires: 339.1056, found: 339.1057 ([M + Na]^+^).

#### (α-**3**) sodium 5-((*R*)-2,3-dihydroxypropoxy)-2-((±)-2,3-dihydroxypropoxy)benzoate

A solution of methyl 5-((*R*)-2,3-dihydroxypropoxy)-2-((±)-2,3-dihydroxypropoxy)benzoate (α-**2**) (100 mg, 0.32 mmol) in MeOH (10 mL) was charged with NaOH (13 mg, 0.32 mmol). The reaction mixture was heated under reflux for 2 h, then cooled to room temperature. The solvent was removed under reduced pressure to afford the title compound as a hygroscopic white solid (103 mg, 99%). **[*α*]**_**D**_^**25**^ −10.48 (c. 1.0, MeOH, 36 : 6 : 4 : 54 *e.r*/*d.r* (*RR*,*SS*,*RS*,*SR*)); **IR***ν*_max_ cm^−1^ 3246 br s (O–H), 2934 w, 2877 w, 1559 s (C=O), 1491 s, 1455 w, 1420 s, 1374 s; ^**1**^**H-NMR** (400 MHz, CD_3_OD) *δ*_H_ 7.36 (d, *J* = 3.1 Hz, 1H, C6–H̲), 7.15 (dd, *J* = 9.0, 3.1 Hz, 1H, C4–H̲), 7.12–7.05 (m, 2H, C6–H̲ & C3–H̲), 6.93 (d, *J* = 9.0 Hz, 1H, C3–H̲), 6.87 (dd, *J* = 9.0, 3.0 Hz, 1H, C4–H̲), 4.16–3.86 (m, 2 × 6H), 3.79–3.57 (m, 2 × 4H); ^**13**^**C-NMR** (101 MHz, CD_3_OD) *δ*_C_ 175.9, 154.4, 154.34, 154.25, 151.7, 132.8, 122.0, 121.5, 117.8, 117.2, 116.7, 116.6, 116.2, 73.2, 72.8, 71.9, 71.8 (2C), 71.6, 71.1, 70.9, 64.3, 64.2, 64.1, 63.9; **LRMS***m*/*z* (ESI+) 325.09 (30%, [M + H]^+^); **HRMS***m*/*z* (ESI+) C_13_H_18_NaO_8_ requires: 325.0894, found: 325.0895 ([M + H]^+^).

#### (β-**3**) sodium 5-((*S*)-2,3-dihydroxypropoxy)-2-((±)-2,3-dihydroxypropoxy)benzoate

A solution of methyl 5-((*S*)-2,3-dihydroxypropoxy)-2-((±)-2,3-dihydroxypropoxy)benzoate (β-**2**) (100 mg, 0.32 mmol) in MeOH (10 mL) was charged with NaOH (13 mg, 0.32 mmol). The reaction mixture was heated under reflux for 2 h, then cooled to room temperature. The solvent was removed under reduced pressure to afford the title compound as a hygroscopic white solid (103 mg, 99%). **[*α*]**_**D**_^**25**^ −8.31 (c. 1.0, MeOH, 1 : 51 : 46 : 2 *e.r*/*d.r* (*RR*,*SS*,*RS*,*SR*)); **IR***ν*_max_ cm^−1^ 3249 br s (O–H), 2934 w, 2879 w, 1559 s (C=O), 1491 s, 1455 w, 1420 s, 1374 s; ^**1**^**H-NMR** (400 MHz, CD_3_OD) *δ*_H_ 7.36 (d, *J* = 3.1 Hz), 7.15 (dd, *J* = 8.9, 3.1 Hz), 7.12–7.07 (m, 1H, C6–H̲), 6.93 (d, *J* = 8.9 Hz, 1H, C3–H̲), 6.87 (dd, *J* = 8.9, 3.1 Hz, 1H, C4–H̲), 4.20–3.84 (m, 2 × 6H), 3.78–3.55 (m, 2 × 4H); ^**13**^**C-NMR** (101 MHz, CD_3_OD) *δ*_C_ 176.4 (C̲O_2_Me), 154.9, 154.9, 154.8, 152.3, 133.3, 122.0, 118.4, 117.7, 117.3, 117.12 (2C), 116.8, 116.7, 73.7, 73.3, 72.4, 72.3, 72.1, 71.6, 71.4, 64.8, 64.7, 64.6, 64.5; **LRMS***m*/*z* (ESI+) 325.09 (30%, [M + H]^+^); **HRMS***m*/*z* (ESI+) C_13_H_18_NaO_8_ requires: 325.0894, found: 325.0890 ([M + H]^+^).

#### (α-**4**) sodium 3-(4-((*S*)-2,3-bis(sulfonatooxy)propoxy)-2-(methoxycarbonyl)phenoxy)propane-1,2-diyl bis(sulfate)

##### Adapted from general procedure **E**^28^

A 25 mL Schlenk tube was charged with α-**5** (81 mg, 0.25 mmol) and Bu_3_N·SO_3_ (530 mg, 2.00 mmol) under Ar and MeCN was added (0.5 mL). The flask was heated at 80 °C for 12 h with monitoring (TLC). The flask was cooled to room temperature and the solvent removed under reduced pressure to give a clear viscous oil. The crude oil was dissolved in ^i^PrOH (5 mL) and transferred to a flask containing ^*t*^BuOMe (35 mL). With vigorous stirring a solution of NEH (5.0 mL, 1.0 M) in ^*t*^BuOMe/^i^PrOH (1 : 7) was added dropwise over 10 min. The precipitate that formed was collected by filtration, washed with ^i^PrOH (2 × 10 mL) and dried under vacuum. Recrystallization from H_2_O/^i^PrOH afforded the title compound as a white solid (160 mg, 88%). **[*α*]**_**D**_^**25**^ −3.35 (c. 1.0, H_2_O, 36 : 6 : 4 : 54 *e.r*/*d.r* (*SS*,*RR*,*SR*,*RS*)); **M.P** 198–200 °C (dec.); **IR***ν*_max_ cm^−1^ 2988 w, 2164 w, 1711 w (C=O), 1500 w, 1443 w, 1221 s (S=O), 1131 s (S=O); ^**1**^**H-NMR** (400 MHz, D_2_O) *δ*_H_ 7.45 (d, *J* = 3.1 Hz, 1H, C6–H̲), 7.27 (dd, *J* = 9.1, 3.1 Hz, 1H, C4–H̲), 7.19 (d, *J* = 9.1 Hz, 1H, C3–H̲), 4.87 (td, *J* = 4.8, 1.8 Hz, 2H, CH̲–OSO_3_Na), 4.45–4.25 (m, 8H, Ar–OCH̲_2_, CH̲_2_–OSO_3_Na), 3.93 (s, 3H, Me); ^**13**^**C-NMR** (101 MHz, D_2_O) *δ* 168.5 (C̲O_2_Me), 152.2 (C2), 152.1 (C5), 121.4 (C4), 120.8 (C1), 117.4 (C6), 116.9 (C3), 75.03 (C̲H–OSO_3_Na), 74.98 (C̲H–OSO_3_Na), 68.2 (O–C̲H_2_), 67.3 (O–C̲H_2_), 66.44 (C̲H_2_–OSO_3_Na), 66.41 (C̲H_2_–OSO_3_Na), 52.8 (Me); **LRMS***m*/*z* (ESI+) 746.86 (20%, [M + Na]^+^); **HRMS***m*/*z* (ESI+) C_14_H_16_Na_5_O_20_S_4_ requires: 746.8601, found: 746.8591 ([M + Na]^+^).

#### (β-**5**) sodium 5-((*R*)-2,3-bis(sulfonatooxy)propoxy)-2-(2,3-bis(sulfonatooxy)propoxy)benzoate

##### Adapted from general procedure **E**^28^

A 25 mL Schlenk tube was charged with β-**3** (100 mg, 0.32 mmol) and Bu_3_N·SO_3_ (678 mg, 2.56 mmol) under Ar_(g)_ and MeCN was added (0.6 mL). The flask was heated at 80 °C for 12 h and with monitoring (TLC). The flask was cooled to room temperature and the solvent removed under reduced pressure to give a clear viscous oil. The crude oil was dissolved in ^i^PrOH (5 mL) and transferred to a flask containing ^*t*^BuOMe (35 mL). With vigorous stirring a solution of NEH (5.0 mL, 1.0 M) in ^*t*^BuOMe/^i^PrOH (1 : 7) was added dropwise over 10 min. The precipitate that formed was collected by filtration, washed with ^i^PrOH (2 × 10 mL) and dried under vacuum. Recrystallization from H_2_O/^i^PrOH afforded the title compound as a white solid (200 mg, 86%). **[*α*]**_**D**_^**25**^ −1.66 (c. 1.0, H_2_O, 1 : 51 : 46 : 2 *e.r*/*d.r* (*SS*,*RR*,*SR*,*RS*)); **M.P** 200–202 °C (dec.); **IR***ν*_max_ cm^−1^ 2988 w, 1712 w (C=O), 1499 w, 1435 w, 1100 s (S=O); ^**1**^**H-NMR** (400 MHz, D_2_O) *δ* 7.47–6.94 (m, 3H), 5.00–4.73 (m, 2H), 4.46–3.99 (m, 8H); ^**13**^**C-NMR** (101 MHz, D_2_O) *δ* 169.6 (C̲O_2_Na), 152.3 (C̲–O), 151.8 (C̲–O), 121.5 (C̲–CO_2_Na), 120.7 (C̲–H), 117.4 (C̲–H), 116.2 (C̲–H), 75.0 (C̲–H), 74.9 (C̲–H), 68.3 (C̲–H_2_), 67.3 (C̲–H_2_), 66.6 (C̲–H_2_), 66.4 (C̲–H_2_); **LRMS***m*/*z* (ESI+) 732.84 (10%, [M + H]^+^); **HRMS***m*/*z* (ESI+) C_13_H_14_Na_5_O_20_S_4_ requires: 732.8444, found: 732.8439 ([M + H]^+^).

#### (*R*-**10**) sodium (*R*)-3-(3-(methoxycarbonyl)-4-(4-(sulfonatooxy)butoxy)phenoxy)propane-1,2-diyl bis(sulfate)

##### Adapted from general procedure **E**^28^

A 25 mL Schlenk tube was charged with *S*-**9** (135 mg, 0.45 mmol) and Bu_3_N·SO_3_ (722 mg, 2.72 mmol) under Ar_(g)_ and MeCN was added (1.5 mL). The flask was heated at 80 °C for 12 h with monitoring (TLC). The flask was cooled to room temperature and the solvent removed under reduced pressure to give a clear viscous oil. The crude oil was dissolved in ^i^PrOH (5 mL) and transferred to a flask containing ^*t*^BuOMe (35 mL). With vigorous stirring a solution of NEH (5.0 mL, 1.0 M) in ^*t*^BuOMe/^i^PrOH (1 : 7) was added dropwise over 10 min. The precipitate that formed was collected by filtration, washed with ^i^PrOH (2 × 10 mL) and dried under vacuum. Recrystallization from H_2_O/^i^PrOH afforded the title compound as a white solid (260 mg, 94%). **[*α*]**_**D**_^**25**^ −3.11 (c. 1.0, CHCl_3_); **M.P** 207–210 °C (dec.); **IR***ν*_max_ cm^−1^ 2972 w, 2166 w, 1703 w (C=O), 1500 w, 1441 w, 1103 s (S=O); ^**1**^**H-NMR** (400 MHz, D_2_O) *δ*_H_ 7.42 (d, *J* = 3.2 Hz, 1H, C6–H̲), 7.25 (dd, *J* = 9.2, 3.2 Hz, 1H, C4–H̲), 7.14 (d, *J* = 9.2 Hz, 1H, C3–H̲), 4.86 (p, *J* = 4.8 Hz, 1H, CH̲–OSO_3_Na), 4.42–4.29 (m, 3H), 4.26 (dd, *J* = 11.0, 5.2 Hz, 1H), 4.16–4.05 (m, 4H), 3.90 (s, 3H, Me), 1.86 (m, 4H, (CH̲_2_)_2_); ^**13**^**C-NMR** (101 MHz, D_2_O) *δ*_C_ 168.4 (CO̲_2_Me), 152.6 (C2), 151.7 (C5), 121.6 (C4), 120.1 (C1), 117.3 (C6), 116.4 (C3), 75.0 (C̲H–OSO_3_Na), 69.7, 69.0, 67.3, 66.4, 52.7 (Me), 25.2 ((C̲H_2_)_2_), 24.9 ((C̲H_2_)_2_); **LRMS***m*/*z* (ESI+) 642.94 (40%, [M + Na]^+^); **HRMS***m*/*z* (ESI+) C_15_H_19_Na_4_O_16_S_3_ requires: 642.9421, found: 642.9426 ([M + Na]^+^).

#### (*S*-**10**) sodium (*S*)-3-(3-(methoxycarbonyl)-4-(4-(sulfonatooxy)butoxy)phenoxy)propane-1,2-diyl bis(sulfate)

##### Adapted from general procedure **E**^28^

A 25 mL Schlenk tube was charged with *R*-**9** (215 mg, 0.72 mmol) and Bu_3_N·SO_3_ (1.145 g, 2.56 mmol) under Ar_(g)_ and MeCN was added (1.5 mL). The flask was heated at 80 °C for 12 h with monitoring (TLC). The flask was cooled to room temperature and the solvent removed under reduced pressure to give a clear viscous oil. The crude oil was dissolved in ^i^PrOH (5 mL) and transferred to a flask containing ^*t*^BuOMe (35 mL). With vigorous stirring a solution of NEH (5.0 mL, 1.0 M) in ^*t*^BuOMe/^i^PrOH (1 : 7) was added dropwise over 10 min. The precipitate that formed was collected by filtration, washed with ^i^PrOH (2 × 10 mL) and dried under vacuum. Recrystallization from H_2_O/^i^PrOH afforded the title compound as a white solid (430 mg, 97%). **[*α*]**_**D**_^**25**^ +3.01 (c. 1.0, H_2_O); **M.P** 207–210 °C (dec.); **IR***ν*_max_ cm^−1^ 2971 w, 2164 w, 1710 w (C=O), 1500 w, 1441 w, 1103 s (S=O); ^**1**^**H-NMR** (400 MHz, D_2_O) *δ*_H_ 7.43 (d, *J* = 3.2 Hz, 1H, C6–H̲), 7.25 (dd, *J* = 9.2, 3.2 Hz, 1H, C4–H̲), 7.14 (d, *J* = 9.2 Hz, 1H, C3–H̲), 4.87 (p, *J* = 4.7 Hz, 1H, CH̲–OSO_3_Na), 4.44–4.29 (m, 3H), 4.26 (dd, *J* = 11.0, 5.2 Hz, 1H), 4.12 (m, 4H), 3.90 (s, 3H, Me), 1.94–1.79 (m, 4H, (CH̲_2_)_2_); ^**13**^**C-NMR** (101 MHz, D_2_O) *δ*_C_ 168.4 (CO̲_2_Me), 152.6 (C2), 151.7 (C5), 121.6 (C4), 120.1 (C1), 117.3 (C6), 116.4 (C3), 75.0 (C̲H–OSO_3_Na), 69.7, 69.0, 67.4, 66.4, 52.7 (Me), 25.2 ((C̲H_2_)_2_), 24.9 ((C̲H_2_)_2_); **LRMS***m*/*z* (ESI+) 642.94 (50%, [M + Na]^+^); **HRMS***m*/*z* (ESI+) C_15_H_19_Na_4_O_16_S_3_ requires: 642.9421, found: 642.9417 ([M + Na]^+^).

## Conflicts of interest

A. M. J. and M. Y. A. have a commercial interest in glycomimetics (UK Patent Application No. 1515850.4 and PCT Patent Application No. PCT/GB2016/052764).

## Supplementary Material

MD-012-D0MD00366B-s001

MD-012-D0MD00366B-s002
