# Supplementary material for: The modulatory role of sulfated and non-sulfated small molecule heparan sulfate-glycomimetics in endothelial dysfunction: absolute structural clarification, molecular docking and simulated dynamics, SAR analyses and ADMET studies
Source: RSC Med Chem. 2021 Apr 23;12(5):779–90. doi: 10.1039/d0md00366b (PMC8152814; doi:10.1039/d0md00366b)
Supplement: MD-012-D0MD00366B-s001 [file MD-012-D0MD00366B-s001.pdf]

Electronic Supplementary Material (ESI) for RSC Medicinal Chemistry.
This journal is © The Royal Society of Chemistry 2021

SUPPORTING INFORMATION

The modulatory role of sulfated and non-sulfated small molecule heparan sulfate-glycomimetics in endothelial dysfunction: Absolute structural clarification, molecular docking and simulated dynamics, SAR analyses and ADMET studies

Daniel M. Gill,[1] Ana Paula R. Povinelli,[1,2] Gabriel Zazeri,[1,2] Sabrina A. Shamir,[3] Ayman M. Mahmoud,[4,5,6] Fiona L. Wilkinson,[6] M. Yvonne Alexander,[6] Marinonio L. Cornelio,[2] and Alan M. Jones[1]*

[1] School of Pharmacy, University of Birmingham, Edgbaston, B15 2TT, United Kingdom

[2] Departamento de Física – IBILCE, Rua Cristovão Colombo, 2265 CEP 15054-000 São José do Rio Preto - São Paulo, Brazil

[3] Department of Natural Sciences, Manchester Metropolitan University, M1 5GD, UK

[4] Physiology Division, Department of Zoology, Faculty of Science, Beni-Suef University, Egypt.

[5] Department of Endocrinology, Diabetes & Nutrition, Center for Cardiovascular Research (CCR), Charité - Universitätsmedizin Berlin, Berlin, Germany.

[6] Centre for Biomedicine, Manchester Metropolitan University, M1 5GD, UK.

*corresponding author: A.M.J.; Tel: +44(0)121-414-7288; e-mail: a.m.jones.2@bham.ac.uk

Contents

General experimental details	pages 3-4
General synthetic procedures	pages 5-8
Biological assay and ADMET experimental details	pages 9-15
Molecular docking and simulation details	pages 16-17
Compound characterisation	pages 18-50
NMR spectra of compounds	page 51-161
Crystallographic data	pages 162-164
Supplementary figures and tables	pages 165-168
Additional Supporting Synthetic Schemes	pages 169-170
References	pages 171-172

General Experimental Methods

All reactions involving moisture sensitive reagents were carried out in oven-dried reaction vessels under a nitrogen or argon atmosphere. All anhydrous solvents were directly obtained from an SPS dispensary or were dried over 4 Å molecular sieves for 24 h prior to use. Solvents used for work up procedures and column chromatography were of technical grade from Honeywell, VWR and Fischer Scientific. Unless stated otherwise, solvents were removed by rotary evaporation under a reduced pressure between 30-50 ˚C. All chemical reagents were used as received unless stated otherwise.

The progress of reactions was monitored by thin layer chromatography (TLC) using Merck® silica gel 60 F254 plates, which were visualized with UV light and potassium permanganate. Flash column chromatography was carried out using Geduran 60 Å silica gel and the indicated solvent systems.

^{1}H- and ^{13}C-NMR spectra were recorded on a Bruker Avance® III (400 MHz) spectrometer at 400 and 101 MHz, respectively. Chemical shift data are reported in parts per million (ppm, δ scale) downfield from tetramethylsilane (TMS: δ 0.0), and referenced internally to the residual solvent signal. The deuterated solvents used for NMR analysis were: chloroform ($CDCl_3$: δ_H 7.26, δ_C 77.16), dimethyl sulfoxide ($(CD_3)_2SO$: δ_H 2.50, δ_C 39.52), acetone ($(CD_3)_2CO$: δ_H 2.05, δ_C 29.84), methanol (CD_3OD: δ_H 3.31, δ_C 49.00) and deuterium oxide (D_2O: δ_H 4.78). Coupling constants are given in hertz (Hz). All individual assignments were made using 2D NMR (^{1}H-^{1}H COSY, ^{1}H-^{13}C HSQC, and ^{1}H-^{13}C HMBC) spectroscopy. The spectroscopic data are presented as follows: Chemical shift, multiplicity (s = singlet, d = doublet, t = triplet, q = quartet, p = pentet (quintet), m = multiplet, br = broad, app. = apparent and combinations thereof), coupling constant, integration and structural assignment.

Mass spectra were recorded on a Waters Xevo G2-XS Tof or Synap G2-S mass spectrometer using Zspray, and a Bruker microTOF® LCMS using Electro-spray ionisation in positive (ESI+) and negative (ESI–) modes.

Infrared spectra were recorded on a Perkin Elmer Spectrum 100 FT-IR and a Varian 660-IR FTIR spectrometer, using Agilent Resolution Pro with absorption maxima 4 (V̄max) reported in cm^{-1}.

Melting points were measured using a Gallenkamp melting point apparatus and are uncorrected.

Optical rotations were measured using a Bellingham and Stanley ADP450 Series-Peltier polarimeter at 25 ˚C using the sodium D line (589.3 nm) with the indicated concentration and solvent.

High performance liquid chromatography (HPLC) analysis was performed using an LC-20 prominence system from Shimazdu, Chromeleon client, version 6.80 SR15, Build 4656, using Phenomenex C18 and Lux® Cellulose-1 (3 µm, 250 x 4.6 mm) columns. A Shimazdu SPD-M20A diode Array Detector was used for the UV detection, monitored at either 210, 220, 254 or 280 nm with the indicated flow rate and solvent system.

General Synthetic Procedures

Procedure A**: The synthesis of hydroxybenzoic acid methyl esters**

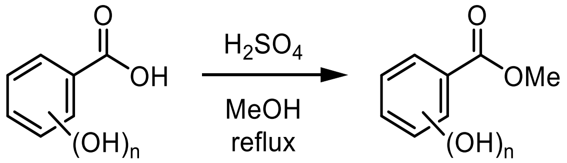

A solution of benzoic acid (10 mmol) in MeOH (20 mL) was charged with conc. H_2SO_4 (0.2 mL). The reaction mixture was heated under reflux until complete consumption of starting material was observed (TLC). The flask was cooled to an ambient temperature and the solvent removed under reduced pressure to afford a crude oil. The oil was neutralised to pH 8 – 9 with satd. $NaHCO_3$ $_{(aq.)}$ (50 mL) and extracted with EtOAc (3 × 30 mL). The combined organic extracts were washed with brine (30 mL) and dried ($MgSO_4$). Filtration of the solids and removal of solvent under reduced pressure afforded the desired benzoic acid methyl ester.

Procedure B**: The synthesis of allyloxy benzoic acid methyl esters**

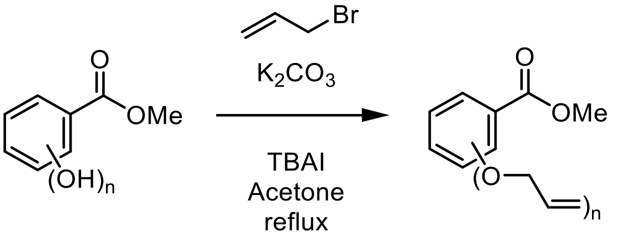

A mixture of hydroxybenzoic acid methyl ester (5.0 mmol), K_2CO_3 (1.2 eq. per OH) and tetrabutylammonium iodide (TBAI) (0.5 eq.) in acetone (25 mL) was charged dropwise with allyl bromide (1.2 eq.) at room temperature. The reaction mixture was heated under reflux until complete consumption of starting material was observed (TLC). The flask was cooled to an ambient temperature and the solvent removed under reduced pressure to afford a crude oil. The oil was charged with satd. NH_4Cl $_{(aq.)}$ (50 mL) and the product extracted with EtOAc (3 × 30 mL). The combined organic extracts were washed with 1.0 M HCl $_{(aq.)}$ (2 × 30 mL), H_2O (30 mL), brine (30 mL) and dried ($MgSO_4$). Filtration of the solids and removal of solvent under reduced pressure afforded a crude mixture. Purification by chromatography (SiO_2, EtOAc/hexane, 1:9) afforded the desired allyloxy benzoic acid methyl ester.

Procedure C**: The Upjohn dihydroxylation of aryl-allyl ethers**

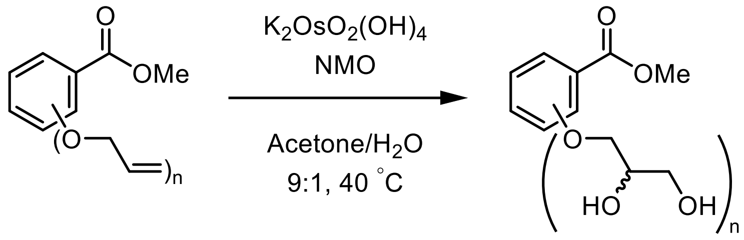

A 25 mL round bottom flask containing *N*-methylmorpholine-*N*-oxide (NMO) (1.2 eq.) was charged with acetone/H_2O (9:1, 10 mL) and stirred vigorously. Potassium osmate (0.01 eq.) was added directly and the reaction mixture was stirred at room temperature for 10 min. The flask was charged with alkene (1.0 eq.) dropwise and heated at 40 ˚C until complete consumption of starting material was observed (TLC, EtOAc/hexane 1:1, or EtOH/EtOAc 1:4). The reaction was quenched with Na_2SO_3 (3.0 eq) and stirred at room temperature for 1 h. The majority of solvent was removed under reduced pressure and the flask was charged with H_2O (10 mL). The aqueous mixture was extracted with CH_2Cl_2 (3 × 30 mL) and the combined organic extracts were washed with 1.0 M HCl $_{(aq.)}$ (2 × 30 mL), brine (30 mL) and dried ($MgSO_4$). Filtration of the solids and removal of solvent under reduced pressure afforded the desired 1,2-propanediol. Alternatively, for highly polar compounds, no work up procedure was required and the crude reaction mixture was purified directly by chromatography (SiO_2, EtOAc/EtOH 4:1).

Procedure D**: The Sharpless AD of aryl-allyl ethers**

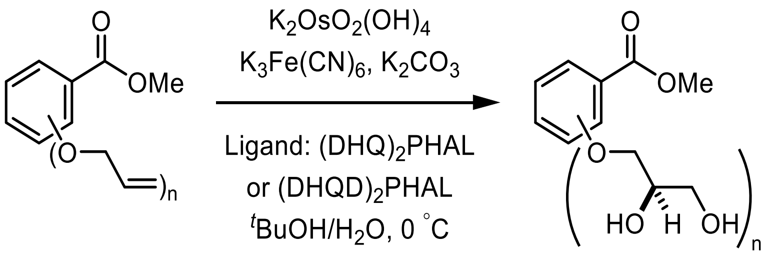

A 25 mL round bottom flask was charged with stock solution **I** (3.0 eq.), **II** (3.0 eq.), **III** (0.002 eq.) and either solution **IV** (ADmix α/DHQ_2(PHAL)) or **V** (ADmix β/$DHQD_2$(PHAL)) (0.02 eq.) forming a biphasic homogeneous mixture. The flask was cooled to 0 ˚C (ice bath) and stirred vigorously for 20 min creating a heterogeneous orange slurry to which the alkene (1.0 eq.) was added directly. The reaction mixture was stirred at 0 ˚C until complete consumption of starting material was observed (TLC, EtOAc/hexane 1:1, or EtOH/EtOAc 1:4). The reaction

was quenched with neat Na_2SO_3 (12.0 eq.) and warmed to room temperature over 1 h. The flask was charged with H_2O (5 mL) and extracted with CH_2Cl_2 (3 × 20 mL). The combined organic extracts were washed with 1.0 M HCl $_{(aq.)}$ (2 × 30 mL), brine (30 mL) and dried ($MgSO_4$). Filtration of the solids and removal of solvent under reduced pressure afforded the desired 1,2-propanediol. Alternatively, for highly polar compounds, no work up procedure was required and the crude reaction mixture was purified directly by chromatography (SiO_2, EtOAc/EtOH 4:1).

Preparation of Stock solutions I-V

Stock solution I*: A 25 mL volumetric flask was charged with $K_3Fe(CN)_6$ (12.347 g, 37.5 mmol) and diluted with distilled water (25.0 mL). The flask was placed in an ultrasonic bath at 50 ˚C to give a 1.5 M solution.

Stock solution II: A 25 mL volumetric flask was charged with K_2CO_3 (5.182 g, 37.5 mmol) and diluted with distilled water (25.0 mL) to give a 1.5 M solution.

Stock solution III: A 10 mL volumetric flask was charged with $K_2OsO_2(OH)_4$ (7.3 mg, 0.02mmol) and diluted with distilled water (10.0 mL) to give a 0.002 M solution.

Stock solution IV*: A 25 mL volumetric flask was charged with $(DHQ)_2PHAL$ (194.7 mg, 0.25 mmol) and diluted with warmed ^{t}BuOH (25.0 mL) to give a 0.01 M solution.

Stock solution V*: A 25 mL volumetric flask was charged with $(DHQD)_2PHAL$ (194.7 mg, 0.25 mmol) and diluted with warmed ^{t}BuOH (25.0 mL) to give a 0.01 M solution.

Note: Stock solutions **II**, **IV** and **V** are stable at room temperature for greater than 2 months. Solutions **I** and **III** were found to be stable up to 3 weeks stored in a fridge at 4 – 8 ˚C under an inert atmosphere of argon.

*Stock solution **I** was placed in an ultrasonic bath at 50 ˚C for 20 min and cooled to room temperature prior to each use. Stock solution **IV** and **V** were warmed to 35 ˚C prior to each use.

Procedure E**: The preparation of persulfates using TBSAB**[S1]

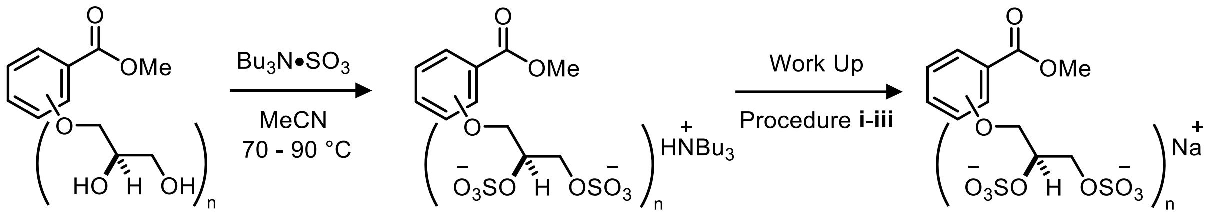

A flame dried 25 mL Schlenk flask was charged with $R\text{-}(OH)_n$ (1.0 mmol) and $Bu_3N•SO_3$ (TBSAB) (2.0 eq. per OH group) and the internal environment was made inert by a pump-purge cycle with dry argon. Anhydrous MeCN was added (giving a concentration of 0.50 – 0.25 Mol dm^{-3} to the limiting reagent) and the reaction mixture heated at 90 ˚C, with monitoring by TLC. The flask was cooled to room temperature and the solvent removed under reduced pressure. The flask was charged with H_2O (20 mL) and the aqueous mixture extracted with EtOAc or CH_2Cl_2 (4 × 30 mL). The organic extracts were pooled, dried ($MgSO_4$), filtered and the solvent was removed under reduced pressure to afford the desired sulfate ester as its $[Bu_3NH]^+$ salt. Alternatively, the crude reaction mixture was used directly in steps **i-iii** without extraction into organic solvent.

Work-Up procedure i: The flask containing the $[Bu_3NH]^+$ salt was charged with EtOH (30 mL) and NEH (5.0 eq. per sulfate group). The reaction mixture was stirred vigorously for 1 h at room temperature. The solid was collected by filtration, washed with EtOH (3 × 20 mL) and dried to a constant weight to afford the desired sulfate as its Na^+ salt.

Work-Up procedure ii: The flask containing the $[Bu_3NH]^+$ salt was charged with Et_2O (30 mL), EtOH (5 mL) and NEH (5.0 eq. per sulfate group). The reaction mixture was stirred vigorously for 1h at room temperature. The solid was removed by filtration, washed with a small amount of cold EtOH and dried to a constant weight to afford the desired sulfate as its Na^+ salt.

Work-Up procedure iii: The flask containing the $[Bu_3NH]^+$ salt was charged with MeCN (20 mL) and NaI (5.0 eq. per sulfate group). The reaction mixture was stirred vigorously for 1h at room temperature. The solid was removed by filtration, washed with MeCN (3 × 30 mL) and dried to a constant weight to afford the desired sulfate as its Na^+ salt.

Biological assay and AMDET experimental details

Cell Viability Assessment (MTT)

HepG2 human hepatocellular carcinoma cells (Cyprotex, Macclesfield, UK) were plated on 96-well tissue culture polystyrene plates for 24 h prior to dosing of the cells. Test compound is diluted in DMSO and serial dilutions are made in 0.5% DMSO in growth media. Test compound at 8 concentrations in triplicate is then incubated for 72 h, and appropriate controls are simultaneously used as quality controls. One hour prior to the end of the incubation period, the cells are loaded with MTT [yellow; 3-(4,5-Dimethyl-2-thiazolyl)-2,5-diphenyl-2H-tetrazolium bromide], the plates are dried and re-solubilised using DMSO. The plates are then scanned at 570 nm. The vehicle controls are used to determine the definitions of "normal" for each parameter, then scanned using a microplate absorbance reader to calculate the percentage of cells that are low or high responders (depending on the biological significance of a particular readout). The vehicle control wells are then used to determine significance limits for wells that have a greater than expected fraction of low or high responders. The minimum effective concentration is determined from the lowest concentration whose mean value exceeds the significance level, provided either a clear dose-response relationship is observed, or at least two consecutive concentration points are above the significance level. AC_{50} values are also determined provided a clear dose-response relationship is observed.

Preparation of FFA-albumin complexes and cell treatments

To generate the in vitro model of oxidative stress, sodium palmitate was bound to bovine serum albumin (BSA) as outlined by Chavez and Summers,[S2] with modifications. Sodium palmitate (FFA) was dissolved in EtOH at 60 ˚C and diluted 1:100 in M199 media supplemented with 2% fatty acid-free bovine serum albumin (BSA) and incubated for 1 h at 37 ˚C. HepG2 cells were treated with 1 µmol/L of the synthesized HS-glycomimetics in the presence of palmitate (100 µmol/L) for 3 h or pre-treated with glycomimetics for 12 h followed by 3 h palmitate (100 µmol/L). Control cells were incubated in serum free M199 containing 2% (wt/vol) fatty acid-free BSA.

NO release assay and Quantification of ROS production

NO release was determined using diaminofluorescein-2 (DAF-2).[S3] The cells were washed with PBS and incubated with *L*-arginine (100 µmol/L in PBS) for 5 min at 37 ˚C before incubation with DAF-2 (0.1 µmol/L) for 2 min, followed by the calcium ionophore calimycin (A23187, 1 µmol L) for 30 min. L-NAME (100 µmol/L), a nitric oxide inhibitor (NOS), was also added 20 min before the addition of *L*-arginine in some experiments to determine the involvement of NOS. DAF-2 fluorescence (arbitrary units, AU) was quantified using a microplate reader (BioTek) with excitation and emission wavelengths of 485 nm and 540 nm, respectively. The auto-fluorescence was subtracted from each value. Cells were incubated with 10 µmol/L of the fluorescent probe: 2′-7′-dichlorodihydrofluorescein diacetate (CM-H2DCFDA, Sigma) for 30 min at 37 ˚C, and the level of fluorescence was determined (excitation 490 nm; emission 540 nm) using a microplate reader (BioTek).

NADPH oxidase activity

The level of NADPH-enhanced superoxide anions ($O_2^{\bullet -}$) in HepG2 homogenates was determined by lucigenin-enhanced chemiluminescence.[S4] Following the various treatment conditions, cells were homogenized in a buffer (20 mmol/L H_2KPO_4 and 1 mmol/L EDTA supplemented with aprotinin, leuprotinin, pepstatin and PMSF). The total protein quantified using the Bicinchoninic acid (BCA) assay (Pierce Biotechnology). To 50 µg of total protein, NADPH (100 µmol/L) was added in a total volume of 500 µL, with lucigenin (5 µmol/L) and was injected automatically. Activity was determined by measuring luminescence over 200 s in a scintillation counter (Lumat LB 9507, Berthold, Germany). Basal values of NADPH oxidase activity were subtracted from the experimental values and expressed as RLU/min/µg protein for cells.

In situ quantification of ROS species

Superoxide dismutase (SOD) and catalase (CAT) activity assays, and *in situ* detection of vascular ROS content. The activity of the enzymes, SOD and CAT, was determined in cell homogenates using assay kits according to the manufacturer's instructions (Cayman; Cat No 706002 and 707002 respectively). A unit of SOD was defined as the amount of enzyme required to demonstrate 50% dismutation of superoxide radical. Similarly, a unit of CAT was defined as the amount required to form 1.0 nmol of formaldehyde per min at 25 ˚C.[S5]

HepG2 cells were incubated in serum-free DMEM containing 2% fatty acid-free BSA (control) or palmitate conjugated BSA (100 μmol/L) in the presence of HS-glycomimetics (1 or 10 μmol/L) for 24 h. The cells were incubated for 30 min in Hepes-buffered solution containing 10 μmol/L dihydroethidium (DHE), which intercalates with DNA in the presence of ROS and is detectable by fluorescence. Cells were counterstained with the nuclear stain DAPI at 0.3 μmol/L. In the following 24 h, the cell cultures were examined using a fluorescence microscope (Leica DMIRB). Sections were photographed and fluorescence was quantified using ImageJ. All parameters (pinhole, contrast, gain and offset) were held constant for all sections from the same experiment. ROS production was estimated from the ratio of ethidium/DAPI fluorescence.[S6]

Western blot analysis

HepG2 wells were lysed in RIPA buffer containing proteinase inhibitors and the total protein was quantified using BCA protein assay kit (Pierce Biotechnology). Proteins (30 μg/well) were denatured and separated by sodium dodecyl sulfate (SDS)-polyacrylamide gel electrophoresis and transferred to polyvinylidene difluoride (PVDF) membranes. Blots were blocked for 1 h in 5% semi-skimmed milk/Tris-Buffered Saline Tween-20 (TBS-T), probed with primary rabbit anti-phospho-protein kinase B (Akt), rabbit anti-Akt (Santa Cruz Biotechnology), rabbit polyclonal anti-phospho-eNOS (Cell Signaling) overnight at 4 ˚C followed by washing and incubation with the corresponding horse radish peroxidase-labelled secondary antibody for 1 h at room temperature. After washing with TBS-T, membranes were incubated with ECL reagent and visualized (Amersham Pharmacia Biotech). Densitometry analysis using ImageJ© (version 1.32j, NIH) was used to quantify the protein signal.[S7]

Turbidimetric Aqueous Solubility

Test compound (10 mM in DMSO) was serially diluted to give solutions of 0.1, 0.3, 1 and 3 mM in DMSO. Each test compound concentration was then further diluted 1 in 100 in buffer (typically 0.01 M phosphate buffered saline pH 7.4) so that the final DMSO concentration is 1 % and the final test compound concentrations are 1, 3, 10, 30 and 100 µM. The experiment is performed at 37 °C and each concentration is incubated in 7 replicate wells. The plates are incubated for 2 h at 37 °C before the absorbance is measured at 620nm. Nicardipine (pH dependent) and pyrene (pH independent) are included as control compounds. The solubility is estimated from the concentration of test compound that produces an increase in absorbance above vehicle control (1% DMSO in buffer).

Microsomal Metabolic Stability

Pooled liver microsomes (final protein concentration 0.5 mg/mL), 0.1 M phosphate buffer pH 7.4 and test compound (final substrate concentration 3 µM; final DMSO concentration 0.25 %) are pre-incubated at 37 °C prior to the addition of NADPH (final concentration 1 mM) to initiate the reaction. The final incubation volume is 50 µL. A minus cofactor control incubation is included for each compound tested where 0.1 M phosphate buffer pH 7.4 is added instead of NADPH (minus NADPH). Two control compounds are included with each species. All incubations are performed singularly for each test compound. Each compound is incubated for 0, 5, 15, 30 and 45 min. The control (minus NADPH) is incubated for 45 min. The reactions are stopped by transferring 20 µL of incubate to 60 µL methanol at the appropriate time points. The termination plates are centrifuged at 2,500 rpm for 20 min at 4 °C to precipitate the protein. Following protein precipitation, the sample supernatants are combined in cassettes of up to 4 compounds, internal standard is added and samples analysed using Cyprotex generic LC-MS/MS conditions. From a plot of ln peak area ratio (compound peak area/internal standard peak area) against time, the gradient of the line is determined. Subsequently, half-life and intrinsic clearance are calculated using the equations below:

Elimination rate constant (k) = (- gradient)

Half-life ($t_{½}$)(min) = $\frac{0.693}{k}$

Intrinsic clearance (CLint)(µL/min/mg protein) = $\frac{V \times 0.693}{t_{½}}$

where V = Incubation volume (µL)/Microsomal protein (mg)

Relevant control compounds are assessed, ensuring intrinsic clearance values fall within the specified limits (if available).

***h*ERG assay**

The experiments are performed on an IonWorks^{TM} automated patch clamp instrument (Molecular Devices LLC), which simultaneously performs electrophysiology measurements for 48 single cells in a specialised 384-well plate (PatchPlate^{TM}). All cell suspensions, buffers and test compound solutions are at room temperature during the experiment. The cells used are Chinese hamster ovary (CHO) cells stably transfected with *h*ERG (cell-line obtained from Cytomyx, UK). A single-cell suspension is prepared in extracellular solution (Dulbecco's phosphate buffered saline with calcium and magnesium pH 7.2) and aliquots added

automatically to each well of a PatchPlate™. The cells are then positioned over a small hole at the bottom of each well by applying a vacuum beneath the plate to form an electrical seal. The vacuum is applied through a single compartment common to all wells which is filled with intracellular solution (buffered to pH 7.2 with HEPES). The resistance of each seal is measured *via* a common ground-electrode in the intracellular compartment and individual electrodes placed into each of the upper wells.

Electrical access to the cell is then achieved by circulating a perforating agent, amphotericin B, underneath the PatchPlate™. The pre-compound *h*ERG current is then measured. An electrode is positioned in the extracellular compartment and a holding potential of -80 mV applied for 15 s. The *h*ERG channels are then activated by applying a depolarising step to +40 mV for 5 s and then clamped at -50 mV for 4 s to elicit the *h*ERG tail current, before returning to -80 mV for 0.3 s. Compound dilutions are prepared by diluting a DMSO solution (10 mM) of the test compound using a factor 5 dilution scheme into DMSO, followed by dilution into extracellular buffer such that the final concentrations tested are typically 0.008, 0.04, 0.2, 1, 5, 25 µM (final DMSO concentration 0.25 %). The IonWorks™ instrument automatically adds test compound dilutions to the upper wells of the PatchPlate™. The test compound is left in contact with the cells for 300 s before recording currents using the same voltage-step protocol as in the pre-compound scan. Quinidine, an established *h*ERG inhibitor, is included as a positive control, and vehicle control (0.25 % DMSO) as negative control. Each concentration is tested in 4 replicate wells on the PatchPlate™ (maximum of 24 data points). Filters are applied to ensure only acceptable cells are used to assess *h*ERG inhibition. The cell must maintain a seal resistance of greater than 50 MΩ and a pre-compound current of at least 0.1 nA, and ensure cell stability between pre-and post-compound measurements.

For each replicate the *h*ERG response is calculated using the following equation:

$$\%\ \mathrm{hERG\ response} = \frac{\mathrm{Post-compound\ current\ (nA)}}{\mathrm{Pre-compound\ current\ (nA)}}\ \mathrm{x}\ 100$$

The % *h*ERG response is plotted against concentration for the test compound and, where concentration-dependent inhibition is observed, the data are fitted to the following equation and an IC_{50} value calculated:

$$y = \frac{y_{max} - y_{min}}{1 + \left(\frac{IC_{50}}{x}\right)^{s}} + y_{min}$$

Where:

y = *h*ERG response

y_{max} = mean vehicle control response

x = concentration

s = Hill slope

Caco-2 Permeability (A-B or Bi-directional)

Caco-2 cells (ATCC, Virginia, USA) are used between passage numbers 40-60. Cells are seeded onto Millipore Multiscreen Transwell plates at 1 x 105 cells/cm^2. The cells are cultured in DMEM and media is changed every two or three days. On day 20 the permeability study is performed. Cell culture and assay incubations are carried out at 37 °C in an atmosphere of 5 % CO_2 with a relative humidity of 95%. On the day of the assay, the monolayers are prepared by rinsing both apical and basolateral surfaces twice with Hanks Balanced Salt Solution (HBSS) at the desired pH warmed to 37 °C. Cells are then incubated with HBSS at the desired pH in both apical and basolateral compartments for 40 min to stabilise physiological parameters.

The dosing solutions are prepared by diluting test compound with assay buffer to give a final test compound concentration of 10 µM (final DMSO concentration of 1 % v/v). The fluorescent integrity marker lucifer yellow is also included in the dosing solution. Analytical standards are prepared from test compound DMSO dilutions and transferred to buffer, maintaining a 1% v/v DMSO concentration. Typical assay buffer is composed of supplemented HBSS pH 7.4 but a range of other buffers and pH values can be used.

For assessment of A-B permeability, HBSS is removed from the apical compartment and replaced with test compound dosing solution. The apical compartment insert is then placed into a companion plate containing fresh buffer (containing 1 % v/v DMSO). For assessment of B-A permeability, HBSS is removed from the companion plate and replaced with test compound dosing solution. Fresh buffer (containing 1 % v/v DMSO) is added to the apical compartment insert, which is then placed into the companion plate.

At 120 min the apical compartment inserts and the companion plates are separated and apical and basolateral samples diluted for analysis. Test compound permeability is assessed in duplicate. Compounds of known permeability characteristics are run as controls on each assay plate. Test and control compounds are quantified by LC-MS/MS cassette analysis using an 8-point calibration with appropriate dilution of the samples. The starting concentration (C_0) is determined from the dosing solution and the experimental recovery calculated from C_0 and both apical and basolateral compartment concentrations.

The integrity of the monolayer throughout the experiment is checked by monitoring lucifer yellow permeation using fluorimetric analysis. Lucifer yellow permeation is high if monolayers have been damaged. If a lucifer yellow P_{app} value is above a pre-defined threshold in one individual test compound well, the compound may be re-tested or an $n = 1$ result is reported. If lucifer yellow P_{app} values are above the threshold in both replicate wells for a test compound, the compound is re-tested. If this re-occurs upon repeat in both wells then toxicity or inherent fluorescence of the test compound is assumed.

The permeability coefficient (P_{app}) for each compound is calculated from the following equation:

$$P_{app} = \left(\frac{dQ/dt}{C_0 \times A}\right)$$

Where dQ/dt is the rate of permeation of the drug across the cells, C_0 is the donor compartment concentration at time zero and A is the area of the cell monolayer. C_0 is obtained from analysis of the dosing solution.

For bi-directional experiments, an efflux ratio (ER) is calculated from mean A-B and B-A data. This is derived from:

$$ER = \frac{P_{app(B-A)}}{P_{app(A-B)}}$$

Three control compounds are screened alongside the test compounds, atenolol (human absorption 50%), propranolol (human absorption 90%) and talinolol (a substrate for P-glycoprotein).

Molecular Docking

Glycomimetic optimized structures and the partial charges were obtained from *ab initio* calculations performed by ATB server.[S8] The protein structure was allowed to relax during 20 ns of molecular dynamics before proceeding with molecular docking. AutoDockTools software of the MGL program Tools 1.5.4[S9] was used to prepare the protein by adding polar hydrogen atoms and Gasteiger charges. The maps were generated by AutoGrid 4.2 program[S9] with a spacing of 0.592Å, dimension of 40x40x40 points and grid center coordinates as being 57.496, 18.131 and 71.217 for x, y and z coordinates, respectively. The AutoDock 4.2 was used to investigate the protein binding site using the Lamarckian Genetic Algorithm (LGA) with a population size of 150, maximum number of generations of 27000 and energy evaluations equal to $2.5x10^6$. The other parameters were selected as software defaults. To generate different conformations, the total number of runs was set to 100. The final energy score was calculated following the equation:

$$\Delta G = \left(V_{bound}^{L-L} - V_{umbound}^{L-L}\right) + \left(V_{bound}^{P-P} - V_{umbound}^{P-P}\right) + \left(V_{bound}^{P-L} - V_{umbound}^{P-L}\right) + \Delta S_{conformation}$$

Where V is the potential employed to perform the calculation:

$$V\ (energy\ score) = I.\sum_{i,j}\left(\frac{A_{ij}}{r_{ij}^{12}} - \frac{B_{ij}}{r_{ij}^{6}}\right) + J.\sum_{i,j} E(t).\left(\frac{C_{ij}}{r_{ij}^{12}} - \frac{D_{ij}}{r_{ij}^{10}}\right)\ + K.\sum_{i,j}\frac{q_i.q_j}{\varepsilon.r_{ij}^2} + \Delta W$$

The weighting constants I, J, E(t) and K are those optimized to calibrate the empirical free energy based on a set of experimentally characterized complexes. The first term is the Lenard-Jones potential, in which parameters A and B are taken from the Amber force field. The second term refers to the hydrogen bond in which the parameters C and D are obtained to ensure a minimum energy of 5 kcal/mol in 1.9 Å for O-H and N-H and 1 kcal/mol in 2.5 Å for S-H. The function E(t) provides directionality based on the angle t of the geometry of an ideal hydrogen bond. The third term is a shielded Coulomb potential for electrostatic interaction. The last term is the desolvation potential based on the volume of the atoms surrounding a given atom and shelter it of the solvent.[S9] The final conformations were visualized by visual molecular dynamics software (VMD).[S10]

Molecular Dynamics: The initial coordinates of the protein–ligand complexes were extracted from the lowest energy cluster obtained from molecular docking. The structure of the protein NK1 was obtained from PDB (1BHT) and was parameterized with GROMOS96/54a7 force field.[S11] The system was solvated with the Simple Point Charge (SPC) water model in a dodecahedral box and neutralized with Na^+ and Cl^- in a concentration of 150mM. Energy minimization was performed with the steepest descent algorithm with 5000 steps and a tolerance of 10 kJ/mol. The first stage of equilibration was performed along 100 ps in NVT

ensemble, coupled to the modified Barendsen thermostat[S12] at 298K, in this stage random velocities were generated by the Maxwell–Boltzman distribution. The second stage of equilibration was performed in the NPT ensemble, coupled to the modified Barendsen thermostat at 298K and to Parrinello–Rahman barostat at 1 atm.[S13] The molecular dynamics simulations were performed by Gromacs 5.1.4,[S14] with steps of 2 fs using the leap-frog algorithm to integrate the equations of motion. The binding free energy (ΔG) was calculated with MM/PBSA a model,[S15] where ΔG is defined as:

$$\Delta G = < G_{complex} > - < G_{protein} > - < G_{glycomimetics} >$$

Where G is expressed as:

$$G = E_{int} + E_{ele} + E_{vdw} + E_{solv} - TS$$

E_{int} is the molecular internal gas-phase energy (from bonds, angles,and dihedral angles), E_{ele} is the electrostatic interaction and E_{vdw} is the van der Waals interaction. E_{solv} is the solvation energy and is calculated with a continuum representation of the solvent for the polar part, and by a relation to the solvent-accessible surface area for the non-polar part. The last term TS is the product of the absolute temperature and the entropy, which is calculated from a normal-mode analysis of a truncated system at the molecular-mechanics level. Molecular dynamics simulations were performed in BlueBear, University of Birmingham and GRID, UNESP.

Compound Characterisation

(**1**) Methyl 2,5-bis(allyloxy)benzoate

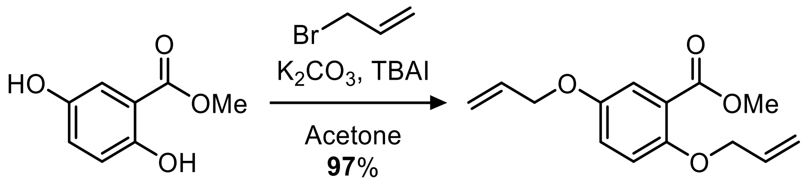

Following general procedure **B**: methyl 2,5-dihydroxybenzoate (**20**) (835 mg, 5.0 mmol), K_2CO_3 (1.658 g, 12.0 mmol) and TBAI (1.612 g, 5.0 mmol) in acetone (25 mL) was charged with allyl bromide (1.04 mL, 12.0 mmol) and heated under reflux for 6 h. Purification by chromatography (SiO_2, EtOAc/hexane 1:9, R_f = 0.41) yielded the title compound as a clear oil (1.201 g, 97%). **IR** ν_{max} cm^{-1} 3082 w, 2949 w, 2865 w, 1730 m (C=O), 1495 m, 1437 w; **^{1}H-NMR** (400 MHz, $CDCl_3$) δ_H 7.35 (d, J = 3.2 Hz, 1H, C6-H), 7.00 (dd, J = 9.0, 3.2 Hz, 1H, C4-H), 6.89 (d, J = 9.0 Hz, 1H, C3-H), 6.09 – 5.96 (m, 2H, CH=CH_2), 5.46 (dq, J = 17.3, 1.6 Hz, 1H, C5-$OCH_2CH=CH_2$), 5.39 (dq, J = 17.3, 1.6 Hz, 1H, C2-$OCH_2CH=CH_2$), 5.26 (dt, J = 10.6, 1.6 Hz, 2H, CH=CH_2), 4.54 (dt, J = 4.9, 1.6 Hz, 2H, C5-OCH_2), 4.49 (dt, J = 5.3, 1.6 Hz, 2H, C2-OCH_2), 3.88 (s, 3H, Me); **^{13}C-NMR** (101 MHz, $CDCl_3$) δ_C 166.6 (CO_2Me), 152.6 (C5), 152.3 (C2), 133.2 (2C, CH=CH_2), 121.3 (C1), 120.4 (C4), 117.8 (CH=CH_2), 117.30 (CH=CH_2), 117.07 (C3), 116.0 (C6), 70.7 (C5-OCH_2), 69.5 (C2-OCH_2), 52.1 (Me); **LRMS** *m/z* (ESI+) 248.09 (50%, $[M]^+$), 207.06 (60%, $[M–C_3H_6]^+$), 175.03 (100%, $[M–(C_3H_6)–CH_3OH)]^+$); **HRMS** *m/z* (ESI+) $C_{14}H_{16}O_4$ Requires: 248.1049, Found: 248.1051 ($[M]^+$).

(**α-2**) Methyl 5-((*R*)-2,3-dihydroxypropoxy)-2-((*S*)-2,3-dihydroxypropoxy)benzoate

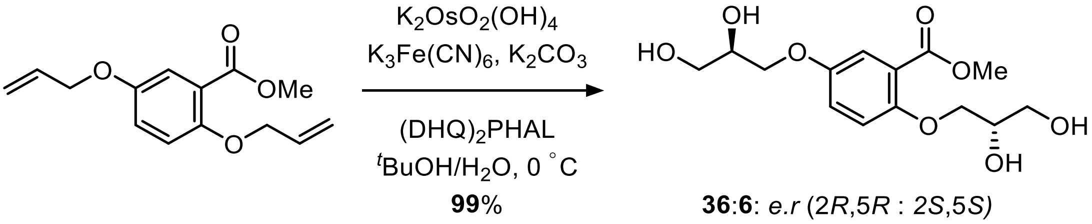

36:**6**: *e.r* (2*R*,5*R* : 2*S*,5*S*)
4:**54** *e.r* (2*R*,5*S* : 2*S*,5*R*)

Adapted from general procedure **D** using stock solutions: **I** (2 mL, 3.0 mmol), **II** (2 mL, 3.0 mmol), **III** (1.0 mL, 2.0 µmol) and **IV** (5.0 mL, 20.0 µmol). Methyl 2,5-bis(allyloxy)benzoate (**1**) (158 mg, 0.50 mmol) was added and the reaction mixture was stirred at 0 ˚C for 6 h with monitoring (EtOH/EtOAc 1:4, R_f = 0.20). The solvent was removed under reduced pressure and the crude mixture was directly purified by chromatography (SiO_2, EtOAc/hexane 1:4) to

afford the title compound as a clear oil (156 mg, 99%). **[α]$_D^{25}$** –13.15 (c. 1.0, MeOH, 36:6:4:54 *e.r*/*d.r* (*2R,5R; 2S,5S; 2R,5S; 2S,5R*)); **IR** V̄max cm^{-1} 3268 w, 2939 w, 2890 w, 1699 s (C=O), 1601 w, 1499 s, 1435 w; **^{1}H-NMR** (400 MHz, $(CD_3)_2SO$) δ_H 7.18 (d, *J* = 2.7 Hz, 1H, C6-H), 7.15 – 7.04 (m, 2H, C3-H & C4-H), 4.94 (d, *J* = 5.0 Hz, 1H, CH-OH), 4.84 (d, *J* = 5.0 Hz, 1H, CH-OH), 4.66 (t, *J* = 5.7 Hz, 1H, CH_2-OH), 4.59 (t, *J* = 5.7 Hz, 1H, CH_2-OH), 4.01 – 3.85 (m, 3H), 3.86 – 3.69 (m, 6H, Me), 3.55 – 3.35 (m, 4H); **^{13}C-NMR** (101 MHz, $(CD_3)_2SO$) δ_C 166.0 (CO_2Me), 152.2 (C5), 152.0 (C2), 121.0 (C1), 119.8 (C3/C4), 115.94 (C3/C4), 115.89 (C6), 71.3 (ArO-CH_2), 70.3 (ArO-CH_2), 69.9 (2C, CH-OH), 62.72 (CH_2-OH), 62.66 (CH_2-OH), 52.0 (Me); **LRMS** *m/z* (ESI+) 339.12 (100%, [M+Na]$^+$); **HRMS** *m/z* (ESI+) $C_{14}H_{20}O_8Na$ Requires: 339.1056, Found: 339.1057 ([M+Na]$^+$).

(**β-2**) Methyl 5-((*S*)-2,3-dihydroxypropoxy)-2-((±)-2,3-dihydroxypropoxy)benzoate

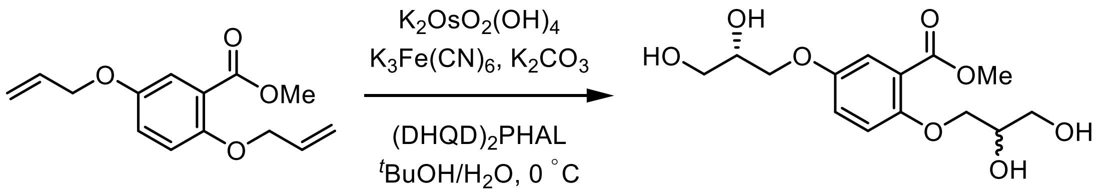

1:**51** *e.r* (2*R5R* : *2S,5S*)
46:**2** *e.r* (2*R,5S* : *2S,5R*)

Adapted from general procedure **D** using stock solutions: **I** (2 mL, 3.0 mmol), **II** (2 mL, 3.0 mmol), **III** (1.0 mL, 2.0 µmol) and **V** (5.0 mL, 20.0 µmol). Methyl 2,5-bis(allyloxy)benzoate (**1**) (158 mg, 0.50 mmol) was added and the reaction mixture was stirred at 0 °C for 6 h with monitoring (EtOH/EtOAc 1:4, R_f = 0.20). The solvent was removed under reduced pressure and the crude mixture was directly purified by chromatography (SiO_2, EtOAc/hexane 1:4) to afford the title compound as a clear oil (156 mg, 99%). **[α]$_D^{25}$** –7.62 (c. 1.0, MeOH, 1:51:46:2 *e.r*/*d.r* (*2R5R, 2S5S, 2R5S, 2S5R*)); **IR** V̄max cm^{-1} 3260 br s (O-H), 2951 w, 2874 w, 1725 (C=O), 1611 w, 1577 w, 1498 s, 1432 w; **^{1}H-NMR** (400 MHz, $(CD_3)_2SO$) δ_H 7.18 (d, *J* = 2.7 Hz, 1H, C6-H), 7.13 – 7.07 (m, 2H, C3-H & C4-H), 4.94 (d, *J* = 5.0 Hz, 1H, CH-OH), 4.84 (d, *J* = 5.0 Hz, 1H, CH-OH), 4.66 (t, *J* = 5.7 Hz, 1H, CH_2-OH), 4.59 (t, *J* = 5.7 Hz, 1H, CH_2-OH), 4.01 – 3.85 (m, 3H), 3.86 – 3.69 (m, 6H, Me), 3.55 – 3.35 (m, 4H); **^{13}C-NMR** (101 MHz, $(CD_3)_2SO$) δ_C 166.1 (CO_2Me), 152.2 (C5), 152.0 (C2), 121.0 (C1), 119.8 (C3/C4), 115.95 (C3/C4), 115.91 (C6), 71.3 (ArO-CH_2), 70.4 (ArO-CH_2), 69.9 (2C, CH-OH), 62.73 (CH_2-OH), 62.67 (CH_2-OH), 52.0 (Me); **LRMS** *m/z* (ESI+) 339.12 (100%, [M+Na]$^+$); **HRMS** *m/z* (ESI+) $C_{14}H_{20}O_8Na$ Requires: 339.1056, Found: 339.1057 ([M+Na]$^+$).

(α-**3**) Sodium 5-((*R*)-2,3-dihydroxypropoxy)-2-((±)-2,3-dihydroxypropoxy)benzoate.

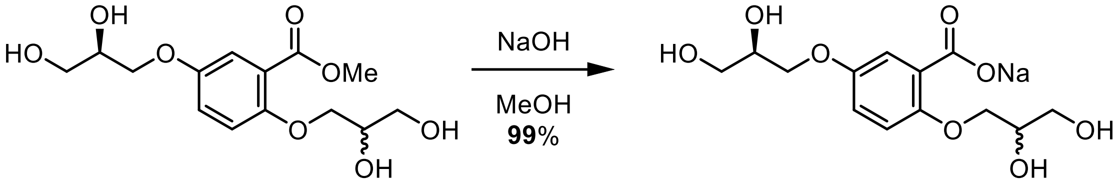

36:**6**: *e.r* (2*R*5*R*, 2*S*5*S*)
4:**54** *e.r* (2*R*5*S*, 2*S*5*R*)

36:**6**: *e.r* (2*R*5*R*, 2*S*5*S*)
4:**54** *e.r* (2*R*5*S*, 2*S*5*R*)

A solution of methyl 5-((*R*)-2,3-dihydroxypropoxy)-2-((±)-2,3-dihydroxypropoxy)benzoate (**α-2**) (100 mg, 0.32 mmol) in MeOH (10 mL) was charged with NaOH (13 mg, 0.32 mmol). The reaction mixture was heated under reflux for 2 h, then cooled to room temperature. The solvent was removed under reduced pressure to afford the title compound as a hygroscopic white solid (103 mg, 99%). **$[\alpha]_D^{25}$** –10.48 (c. 1.0, MeOH, 36:6:4:54 *e.r*/*d.r* (*RR,SS,RS,SR*)); **IR** $\bar{\nu}$max cm^{-1} 3246 br s (O-H), 2934 w, 2877 w, 1559 s (C=O), 1491 s, 1455 w, 1420 s, 1374 s; **^{1}H-NMR** (400 MHz, CD_3OD) δ_H 7.36 (d, *J* = 3.1 Hz, 1H, C6-H), 7.15 (dd, *J* = 9.0, 3.1 Hz, 1H, C4-H), 7.12 – 7.05 (m, 2H, C6-H & C3-H), 6.93 (d, *J* = 9.0 Hz, 1H, C3-H), 6.87 (dd, *J* = 9.0, 3.0 Hz, 1H, C4-H), 4.16 – 3.86 (m, 2 × 6H), 3.79 – 3.57 (m, 2 × 4H); **^{13}C-NMR** (101 MHz, CD_3OD) δ_C 175.9, 154.4, 154.34, 154.25, 151.7, 132.8, 122.0, 121.5, 117.8, 117.2, 116.7, 116.6, 116.2, 73.2, 72.8, 71.9, 71.8 (2C), 71.6, 71.1, 70.9, 64.3, 64.2, 64.1, 63.9; **LRMS** *m/z* (ESI+) 325.09 (30%, $[M+H]^+$); **HRMS** *m/z* (ESI+) $C_{13}H_{18}NaO_8$ Requires: 325.0894, Found: 325.0895 ($[M+H]^+$).

(β-**3**) Sodium 5-((*S*)-2,3-dihydroxypropoxy)-2-((±)-2,3-dihydroxypropoxy)benzoate

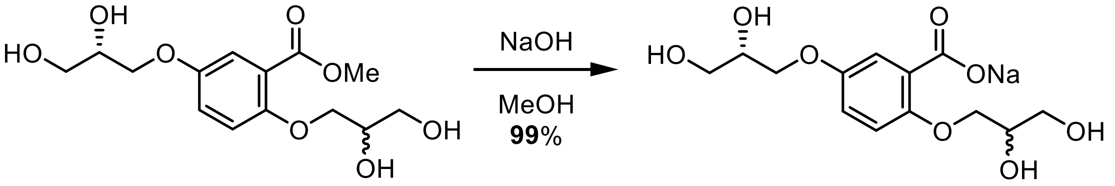

1:**51** *e.r* (2*R*5*R*, 2*S*5*S*)
46:**2** *e.r* (2*R*5*S*, 2*S*5*R*)

1:**51** *e.r* (2*R*5*R*, 2*S*5*S*)
46:**2** *e.r* (2*R*5*S*, 2*S*5*R*)

A solution of methyl 5-((*S*)-2,3-dihydroxypropoxy)-2-((±)-2,3-dihydroxypropoxy)benzoate (**β-2**) (100 mg, 0.32 mmol) in MeOH (10 mL) was charged with NaOH (13 mg, 0.32 mmol). The reaction mixture was heated under reflux for 2 h, then cooled to room temperature. The solvent was removed under reduced pressure to afford the title compound as a hygroscopic white solid (103 mg, 99%). **$[\alpha]_D^{25}$** –8.31 (c. 1.0, MeOH, 1:51:46:2 *e.r*/*d.r* (*RR,SS,RS,SR*)); **IR** $\bar{\nu}$max cm^{-1} 3249 br s (O-H), 2934 w, 2879 w, 1559 s (C=O), 1491 s, 1455 w, 1420 s, 1374 s; **^{1}H-**

NMR (400 MHz, CD_3OD) δ_H 7.36 (d, *J* = 3.1 Hz), 7.15 (dd, *J* = 8.9, 3.1 Hz), 7.12 – 7.07 (m, 1H, C6-H), 6.93 (d, *J* = 8.9 Hz, 1H, C3-H), 6.87 (dd, *J* = 8.9, 3.1 Hz, 1H, C4-H), 4.20 – 3.84 (m, 2 × 6H), 3.78 – 3.55 (m, 2 × 4H); **^{13}C-NMR** (101 MHz, CD_3OD) δ_C 176.4 ($\underline{C}O_2Me$), 154.9, 154.9, 154.8, 152.3, 133.3, 122.0, 118.4, 117.7, 117.3, 117.12 (2C), 116.8, 116.7, 73.7, 73.3, 72.4, 72.3, 72.1, 71.6, 71.4, 64.8, 64.7, 64.6, 64.5; **LRMS** *m/z* (ESI+) 325.09 (30%, $[M+H]^+$); **HRMS** *m/z* (ESI+) $C_{13}H_{18}NaO_8$ Requires: 325.0894, Found: 325.0890 ($[M+H]^+$).

(α-**4**) Sodium 3-(4-((*S*)-2,3-bis(sulfonatooxy)propoxy)-2-(methoxycarbonyl)phenoxy)propane-1,2-diyl bis(sulfate)

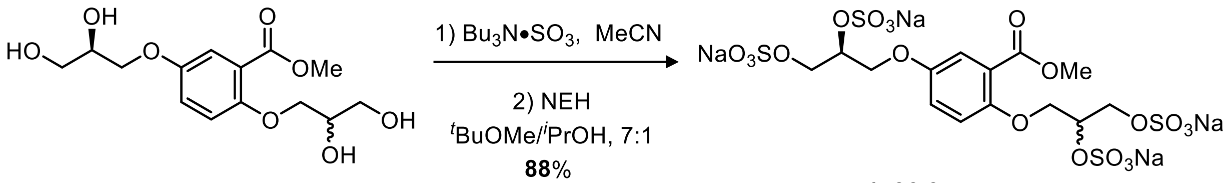

α-**5**, **36**:**6**: *e.r* (*RR,SS*)
4:**54** *e.r* (*RS,SR*)

α-**1**, **36**:**6**: *e.r* (*SS,RR*)
4:**54** *e.r* (*SR,RS*)

Adapted from General procedure **E**: A 25 mL Schlenk tube was charged with **α-5** (81 mg, 0.25 mmol) and $Bu_3N•SO_3$ (530 mg, 2.00 mmol) under Ar and MeCN was added (0.5 mL). The flask was heated at 80 ˚C for 12 h with monitoring (TLC). The flask was cooled to room temperature and the solvent removed under reduced pressure to give a clear viscous oil. The crude oil was dissolved in ^{i}PrOH (5 mL) and transferred to a flask containing ^{t}BuOMe (35 mL). With vigorous stirring a solution of NEH (5.0 mL, 1.0 M) in ^{t}BuOMe/^{i}PrOH (1:7) was added dropwise over 10 min. The precipitate that formed was collected by filtration, washed with ^{i}PrOH (2 × 10 mL) and dried under vacuum. Recrystallization from H_2O/^{i}PrOH afforded the title compound as a white solid (160 mg, 88%). **$[\alpha]_D^{25}$** –3.35 (c. 1.0, H_2O, 36:6:4:54 *e.r*/*d.r* (*SS,RR,SR,RS*)); **M.P** 198 – 200 ˚C (dec.); **IR** ν_{max} cm^{-1} 2988 w, 2164 w, 1711 w (C=O), 1500 w, 1443 w, 1221 s (S=O), 1131 s (S=O); **^{1}H-NMR** (400 MHz, D_2O) δ_H 7.45 (d, *J* = 3.1 Hz, 1H, C6-H), 7.27 (dd, *J* = 9.1, 3.1 Hz, 1H, C4-H), 7.19 (d, *J* = 9.1 Hz, 1H, C3-H), 4.87 (td, *J* = 4.8, 1.8 Hz, 2H, CH-OSO_3Na), 4.45 – 4.25 (m, 8H, Ar-OC$\underline{H}_2$, C$\underline{H}_2$-OSO_3Na), 3.93 (s, 3H, Me); **^{13}C-NMR** (101 MHz, D_2O) δ 168.5 ($\underline{C}O_2Me$), 152.2 (C2), 152.1 (C5), 121.4 (C4), 120.8 (C1), 117.4 (C6), 116.9 (C3), 75.03 ($\underline{C}H$-OSO_3Na), 74.98 ($\underline{C}H$-OSO_3Na), 68.2 (O-$\underline{C}H_2$), 67.3 (O-$\underline{C}H_2$), 66.44 ($\underline{C}H_2$-OSO_3Na), 66.41 ($\underline{C}H_2$-OSO_3Na), 52.8 (Me); **LRMS** *m/z* (ESI+) 746.86 (20%, $[M+Na]^+$); **HRMS** *m/z* (ESI+) $C_{14}H_{16}Na_5O_{20}S_4$ Requires: 746.8601, Found: 746.8591 ($[M+Na]^+$).

(β-**5**) Sodium 5-((*R*)-2,3-bis(sulfonatooxy)propoxy)-2-(2,3-bis(sulfonatooxy)propoxy)benzoate

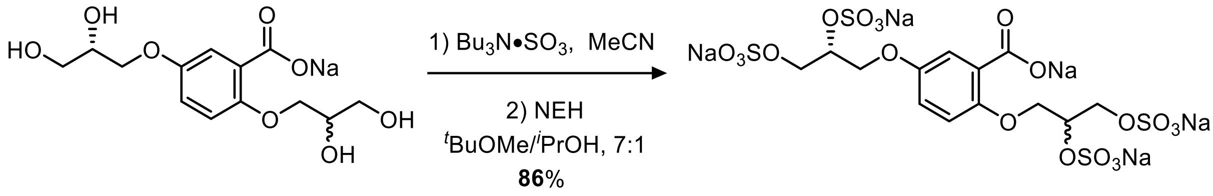

1:**51** *e.r* (*2R5R, 2S5S)*
46:**2** *e.r* (*2R5S, 2S5R)*

1:**51** *e.r* (*2S5S, 2R5R)*
46:**2** *e.r* (*2S5R, 2R5S)*

Adapted from General procedure **E**: A 25 mL Schlenk tube was charged with **β-3** (100 mg, 0.32 mmol) and Bu_3N•SO_3 (678 mg, 2.56 mmol) under Ar $_{(g)}$ and MeCN was added (0.6 mL). The flask was heated at 80 ˚C for 12 h and with monitoring (TLC). The flask was cooled to room temperature and the solvent removed under reduced pressure to give a clear viscous oil. The crude oil was dissolved in ^{i}PrOH (5 mL) and transferred to a flask containing ^{t}BuOMe (35 mL). With vigorous stirring a solution of NEH (5.0 mL, 1.0 M) in ^{t}BuOMe/^{i}PrOH (1:7) was added dropwise over 10 min. The precipitate that formed was collected by filtration, washed with ^{i}PrOH (2 × 10 mL) and dried under vacuum. Recrystallization from H_2O/^{i}PrOH afforded the title compound as a white solid (200 mg, 86%). **$[\alpha]_D^{25}$** –1.66 (c. 1.0, H_2O, 1:51:46:2 *e.r*/*d.r* (*SS,RR,SR,RS*)); **M.P** 200 – 202 ˚C (dec.); **IR** Vmax cm^{-1} 2988 w, 1712 w (C=O), 1499 w, 1435 w, 1100 s (S=O); **^{1}H-NMR** (400 MHz, D_2O) δ 7.47 – 6.94 (m, 3H), 5.00 – 4.73 (m, 2H), 4.46 – 3.99 (m, 8H); **^{13}C-NMR** (101 MHz, D_2O) δ 169.6 (<u>C</u>O_2Na), 152.3 (<u>C</u>-O), 151.8 (<u>C</u>-O), 121.5 (<u>C</u>-CO_2Na), 120.7 (<u>C</u>-H), 117.4 (<u>C</u>-H), 116.2 (<u>C</u>-H), 75.0 (<u>C</u>-H), 74.9 (<u>C</u>-H), 68.3 (<u>C</u>-H_2), 67.3 (<u>C</u>-H_2), 66.6 (<u>C</u>-H_2), 66.4 (<u>C</u>-H_2); **LRMS** *m/z* (ESI+) 732.84 (10%, $[M+H]^+$); **HRMS** *m/z* (ESI+) $C_{13}H_{14}Na_5O_{20}S_4$ Requires: 732.8444, Found: 732.8439 ($[M+H]^+$).

(**6**) Methyl 5-(allyloxy)-2-hydroxybenzoate

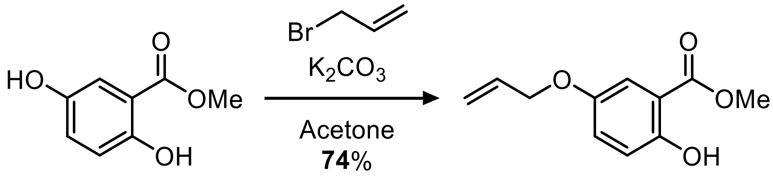

Adapted from general procedure **B**: methyl 2,5-dihydroxybenzoate, **S1** (835 mg, 5.0 mmol) and K_2CO_3 (691 mg, 5.0 mmol) in acetone (50 mL) was charged dropwise with allyl bromide (0.43 mL, 5.0 mmol) and the reaction mixture was stirred at room temperature for 48 h. Purification by chromatography (SiO_2, EtOAc/hexane, 1:9, R_f = 0.62) yielded the title compound as a colourless oil (770 mg, 74%). **IR** Vmax cm^{-1} 3222 w, 2955 w, 1677 S (C=O),

1615 w, 1486 s, 1439 s; **^{1}H-NMR** (400 MHz, $CDCl_3$) δ_H 10.39 (s, 1H, C2-O<u>H</u>), 7.33 (d, *J* = 3.1 Hz, 1H, C6-<u>H</u>), 7.12 (dd, *J* = 9.0, 3.1 Hz, 1H, C4-<u>H</u>), 6.93 (d, *J* = 9.0 Hz, 1H, C3-<u>H</u>), 6.06 (ddt, *J* = 17.2, 10.5, 5.4 Hz, 1H, C<u>H</u>=CH_2), 5.43 (dd, *J* = 17.2, 1.5 Hz, 1H, CH=C<u>H</u>$_2$), 5.31 (dd, *J* = 10.5, 1.5 Hz, 1H, CH=C<u>H</u>$_2$), 4.51 (d, *J* = 5.4 Hz, 2H, C5-OC<u>H</u>$_2$), 3.96 (s, 3H, Me); **^{13}C-NMR** (101 MHz, $CDCl_3$) δ_C 170.4 (<u>C</u>O_2Me), 156.3 (C5), 151.1 (C2), 133.3 (<u>C</u>H=CH_2), 124.9 (C4), 118.6 (C3), 117.9 (CH=<u>C</u>H_2), 113.4 (C6), 112.0 (C1), 69.7 (C5-O<u>C</u>H_2), 52.5 (Me); **LRMS** *m/z* (ESI+) 208.06 (30%, $[M]^+$), 167.02 (60%, $[M–C_3H_5]^+$), 134.99 (100%, $[M–(C_3H_5)–CH_3OH]^+$); **HRMS** *m/z* (ESI+) $C_{11}H_{12}O_4$ Requires: 208.0736, Found: 208.0737 ($[M]^+$).

(**7**) Methyl 2-(4-acetoxybutoxy)-5-(allyloxy)benzoate

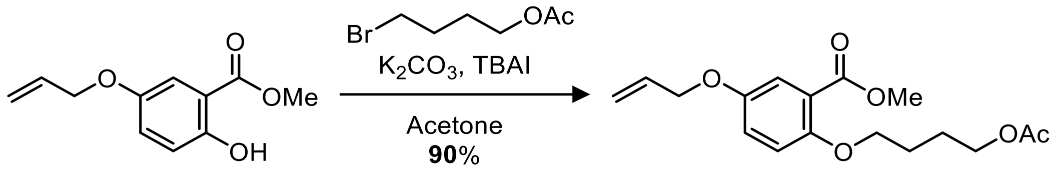

A flask containing methyl 5-(allyloxy)-2-hydroxybenzoate (**6**) (1.00 g, 4.8 mmol), K_2CO_3 (1.29 g, 9.6 mmol) and TBAI (0.77 g, 2.4 mmol) in acetone (20 mL) was charged with 4-bromobutyl acetate (6.1 g, 14.4 mmol) and the reaction mixture was heated under reflux for 12 h. The flask was cooled to room temperature and the solvent removed under reduced pressure. The crude residue was charged with H_2O (20 mL) and extracted with EtOAc (3 × 50 mL). The combined organic extracts were washed with brine, dried ($MgSO_4$), and filtration of the solids and removal of the solvent under reduced pressure afforded a yellow oil. Purification by chromatography (SiO_2, EtOAc/hexane 1:5, R_f = 0.23) afforded the title compound as a clear oil (1.54 g, 90%). **IR** ν_{max} cm^{-1} 2946 m, 1734s (C=O), 1499 m, 1439 w; **^{1}H-NMR** (400 MHz, $CDCl_3$) δ_H 7.33 (d, *J* = 3.2 Hz, 1H, C6-<u>H</u>), 7.01 (dd, *J* = 9.0, 3.2 Hz, 1H, C4-<u>H</u>), 6.88 (d, *J* = 9.0 Hz, 1H, C3-<u>H</u>), 6.02 (ddt, *J* = 17.2, 10.5, 5.3 Hz, 1H, C<u>H</u>=CH_2), 5.39 (dd, *J* = 17.2, 1.5 Hz, 1H, CH=C<u>H</u>$_2$), 5.27 (dd, *J* = 10.5, 1.5 Hz, 1H, CH=C<u>H</u>$_2$), 4.50 (d, *J* = 5.3 Hz, 2H, C5-OC<u>H</u>$_2$), 4.16 – 4.10 (m, 2H, C<u>H</u>$_2$-OAc), 4.02 – 3.97 (m, 2H, C2-OC<u>H</u>$_2$), 3.87 (s, 3H, Me), 2.03 (s, 3H, COC<u>H</u>$_3$), 1.91 – 1.80 (m, 4H, (C<u>H</u>$_2$)$_2$); **^{13}C-NMR** (101 MHz, $CDCl_3$) δ_C 171.3 (Ac), 166.7 (<u>C</u>O_2Me), 153.0 (C2), 152.2 (C5), 133.2 (<u>C</u>H=CH_2), 121.1 (C1), 120.6 (C4), 117.9 (CH=<u>C</u>H_2), 117.1 (C6), 115.4 (C3), 69.6 (C5-O<u>C</u>H_2), 69.3 (C2-O<u>C</u>H_2), 64.3 (<u>C</u>H_2-OAc), 52.1 (Me), 26.1 (<u>C</u>H_2), 25.4 (<u>C</u>H_2), 21.1 (CO<u>C</u>H_3); **LRMS** *m/z* (ESI+) 345.13 (100%, $[M+Na]^+$), 323.13 (60%, $[M+H]^+$); **HRMS** *m/z* (ESI+) $C_{17}H_{22}O_6Na$ Requires: 345.1309, Found: 345.1309 ($[M+Na]^+$).

(R-**8**) Methyl (R)-2-(4-acetoxybutoxy)-5-(2,3-dihydroxypropoxy)benzoate

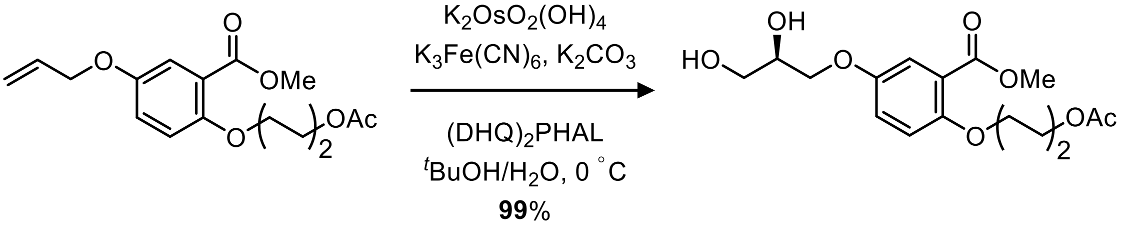

A 25 mL round bottom flask was charged with ADmix α (1.4 g, 3.0 mmol eq.), H_2O/^{t}BuOH (1:1, 10 mL) and stirred at room temperature for 2 min. The mixture was cooled to 0 ˚C (ice bath) for 10 min and methyl 2-(4-acetoxybutoxy)-5-(allyloxy)benzoate (**7**) (322 mg, 1.0 mmol) was added directly. The reaction mixture was stirred at 0 ˚C for 12 h with monitoring (EtOH/EtOAc 1:4, R_f = 0.50). The reaction was quenched with neat Na_2SO_3 (1.5 g) and warmed to room temperature over 1 h. The flask was charged with H_2O (10 mL) and extracted with EtOAc (4 × 30 mL). The combined organic extracts were washed with 1M HCl $_{(aq.)}$ (2 × 25 mL), brine (25 mL), and dried ($MgSO_4$). Filtration of the solids and removal of solvent under reduced pressure afforded the title compound as a clear oil (355 mg, 99%). **$[\alpha]_D^{25}$** +8.14 (c. 1.0, $CHCl_3$); **IR** ν_{max} cm^{-1} 3390 br s (O-H), 2950 w, 1734 s (C=O), 1715 s (C=O), 1611 w, 1580 w, 1498 s, 1468 w, 1438 s; **^{1}H-NMR** (400 MHz, $CDCl_3$) δ_H 7.32 (d, J = 3.2 Hz, 1H, C6-H), 6.99 (dd, J = 9.0, 3.2 Hz, 1H, C4-H), 6.86 (d, J = 9.0 Hz, 1H, C3-H), 4.24 – 4.03 (m, 2H, CH_2-OAc), 4.09 – 4.01 (m, 1H, CH-OH), 4.01 – 3.92 (m, 4H, C2-OCH_2 & C5-OCH_2), 3.85 (s, 3H, Me), 3.83 – 3.67 (m, 2H, CH_2-OH), 3.22 (br s, 1H, OH), 2.80 (br s, 1H, OH), 2.03 (s, 3H, COCH_3), 1.90 – 1.78 (m, 4H, $(CH_2)_2$); **^{13}C-NMR** (101 MHz, $CDCl_3$) δ_C 171.4 (Ac), 166.7 (CO_2Me), 153.2 (C2), 152.1 (C5), 121.0 (C1), 120.3 (C4), 117.0 (C6), 115.4 (C3), 70.5 (CH-OH), 69.7 (C5-OCH_2), 69.3 (C2-OCH_2), 64.3 (CH_2-OAc), 63.7 (CH_2-OH), 52.2 (Me), 26.0 (CH_2), 25.4 (CH_2), 21.1 (Ac); **LRMS** m/z (ESI+) 379.14 (100%, $[M+Na]^+$); **HRMS** m/z (ESI+) $C_{17}H_{24}NaO_8$ Requires: 379.1363, Found: 379.1362 ($[M+Na]^+$).

(S-**8**) Methyl (S)-2-(4-acetoxybutoxy)-5-(2,3-dihydroxypropoxy)benzoate

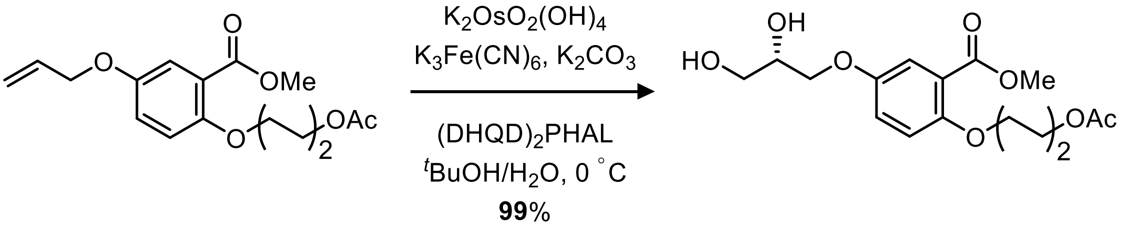

A 25 mL round bottom flask was charged with ADmix β (1.4 g, 3.0 mmol eq.), H_2O/^{t}BuOH (1:1, 10 mL) and stirred at room temperature for 2 min. The mixture was cooled to 0 ˚C (ice bath) for 10 min and methyl 2-(4-acetoxybutoxy)-5-(allyloxy)benzoate (**7**) (322 mg, 1.0 mmol) was added directly. The reaction mixture was stirred at 0 ˚C for 12 h with monitoring (EtOH/EtOAc 1:4, R_f = 0.50). The reaction was quenched with neat Na_2SO_3 (1.5 g) and warmed to room temperature over 1 h. The flask was charged with H_2O (10 mL) and extracted with EtOAc (4 × 30 mL). The combined organic extracts were washed with 1.0 M HCl $_{(aq.)}$ (2 × 25 mL), brine (25 mL), and dried ($MgSO_4$). Filtration of the solids and removal of solvent under reduced pressure gave the title compound as a clear oil (355 mg, 99%). **$[\alpha]_D^{25}$** –6.23 (c. 1.0, $CHCl_3$); **IR** ν_{max} cm^{-1} 3412 br s (O-H), 2980 s, 2901 s, 1733 s (C=O), 1716 s (C=O), 1611 w, 1580 w, 1498 s, 1470 w, 1438 s; **^{1}H-NMR** (400 MHz, $CDCl_3$) δ_H 7.32 (d, *J* = 3.2 Hz, 1H, C6-H), 6.99 (dd, *J* = 9.0, 3.2 Hz, 1H, C4-H), 6.86 (d, *J* = 9.0 Hz, 1H, C3-H), 4.24 – 4.03 (m, 2H, CH_2-OAc), 4.09 – 4.01 (m, 1H, CH-OH), 4.01 – 3.92 (m, 4H, C2-OCH_2 & C5-OCH_2), 3.85 (s, 3H, Me), 3.83 – 3.67 (m, 2H, CH_2-OH), 3.22 (br s, 1H, OH), 2.80 (br s, 1H, OH), 2.03 (s, 3H, CO_2CH_3), 1.90 – 1.78 (m, 4H, $(CH_2)_2$); **^{13}C-NMR** (101 MHz, $CDCl_3$) δ_C 171.4 (Ac), 166.7 (CO_2CH_3), 153.2 (C2), 152.0 (C5), 121.0 (C1), 120.3 (C4), 117.0 (C6), 115.4 (C3), 70.5 (CH-OH), 70.0 (C5-OCH_2), 69.2 (C2-OCH_2), 64.3 (CH_2-OAc), 63.7 (CH_2-OH), 52.2 (Me), 26.0 (CH_2), 25.4 (CH_2), 21.1 (Ac); **LRMS** *m/z* (ESI+) 379.14 (100%, $[M+Na]^+$); **HRMS** *m/z* (ESI+) $C_{17}H_{24}NaO_8$ Requires: 379.1363, Found: 379.1360 ($[M+Na]^+$).

(*R*-**9**) Methyl (*R*)-2-(4-hydroxybutoxy)-5-(2,3-dihydroxypropoxy)benzoate

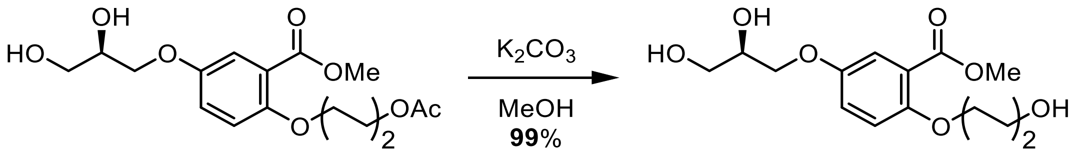

A solution of methyl (*R*)-2-(4-acetoxybutoxy)-5-(2,3-dihydroxypropoxy)benzoate (***R*-8**) (60 mg, 0.17 mmol) in MeOH (10 mL) was charged with K_2CO_3 (47 mg, 34.0 mmol). The reaction mixture was stirred at room temperature for 12 h. The reaction was slowly neutralised by the dropwise addition of conc. HCl $_{(aq.)}$ (pH 6) and filtered. The filtrate was collected and the solvent removed under reduced pressure to yield the title compound as a yellow oil (53 mg, 99%). **$[\alpha]_D^{25}$** +8.93 (c. 1.0, $CHCl_3$); **IR** ν_{max} cm^{-1} 3341 br s (O-H), 2988 s, 2901 s, 1712 s (C=O), 1610 w, 1579 w, 1498 s, 1467 w, 1438 s, 1417 w; **^{1}H-NMR** (400 MHz, CD_3OD) δ_H 7.30 (d, *J* = 3.2 Hz, 1H, C6-H), 7.11 (dd, *J* = 9.1, 3.2 Hz, 1H, C4-H), 7.02 (d, *J* = 9.1 Hz, 1H, C3-H), 4.08 – 3.98 (m, 3H, C2-OCH_2 & CH-OH), 3.98 – 3.91 (m, 2H, C5-OCH_2), 3.85 (s, 3H, CO_2CH_3), 3.74 – 3.58 (m, 4H, CH_2CH_2-OH, CH(OH)CH_2-OH), 1.91 – 1.78 (m, 2H, CH_2), 1.78 – 1.66 (m,

2H, C$\underline{H}_2$); **^{13}C-NMR** (101 MHz, CD_3OD) δ_C 168.4 ($\underline{C}O_2Me$), 154.2 (C2), 153.9 (C5), 122.2 (C1), 121.1 (C4), 117.7 (C6), 116.6 (C3), 71.8 (C5-O$\underline{C}H_2$), 71.1 ($\underline{C}$H-OH), 70.7 (C2-O$\underline{C}H_2$), 64.1 (CH(OH)$\underline{C}H_2$-OH), 62.6 ($CH_2\underline{C}H_2$-OH), 52.5 ($CO_2\underline{C}H_3$), 30.3 ($\underline{C}H_2$), 26.9 ($\underline{C}H_2$); **LRMS** *m/z* (ESI+) 337.13 (100%, $[M+Na]^+$); **HRMS** *m/z* (ESI+) $C_{15}H_{22}NaO_7$ Requires: 337.1258, Found: 337.1256 ($[M+Na]^+$).

(*S*-**9**) Methyl (*S*)-2-(4-hydroxybutoxy)-5-(2,3-dihydroxypropoxy)benzoate

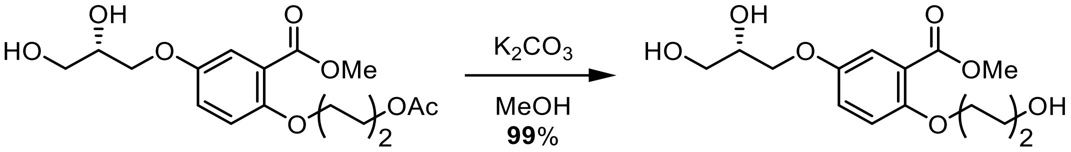

A solution of methyl (*S*)-2-(4-acetoxybutoxy)-5-(2,3-dihydroxypropoxy)benzoate (***S*-8**) (71 mg, 0.19 mmol) in MeOH (10 mL) was charged with K_2CO_3 (55 mg, 0.38 mmol). The reaction mixture was stirred at room temperature for 12 h. The reaction was slowly neutralised by the dropwise addition of conc. HCl $_{(aq.)}$ (pH 6) and filtered. The filtrate was collected and the solvent removed under reduced pressure to yield the title compound as a yellow oil (67 mg, 99%). **$[\alpha]_D^{25}$** –7.01 (c. 1.0, $CHCl_3$); **IR** $\tilde{\nu}_{max}$ cm^{-1} 3366 br s (O-H), 2942 s, 2876 s, 1712 s (C=O), 1610 w, 1579 w, 1498 s, 1467 w, 1438 s, 1417 w; **^{1}H-NMR** (400 MHz, CD_3OD) δ_H 7.30 (d, *J* = 3.2 Hz, 1H, C6-$\underline{H}$), 7.11 (dd, *J* = 9.1, 3.2 Hz, 1H, C4-$\underline{H}$), 7.02 (d, *J* = 9.1 Hz, 1H, C3-$\underline{H}$), 4.08 – 3.98 (m, 3H, C2-OC$\underline{H}_2$ & C$\underline{H}$-OH), 3.98 – 3.91 (m, 2H, C5-OC$\underline{H}_2$), 3.85 (s, 3H, $CO_2C\underline{H}_3$), 3.74 – 3.58 (m, 4H, $CH_2C\underline{H}_2$-OH, CH(OH)$\underline{C}H_2$-OH), 1.91 – 1.78 (m, 2H, C$\underline{H}_2$), 1.78 – 1.66 (m, 2H, C$\underline{H}_2$); **^{13}C-NMR** (101 MHz, CD_3OD) δ_C 168.4 ($\underline{C}O_2Me$), 154.2 (C2), 153.9 (C5), 122.2 (C1), 121.1 (C4), 117.7 (C6), 116.5 (C3), 71.8 (C5-O$\underline{C}H_2$), 71.1 ($\underline{C}$H-OH), 70.7 (C2-O$\underline{C}H_2$), 64.1 (CH(OH)$\underline{C}H_2$-OH), 62.6 ($CH_2\underline{C}H_2$-OH), 52.5 (Me), 30.3 ($\underline{C}H_2$), 26.9 ($\underline{C}H_2$); **LRMS** *m/z* (ESI+) 337.13 (100%, $[M+Na]^+$); **HRMS** *m/z* (ESI+) $C_{15}H_{22}NaO_7$ Requires: 337.1258, Found: 337.1260 ($[M+Na]^+$).

(*R*-**10**) Sodium (*R*)-3-(3-(methoxycarbonyl)-4-(4-(sulfonatooxy)butoxy)phenoxy)propane-1,2-diyl bis(sulfate)

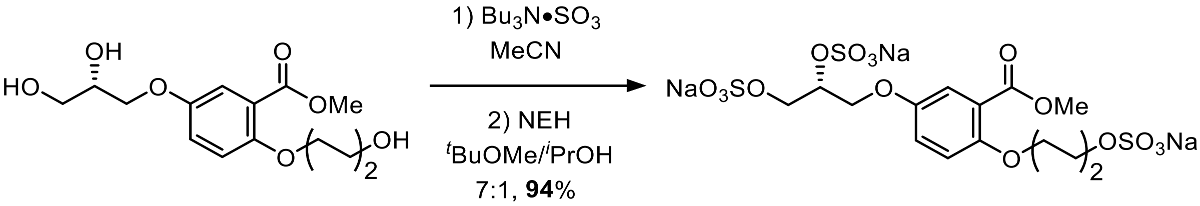

Adapted from General procedure **E**: A 25 mL Schlenk tube was charged with ***S*-9** (135 mg, 0.45 mmol) and $Bu_3N•SO_3$ (722 mg, 2.72 mmol) under Ar $_{(g)}$ and MeCN was added (1.5 mL). The flask was heated at 80 ˚C for 12 h with monitoring (TLC). The flask was cooled to room temperature and the solvent removed under reduced pressure to give a clear viscous oil. The crude oil was dissolved in ^{i}PrOH (5 mL) and transferred to a flask containing ^{t}BuOMe (35 mL). With vigorous stirring a solution of NEH (5.0 mL, 1.0 M) in ^{t}BuOMe/^{i}PrOH (1:7) was added dropwise over 10 min. The precipitate that formed was collected by filtration, washed with ^{i}PrOH (2 × 10 mL) and dried under vacuum. Recrystallization from H_2O/^{i}PrOH afforded the title compound as a white solid (260 mg, 94%). **$[\alpha]_D^{25}$**–3.11 (c. 1.0, $CHCl_3$); **M.P** 207 – 210 ˚C (dec.); **IR** Vmax cm^{-1} 2972 w, 2166 w, 1703 w (C=O), 1500 w, 1441 w, 1103 s (S=O); **^{1}H-NMR** (400 MHz, D_2O) δ_H 7.42 (d, *J* = 3.2 Hz, 1H, C6-H), 7.25 (dd, *J* = 9.2, 3.2 Hz, 1H, C4-H), 7.14 (d, *J* = 9.2 Hz, 1H, C3-H), 4.86 (p, *J* = 4.8 Hz, 1H, CH-OSO_3Na), 4.42 – 4.29 (m, 3H), 4.26 (dd, *J* = 11.0, 5.2 Hz, 1H), 4.16 – 4.05 (m, 4H), 3.90 (s, 3H, Me), 1.86 (m, 4H, $(CH_2)_2$); **^{13}C-NMR** (101 MHz, D_2O) δ_C 168.4 (CO_2Me), 152.6 (C2), 151.7 (C5), 121.6 (C4), 120.1 (C1), 117.3 (C6), 116.4 (C3), 75.0 (CH-OSO_3Na), 69.7, 69.0, 67.3, 66.4, 52.7 (Me), 25.2 ($(CH_2)_2$), 24.9 ($(CH_2)_2$); **LRMS** *m/z* (ESI+) 642.94 (40%, $[M+Na]^+$); **HRMS** *m/z* (ESI+) $C_{15}H_{19}Na_4O_{16}S_3$ Requires: 642.9421, Found: 642.9426 ($[M+Na]^+$).

(*S*-**10**) Sodium (*S*)-3-(3-(methoxycarbonyl)-4-(4-(sulfonatooxy)butoxy)phenoxy)propane-1,2-diyl bis(sulfate)

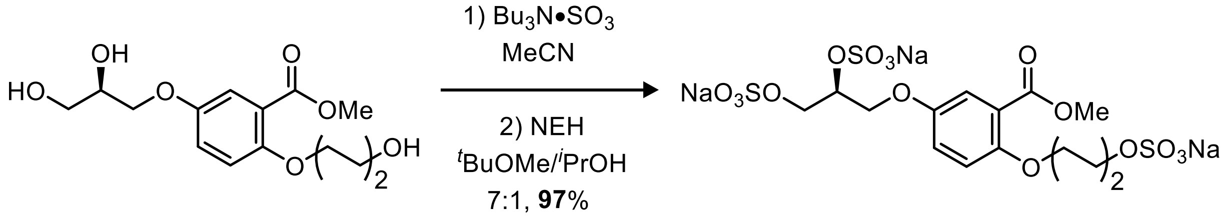

Adapted from General procedure **E**: a 25 mL Schlenk tube was charged with ***R*-9** (215 mg, 0.72 mmol) and $Bu_3N•SO_3$ (1.145 g, 2.56 mmol) under Ar $_{(g)}$ and MeCN was added (1.5 mL). The flask was heated at 80 ˚C for 12 h with monitoring (TLC). The flask was cooled to room temperature and the solvent removed under reduced pressure to give a clear viscous oil. The crude oil was dissolved in ^{i}PrOH (5 mL) and transferred to a flask containing ^{t}BuOMe (35 mL). With vigorous stirring a solution of NEH (5.0 mL, 1.0 M) in ^{t}BuOMe/^{i}PrOH (1:7) was added dropwise over 10 min. The precipitate that formed was collected by filtration, washed with ^{i}PrOH (2 × 10 mL) and dried under vacuum. Recrystallization from H_2O/^{i}PrOH afforded the title compound as a white solid (430 mg, 97%). **$[\alpha]_D^{25}$** +3.01 (c. 1.0, H_2O); **M.P** 207 – 210 ˚C (dec.); **IR** Vmax cm^{-1} 2971 w, 2164 w, 1710 w (C=O), 1500 w, 1441 w, 1103 s (S=O); **^{1}H-**

NMR (400 MHz, D_2O) δ_H 7.43 (d, *J* = 3.2 Hz, 1H, C6-H), 7.25 (dd, *J* = 9.2, 3.2 Hz, 1H, C4-H), 7.14 (d, *J* = 9.2 Hz, 1H, C3-H), 4.87 (p, *J* = 4.7 Hz, 1H, CH-OSO_3Na), 4.44 – 4.29 (m, 3H), 4.26 (dd, *J* = 11.0, 5.2 Hz, 1H), 4.12 (m, 4H), 3.90 (s, 3H, Me), 1.94 – 1.79 (m, 4H, $(CH_2)_2$); **^{13}C-NMR** (101 MHz, D_2O) δ_C 168.4 (CO_2Me), 152.6 (C2), 151.7 (C5), 121.6 (C4), 120.1 (C1), 117.3 (C6), 116.4 (C3), 75.0 (CH-OSO_3Na), 69.7, 69.0, 67.4, 66.4, 52.7 (Me), 25.2 ($(CH_2)_2$), 24.9 ($(CH_2)_2$); **LRMS** *m/z* (ESI+) 642.94 (50%, $[M+Na]^+$); **HRMS** *m/z* (ESI+) $C_{15}H_{19}Na_4O_{16}S_3$ Requires: 642.9421, Found: 642.9417 ($[M+Na]^+$).

(**14a**) Methyl 2,3-dihydroxybenzoate[S16]

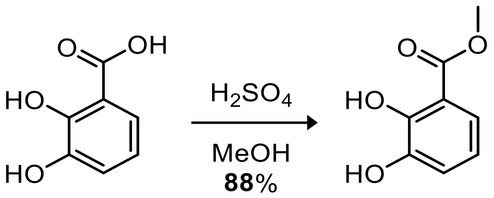

Following the general procedure **A**: using 2,3-dihydroxybenzoic acid, **13a** (5.00 g, 32.46 mmol). Recrystallization from CH_2Cl_2/hexane yielded the title compound as a pink solid (4.79 g, 88%). **M.P** 82 – 84 ˚C; **IR** Ѵmax cm^{-1} 3657 w (O-H), 3458 w (O-H), 2980 s, 1670 w (C=O), 1460 w; **^{1}H-NMR** (400 MHz, $CDCl_3$) δ_H 10.89 (s, 1H, C2-OH)), 7.36 (dd, J = 8.0, 1.5 Hz, 1H, C6-H), 7.16 – 7.06 (m, 1H, C4-H), 6.80 (t, J = 8.0 Hz, 1H, C5-H), 5.69 (s, 1H, C3-OH), 3.95 (s, 3H, Me); **^{13}C-NMR** (101 MHz, $CDCl_3$) δ_C 170.9 (CO_2Me), 148.9 (C2), 145.1 (C1), 120.7 (C6), 119.9 (C4), 119.3 (C5), 112.5 (C3), 52.6 (Me); **LRMS** *m/z* (ESI+) 169.05 (10%, $[M+H]^+$), 186.23 (100%, $[M+H_2O]^+$) Data were in accordance with the literature.[S16]

(**14b**) Methyl 2,4-dihydroxybenzoate[S17]

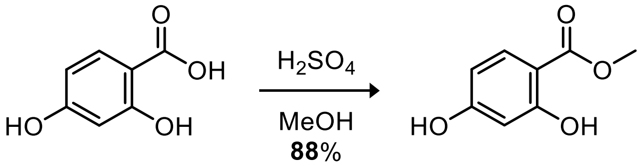

Following the general procedure **A**: using 2,4-dihydroxybenzoic acid, **13b** (5.00 g, 32.46 mmol). Recrystallization from CH_2Cl_2/hexane yielded the title compound as a white solid (4.79 g, 88%). **M.P** 114 – 117 ˚C; **IR** Ѵmax cm^{-1} 3085 w, 3019 w, 2950 w, 1720s (C=O), 1594 s; **^{1}H-NMR** (400 MHz, $CDCl_3$) δ_H 10.99 (s, 1H, C2-OH), 7.73 (d, *J* = 8.6 Hz, 1H, C6-H), 6.40 (d, *J* = 2.5 Hz, 1H, C3-H), 6.37 (dd, *J* = 8.6, 2.5 Hz, 1H, C5-H), 5.63 – 5.55 (m, 1H, C4-OH), 3.91 (s,

3H, Me); **^{13}C-NMR** (101 MHz, $CDCl_3$) δ_C 170.5 (<u>C</u>O_2Me), 163.7 (C4), 162.0 (C2), 132.1 (C6), 108.0 (C3), 106.0 (C1), 103.3 (C5), 52.2 (Me); **LRMS** *m/z* (ESI+) 186.23 (30%, $[M+H_2O]^+$). Data were in accordance with the literature.[S17]

(**14c**) Methyl 2,6-dihydroxybenzoate[S18]

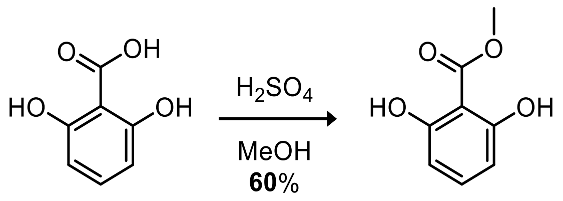

Following the general procedure **A**: using 2,6-dihydroxybenzoic acid, **13c** (5.00 g, 32.46 mmol). Recrystallization from CH_2Cl_2/hexane yielded the title compound as large off white crystals (3.26 g, 60%). **M.P** 83 – 85 ˚C; **IR** ν_{max} cm^{-1} 3412 br s (O-H), 3072 br w, 2968 w, 1667 m (C=O), 1574 s; **^{1}H-NMR** (400 MHz, $(CD_3)_2SO$) δ_H 9.95 (s, 2H, C2-O<u>H</u>), 7.09 (t, J = 8.2 Hz, 1H, C4-<u>H</u>), 6.34 (d, J = 8.2 Hz, 2H, C3-<u>H</u>), 3.78 (s, 3H, Me); **^{13}C-NMR** (101 MHz, $(CD_3)_2SO$) δ_C 168.3 (<u>C</u>O_2Me), 157.4 (C2), 132.4 (C4), 107.0 (C1), 106.7 (C3), 51.9 (Me); **LRMS** *m/z* (ESI+) 186.22 (20%, $[M+H_2O]^+$). Data were in accordance with the literature.[S10]

(**14d**) Methyl 3,4-dihydroxybenzoate[S19]

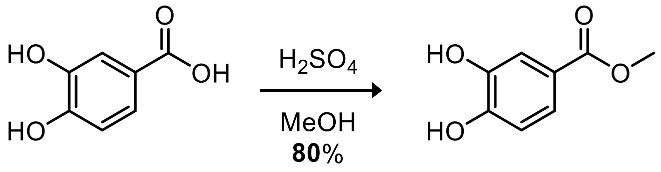

Following the general procedure **A**: using 3,4-dihydroxybenzoic acid, **13d** (5.00 g, 32.46 mmol). Recrystallization from CH_2Cl_2/hexane yielded the title compound as long fine white crystals (4.34 g, 80%). **M.P** 127 – 129 ˚C; **IR** ν_{max} cm^{-1} 3463 br s (O-H), 3254 br s (O-H), 2969, 1680 m (C=O), 1608 s, 1442 s; **^{1}H-NMR** (400 MHz, $(CD_3)_2SO$) δ_H 9.56 (Br s, 2H, Ar-O<u>H</u>), 7.35 (d, *J* = 2.1 Hz, 1H, C2<u>H</u>), 7.31 (dd, *J* = 8.3, 2.1 Hz, 1H, C6<u>H</u>), 6.80 (d, *J* = 8.3 Hz, 1H, C5<u>H</u>), 3.76 (s, 3H, Me); **^{13}C-NMR** (101 MHz, $(CD_3)_2SO$) δ_C 166.1 (<u>C</u>O_2Me), 150.4 (C4), 145.0 (C3), 121.7 (C6), 120.5 (C1), 116.2 (C2), 115.3 (C5), 51.6 (Me); **LRMS** *m/z* (ESI+) 191.03 (90%, $[M+Na]^+$), 209.04 (60%, $[M+Na+H_2O]^+$). Data were in accordance with the literature.[S19]

(**14e**) Methyl 3,5-dihydroxybenzoate[S20]

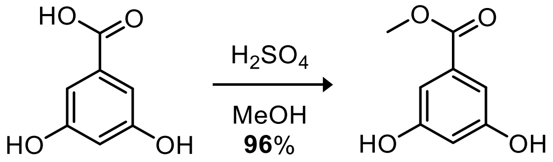

Following the general procedure **A**: using 3,5-dihydroxybenzoic acid, **13e** (5.00 g, 32.46 mmol). Recrystallization from MeOH yielded the title compound as a white solid (5.21 g, 96%). **M.P** 156 – 160 ˚C; **IR** Vmax cm^{-1} 3227 s br (O-H), 2998 w, 2952 w, 1687 s (C=O), 1600 s, 1486 w; **^{1}H-NMR** (400 MHz, $(CD_3)_2SO$) δ_H 9.62 (s, 2H, Ar-O<u>H</u>), 6.81 (d, *J* = 2.3 Hz, 2H, C2-<u>H</u>), 6.44 (t, *J* = 2.3 Hz, 1H, C4-<u>H</u>), 3.78 (s, 3H, Me); **^{13}C-NMR** (101 MHz, $(CD_3)_2SO$) δ_C 166.6 (<u>C</u>O_2Me), 158.9 (C3), 131.7 (C1), 107.6 (C4), 107.5 (C2), 52.4 (Me); **LRMS** *m/z* (ESI+) 186.22 (65%, $[M+Na+H_2O]^+$), 191.03 (90%, $[M+Na]^+$), 209.04 (45%, $[M+Na+H_2O]^+$). Data were in accordance with the literature.[S20]

(**14g**) Methyl salicylate[S21]

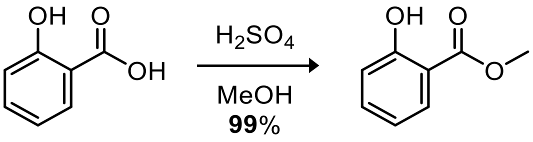

Following general procedure **A**: salicylic acid, **13g** (1.38 g, 10.0 mmol) in MeOH was charged with conc. H_2SO_4 and heated under reflux for 21 h with monitoring (EtOAc/hexane 1:3, R_f = 0.85). The title compound was afforded as a golden oil (1.50 g, 99%). **IR** Vmax cm^{-1} 3184 w (O-H), 2955 w, 1674 s (C=O), 1328 m, 1251 m; **^{1}H-NMR** (400 MHz, $CDCl_3$) δ_H 10.77 (s, 1H, C2-O<u>H</u>), 7.80 (dd, J = 8.2, 1.3 Hz, 1H, C6-<u>H</u>), 7.47 – 7.37 (m, 1H, C4-<u>H</u>), 6.96 (dd, J = 8.2, 1.3 Hz, 1H, C3-<u>H</u>), 6.85 (ddd, J = 8.2, 7.1, 1.3 Hz, 1H, C5-<u>H</u>), 3.91 (s, 3H, Me); **^{13}C-NMR** (101 MHz, $CDCl_3$) δ_c 170.5 (<u>C</u>O_2Me), 161.6 (C2), 135.6 (C4), 129.9 (C6), 119.1 (C5), 117.5 (C3), 112.3 (C1), 52.2 (Me); **LRMS** *m/z* (ESI+) 152.0 (50%, $[M]^+$), 120.0 (100%, $[M-CH_3OH]^+$), 92.1 (100%, $[C_7H_8]^+$). Data were in accordance with the literature.[S21]

(**14h**) Methyl 3-hydroxybenzoate[S22]

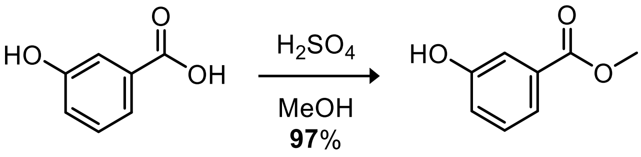

Following general procedure **A**: 3-hydroxybenzoic acid, **14h** (1.38g, 10.0 mmol) in MeOH was charged with conc. H_2SO_4 and heated under reflux for 7 h with monitoring (EtOAc/hexane 1:3, R_f = 0.41). A white solid was afforded after work up, recrystallization (CH_2Cl_2/hexane) yielded the title compound as large white crystals (1.48 g, 97%). **M.P** 85 – 86 ˚C; **IR** V̄max cm^{-1} 3385 br (O-H), 2953 w (C-H), 1695 m (C=O), 1454 m (C-C), 1292 s (C-O); ^{1}**H-NMR** (400 MHz, $(CD_3)_2CO$) δ_H 8.66 (s, 1H, C3-O<u>H</u>), 7.49 (dd, J = 8.0, 1.5 Hz, 2H, C6-<u>H</u> and C2-<u>H</u> stack), 7.33 (app. t, J = 8.0 Hz, 1H, C5-<u>H</u>), 7.12 – 7.07 (m, 1H, C4-<u>H</u>), 3.86 (s, 3H, Me); ^{13}**C-NMR** (101 MHz, $(CD_3)_2CO$) δ_C 167.1 (CO_2Me), 158.3 (C3), 132.5 (C1), 130.5 (C5), 121.3 (C2), 120.9 (C4), 116.7 (C6), 52.3 (Me); **LRMS** *m/z* (ESI+) 152.0 (95%, $[M]^+$), 121.0 (100%, $[M–CH_3OH]^+$), 93.0 (95%, $[C_6H_5OH]^+$). Data were in accordance with the literature.[S22]

(**14i**) Methyl 4-hydroxybenzoate[S23]

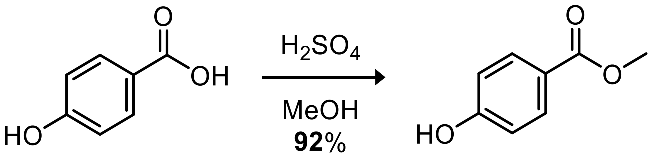

Following general procedure **A**: 4-hydroxybenzoic acid, **13i** (1.38g, 10.0 mmol) in MeOH was charged with conc. H_2SO_4 and heated under reflux for 10 h with monitoring (EtOAc/Hexane 1:3, R_f = 0.34). A white solid was afforded after work up, recrystallization (CH_2Cl_2/hexane) yielded the title compound as large white crystals (1.40 g, 92%). **M.P** 114 – 115 ˚C; **R** V̄max cm^{-1} 3287 br (O-H), 2964 w, 1683 s (C=O), 1616 w, 1502 s, 1436 s; ^{1}**H-NMR** (400 MHz, $CDCl_3$) δ_H 7.95 (d, *J* = 8.8 Hz, 2H, C3-<u>H</u>), 6.88 (d, *J* = 8.8 Hz, 2H, C2-<u>H</u>), 3.90 (s, 3H, Me); ^{13}**C-NMR** (101 MHz, $CDCl_3$) δ_C 167.5 (<u>C</u>O_2Me), 160.3 (C4), 132.1 (C3), 122.5 (C1), 115.4 (C2), 52.2 (Me); **LRMS** *m/z* (ESI+) 152.04 (60%, $[M]^+$), 121.0 (100%, $[M-CH_3OH]^+$); **HRMS** *m/z* (ESI+) $C_8H_8O_3$ Requires: 152.0473, Found: 152.0472 ($[M]^+$). Data were in accordance with the literature.[S23]

(**15a**) Methyl 2,3-bis(allyloxy)benzoate

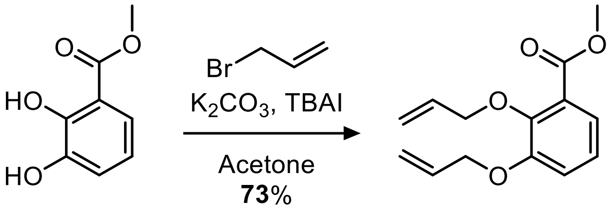

Following the general procedure **B** using: methyl 2,3-dihydroxy benzoate (**14a**) (1.00 g, 5.9 mmol), K_2CO_3 (2.45 g, 17.7 mmol), TBAI (0.71 g, 2.2 mmol) and allyl bromide (1.14 mL, 13.2 mmol). The flask was heated under reflux for 8 h. Purification by silica gel chromatography (EtOAc/hexane, 1:4, R_f = 0.28) yielded the title compound as a clear oil (1.22 g, 73%). **IR** $\tilde{\nu}_{max}$ cm^{-1} 3080 w, 3020 w, 2949 w, 1728 m (C=O), 1580 w (C=C), 1472 m; **^{1}H-NMR** (400 MHz, $CDCl_3$) δ_H 7.31 (t, *J* = 4.8 Hz, 1H, C5-$\underline{H}$), 7.02 (d, *J* = 4.8 Hz, 2H, C4-$\underline{H}$/C6-$\underline{H}$), 6.21 – 5.96 (m, 2H, C$\underline{H}$=CH_2), 5.45 – 5.30 (m, 2H, CH=C$\underline{H}_2$), 5.29 – 5.17 (m, 2H, CH=C$\underline{H}_2$), 4.61 – 4.52 (m, 4H, Ar-OC$\underline{H}_2$), 3.86 (s, 3H, Me); **^{13}C-NMR** (101 MHz, $CDCl_3$) δ_C 166.8 ($\underline{C}O_2$Me), 152.6 (C3), 148.2 (C2), 134.2 ($\underline{C}$H=CH_2), 132.9 ($\underline{C}$H=CH_2), 126.6 (C1), 123.8, 122.6 (C5), 117.8, 117.6 (2C, CH=$\underline{C}H_2$), 74.7 (Ar-O$\underline{C}H_2$), 69.9 (Ar-O$\underline{C}H_2$), 52.1 (Me); **LRMS** *m/z* (ESI+) 271.09 (100%, $[M+Na]^+$); **HRMS** *m/z* (ESI+) $C_{14}H_{16}NaO_4$ Requires: 271.0941, Found: 271.0949 ($[M+Na]^+$).

(**15b**) Methyl 2,4-bis(allyloxy)benzoate

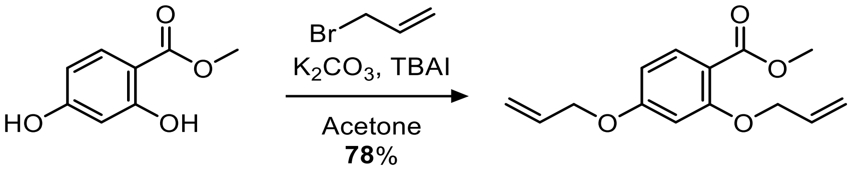

Following the general procedure **B** using: methyl 2,4-dihydroxy benzoate (**14b**) (1.00 g, 5.9 mmol), K_2CO_3 (2.45 g, 17.7 mmol), TBAI (0.71 g, 2.2 mmol) and allyl bromide (1.14 mL, 13.2 mmol). The flask was heated under reflux for 8 h. Purification by silica gel chromatography (EtOAc/hexane, 1:4, R_f = 0.28) yielded the title compound as a clear oil (1.30 g, 78%). **IR** $\tilde{\nu}_{max}$ cm^{-1} 3082 w, 2948 w, 1721 s (C=O), 1605 s (C=C); **^{1}H-NMR** (400 MHz, $CDCl_3$) δ_H 7.83 (m, 1H, C3-$\underline{H}$), 6.48 (m, 2H, C5-$\underline{H}$ & C6-$\underline{H}$), 6.03 (m, 2H, C$\underline{H}$=CH_2), 5.53 (dd, J = 17.2, 1.8 Hz, 1H, CH=C$\underline{H}_2$), 5.40 (dd, J = 17.2, 1.5 Hz, 1H, CH=C$\underline{H}_2$), 5.29 (dd, J = 10.6, 1.5 Hz, 2H, CH=C$\underline{H}_2$), 4.58 (dt, J = 4.8, 1.8 Hz, 2H, Ar-OC$\underline{H}_2$), 4.54 (dt, J = 5.4, 1.5 Hz, 2H, Ar-OC$\underline{H}_2$), 3.84 (s, 3H, Me); **^{13}C-NMR** (101 MHz, $CDCl_3$) δ_C 166.1 ($\underline{C}O_2$Me), 163.0 (C2), 160.2 (C4), 133.8 (C3), 132.6 ($\underline{C}$H=CH_2), 118.2 (CH=$\underline{C}H_2$), 117.4 (CH=$\underline{C}H_2$), 112.8 (C1), 105.7 (C6), 101.0 (C5),

69.4 (Ar-O<u>C</u>H$_2$), 68.9 (Ar-O<u>C</u>H$_2$), 51.7 (Me); **LRMS** *m/z* (ESI+) 271.10 (100%, [M+Na]$^+$); **HRMS** *m/z* (ESI+) $C_{14}H_{16}NaO_4$ Requires: 271.0941, Found: 271.0940 ([M+Na]$^+$).

(**15c**) Methyl 2,6-bis(allyloxy)benzoate

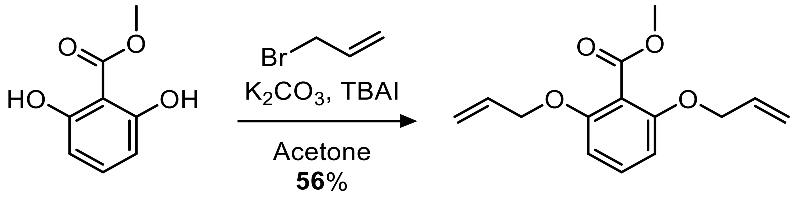

Following the general procedure **B** using: methyl 2,6-dihydroxy benzoate (**14c**) (1.00 g, 5.9 mmol), K_2CO_3 (2.45 g, 17.7 mmol), TBAI (0.71 g, 2.2 mmol) and allyl bromide (1.14 mL, 13.2 mmol). The flask was heated under reflux for 8 h. Purification by silica gel chromatography (EtOAc/hexane, 1:4, R_f = 0.28) yielded the title compound as a green oil (0.94 g, 56%). **IR** V̄max cm^{-1} 3087 w, 3021 w, 2948 w, 1731 s (C=O), 1596 s (C=C), 1469 w; ^{1}**H-NMR** (400 MHz, $CDCl_3$) δ_H 7.22 (t, 1H, *J* = 8.4 Hz, C4-<u>H</u>), 6.54 (d, *J* = 8.4 Hz, 2H, C3-<u>H</u>), 5.98 (ddt, *J* = 17.2, 10.6, 4.8 Hz, 2H, C<u>H</u>=CH$_2$), 5.37 (dd, *J* = 17.2, 1.6 Hz, 2H, CH=C<u>H</u>$_2$), 5.24 (dd, *J* = 10.6, 1.6 Hz, 2H, CH=C<u>H</u>$_2$), 4.55 (dt, *J* = 4.8, 1.6 Hz, 4H, C2-OC<u>H</u>$_2$), 3.90 (s, 3H, Me); ^{13}**C-NMR** (101 MHz, $CDCl_3$) δ_C 166.8 (<u>C</u>O$_2$Me), 156.4 (C2), 132.8 (<u>C</u>H=CH$_2$), 130.8 (C4), 117.2 (CH=<u>C</u>H$_2$), 114.0 (C1), 105.5 (C3), 69.3 (C2-O<u>C</u>H$_2$), 52.3 (Me); **LRMS** *m/z* (ESI+) 249.10 (5%, [M]$^+$), 271.09 (100%, [M+Na]$^+$); **HRMS** *m/z* (ESI+) $C_{14}H_{16}NaO_4$ Requires: 271.0941, Found: 271.0939 ([M+Na]$^+$).

(**15d**) Methyl 3,4-bis(allyloxy)benzoate

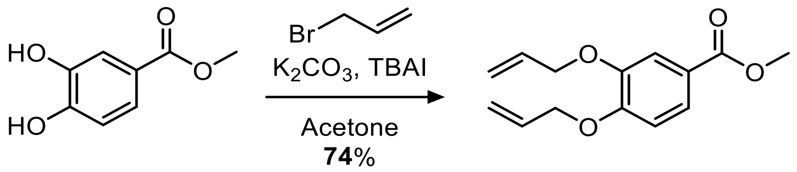

Following the general procedure **B** using: methyl 3,4-dihydroxy benzoate (**14d**) (1.00 g, 5.9 mmol), K_2CO_3 (2.45 g, 17.7 mmol), TBAI (0.71 g, 2.2 mmol) and allyl bromide (1.14 mL, 13.2 mmol). The flask was heated under reflux for 8 h. Purification by silica gel chromatography (EtOAc/hexane, 1:4, R_f = 0.28) yielded the title compound as a clear oil (1.24 g, 74%). **IR** V̄max cm^{-1} 3083 w, 2949 w, 2865 w, 1712 s (C=O), 1598 w (C=C); ^{1}**H-NMR** (400 MHz, $CDCl_3$) δ_H 7.63 (dd, *J* = 8.5, 2.0 Hz, 1H, C6-<u>H</u>), 7.55 (d, *J* = 2.0 Hz, 1H, C2-<u>H</u>), 6.88 (d, *J* = 8.5 Hz,

1H, C5-H), 6.21 – 5.95 (m, 2H, CH=CH_2), 5.52 – 5.37 (m, 2H, CH=CH_2), 5.29 (qd, J = 10.5, 2.8 Hz, 2H, CH=CH_2),), 4.74 – 4.57 (m, 4H, Ar-OCH_2), 3.87 (s, 3H, Me); **^{13}C-NMR** (101 MHz, $CDCl_3$) δ_C 166.8 (CO_2Me), 152.4 (C4), 147.9 (C3), 133.0 (CH=CH_2), 132.7 (CH=CH_2), 123.7 (C6), 122.8 (C1), 118.1 (CH=CH_2), 118.0 (CH=CH_2), 114.5 (C2), 112.4 (C5), 69.8 (Ar-OCH_2), 69.6 (Ar-OCH_2), 52.0 (Me); **LRMS** *m/z* (ESI+) 249.09 (2%, $[M]^+$), 271.09 (90%, $[M+Na]^+$); **HRMS** *m/z* (ESI+) $C_{14}H_{16}NaO_4$ Requires: 271.0941, Found: 271.0941 ($[M+Na]^+$).

(**15e**) Methyl 3,5-bis(allyloxy)benzoate

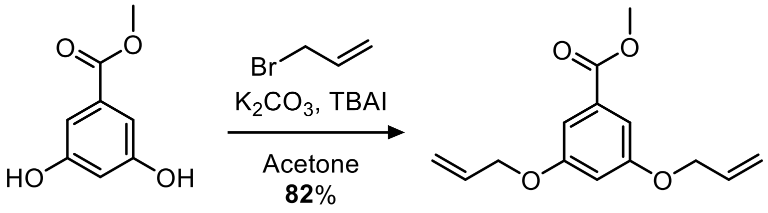

Following the general procedure **B** using: methyl 3,5-dihydroxy benzoate (**14e**) (1.00 g, 5.9 mmol), K_2CO_3 (2.45 g, 17.7 mmol), TBAI (0.71 g, 2.2 mmol) and allyl bromide (1.14 mL, 13.2 mmol). The flask was heated under reflux for 8 h. Purification by silica gel chromatography (EtOAc/hexane, 1:4, R_f = 0.28) yielded the title compound as a transparent solid (1.38 g, 82%). **M.P** 39 ˚C; **IR** Vmax cm^{-1} 2990 w, 2951 w, 1720 s (C=O), 1595 s (C=C), 1441 s; **^{1}H-NMR** (400 MHz, $(CD_3)_2CO$) δ_H 7.17 (d, J = 2.4 Hz, 2H, C2-H), 6.80 (t, J = 2.4 Hz, 1H, C4-H), 6.09 (ddt, J = 16.1, 10.5, 5.2 Hz, 2H, CH=CH_2), 5.45 (dd, J = 17.3, 1.3 Hz, 2H, CH=CH_2), 5.28 (dd, J = 10.5, 1.3 Hz, 2H, CH=CH_2), 4.64 (d, J = 5.2 Hz, 4H, C3-OCH_2), 3.88 (s, 3H, Me); **^{13}C-NMR** (101 MHz, $(CD_3)_2CO$) δ_C 166.0 (CO_2Me), 159.8 (C3), 133.4 (CH=CH_2), 132.2 (C1), 116.7 (CH=CH_2), 107.9 (C2), 106.3 (C4), 68.7 (C3-OCH_2), 51.6 (Me); **LRMS** *m/z* (ESI+) 249.10 (4%, $[M]^+$), 271.09 (90%, $[M+Na]^+$); **HRMS** *m/z* (ESI+) $C_{14}H_{16}NaO_4$ Requires: 271.0941, Found: 271.0942 ($[M+Na]^+$).

(**15f**) 1,4-Bis(allyloxy)benzene[S24]

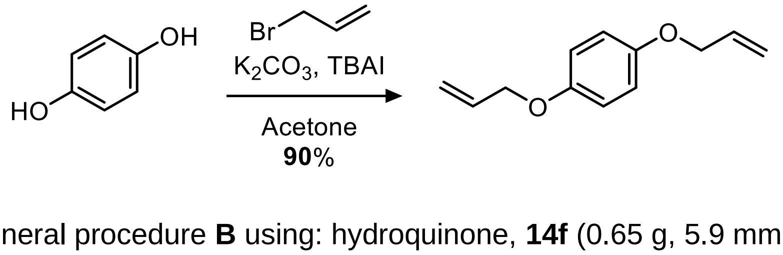

Following the general procedure **B** using: hydroquinone, **14f** (0.65 g, 5.9 mmol), K_2CO_3 (2.45 g, 17.7 mmol), TBAI (0.71 g, 2.2 mmol) and allyl bromide (1.14 mL, 13.2 mmol). The flask was

heated under reflux for 8 h. Purification by silica gel chromatography (EtOAc/hexane, 1:4, R_f = 0.40) yielded the title compound as a crystalline yellow solid (1.04 g, 90%). **M.P** 36 – 37 ˚C; **IR** Ṽmax cm^{-1} 3020 w, 2859 w, 1506 s, 1457 w; **^{1}H-NMR** (400 MHz, $CDCl_3$) δ_H 6.85 (s, 4H, Ar-H), 6.05 (ddt, *J* = 17.1, 10.5, 5.4 Hz, 2H, CH=CH_2), 5.40 (dd, *J* = 17.1, 1.5 Hz, 2H, CH=CH_2), 5.27 (dd, *J* = 10.5, 1.5 Hz, 2H, CH=CH_2), 4.49 (d, *J* = 5.4 Hz, 4H, Ar-OCH_2); **^{13}C-NMR** (101 MHz, $CDCl_3$) δ_C 153.0 (Ar-OCH_2), 133.7 (CH=CH_2), 117.6 (CH=CH_2), 115.8 (Ar-H), 69.6 (Ar-OCH_2); **LRMS** *m/z* (ESI+) 190.09 (90%, $[M]^+$), 149.10 (100%, $[M-C_3H_5]^+$). Data were in accordance with the literature.[S24]

(**15g**) Methyl 2-(allyloxy)benzoate

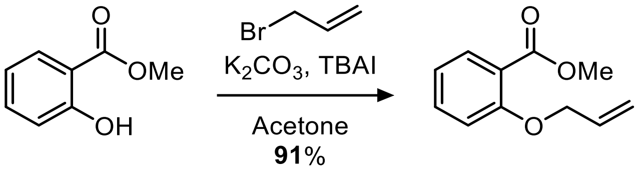

Following general procedure **B**: methyl salicylate (**14g**) (760 mg, 5.0 mmol), K_2CO_3 (829 mg, 6.0 mmol) and TBAI (806 mg, 2.5 mmol) in acetone (25 mL) was charged with allyl bromide (0.52 mL, 6.0 mmol) and heated under reflux for 4 h. Purification by chromatography (SiO_2, EtOAc/hexane 1:9, R_f = 0.55) yielded the title compound as a clear oil (882 mg, 91%). **IR** Ṽmax cm^{-1} 2951 w, 1723 s (C=O), 1599 m, 1489 m, 1449; **^{1}H-NMR** (400 MHz, $CDCl_3$) δ_H 7.77 (dd, *J* = 7.5, 1.8 Hz, 1H, C6-H), 7.39 (ddd, *J* = 8.3, 7.5, 1.8 Hz, 1H, C5-H), 6.99 – 6.86 (m, 2H, C4-H & C3-H), 6.02 (ddt, *J* = 17.3, 10.6, 4.9 Hz, 1H, CH=CH_2), 5.49 (dq, *J* = 17.3, 1.7 Hz, 1H, CH=CH_2), 5.26 (dq, *J* = 10.6, 1.7 Hz, 1H, CH=CH_2), 4.57 (dt, *J* = 4.9, 1.7 Hz, 2H, C2-OCH_2), 3.85 (s, 3H, Me); **^{13}C-NMR** (101 MHz, $CDCl_3$) δ_C 166.7 (CO_2Me), 158.0 (C2), 133.3 (C5), 132.7 (CH=CH_2), 131.6 (C6), 120.5 (C1), 120.3 (C4), 117.2 (CH=CH_2), 113.6 (C3), 69.3 (C2-OCH_2), 51.9 (Me); **LRMS** *m/z* (ESI+) 192.08 (80%, $[M]^+$), 160.05 (70%, $[M–CH_3OH]^+$), 132.05 (100%); **HRMS** *m/z* (ESI+) $C_{11}H_{12}O_3$ Requires: 192.0786, Found: 192.0784 ($[M]^+$).

(**15h**) Methyl 3-(allyloxy)benzoate

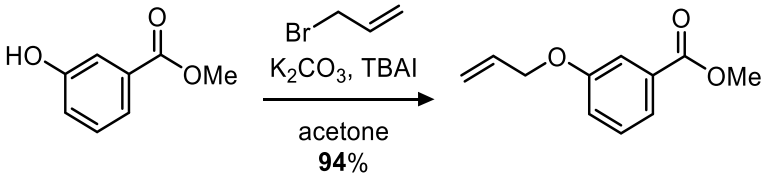

Following general procedure **B**: methyl 3-hydroxybenzoate (**14h**) (760 mg, 5.0 mmol), K_2CO_3 (829 mg, 6.0 mmol) and TBAI (806 mg, 2.5 mmol) in acetone (25 mL) was charged with allyl bromide (0.52 mL, 6.0 mmol) and heated under reflux for 4 h. Purification by chromatography (SiO_2, EtOAc/hexane 1:9, R_f = 0.74) yielded the title compound as a clear oil (900 mg, 94%). **IR** V̄max cm^{-1} 3020 w, 2950 w, 1720 s (C=O), 1586 w, 1445 w; **^{1}H-NMR** (400 MHz, $CDCl_3$) δ_H 7.63 (dt, *J* = 8.0, 1.2 Hz, 1H, C6-H), 7.57 (dd, *J* = 2.7, 1.2 Hz, 1H, C2-H), 7.33 (app. t, *J* = 8.0 Hz, 1H, C5-H), 7.11 (ddd, *J* = 8.0, 2.7, 1.2 Hz, 1H, C4-H), 6.05 (ddt, *J* = 17.3, 10.5, 5.2 Hz, 1H, CH=CH_2), 5.42 (dd, *J* = 17.3, 1.5 Hz, 1H, CH=CH_2), 5.29 (dd, *J* = 10.5, 1.5 Hz, 1H, CH=CH_2), 4.57 (d, *J* = 5.2 Hz, 2H, C3-OCH_2), 3.90 (s, 3H, Me); **^{13}C-NMR** (101 MHz, $CDCl_3$) δ_C 167.0 (CO_2Me), 158.6 (C3), 133.0 (CH=CH_2), 131.5 (C1), 129.5 (C5), 122.3 (C6), 120.3 (C4), 118.0 (CH=CH_2), 115.0 (C2), 69.0 (C3-OCH_2), 52.3 (Me). **LRMS** *m/z* (ESI+) 192.06 ([M]$^+$, 100%), 177.04 (80%, [M–CH_3]$^+$), 161.05 (65%, [M–CH_3OH]$^+$), 92.02 (70%); **HRMS** *m/z* (ESI+) $C_{11}H_{12}O_3$ Requires: 192.0786, Found: 192.0785 ([M]$^+$).

(**15i**) Methyl 4-(allyloxy)benzoate

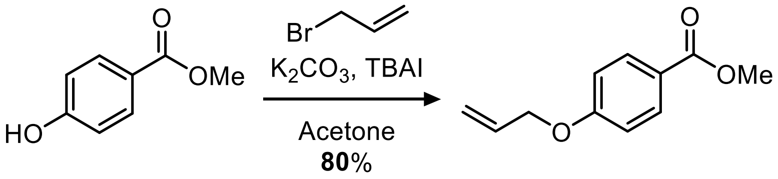

Following general procedure **B**: methyl 4-hydroxybenzoate (**14i**) (760 mg, 5.0 mmol), K_2CO_3 (829 mg, 6.0 mmol) and TBAI (806 mg, 2.5 mmol) in acetone (25 mL) was charged with allyl bromide (0.52 mL, 6.0 mmol) and heated under reflux for 4 h. Purification by chromatography (SiO_2, EtOAc/hexane 1:9, R_f = 0.57) yielded the title compound as a clear oil (768 mg, 80%); **IR** V̄max cm^{-1} 2951 w, 1712 s (C=O), 1603 s, 1579 w, 1509 s, 1434 s; **^{1}H-NMR** (400 MHz, $CDCl_3$) δ 7.99 – 7.93 (m, 2H, C3-H), 6.93 – 6.87 (m, 2H, C2-H), 6.02 (ddt, *J* = 17.3, 10.5, 5.3 Hz, 1H, CH=CH_2), 5.40 (dq, *J* = 17.3, 1.5 Hz, 1H, CH=CH_2), 5.29 (dq, *J* = 10.5, 1.5 Hz, 1H, CH=CH_2), 4.55 (dt, *J* = 5.3, 1.6 Hz, 2H, C4-OCH_2), 3.86 (s, 3H, Me); **^{13}C-NMR** (101 MHz, $CDCl_3$) δ 166.8 (CO_2Me), 162.3 (C4), 132.6 (CH=CH_2), 131.6 (C3), 122.7 (C1), 118.1 (CH=CH_2), 114.3 (C2), 68.8 (C4-OCH_2), 51.9 (Me); **LRMS** *m/z* (ESI+) 192.06 (100%, [M]$^+$), 161.04 (80%, [M–CH_3OH]$^+$), 133.06 (70%); **HRMS** *m/z* (ESI+) $C_{11}H_{12}O_3$ Requires: 192.0786, Found: 192.0782 ([M]$^+$).

(**16a**) Methyl 2,3-bis(2,3-dihydroxypropoxy)benzoate

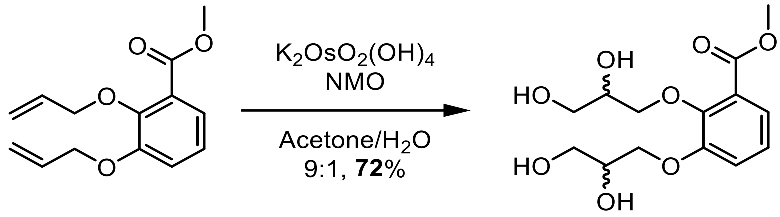

Following the general procedure **C**: Methyl 2,3-bis(allyloxy)benzoate (**15a**) (0.25 g, 1.0 mmol) was added dropwise and the reaction mixture was stirred vigorously at RT for 24 h. Purification by silica gel chromatography (EtOH/EtOAc, 1:4, R_f = 0.24) afforded the title compound as a clear oil (0.19 g, 72%). **IR** ṽmax cm^{-1} 3346 s br (O-H), 2943 w, 2891 w, 1710 s (C=O), 1477 s; **^{1}H-NMR** (400 MHz, CD_3OD) δ_H 7.32 (dd, *J* = 8.0, 1.5 Hz, 1H, C6-H), 7.24 (dd, *J* = 8.0, 1.5 Hz, 1H, C4-H), 7.12 (t, *J* = 8.0 Hz, 1H, C5-H), 4.23 (ddd, *J* = 9.7, 4.2, 2.0 Hz, 1H, C2-OCH_2), 4.17 – 3.99 (m, 4H, C2-OCH_2 & C3-OCH_2, CH-OH), 3.99 – 3.92 (m, 1H, CH-OH), 3.88 (s, 3H, Me), 3.75 – 3.61 (m, 4H, CH_2-OH); **^{13}C-NMR** (101 MHz, CD_3OD) δ_C 168.5 (CO_2Me), 154.0 (C3), 149.5 (C2), 126.6 (C1), 125.1 (C5), 123.5 (C6), 118.7 (C4), 76.45 (C2-OCH_2), 76.42 (C2-OCH_2), 72.43 (CH-OH), 72.42 (CH-OH), 71.7 (C3-OCH_2), 71.5 (CH-OH), 64.10 (CH_2-OH), 64.07 (CH_2-OH), 52.8 (Me); **LRMS** *m/z* (ESI+) 281.10 (100%, $[M-H_2O_2]^+$); **HRMS** *m/z* (ESI+) $C_{14}H_{18}O_6$Na Requires: 281.1001, Found: 281.9999 ($[M-H_2O_2]^+$).

(**16b**) Methyl 2,4-bis(2,3-dihydroxypropoxy)benzoate

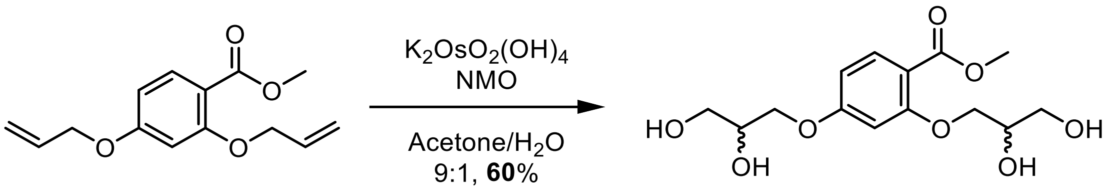

Following the general procedure **C**: Methyl 2,3-bis(allyloxy)benzoate (**15b**) (0.25 g, 1.0 mmol) was added dropwise and the reaction mixture was stirred vigorously at RT for 24 h. Purification by silica gel chromatography (EtOH/EtOAc, 1:4, R_f = 0.24) afforded the title compound as a clear oil (0.15 g, 60%). **IR** ṽmax cm^{-1} 3346 br s, 2943 w, 1703 s (C=O), 1609 w, 1437 s; **^{1}H-NMR** (400 MHz, CD_3OD) δ_H 7.81 (d, *J* = 8.8 Hz, 1H, C6-H), 6.69 (d, *J* = 2.3 Hz, 1H, C3-H), 6.62 (dd, *J* = 8.8, 2.3 Hz, 1H, C5-H), 4.17 – 3.94 (m, 6H, Ar-OCH_2 & CH-OH), 3.83 (s, 3H, Me), 3.78 – 3.56 (m, 4H, CH_2-OH); **^{13}C-NMR** (101 MHz, CD_3OD) δ_C 168.0 (CO_2Me), 165.4 (C4), 162.3 (C2), 134.7 (C6), 113.3 (C1), 107.5 (C5), 101.7 (C3), 72.0 (Ar-OCH_2), 71.6 (CH-OH), 71.4 (CH-OH), 70.7 (Ar-OCH_2), 64.3 (CH_2-OH), 64.0 (CH_2-OH), 52.2 (Me); **LRMS** *m/z* (ESI+)

339.10 (100%, [M+Na]+); **HRMS** *m/z* (ESI+) $C_{14}H_{20}O_8Na$ Requires: 339.1050, Found: 339.1053 ([M+Na]+).

(**16c**) Methyl 2,6-bis(2,3-dihydroxypropoxy)benzoate

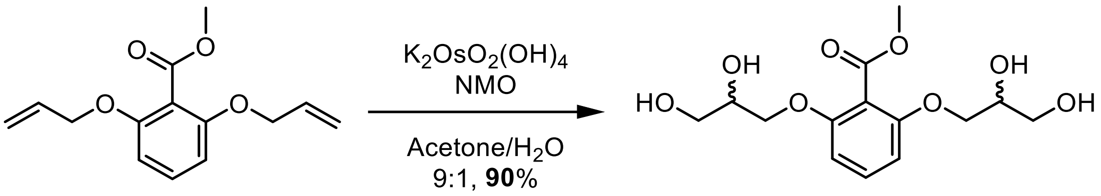

Following the general procedure **C**: Methyl 2,6-bis(allyloxy)benzoate (**15c**) (0.25 g, 1.0 mmol) was added dropwise and the reaction mixture was stirred vigorously at RT for 24 h. Purification by silica gel chromatography (EtOH/EtOAc, 1:4, R_f = 0.24) afforded the title compound as a clear oil (0.22 g, 90%). **M.P** 95 – 98 ˚C; **IR** Vmax cm^{-1} 3313 w br (O-H), 2939 w, 2880 w, 1730s (C=O), 1597 s, 1455 s; **^{1}H-NMR** (400 MHz, CD_3OD) δ_H 7.33 (t, *J* = 8.4 Hz, 1H, C4-H), 6.71 (d, *J* = 8.4 Hz, 2H, C3-H), 4.14 – 3.98 (m, 4H, C2-OCH_2), 3.91 (p, *J* = 5.4 Hz, 2H, CH-OH), 3.87 (s, 3H, Me), 3.71 – 3.55 (m, 4H, CH_2-OH); **^{13}C-NMR** (101 MHz, CD_3OD) δ_C 169.1 (CO_2Me), 158.1 (C2), 132.7 (C4), 114.9 (C1), 106.7 (C3), 71.6 (CH-OH), 71.2 (C2-OCH_2), 64.1 (CH_2-OH), 52.8 (Me); **LRMS** *m/z* (ESI+) 339.11 (100%, [M+Na]+); **HRMS** *m/z* (ESI+) $C_{14}H_{20}O_8Na$ Requires: 339.1050, Found: 339.1052 ([M+Na]+).

(**16d**) Methyl 3,4-bis(2,3-dihydroxypropoxy)benzoate

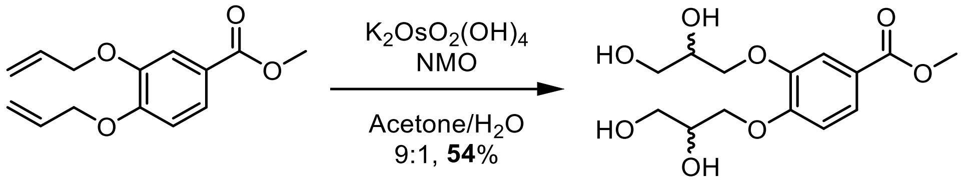

Following the general procedure **C**: Methyl 3,4-bis(allyloxy)benzoate (**15d**) (0.25 g, 1 mmol) was added dropwise and the reaction mixture was stirred vigorously at RT for 24 h. Purification by silica gel chromatography (EtOH/EtOAc, 1:4, R_f = 0.24) afforded the title compound as a clear oil (0.13 g, 54%). **M.P** 92 – 93 ˚C; **IR** Vmax cm^{-1} 3302 br s (O-H), 2942 w, 1714 s (C=O), 1596 s; **^{1}H-NMR** (400 MHz, CD_3OD) δ_H 7.66 (dd, *J* = 8.5, 2.0 Hz, 1H, C6-H), 7.60 (d, *J* = 2.0 Hz, 1H, C2-H), 7.06 (d, *J* = 8.5 Hz, 1H, C5-H), 4.29 – 3.92 (m, 6H, Ar-OCH_2 & CH-OH), 3.87 (s, 3H, Me), 3.77 – 3.63 (m, 4H, CH_2-OH); **^{13}C-NMR** (101 MHz, CD_3OD) δ_C 168.3 (CO_2Me), 154.6 (C3), 149.8 (C4), 125.2 (C6), 124.0 (C1), 115.8 (C2), 113.6 (C5), 71.9, 71.7, 71.6, 71.5,

64.1 (CH_2-OH), 64.0 (CH_2-OH), 52.5 (Me); **LRMS** *m/z* (ESI+) 339.11 (100%, $[M+Na]^+$); **HRMS** *m/z* (ESI+) $C_{14}H_{20}NaO_8$ Requires: 339.1050, Found: 339.1052 ($[M+Na]^+$).

(**16e**) Methyl 3,5-bis(2,3-dihydroxypropoxy)benzoate

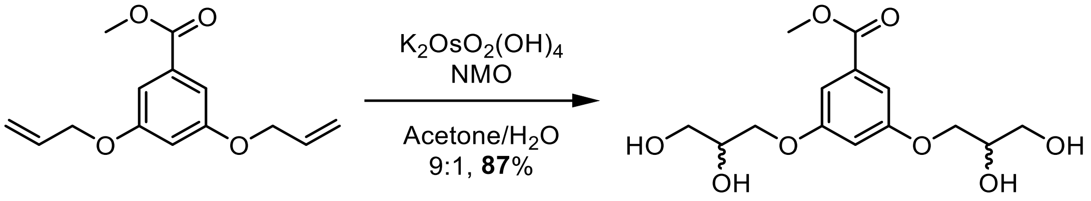

Following the general procedure **C**: Methyl 3,5-bis(allyloxy)benzoate (**15e**) (0.25 g, 1.0 mmol) was added dropwise and the reaction mixture was stirred vigorously at RT for 24 h. Purification by silica gel chromatography (EtOH/EtOAc, 1:4, R_f = 0.24) afforded the title compound as a clear oil (0.22 g, 87%). **M.P** 93 – 96 ˚C; **IR** ν_{max} cm^{-1} 3277 s br (O-H), 2943 w, 1711 s (C=O), 1601 m, 1446 m; **^{1}H-NMR** (400 MHz, CD_3OD) δ_H 7.19 (d, *J* = 2.3 Hz, 2H, C2-H), 6.81 (t, *J* = 2.3 Hz, 1H, C4-H), 4.14 – 4.04 (m, 2H, CH-OH), 4.04 – 3.92 (m, 4H, C3-OCH_2), 3.89 (s, 3H, Me), 3.74 – 3.59 (m, 4H, CH_2-OH); **^{13}C-NMR** (101 MHz, CD_3OD) δ_C 168.2 (CO_2Me), 161.5 (C3), 133.2 (C1), 109.0 (C2), 107.5 (C4), 71.7 (C3-OCH_2), 70.7 (CH-OH), 64.1 (CH_2-OH), 52.8 (Me); **LRMS** *m/z* (ESI+) 339.10 (100%, $[M+Na]^+$); **HRMS** *m/z* (ESI+) $C_{14}H_{20}NaO_8$ Requires: 339.1050, Found: 339.1047 ($[M+Na]^+$).

(**16f**) 1,4-Phenylenebis(oxy))bis(propane-1,2-diol

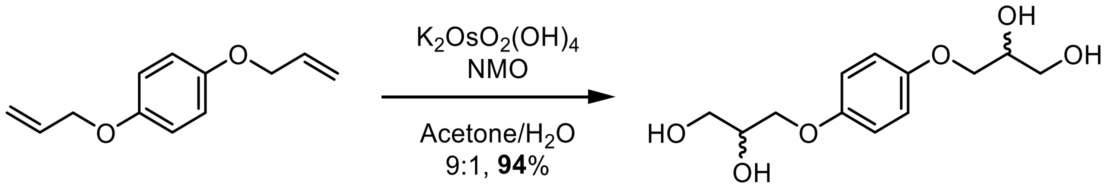

Following the general procedure **C**: 1,4-bis(allyloxy)benzene (**15f**) (0.19 g, 1.0 mmol). The reaction mixture was stirred at 40 ˚C for 16 h. Purification by silica gel chromatography (EtOH/EtOAc, 1:4, R_f = 0.30) afforded the title compound as a white solid (0.24 g, 94%). **M.P** 97 – 99 ˚C; **IR** ν_{max} cm^{-1} 3232 br s (O-H), 2909 s, 2867 s; **^{1}H-NMR** (400 MHz, CD_3OD) δ_H 6.88 (s, 4H, Ar-H), 4.02 – 3.87 (m, 6H, Ar-OCH_2 & CH-OH), 3.65 (qd, *J* = 11.3, 5.2 Hz, 4H, CH_2-OH); **^{13}C-NMR** (101 MHz, CD_3OD) δ_C 154.7 (Ar-O), 116.5 (Ar-H), 71.9 (CH-OH), 71.0 (Ar-OCH_2), 64.2 (CH_2-OH); **LRMS** *m/z* (ESI+) 281.10 (100%, $[M-H_2O_2]^+$); **HRMS** *m/z* (ESI+) $C_{14}H_{18}O_6Na$ Requires: 281.1001, Found: 281.9999 ($[M-H_2O_2]^+$).

(±**16g**) Methyl (±)-2-(2,3-dihydroxypropoxy)benzoate

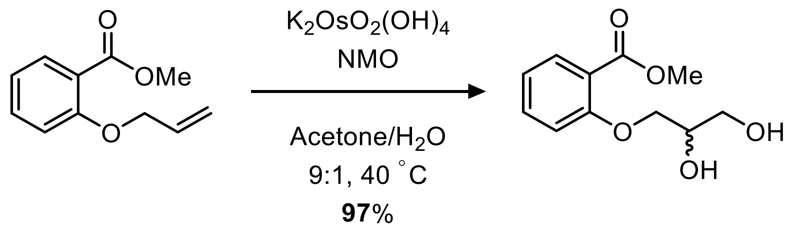

Following general procedure **C**: NMO (140 mg, 1.20 mmol) was charged with acetone/H_2O (9:1, 10 mL) and $K_2OsO_2(OH)_4$ (3.7 mg, 0.01 mmol). Methyl 2-(allyloxy)benzoate (**15g**) (192 mg, 1.00 mmol) was added, the reaction mixture was heated at 40 ˚C for 8 h and monitored (TLC, EtOAc/hexane 1:1, R_f = 0.26). Afforded the title compound as a clear oil (220 mg, 97%). **IR** $\tilde{\nu}_{max}$ cm^{-1} 3387 br (O-H), 2951 w, 1716 s (C=O), 1586 m, 1489 m, 1444 s; **^{1}H-NMR** (400 Hz, $(CD_3)_2SO$) δ_H 7.64 (dd, *J* = 7.5, 1.8 Hz, 1H, C6-H), 7.51 (ddd, *J* = 8.5, 7.5, 1.8 Hz, 1H, C5-H), 7.15 (dd, *J* = 8.5, 1.0 Hz, 1H, C4-H), 7.01 (td, *J* = 7.5, 1.0 Hz, 1H, C3-H), 4.89 (d, *J* = 5.0 Hz, 1H, CH-OH), 4.63 (t, *J* = 5.7 Hz, 1H, CH_2-OH), 3.05 – 3.91 (m, 2H, C2-OCH_2), 3.78 (m, 4H, CH-OH & Me), 3.57 – 3.42 (m, 2H, CH_2-OH); **^{13}C-NMR** (101 MHz, $(CD_3)_2SO$) δ_C 166.3 (CO_2Me), 157.8 (C2), 133.6 (C5), 130.7 (C6), 120.3 (C1), 120.2 (C3), 113.8 (C4), 70.2 (C2-OCH_2), 69.8 (CH-OH), 62.7 (CH_2-OH), 51.9 (Me); **LRMS** (ESI+) 249.07 (30%, $[M+Na]^+$), 226.08 (10%, $[M]^+$), 195.10 (40%, $[M–CH_3OH]^+$), 120.08 (100%, $[C_7H_4O_2]^+$), 152.20 (50%, [**3s**]$^+$); **HRMS** (ESI+) $C_{11}H_{14}O_5Na$ Requires: 249.0739, Found: 249.0741 ($[M+Na]^+$).

(±**16h**) Methyl (±)-3-(2,3-dihydroxypropoxy)benzoate

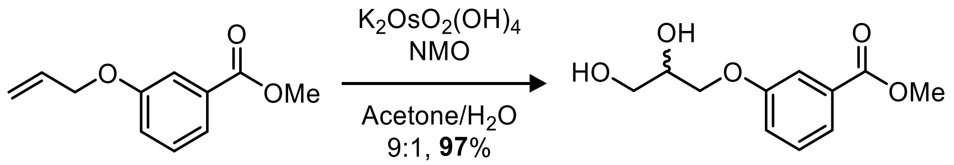

Following general procedure **C**: NMO (140 mg, 1.20 mmol) was charged with acetone/H_2O (9:1, 10 mL) and $K_2OsO_2(OH)_4$ (3.7 mg, 0.01 mmol). Methyl 3-(allyloxy)benzoate (**15h**) (192 mg, 1.00 mmol) was added, the reaction mixture was heated at 40 ˚C for 8 h and monitored (TLC, EtOAc/hexane 1:1, R_f = 0.19). Afforded the title compound as a clear oil (220 mg, 97%). **IR** $\tilde{\nu}_{max}$ cm^{-1} 3408 br (O-H), 2951 w, 1706 s (C=O), 1600 w; **^{1}H-NMR** (400 Hz, $(CD_3)_2SO$) δ_H 7.54 (dt, *J* = 7.7, 1.2 Hz, 1H, C6-H), 7.45 (app. t, *J* = 1.2 Hz, 1H, C2-H), 7.42 (obsv. d, *J* = 7.7 Hz, 1H, C5-H), 7.23 (ddd, *J* = 8.2, 2.7, 1.2 Hz, 1H, C4-H), 5.01 (d, *J* = 5.1 Hz, 1H, CH-OH), 4.72 (t, *J* = 5.7 Hz, 1H, CH_2-OH), 4.05 (dd, *J* = 9.8, 4.0 Hz, 1H, C3-OCH_2), 3.90 (dd, *J* = 9.8, 6.0 Hz, 1H, C3-OCH_2), 3.85 (s, 3H, Me), 3.80 (pd, *J* = 6.0, 4.0 Hz, 1H, CH-OH), 3.52 – 3.42

(m, 2H, C<u>H</u>$_2$-OH); **^{13}C-NMR** (101 MHz, $(CD_3)_2SO$) δ_C 166.1 (<u>C</u>O$_2$Me), 158.9 (C3), 131.0 (C1), 130.0 (C5), 121.4 (C6), 119.9 (C2), 114.4 (C4), 69.9 (C3-O<u>C</u>H$_2$), 69.9 (<u>C</u>H-OH), 62.6 (<u>C</u>H$_2$-OH), 52.3 (Me); **LRMS** (ESI+) 271.10 (90%, [M+2(Na)–H]$^+$), 249.07 (100%, [M+Na]$^+$); **HRMS** (ESI+) $C_{11}H_{14}O_5Na$ Requires: 249.0739, Found: 249.0740 ([M+Na]$^+$).

(±**16i**) Methyl (±)-4-(2,3-dihydroxypropoxy)benzoate

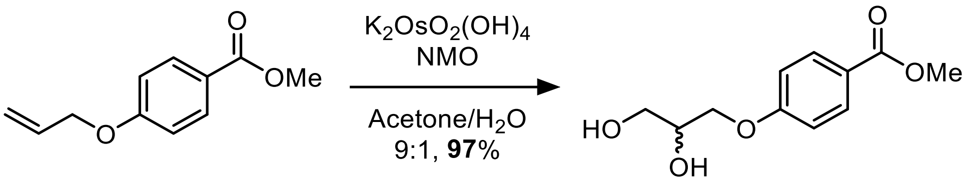

Following general procedure **C**: NMO (140 mg, 1.20 mmol) was charged with acetone/H_2O (9:1, 10 mL) and $K_2OsO_2(OH)_4$ (3.7 mg, 0.01 mmol). Methyl 4-(allyloxy)benzoate (**15i**) (192 mg, 1.00 mmol) was added and the reaction mixture was heated at 40 ˚C for 8 h and monitored (TLC, EtOAc/hexane 1:1, R_f = 0.14). Afforded the title compound as a white solid (220 mg, 97%). **M.P** 68 – 70 ˚C; **IR** V̄max cm^{-1} 3353 br s (O-H), 1957 br s, 2901 br s, 1718 s (C=O), 1605 s, 1509 w; **^{1}H-NMR** (400 MHz, $(CD_3)_2CO$) δ_H 7.98 – 7.92 (m, 2H, C3-<u>H</u>), 7.07 – 7.01 (m, 2H, C2-<u>H</u>), 4.18 (dd, J = 9.6, 4.4 Hz, 1H, C4-OC<u>H</u>$_2$), 4.14 (d, J = 5.1 Hz, 1H, CH-O<u>H</u>), 4.07 (dd, J = 9.6, 6.1 Hz, 1H, C4-OC<u>H</u>$_2$), 4.05 – 3.97 (m, 1H, C<u>H</u>-OH), 3.84 (s, 3H, Me), 3.78 (t, J = 5.8 Hz, 1H, CH$_2$-O<u>H</u>), 3.73 – 3.61 (m, 2H, C<u>H</u>$_2$-OH); **^{13}C-NMR** (101 MHz, $(CD_3)_2CO$) δ_C 166.0 (<u>C</u>O$_2$Me), 163.0 (C4), 131.3 (C3), 122.6 (C1), 114.3 (C2), 70.4 (C4-O<u>C</u>H$_2$), 69.8 (<u>C</u>H-OH), 63.1 (<u>C</u>H$_2$-OH), 51.1 (Me); **LRMS** (ESI+) 249.08 (100%, [M+Na]$^+$), 227.07 (90%, [M+H]$^+$), 186.16 (60%), 179.05 (90%), 177.10 (100%); **HRMS** (ESI+) $C_{11}H_{14}O_5Na$ Requires: 249.0739, Found: 249.0741 ([M+Na]$^+$).

(**17a**) Sodium 3-(2-(2,3-bis(sulfonatooxy)propoxy)-3-(methoxycarbonyl)phenoxy)propane-1,2-diyl bis(sulfate)

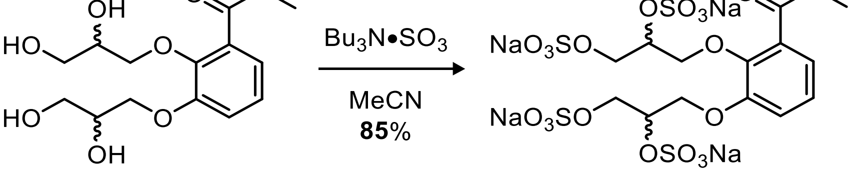

Adapted from general procedure **E**: Methyl 2,3-bis(2,3-dihydroxypropoxy)benzoate (**16a**) (100 mg, 0.33 mmol) and $Bu_3N•SO_3$ (881 mg, 3.31 mmol) were dissolved in MeCN (2 mL) and

heated at 90 ˚C for 24 h. The intermediate $[Bu_3NH]^+$ salt was subjected to ion exchange following procedure **i**, affording the title compound as a white solid (200 mg, 85%). **IR** V̄max cm^{-1} 2953 w, 1711 w, 1583 w, 1480 w, 1207 w; **^{1}H-NMR** (400 MHz, D_2O) δ_H 7.32 (d, *J* = 8.0 Hz, 2H, C4-H̲ & C6-H̲), 7.19 (t, *J* = 8.0 Hz, 1H, C5-H̲), 4.92 (p, *J* = 4.8 Hz, 1H, CH̲-OSO_3Na), 4.85 (p, *J* = 4.8 Hz, 1H, CH̲-OSO_3Na), 4.52 – 4.38 (m, 8H, Ar-OCH̲$_2$ & CH̲$_2$-OSO_3Na), 4.00 (s, 3H, Me); **^{13}C-NMR** (101 MHz, D_2O) δ_C 169.0 (C̲O_2Me), 151.4 (C3), 146.7 (C2), 125.5 (C5), 125.0 (C1), 122.9 (C4), 118.8 (C6), 75.93 (C̲H- OSO_3Na), 75.0 (C̲H- OSO_3Na), 72.2, 67.1, 66.8, 66.7, 53.1 (Me); **LRMS** *m/z* (ESI+) 888.78 (30%), 746.86 (100%, $[M+Na]^+$); **HRMS** *m/z* (ESI+) $C_{14}H_{16}O_{20}Na_5S_4$ Requires: 746.8606, Found: 746.8611 ($[M+Na]^+$).

(**17b**) Sodium 3-(5-(2,3-bis(sulfonatooxy)propoxy)-2-(methoxycarbonyl)phenoxy)propane-1,2-diyl bis(sulfate)

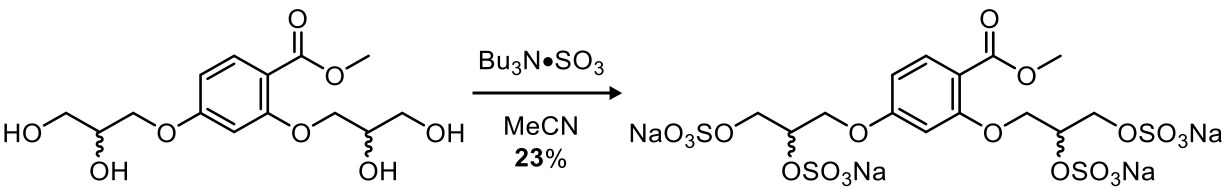

Adapted from general procedure **E**: Methyl 2,4-bis(2,3-dihydroxypropoxy)benzoate (**16b**) (30 mg, 0.09 mmol) and $Bu_3N\cdot SO_3$ (504 mg, 1.90 mmol) were dissolved in MeCN (2 mL) and heated at 90 ˚C for 24 h. The intermediate $[Bu_3NH]^+$ salt was subjected to ion exchange following procedure **i**, affording the title compound as a white solid (16 mg, 23%). **IR** V̄max cm^{-1} 2946 w, 1708 w, 158 w, 1479 w, 1210 w; **^{1}H-NMR** (400 MHz, $(CD_3)_2SO$) δ_H 7.68 (d, *J* = 8.5 Hz, 1H, C6-H̲), 6.72 – 6.52 (m, 2H C5-H̲ & C3-H̲), 4.51 – 4.44 (m, 2H, CH̲- OSO_3Na), 4.28 – 4.02 (m, 4H), 4.06 – 3.89 (m, 3H), 3.85 (dd, *J* = 10.0, 6.7 Hz, 1H), 3.75 (s, 3H, Me); **LRMS** *m/z* (ESI+) 866.81 (30%), 746.86 (50%, $[M+Na]^+$), 644.93 (100%, $[M–SO_3]^+$), 542.98 (90%, $[M–Na_2SO_3]^+$); **HRMS** *m/z* (ESI+) $C_{14}H_{16}O_{20}Na_5S_4$ Requires: 746.8606, Found: 746.8614 ($[M+Na]^+$).

(**17c**) Sodium ((2-(methoxycarbonyl)-1,3-phenylene)bis(oxy))bis(propane-3,1,2-triyl) tetrakis(sulfate)

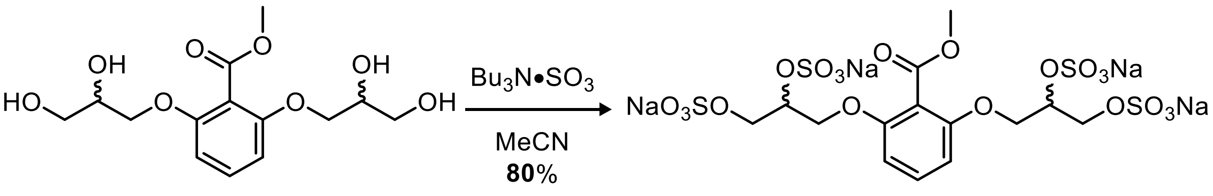

Adapted from general procedure **E**: Methyl 2,6-bis(2,3-dihydroxypropoxy)benzoate (**16c**) (100 mg, 0.32 mmol) and $Bu_3N•SO_3$ (671 mg, 2.53 mmol) were dissolved in MeCN (2 mL) and heated at 90 ˚C for 12 h. The intermediate $[Bu_3NH]^+$ salt was subjected to ion exchange following procedure **i**, affording the title compound as a white solid (185 mg, 80%). **IR** ν_{max} cm^{-1} 2953 w, 1715 w, 1475 w, 1210 w; **^{1}H-NMR** (400 MHz, $(CD_3)_2SO$) δ_H 7.29 (t, *J* = 8.5 Hz, 1H, C4-H), 6.73 (d, *J* = 8.5 Hz, 2H, C3-<u>H</u>), 4.47 – 4.31 (m, 2H, C<u>H</u>-OSO_3Na), 4.17 – 4.01 (m, 4H, C<u>H</u>$_2$-OSO_3Na), 3.95 – 3.78 (m, 4H, C2-OC<u>H</u>$_2$), 3.76 (s, 3H, Me); **^{13}C-NMR** (101 MHz, $(CD_3)_2SO$) δ_C 165.6 (<u>C</u>O_2Me), 156.0 (C2), 131.1 (C4), 113.4 (C1), 105.4 (C3), 72.2 (<u>C</u>H-OSO_3Na), 67.1 (<u>C</u>H_2-OSO_3Na), 64.2 (C2-O<u>C</u>H_2), 51.8 (Me); **LRMS** *m/z* (ESI+) 746.86 (40%, $[M+Na]^+$), 644.93 (60%, $[M–SO_3]^+$), 542.98 (100%, $[M–(SO_3)_2]^+$); **HRMS** *m/z* (ESI+) $C_{14}H_{16}O_{20}Na_5S_4$ Requires: 746.8606, Found: 746.8616 ($[M+Na]^+$).

(**17d**) Sodium 3-(2-(2,3-bis(sulfonatooxy)propoxy)-4-(methoxycarbonyl)phenoxy)propane-1,2-diyl bis(sulfate)

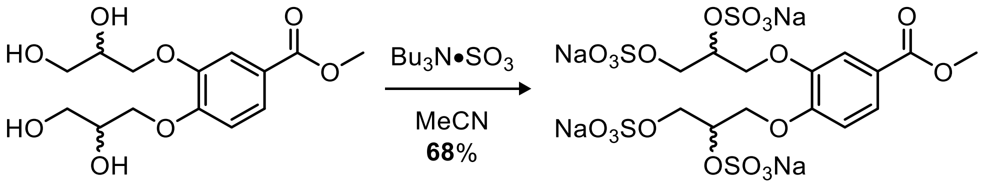

Adapted from general procedure **E**: Methyl 3,4-bis(2,3-dihydroxypropoxy)benzoate (**16d**) (100 mg, 0.32 mmol) and $Bu_3N•SO_3$ (671 mg, 2.53 mmol) were dissolved in MeCN (2 mL) and heated at 90 ˚C for 12 h. The intermediate $[Bu_3NH]^+$ salt was subjected to ion exchange following procedure **i**, affording the title compound as a white solid (157 mg, 68%). **IR** ν_{max} cm^{-1} 2955 w, 1708 w, 1637 w, 1514 w, 1441 w, 1213 s; **^{1}H-NMR** (400 MHz, D_2O) δ_H 7.78 – 7.74 (m, 2H, C2-<u>H</u> & C6-<u>H</u>), 7.23 (d, *J* = 9.1 Hz, 1H, C5-<u>H</u>), 5.09 – 4.88 (m, 2H, C<u>H</u>-OSO_3Na), 4.59 – 4.32 (m, 8H, Ar-OCH_2 & C<u>H</u>-OSO_3Na), 3.94 (s, 3H, Me); **^{13}C-NMR** (101 MHz, D_2O) δ_C 168.8 (<u>C</u>O_2Me), 152.7 (C3), 147.4 (C4), 125.1 (C6), 123.0 (C1), 116.6 (C2), 114.0 (C5), 75.1 (<u>C</u>H-OSO_3Na), 75.0 (<u>C</u>H-OSO_3Na), 74.9 (<u>C</u>H-OSO_3Na), 74.8 (<u>C</u>H-OSO_3Na), 68.04, 67.99, 67.1, 66.6, 52.6 (Me); **LRMS** *m/z* (ESI+) 746.86 (100%, $[M+Na]^+$); **HRMS** *m/z* (ESI+) $C_{14}H_{16}O_{20}Na_5S_4$ Requires: 746.8606, Found: 746.8603 ($[M+Na]^+$).

(**17e**) Sodium ((5-(methoxycarbonyl)-1,3-phenylene)bis(oxy))bis(propane-3,1,2-triyl) tetrakis(sulfate)

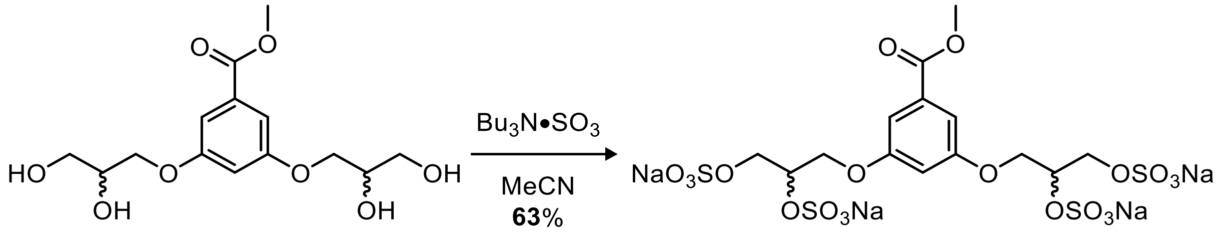

Adapted from general procedure **E**: Methyl 3,5-bis(2,3-dihydroxypropoxy)benzoate (**16e**) (50 mg, 0.16 mmol) and Bu_3N•SO_3 (336 mg, 1.26 mmol) were dissolved in MeCN (2 mL) and heated at 90 ˚C for 12 h. The intermediate $[Bu_3NH]^+$ salt was subjected to ion exchange following procedure **i**, affording the title compound as a white solid (72 mg, 63%). **IR** V̄max cm^{-1} 1710 w, 1602 w, 1448 w, 1353 w, 1236 s; **^{1}H-NMR** (400 MHz, D_2O) δ_H 7.35 (d, *J* = 2.3 Hz, 2H, C2-H), 7.00 (t, *J* = 2.3 Hz, 1H, C4-H), 4.94 (p, *J* = 4.7 Hz, 2H, CH-OSO_3Na), 4.49 – 4.33 (m, 8H, C3-OCH_2 & CH_2-OSO_3Na), 3.96 (s, 3H, Me); **^{13}C-NMR** (101 MHz, D_2O) δ_C 168.6 (CO_2Me), 159.2 (C3), 131.7 (C1), 109.0 (C2), 107.6 (C4), 75.0 (CH-OSO_3Na), 66.8, 66.5, 52.8 (Me); **LRMS** *m/z* (ESI+) 644.93 (100%, $[M–SO_3]^+$).

(**17f**) Sodium (1,4-phenylenebis(oxy))bis(propane-3,1,2-triyl) tetrakis(sulfate)

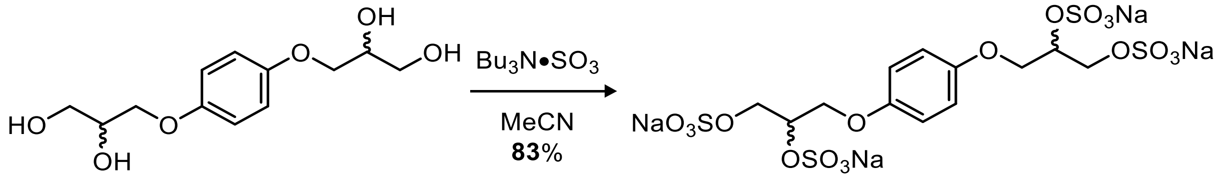

Adapted from general procedure **E**: 1,4-Phenylenebis(oxy))bis(propane-1,2-diol (**16f**) (50 mg, 0.19 mmol) and Bu_3N•SO_3 (257 mg, 0.97 mmol) were dissolved in MeCN (1 mL) and heated at 90 ˚C for 5 h. The intermediate $[Bu_3NH]^+$ salt was subjected to Ion exchange following procedure **i**, affording the title compound as a white solid (91 mg, 83%). **IR** V̄max cm^{-1} 2947 w, 1634 w, 1509 s, 1459 w, 1208 s; **^{1}H-NMR** (400 MHz, D_2O) δ_H 7.02 (s, 4H, Ar-H), 4.84 (p, *J* = 4.8 Hz, 2H, CH-OSO_3Na), 4.42 – 4.28 (m, 6H ArO-CH_2 & CH_2-OSO_3Na), 4.23 (m, 2H, ArO-CH_2); **^{13}C-NMR** (101 MHz, D_2O) δ_C 152.7 (Ar-O), 116.4 (Ar-H), 75.1 (CH-OSO_3Na), 67.3 (ArO-CH_2), 66.5 (CH_2-OSO_3Na); **LRMS** *m/z* (ESI–) 642.88 (50%, $[M–Na]^-$), 540.94 (50% $[M–Na_2SO_3]^-$), 529.46 (100%); **HRMS** *m/z* (ESI–) $C_{12}H_{14}O_{18}Na_3S_4$ Requires: 642.8756, Found: 642.8760 ($[M–Na]^-$).

(**17g**) Sodium 3-(2-(methoxycarbonyl)phenoxy)propane-1,2-diyl bis(sulfate)

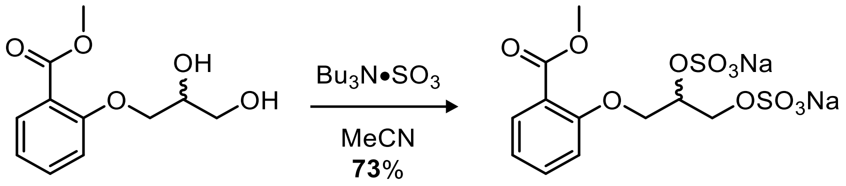

Adapted from general procedure **E**: Methyl (±)-2-(2,3-dihydroxypropoxy)benzoate (**±16g**) (226 mg, 1.0 mmol) and $Bu_3N{\bullet}SO_3$ (1.60 g, 4.0 mmol) were dissolved in MeCN (4 mL) and heated at 90 ˚C for 8 h. The intermediate $[Bu_3NH]^+$ salt was subjected to ion exchange following procedure **i**, affording the title compound as a white solid (300 mg, 70%). **IR** Vmax cm^{-1} 1709 w, 1602 w, 1441 w, 1349 w, 1214 s; **^{1}H-NMR** (400 MHz, $(CD_3)_2SO$) δ_H 7.65 (dd, *J* = 7.5, 1.9 Hz, 1H, C6-H), 7.51 (ddd, *J* = 8.6, 7.5, 1.9 Hz, 1H, C5-H), 7.15 (d, *J* = 8.6 Hz, 1H, C4-H), 7.01 (t, *J* = 7.5 Hz, 1H, C3-H), 4.48 (p, *J* = 5.3 Hz, 1H, CH-OSO_3Na), 4.22 (dd, *J* = 9.9, 5.0 Hz, 1H, C2-OCH_2), 4.11 (dd, *J* = 9.9, 3.5 Hz, 1H, C2-OCH_2), 4.03 – 3.88 (m, 2H, CH_2-OSO_3Na), 3.80 (s, 3H, Me); **^{13}C-NMR** (101 MHz, $(CD_3)_2SO$) δ_C 166.5 (CO_2Me), 157.6 (C2), 133.6 (C5), 130.8 (C6), 120.3 (C1), 120.2 (C3), 113.6 (C4), 72.3 (CH-OSO_3Na), 67.1 (C2-OCH_2), 56.0 (CH_2-OSO_3Na), 51.8 (Me); **LRMS** *m/z* (ESI+) 452.95 (90%, $[M+Na]^+$), 351. 01 (100%, $[M–SO_3]^+$); **HRMS** *m/z* (ESI+) $C_{11}H_{12}S_2O_{11}Na_3$ Requires: 452.9514, Found: 452.9516 ($[M+Na]^+$).

(**17h**) Sodium 3-(3-(methoxycarbonyl)phenoxy)propane-1,2-diyl bis(sulfate)

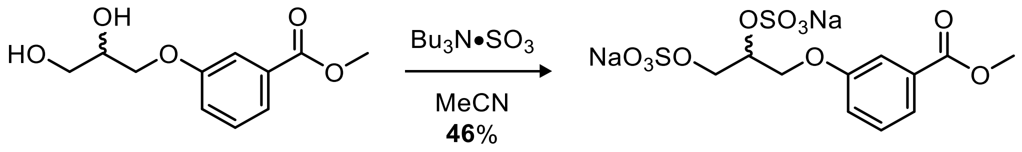

Adapted from general procedure **E**: Methyl (±)-3-(2,3-dihydroxypropoxy)benzoate (**±16h**) (226 mg, 1.0 mmol) and $Bu_3N{\bullet}SO_3$ (1.60 g, 4.0 mmol) were dissolved in MeCN (4.0 mL) and heated at 90 ˚C for 8 h. The intermediate $[Bu_3NH]^+$ salt was subjected to ion exchange following procedure **i**, affording the title compound as a white solid (197 mg, 46%). **IR** Vmax cm^{-1}1704 w, 1606 w, 1440 w, 1353 w, 1215 s; **^{1}H-NMR** (400 MHz, $(CD_3)_2SO$) δ_H 7.54 (dt, *J* = 8.2, 1.1 Hz, 1H, C4-H), 7.48 – 7.38 (m, 2H, C5-H & C2-H), 7.25 (ddd, *J* = 8.2, 2.8, 1.1 Hz, 1H, C6-H), 4.47 (dq, *J* = 6.8, 4.4 Hz, 1H, CH-OSO_3Na), 4.22 – 4.05 (m, 2H, C3-OCH_2), 3.98 (dd, *J* = 10.0, 5.0 Hz, 1H, CH_2-OSO_3Na), 3.91 – 3.81 (m, 4H, Me & CH_2-OSO_3Na); **^{13}C-NMR** (101 MHz, $(CD_3)_2SO$) δ_C 166.0 (CO_2Me), 158.7 (C3), 130.9 (C1), 129.9 (C5), 121.4 (C4), 119.6 (C6), 114.6 (C2), 72.1 (CH-OSO_3Na), 66.7 (C3-OCH_2), 63.9 (CH_2-OSO_3Na), 52.2 (Me); **LRMS** *m/z*

(ESI+) 452.95 (70%, $[M+Na]^+$), 351. 01 (100%, $[M–SO_3]^+$), 249.07 (50%, $[M–(SO_3)_2]^+$); **HRMS** *m/z* (ESI+) $C_{11}H_{12}S_2O_{11}Na_3$ Requires: 452.9514, Found: 452.9529 ($[M+Na]^+$).

(**17i**) Sodium 4-(3-(methoxycarbonyl)phenoxy)propane-1,2-diyl bis(sulfate)

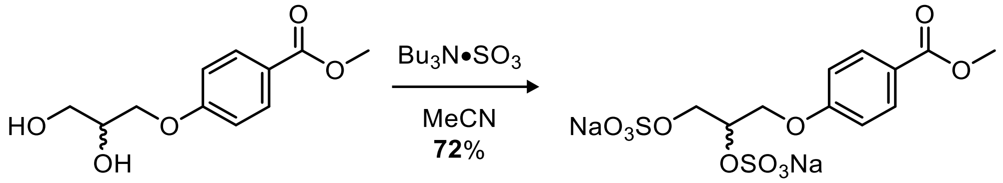

Adapted from general procedure **E**: Methyl (±)-3-(2,3-dihydroxypropoxy)benzoate (**±16i**) (226 mg, 1.0 mmol) and $Bu_3N•SO_3$ (1.60 g, 4.0 mmol) were dissolved in MeCN (4 mL) and heated at 90 ˚C for 8 h. The intermediate $[Bu_3NH]^+$ salt was subjected to ion exchange following procedure **i**, affording the title compound as a white solid (310 mg, 72%). **IR** Vmax cm^{-1} 1701 w, 1602 w, 1442 w, 1348 w, 1210 s; **^{1}H-NMR** (400 MHz, D_2O) δ_H 8.00 – 7.91 (m, 2H, C2-H), 7.12 – 7.04 (m, 2H, C3-H), 4.95 – 4.86 (m, 1H, CH-OSO_3Na), 4.48 – 4.31 (m, 4H, C4-OCH_2 & CH_2-OSO_3Na), 3.88 (s, 3H, Me); **^{13}C-NMR** (101 MHz, D_2O) δ_C 169.1 (CO_2Me), 162.2 (C4), 131.6 (C2), 122.3 (C1), 114.7 (C3), 74.8 (CH-OSO_3Na), 66.4 (CH_2), 66.3 (CH_2), 52.4 (Me); **LRMS** *m/z* (ESI+) 452.90 (100%, $[M+Na]^+$); **HRMS** *m/z* (ESI+) $C_{11}H_{12}S_2O_{11}Na_3$ Requires: 452.9515, Found: 452.9514 ($[M+Na]^+$).

(**20**) Methyl 2,5-hydroxybenzoate

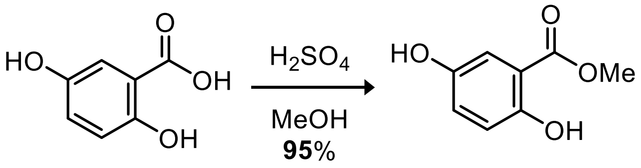

Adapted from general procedure **A**: 2,5-dihydroxybenzoic acid, **S1** (15.40 g, 0.10 mol) in MeOH (200 mL) was charged with conc. H_2SO_4 (2 mL) and heated under reflux for 21 h with monitoring (EtOAc/hexane 1:3, R_f = 0.41). A yellow solid was afforded after work up, recrystallization (CH_2Cl_2/hexane) yielded the title compound as long yellow crystals (15.92 g, 95%). **M.P** 87 – 90 ˚C; **IR** Vmax cm^{-1} 3331m (O-H), 2960 w, 1683 s (C=O); **^{1}H-NMR** (400 MHz, $CDCl_3$) δ_H 10.34 (s, 1H, C2-OH), 7.28 (d, J = 3.1 Hz, 1H, C6-H), 7.01 (dd, *J* = 8.9, 3.1 Hz, 1H, C4-H), 6.89 (d, *J* = 8.9 Hz, 1H, C3-H), 4.66 (s, 1H, C5-OH), 3.94 (s, 3H, Me); **^{13}C-NMR** (101 MHz, $CDCl_3$) δ_C 170.2 (CO_2Me), 156.0 (C2), 147.8 (C5), 124.2 (C4), 118.7 (C3),

114.9 (C6), 112.3 (C1), 52.5 (Me); **LRMS** *m/z* (ESI–) 167.03 (100%, $[M–H]^-$), 135.01 (30%, $[M-CH_3OH_2]^-$); **HRMS** *m/z* (ESI–) $C_8H_7O_4$ Requires: 167.0524, Found: 167.0525 ($[M–H]^-$).

(**22**) Methyl 3,6-dihydroxy-2-nitrobenzoate[S25]

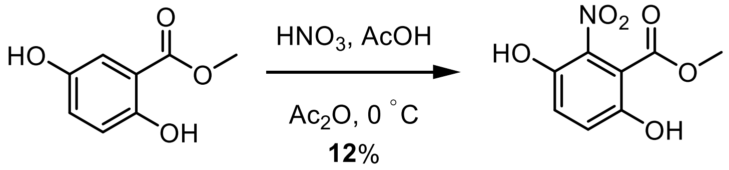

A cooled solution of HNO_3 (1.1 mL, 25.8 mmol) in AcOH (6.8 mL) was added dropwise over 5 min to a stirred solution of 2,5-dihydroxybenzoate (**20**) (2.0 g, 11.8 mmol) in AcOH (14.4 mL) and Ac_2O (7.41 mL) at 0 ˚C. The reaction was warmed to room temperature, stirred for 30 min and neutralised with $NaHCO_3$ $_{(aq.)}$ (250 mL). The resulting slurry was then extracted with ether (3 × 150 mL) dried ($MgSO_4$) and the solvent removed under reduced pressure to afford an orange oil. The crude oil was purified by column chromatography (SiO_2, 1:9 EtOAc/hexane, R_f = 0.08) to give an orange solid. Recrystallization (CH_2Cl_2/hexane) gave the title compound as dark orange crystals (300 mg, 12%). **M.P** 125 – 127 ˚C; **IR** $\tilde{\nu}_{max}$ cm^{-1} 3251 br, 2961 w, 1685 s, 1628 w, 1592 w, 1538 s, 1442 s; **^{1}H-NMR** (400 MHz, $CDCl_3$) δ_H 8.89 (d, 2H, OH), 7.21 (s, 2H, C3-H, C4-H), 3.90 (s, 3H, Me); **^{13}C-NMR** (101 MHz, $CDCl_3$) δ_C 167.5 (CO_2Me), 153.6 (C6), 146.7 (C3), 126.2 (C4), 125.4 (C5), 107.7 (C1), 53.5 (Me), C2 gave no resonance signal in ^{13}C-NMR, ^{1}H-^{13}C HSQC displayed coupling between C4 and C2; **LRMS** *m/z* (ESI+) 183.09 (100%, $[M–MeOH]^+$), 213.72 (10%, $[M]^+$). Data were in accordance with the literature.[S25]

(**23**) Methyl 3,6-bis(allyloxy)-2-nitrobenzoate

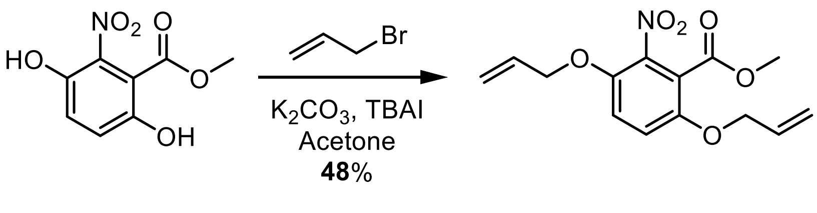

A flask containing methyl 3,6-dihydroxy-2-nitrobenzoate (**22**) (300 mg, 1.41 mmol), K_2CO_3 (428 mg, 3.1 mmol) and TBAI (284 mg, 0.77 mmol) was charged with acetone (10 mL) and the mixture was stirred at room temperature for 10 min. Allyl bromide (0.27 mL, 3.1 mmol) was added dropwise over 5 min and the reaction mixture was heated under reflux for 4 h. The flask was cooled, the solvent removed under reduced pressure and the flask was charged with H_2O

(10 mL). The aqueous mixture was extracted with EtOAc (3 × 30 mL), the combined organic extracts were washed with brine (20 mL) and dried ($MgSO_4$). Removal of the solvent under reduced pressure afforded a crude oil that was purified by chromatography (SiO_2, EtOAc/hexane 1:9, R_f = 0.33) to give the title compound as a yellow oil (200 mg, 48%). **IR** ν_{max} cm^{-1} 2955 w, 1731 s, 1649 w, 1578 w, 1537 s, 1485 s, 1442 s, 1423 s, 1363 s; **^{1}H-NMR** (400 MHz, $CDCl_3$) δ_H 7.12 – 6.98 (m, 2H, C3-H & C4-H), 6.05 – 5.89 (m, 2H, CH=CH_2), 5.44 – 5.34 (m, 2H, CH=CH_2), 5.29 (ddq, *J* = 10.6, 3.0, 1.4 Hz, 2H, CH=CH_2), 4.58 (ddt, *J* = 9.4, 5.0, 1.6 Hz, 4H, O-CH_2), 3.87 (s, 3H, Me); **^{13}C-NMR** (101 MHz, $CDCl_3$) δ_C 163.5 (CO_2Me), 150.0 (C6), 144.6 (C3), 140.2 (C2, weak signal), 132.3 (CH=CH_2), 131.9 (CH=CH_2), 119.0 (C1), 118.7 (CH=CH_2), 118.2 (CH=CH_2), 117.8 (C4), 117.6 (C5), 71.2 (O-CH_2), 70.9 (O-CH_2), 53.3 (Me); **LRMS** *m/z* (ESI+) 316.08 (100%, $[M+Na]^+$); **HRMS** *m/z* (ESI+) $C_{14}H_{15}NO_6Na$ Requires: 316.0792, Found: 316.0795 ($[M+Na]^+$).

(**24**) Methyl 3,6-bis(2,3-dihydroxypropoxy)-2-nitrobenzoate

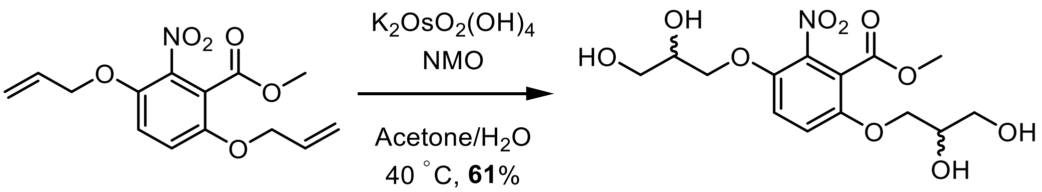

A flask containing methyl 3,6-bis(allyloxy)-2-nitrobenzoate (**23**) (188 mg, 0.64 mmol), was charged with NMO (208 mg, 1.54 mmol) and acetone/H_2O (9:1, 10 mL), the mixture was stirred at room temperature for 10 min. $K_2OsO_2(OH)_4$ (1 mg, 0.0027 mmol) was added and the reaction mixture was heated at 40 ˚C for 12 h. The flask was cooled, satd. Na_2SO_3 $_{(aq.)}$ (10 mL) was added and the aqueous mixture was washed with EtOAc (2 × 20 mL). The flask was placed in liq. N_2 for 10 min and the water was removed by freeze drying for 12 h to give a light yellow solid. The solid was suspended in EtOH (50 mL) and placed in an ultrasonic bath for 10 min. The mixture was filtered through celite and the filtrate was kept, dried ($MgSO_4$) and filtered again. Removal of the solvent under reduced pressure afforded the title compound as a yellow oil (140 mg, 61%). **IR** ν_{max} cm^{-1} 3340 br s, 2952 w, 2889 w, 1724 s, 1618 w, 1577 w, 1535 s, 1488 s, 1441 s, 1362 s; **^{1}H-NMR** (400 MHz, $(CD_3)_2SO$) δ_H 7.56 – 7.33 (m, 2H, C4-H, C5-H), 4.99 (d, *J* = 5.1 Hz, 1H, CH-OH), 4.94 (d, *J* = 5.1 Hz, 1H, CH-OH), 4.67 (dt, *J* = 12.6, 5.7 Hz, 2H, CH_2-OH), 4.16 – 3.92 (m, 4H, Ar-OCH_2), 3.78 (s, 3H, Me), 3.77 – 3.67 (m, 2H, CH-OH), 3.47 – 3.35 (m, 4H, CH_2-OH); **^{13}C-NMR** (101 MHz, $(CD_3)_2SO$) δ_C 163.3 (CO_2Me), 149.6 (C6), 144.3 (C3), 138.6 (C2), 118.7 (C4), 118.5 (C5), 117.2 (C1), 71.7 (Ar-OCH_2), 71.6 (Ar-OCH_2), 69.8 (CH-OH), 69.7 (CH-OH), 62.4 (CH_2-OH), 62.3 (CH_2-OH), 53.0 (Me); **LRMS**

m/z (ESI+) 384.09 (60%, [M+Na]$^+$), 413.26 (100%); **HRMS** *m/z* (ESI+) $C_{14}H_{19}NO_{10}Na$ Requires: 384.0901, Found: 384.0902 ([M+Na]$^+$).

(**25**) Methyl 2-amino-3,6-bis(2,3-dihydroxypropoxy)benzoate

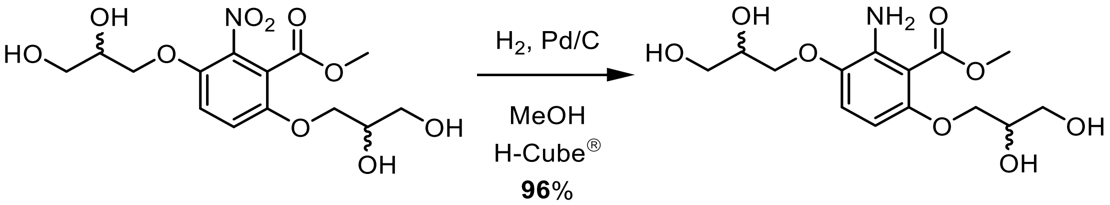

A solution of methyl 3,6-bis(2,3-dihydroxypropoxy)-2-nitrobenzoate (**24**) (50 mg, 0.14 mmol) in MeOH (20 mL) was placed (1 mL min^{-1}) through a Thales Nano H-Cube mini® using a Pd/C cartridge at 30 ˚C (10 – 15 bar H_2). The solution was recirculated for 80 min with monitoring (TLC, EtOAc/MeOH, 9:1, R_f = 0.45). The solvent was removed under reduced pressure and the crude oil was purified by chromatography (SiO_2, EtOAc/MeOH, 9:1) to afford the title compound as a light brown oil (44 mg, 96%). **IR** Vmax cm^{-1} 3453 s, 3343 s, 3244 br s, 2929 s, 2878 s, 1683 s, 1615 s, 1591 s, 1566 s, 1480 s, 1447 s, 1437 s; **^{1}H-NMR** (400 MHz, $(CD_3)_2SO$) δ_H 6.80 (obs. d, *J* = 8.8 Hz, 1H, C4-H), 6.12 (obs. d, *J* = 8.8 Hz, 1H, C5-H), 5.53 (br s, 2H, C2-NH_2), 5.06 (d, *J* = 5.1 Hz, 1H, CH-OH), 4.78 (d, *J* = 5.1 Hz, 1H, CH-OH), 4.67 (t, *J* = 5.7 Hz, 1H, CH_2-OH), 4.56 (t, *J* = 5.7 Hz, 1H, CH_2-OH), 3.97 – 3.88 (m, 1H, CH-OH), 3.86 – 3.67 (m, 8H, Me, Ar-OCH_2 & CH-OH), 3.51 – 3.36 (m, 4H, CH_2-OH); **^{13}C-NMR** (101 MHz, $(CD_3)_2SO$) δ_C 167.7 (CO_2Me), 152.2 (C2), 140.3 (C3), 139.5 (C6), 114.4 (C4), 105.2 (C1), 98.8 (C5), 71.1 (CH-OH), 70.4 (CH-OH), 70.0 (2C, Ar-OCH_2), 62.8 (CH_2-OH), 62.5 (CH_2-OH), 51.6 (Me); **LRMS** *m/z* (ESI+) 354.10 (100%, [M+Na]$^+$); **HRMS** *m/z* (ESI+) $C_{14}H_{21}NO_8Na$ Requires: 354.1159, Found: 354.1162 ([M+Na]$^+$).

(**26**) Sodium 3-(4-(2,3-bis(sulfonatooxy)propoxy)-2-(methoxycarbonyl)-3-(sulfonatoamino)phenoxy)propane-1,2-diyl bis(sulfate)

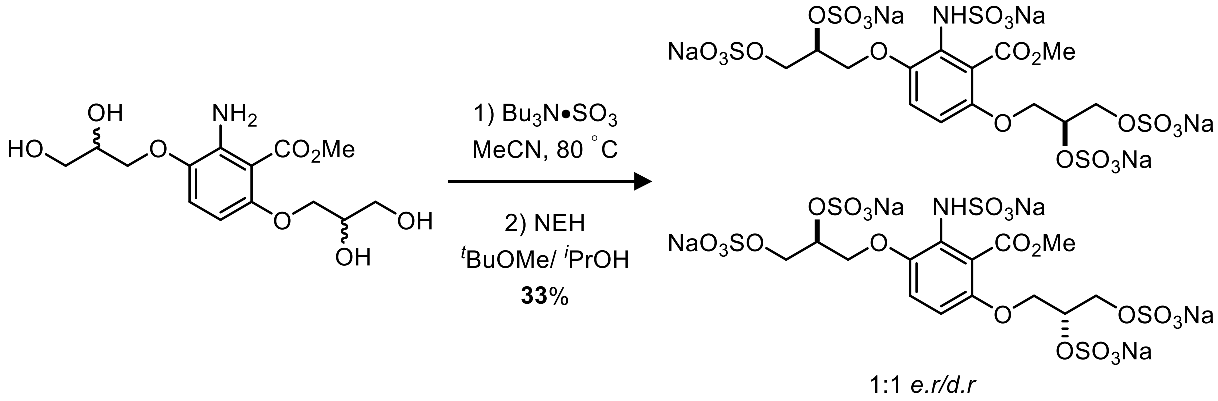

A flask containing methyl 4-amino-2,5-bis(2,3-dihydroxypropoxy)benzoate (**25**) (12 mg, 0.036 mmol) was charged with $Bu_3N•SO_3$ (96 mg, 0.36 mmol) and sealed with Ar. MeCN (1 mL) was added and the reaction mixture was heated at 80 ˚C for 12 h. The flask was cooled and the solvent removed under reduced pressure to give an oil. The crude oil was placed on a silica plug and eluted with EtOAc/MeOH (9:1) to give a clear oil. The oil was dissolved in ^{i}PrOH (1 mL) and transferred to a flask containing ^{t}BuOMe (7 mL). With vigorous stirring a solution of NEH (1.0 mL, 1.0 M) in ^{t}BuOMe/^{i}PrOH (1:7) was added dropwise over 2 min and the reaction mixture was stirred at room temperature for 72 h. The precipitate was collected, washed with ^{i}PrOH (2 × 10 mL) and dried under vacuum. Recrystallization from H_2O/^{i}PrOH afforded the title compound as a white solid (10 mg, 33%). **IR** V̄max cm^{-1} 3472 br s, 2492 w, 1622 br s, 1437 s, 1356 s, 1220 s; **^{1}H-NMR** (500 MHz, D_2O) δ_H 7.40 (s, 2H), 7.09 (obs. d, *J* = 8.8 Hz, 1H), 6.50 (obs. d, *J* = 8.9 Hz, 1H), 5.03 – 4.82 (m, 4H), 4.60 – 4.13 (m, 16H), 4.03 (s, 3H), 3.96 (s, 3H); **^{13}C-NMR** (126 MHz, D_2O) δ_C 168.2, 163.1, 149.2, 141.6, 141.2, 138.7, 124.0, 116.7, 113.8, 102.6, 75.7, 75.3, 75.2, 75.0, 73.8, 68.2, 67.9, 67.8, 67.5, 66.7, 66.6, 66.5, 53.3, 52.7; **LRMS** *m/z* (ESI+) 657.14 (100%), 863.81 (5%, $[M+Na]^+$); **HRMS** *m/z* (ESI+) $C_{14}H_{16}NNa_6O_{23}S_5$ Requires: 863.8097, Found: 863.8114.

Copies of ^{1}H and ^{13}C NMR spectra

^{1}H NMR spectrum of **1** ($CDCl_3$, 400 MHz)

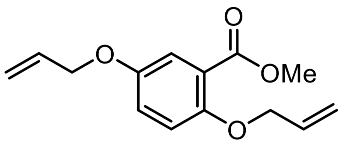

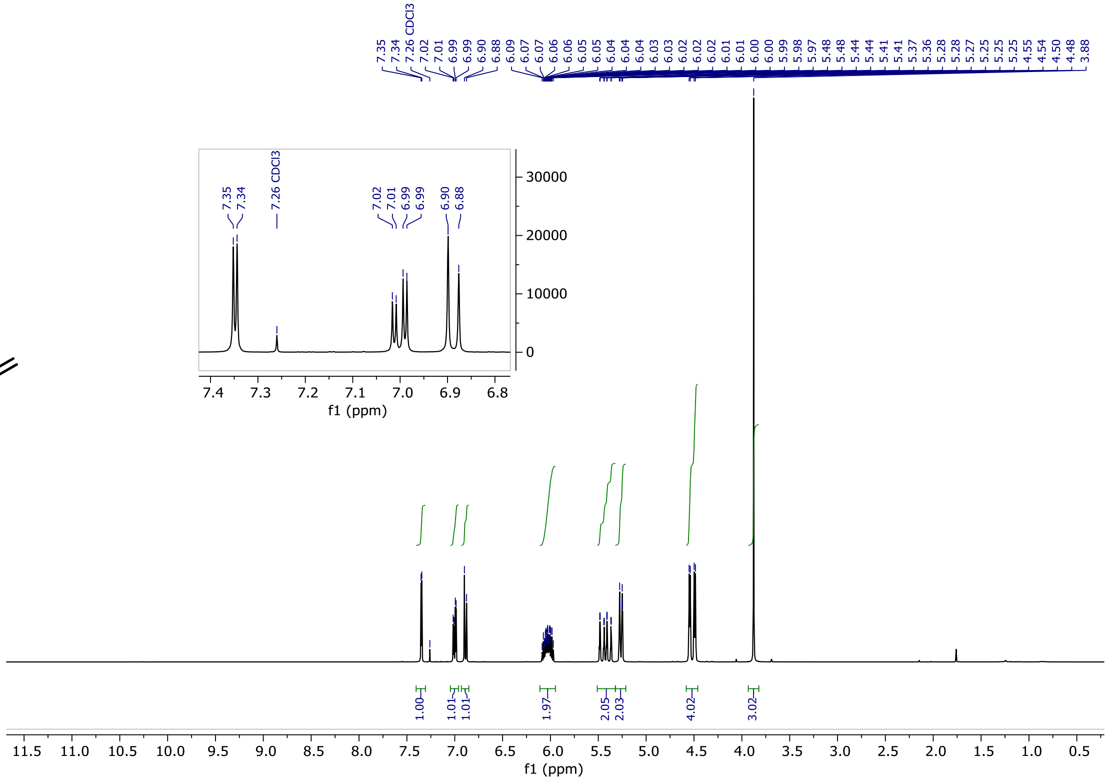

^{13}C NMR spectrum of **1** ($CDCl_3$, 101 MHz)

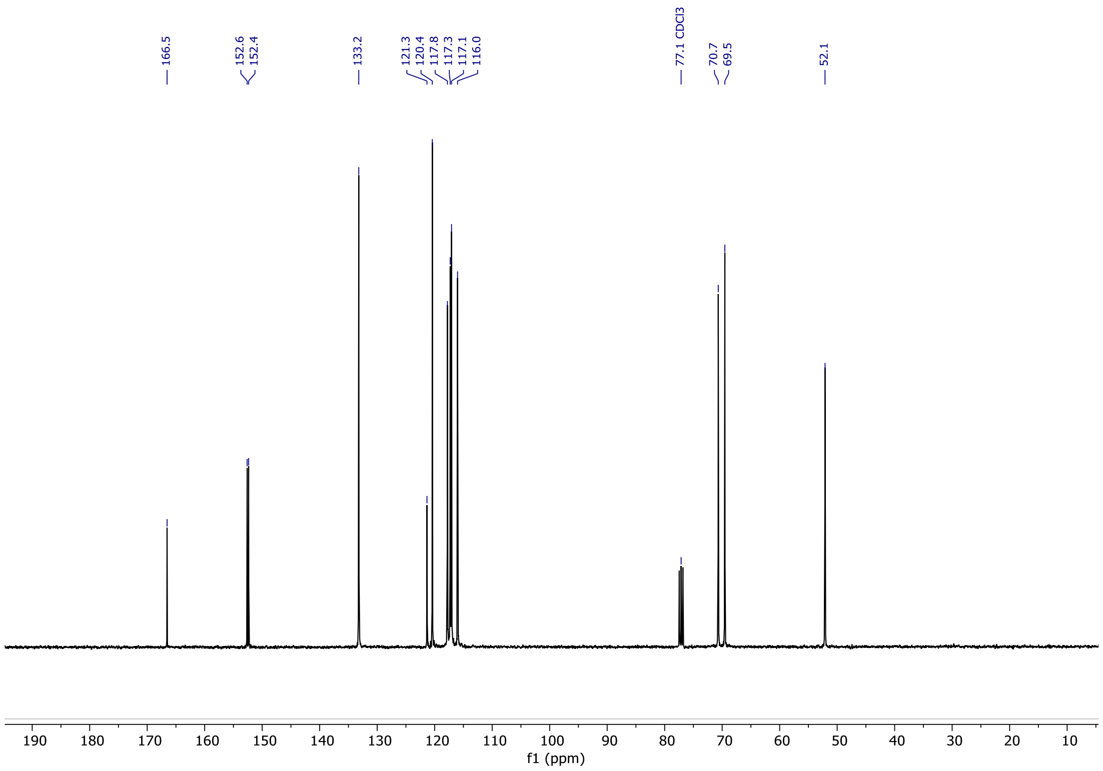

^{1}H NMR spectrum of α-**2** ((CD_3)$_2$SO, 400 MHz)

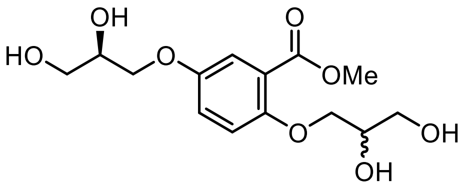

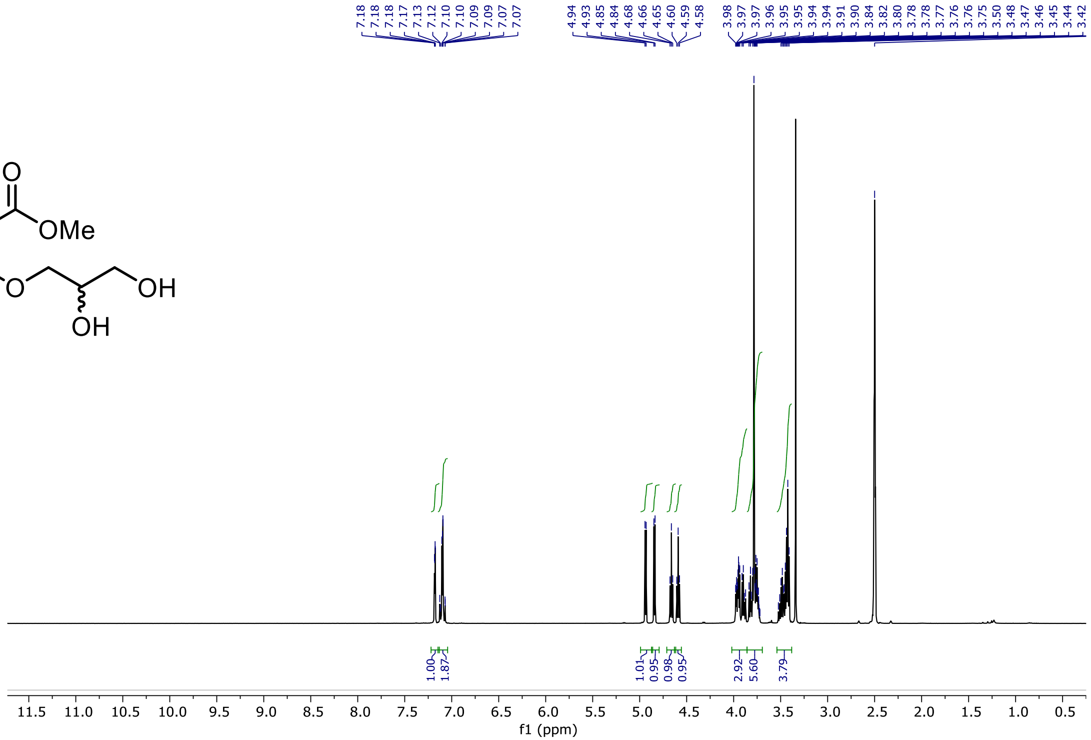

^{13}C NMR spectrum of α-**2** ($(CD_3)_2SO$, 101 MHz)

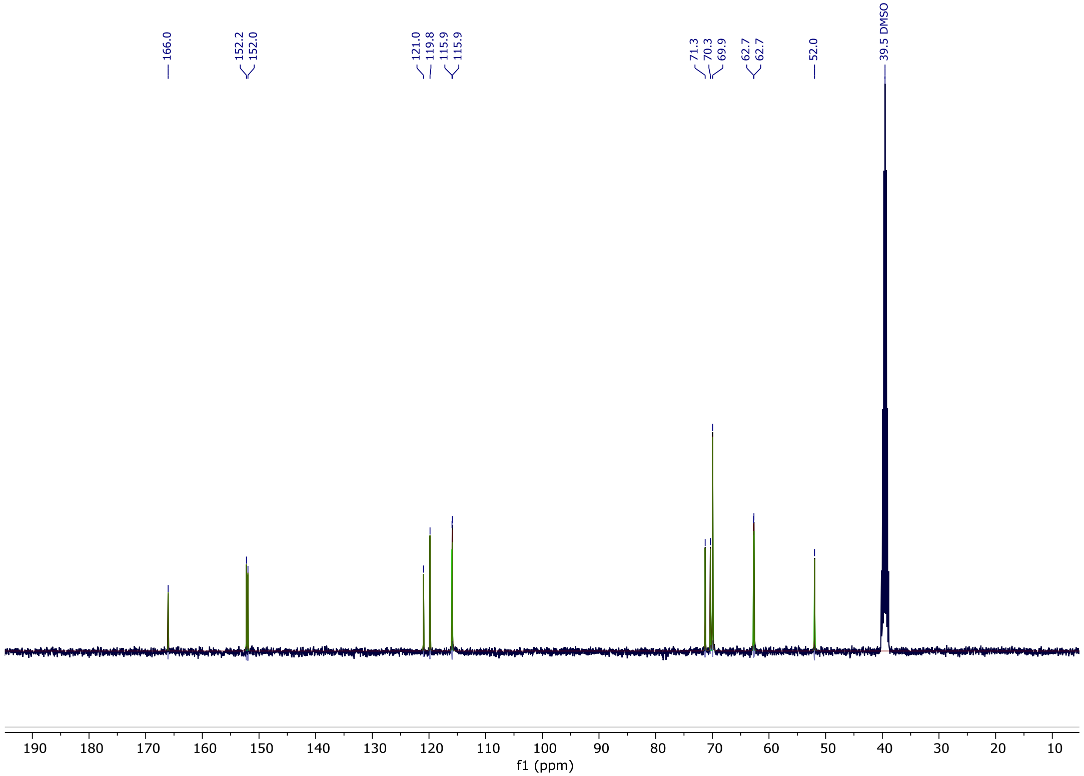

^{1}H NMR spectrum of β-**2** ($(CD_3)_2SO$, 400 MHz)

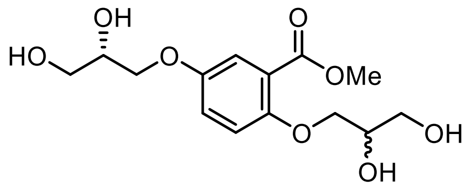

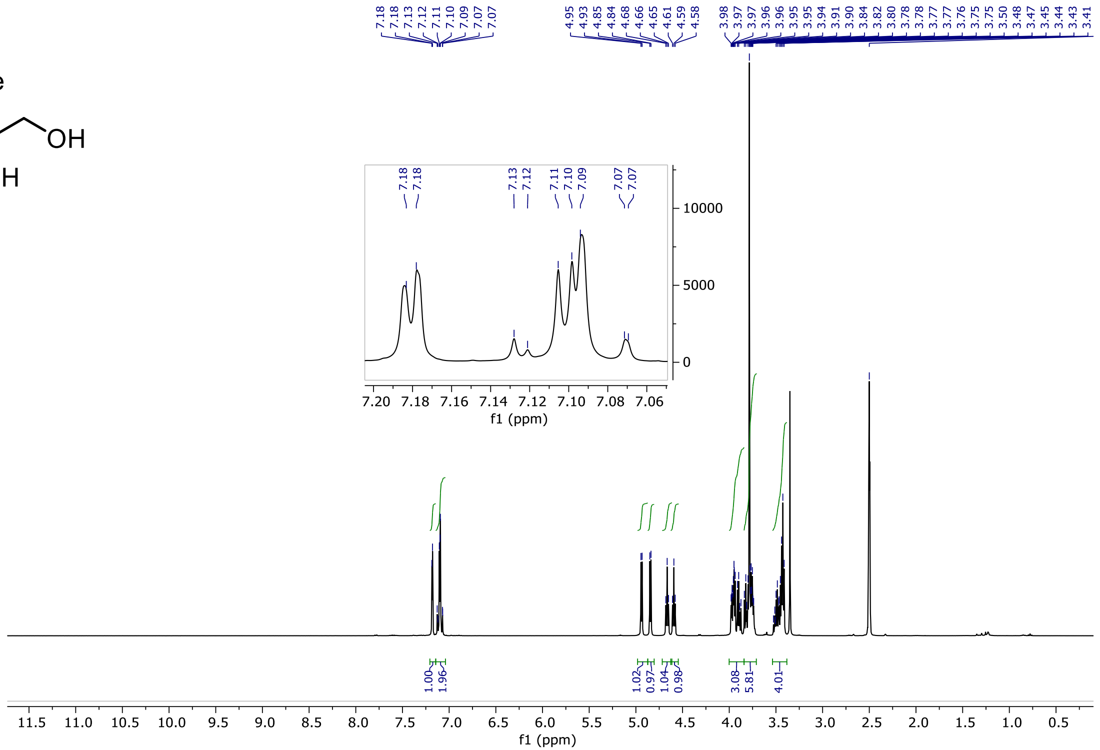

^{13}C NMR spectrum of β-**2** (($(CD_3)_2SO$, 101 MHz)

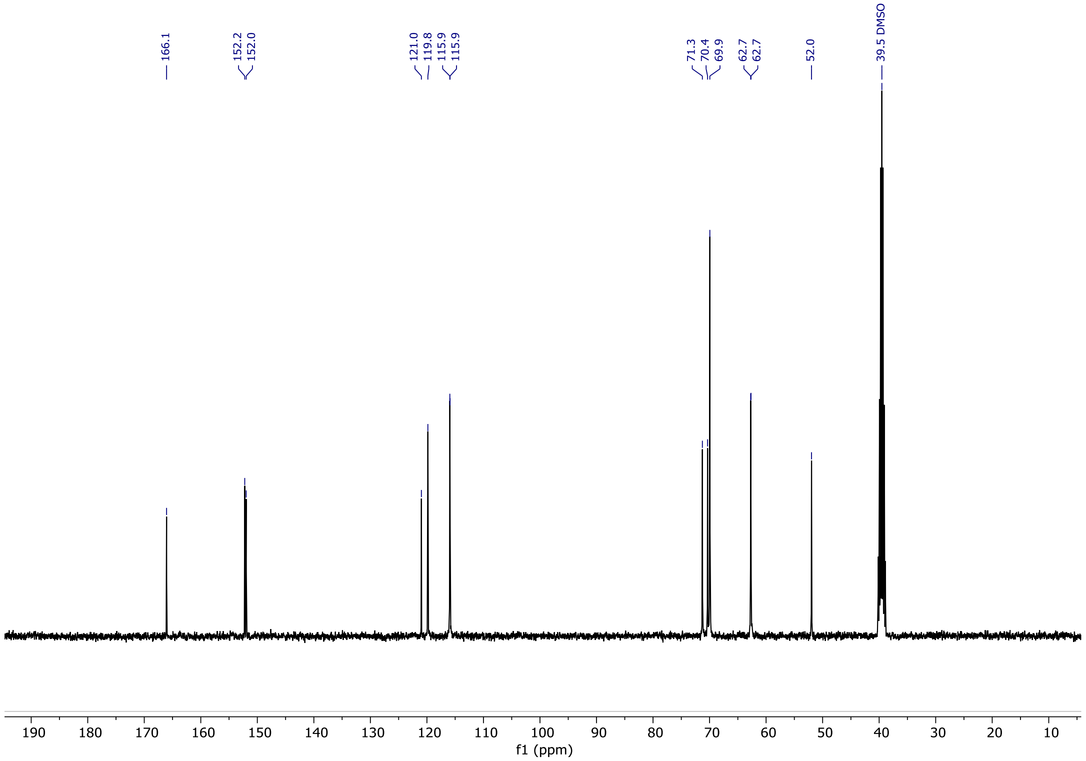

^{1}H NMR spectrum of **α-3** (CD_3OD, 400 MHz)

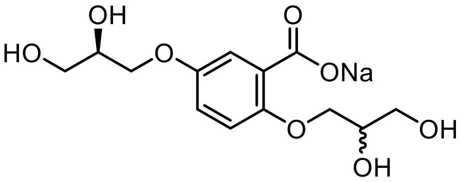

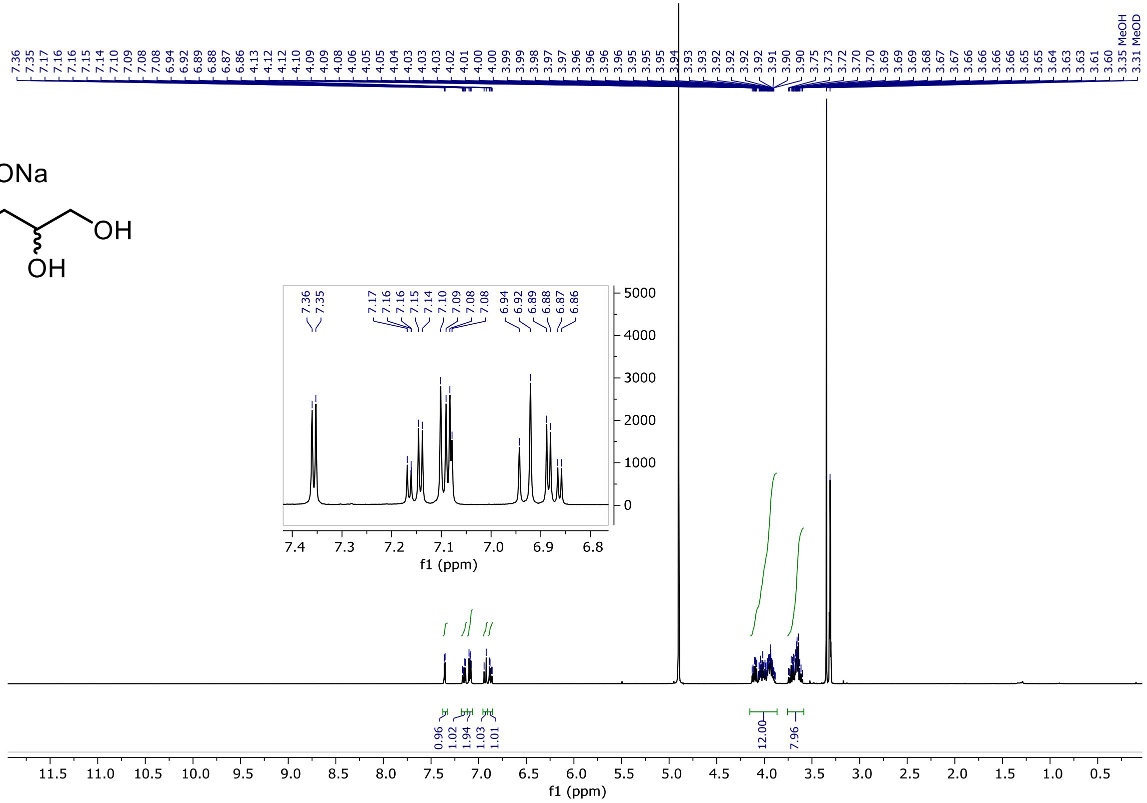

^{13}C NMR spectrum of **α-3** (CD_3OD, 101 MHz)

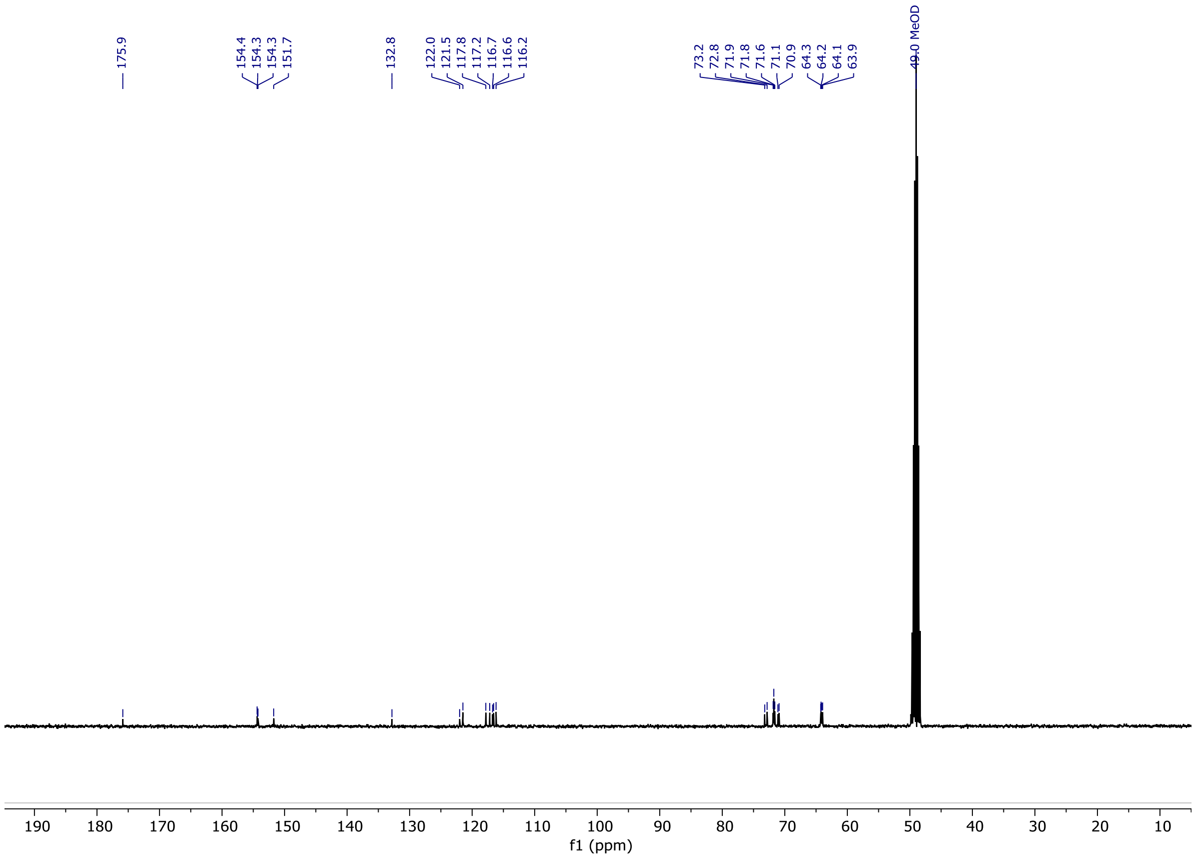

^{1}H NMR spectrum of **β-3** (CD_3OD, 400 MHz)

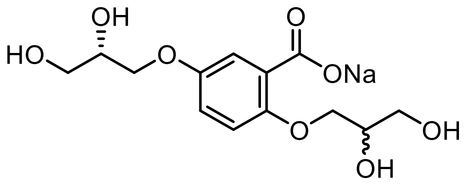

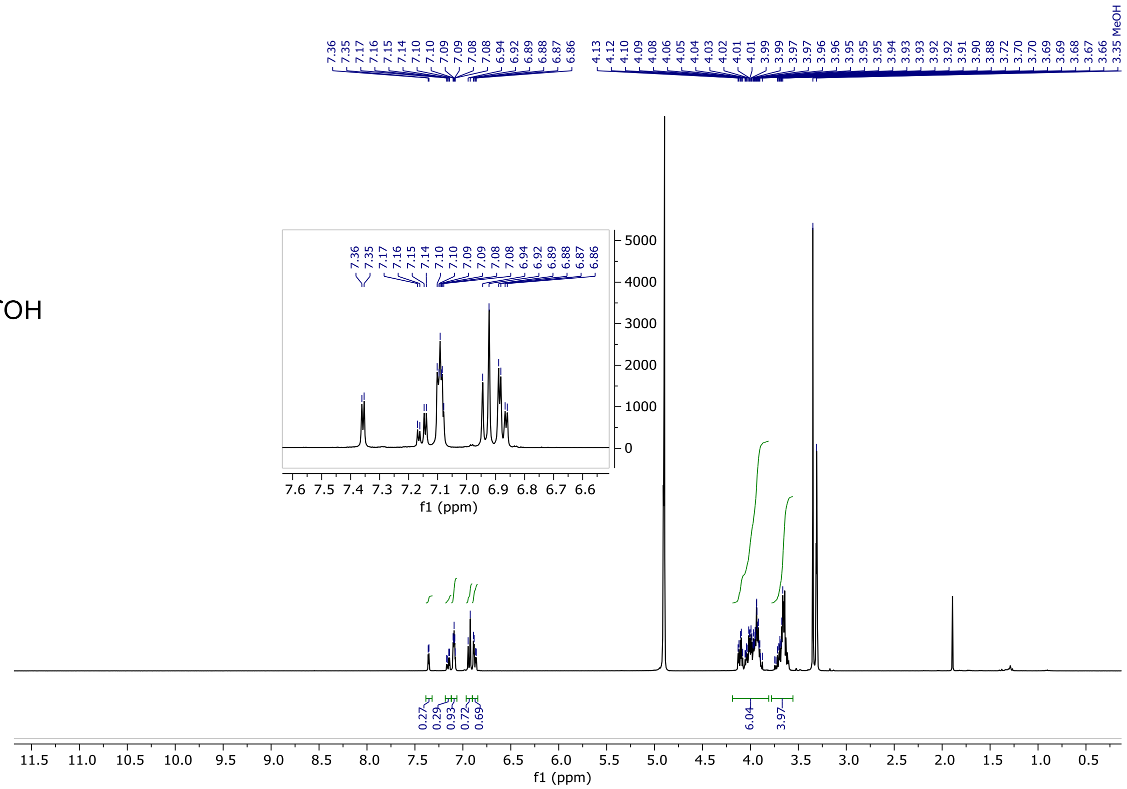

^{13}C NMR spectrum of **β-3** (CD_3OD, 101 MHz)

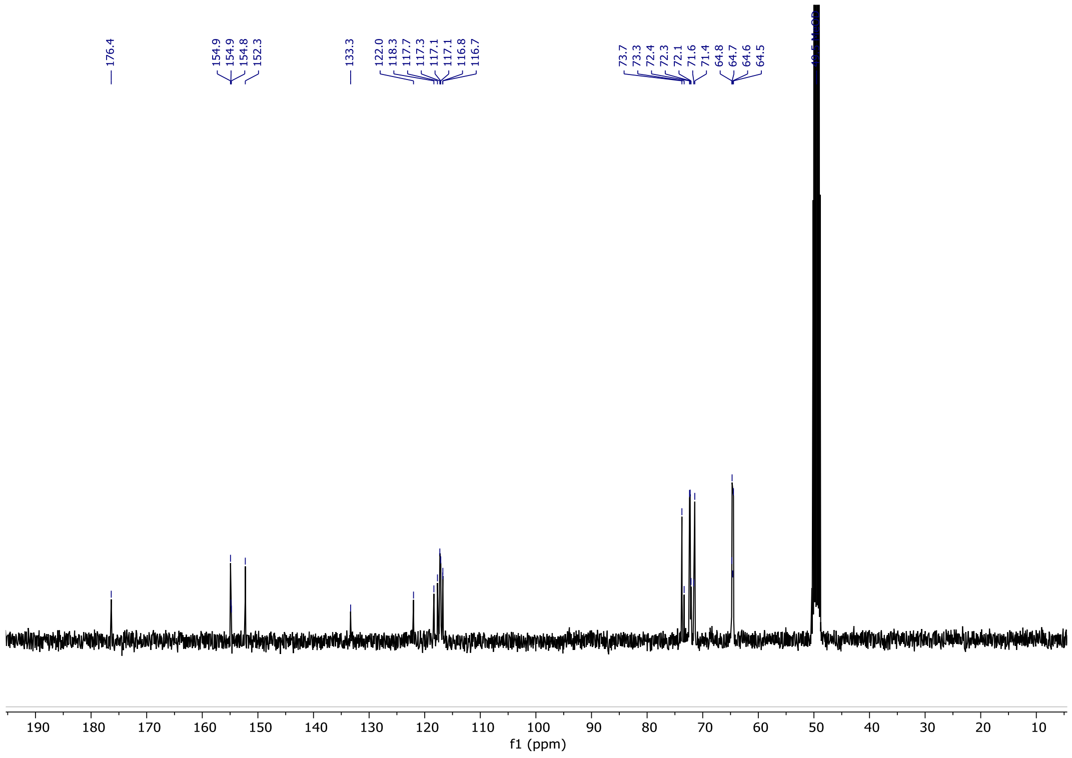

^{1}H NMR spectrum of α-**4** (D_2O, 400 MHz)

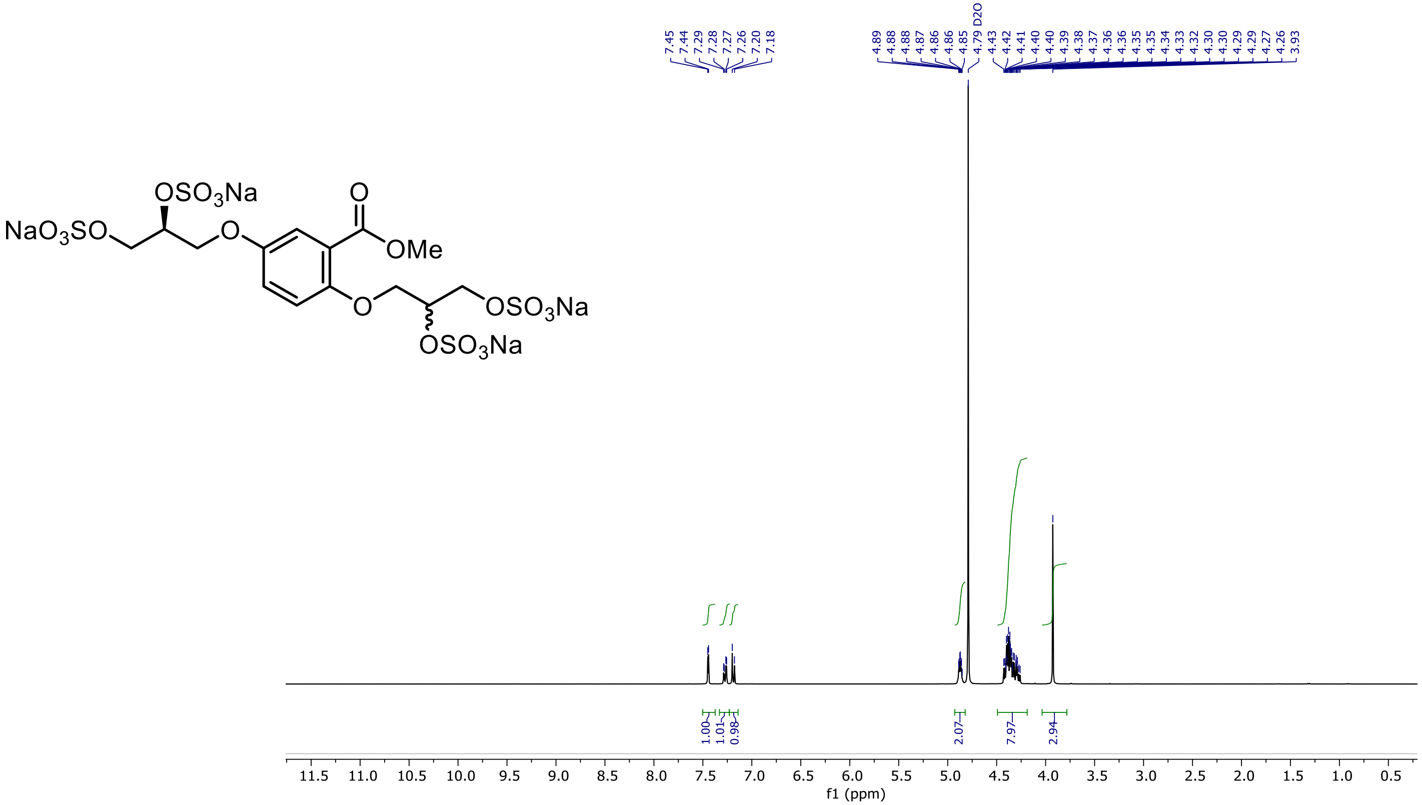

^{13}C NMR spectrum of α-**4** (D_2O, 101 MHz)

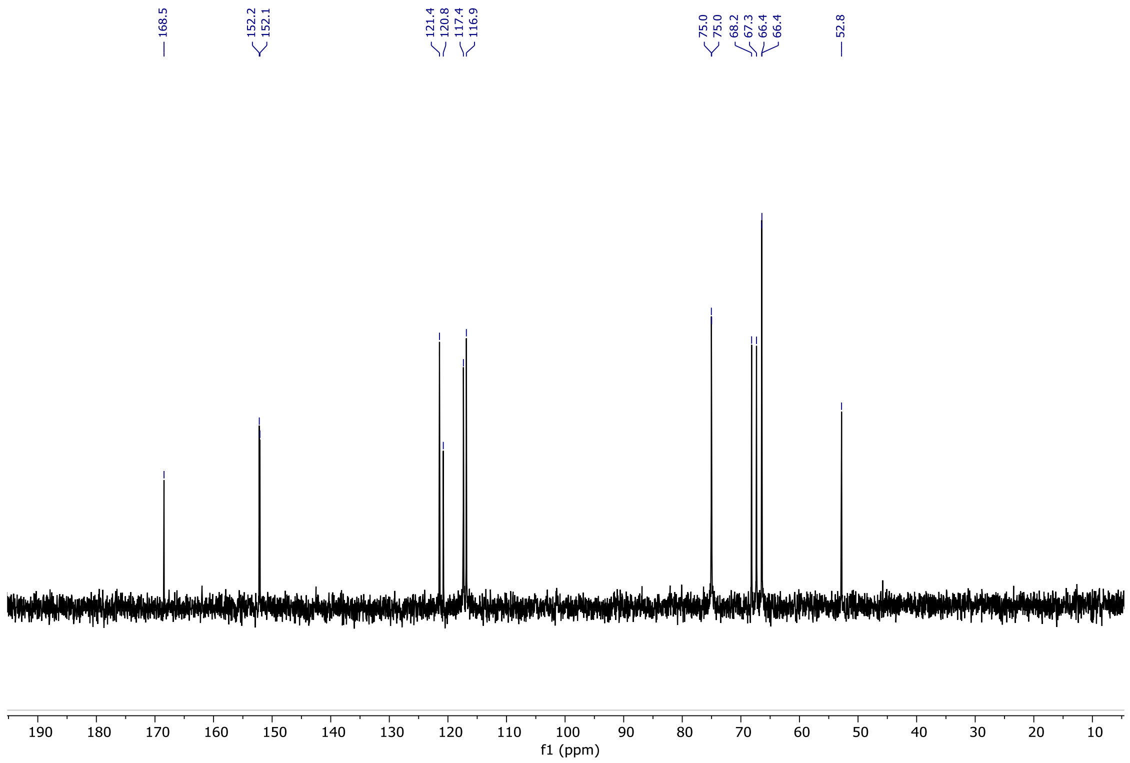

^{1}H NMR spectrum of β-**5** (D_2O, 400 MHz)

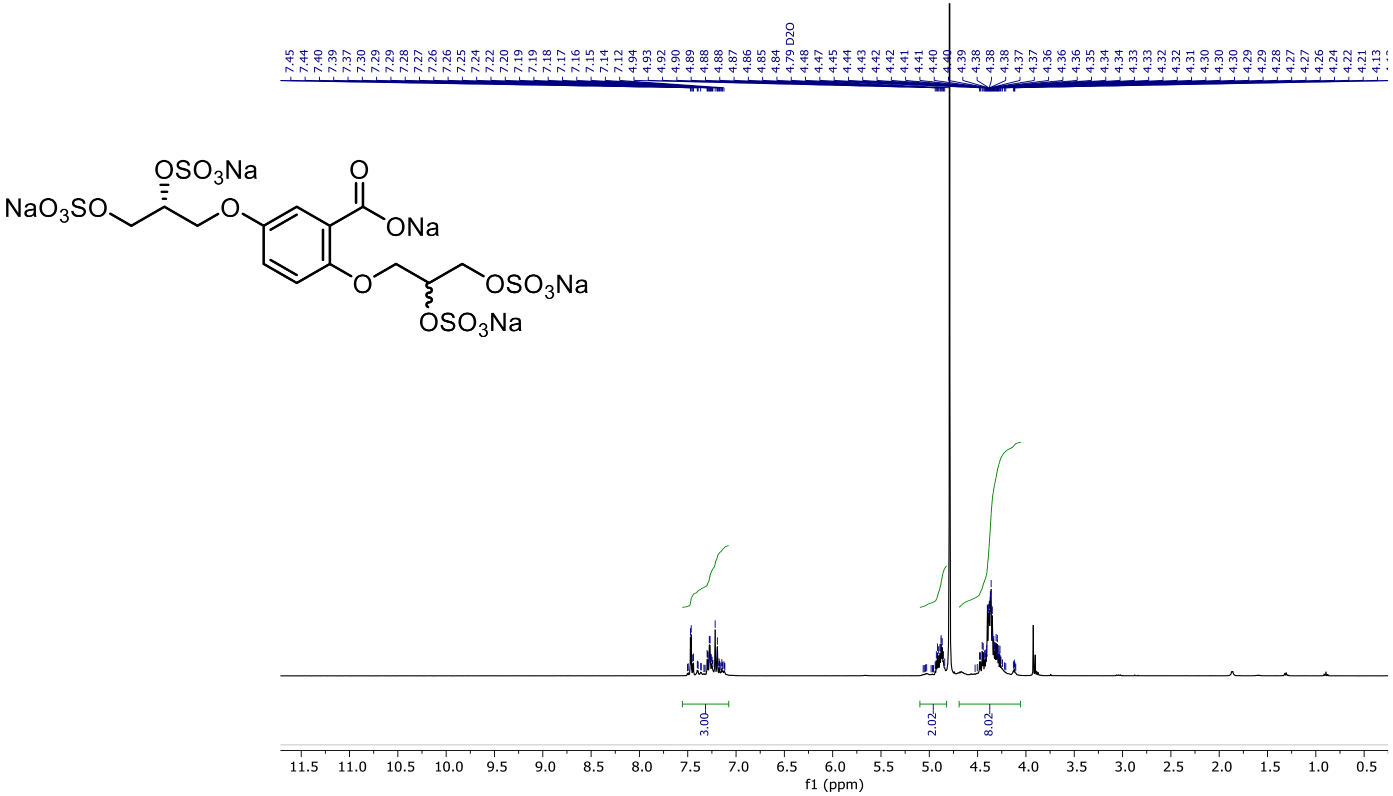

^{13}C NMR spectrum of β-**5** (D_2O, 101 MHz)

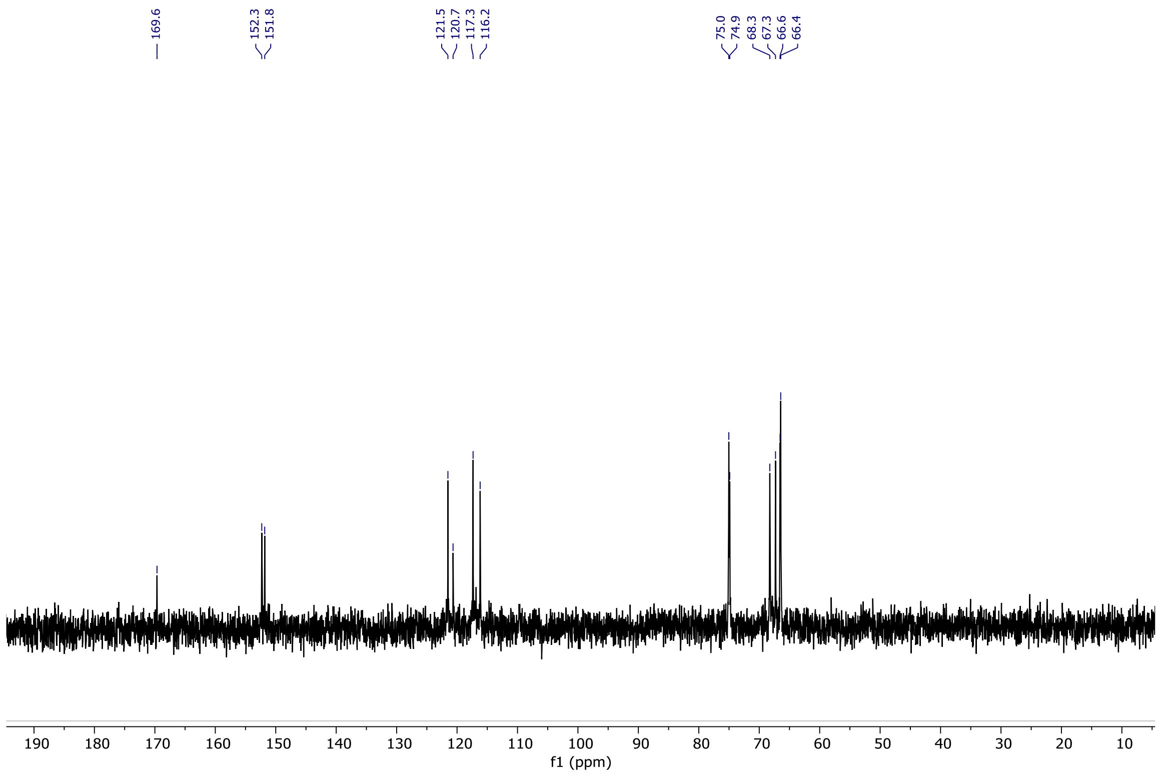

H NMR spectrum of **6** ($CDCl_3$, 400 MHz)

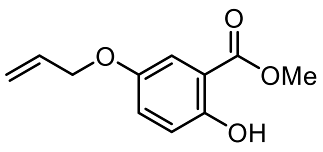

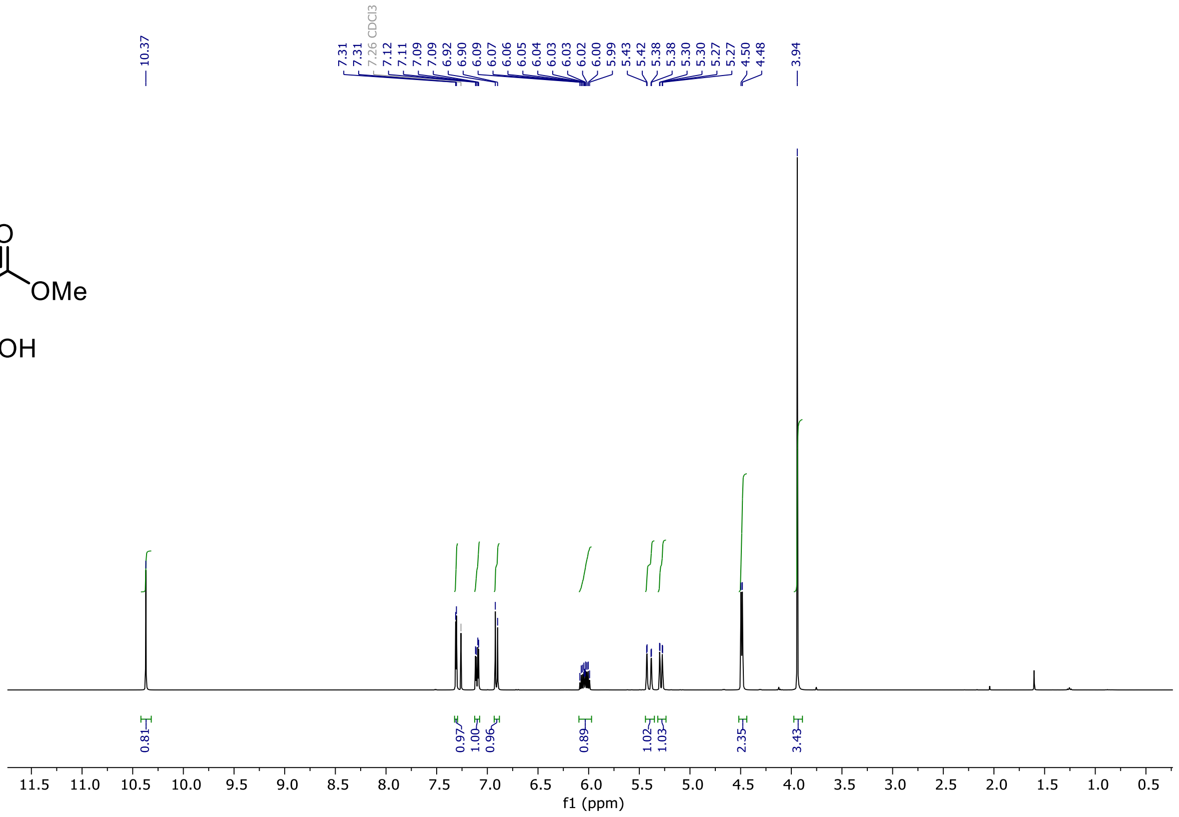

^{13}C NMR spectrum of **6** ($CDCl_3$, 101 MHz)

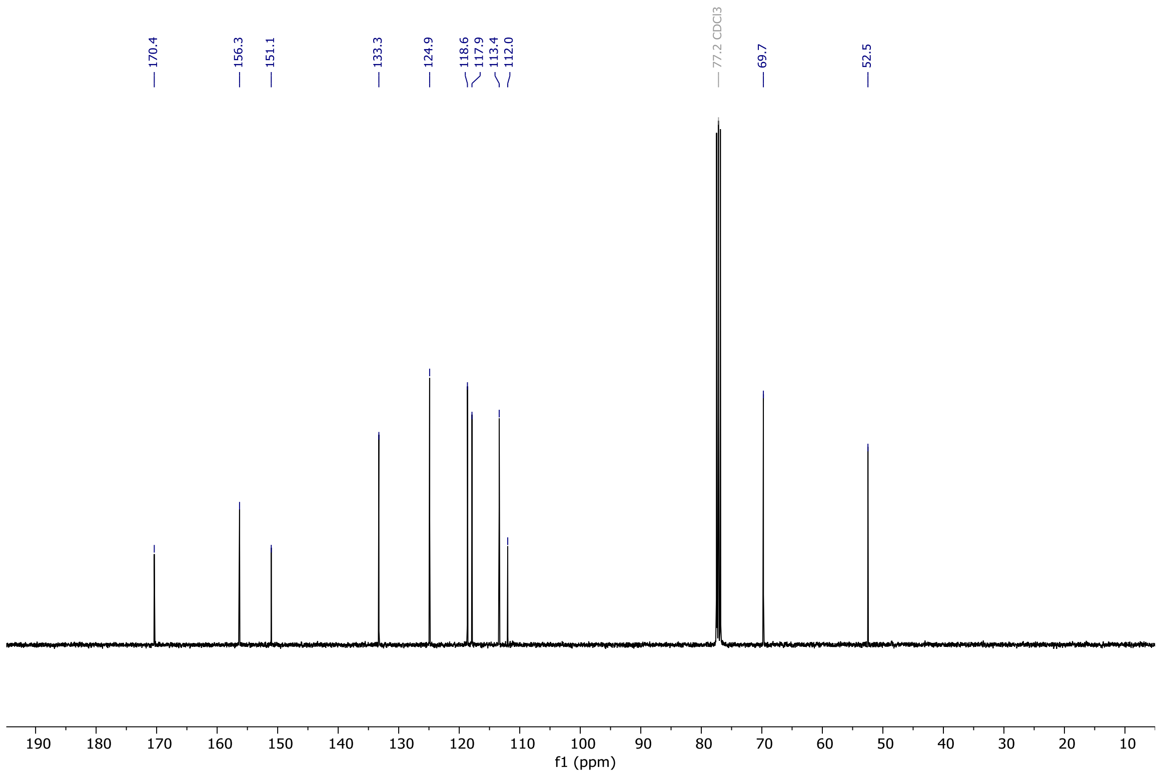

^{1}H NMR spectrum of **7** ($CDCl_3$, 400 MHz)

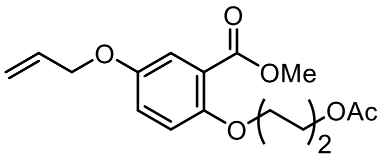

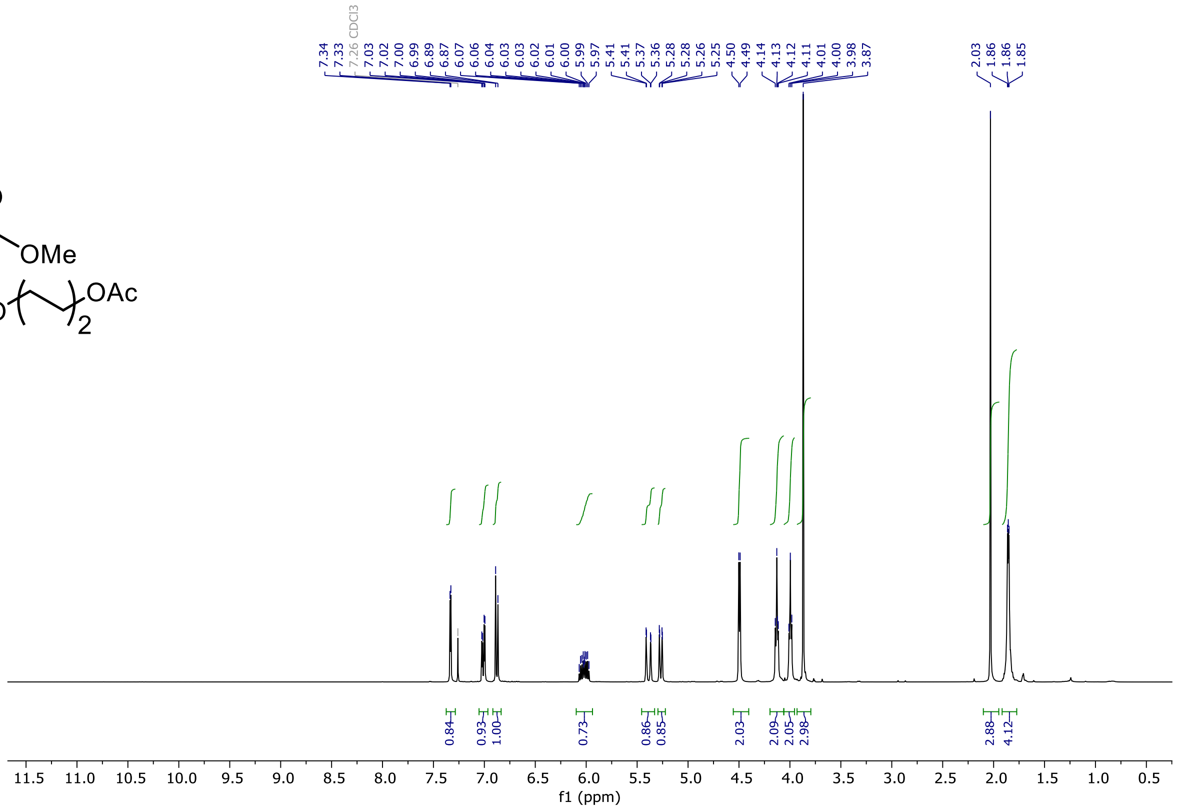

^{13}C NMR spectrum of **7** ($CDCl_3$, 101 MHz)

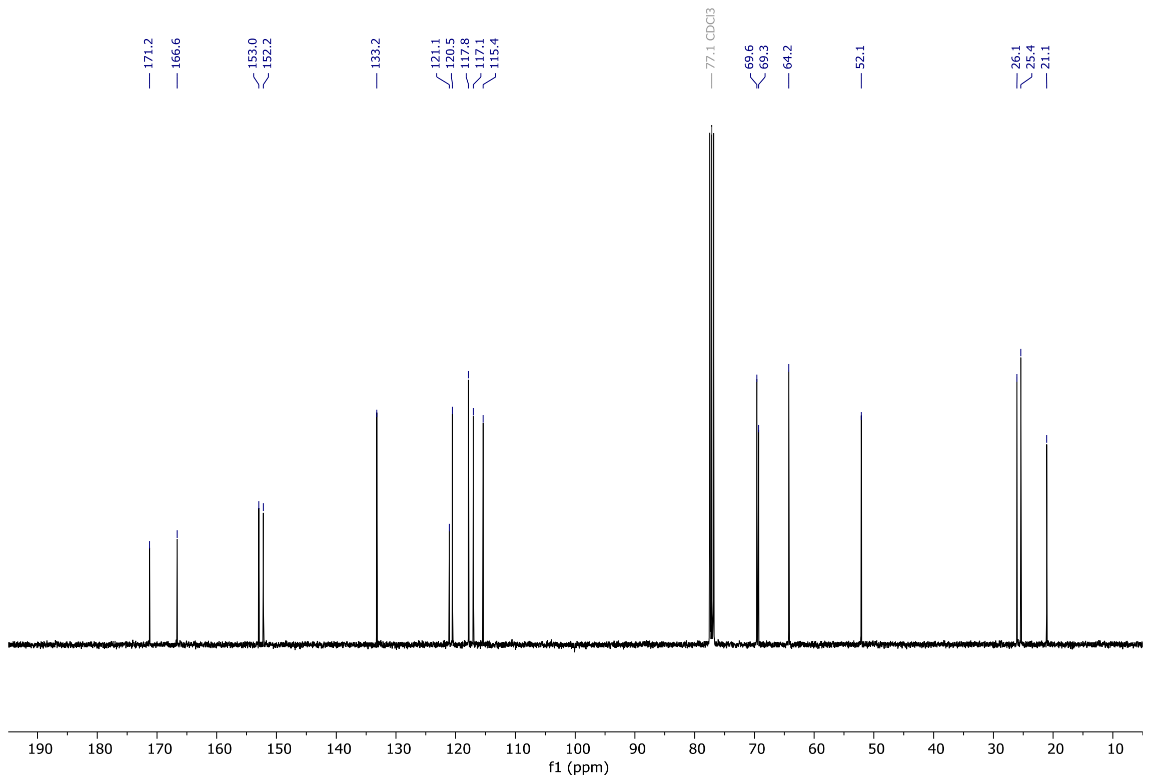

^{1}H NMR spectrum of *R*-**8** ($CDCl_3$, 400 MHz)

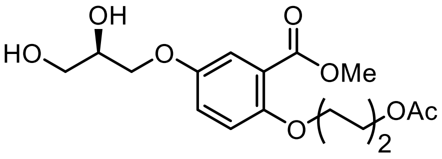

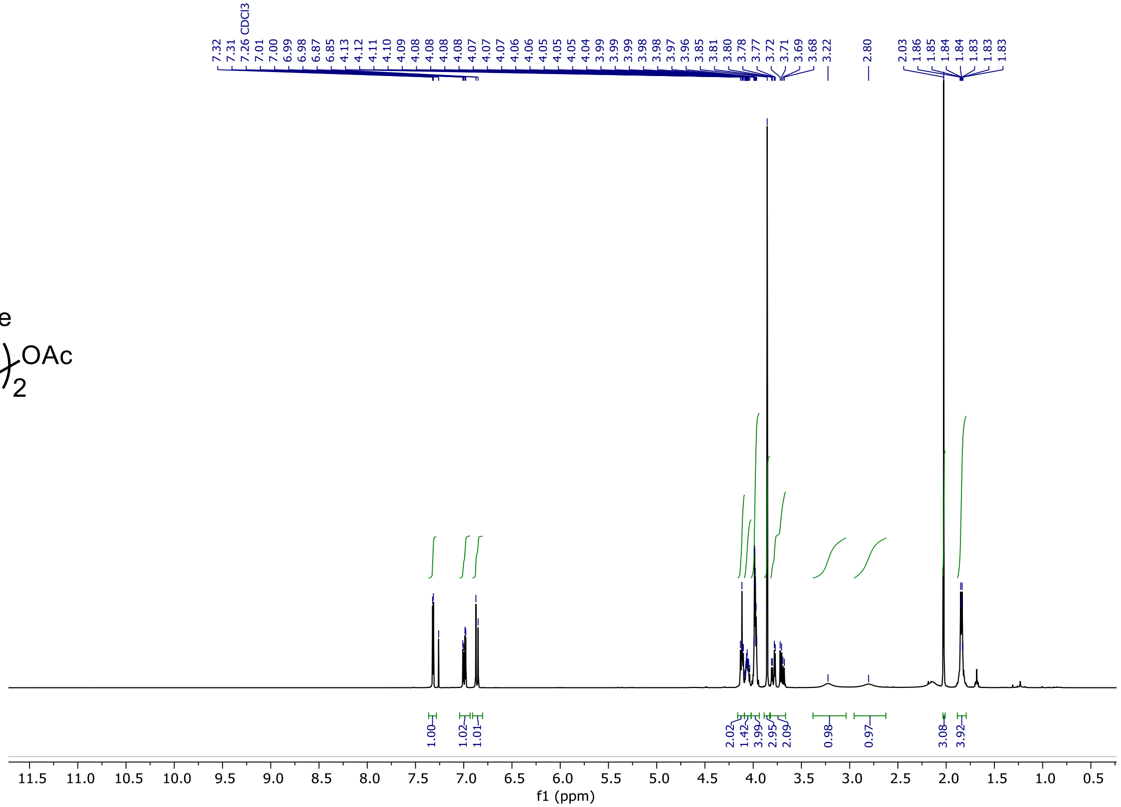

^{13}C NMR spectrum of *R*-**8** ($CDCl_3$, 101 MHz)

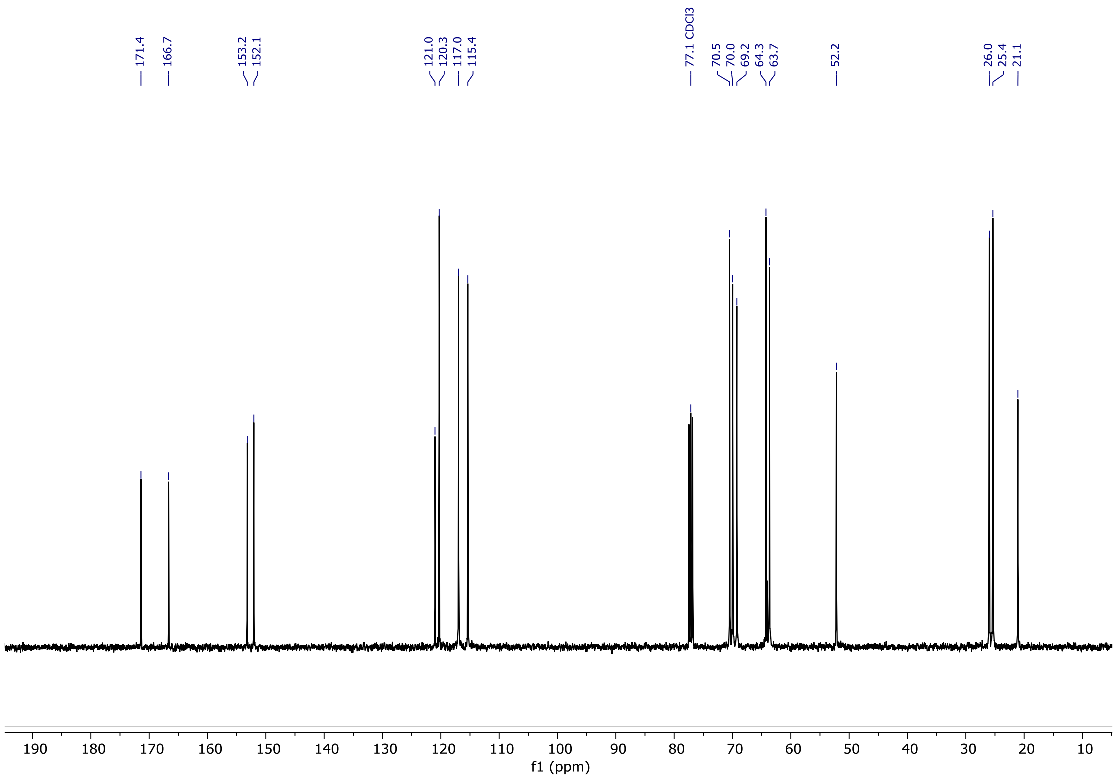

^{1}H NMR spectrum of *S*-**8** ($CDCl_3$, 400 MHz)

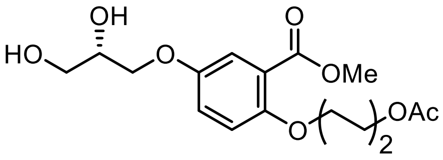

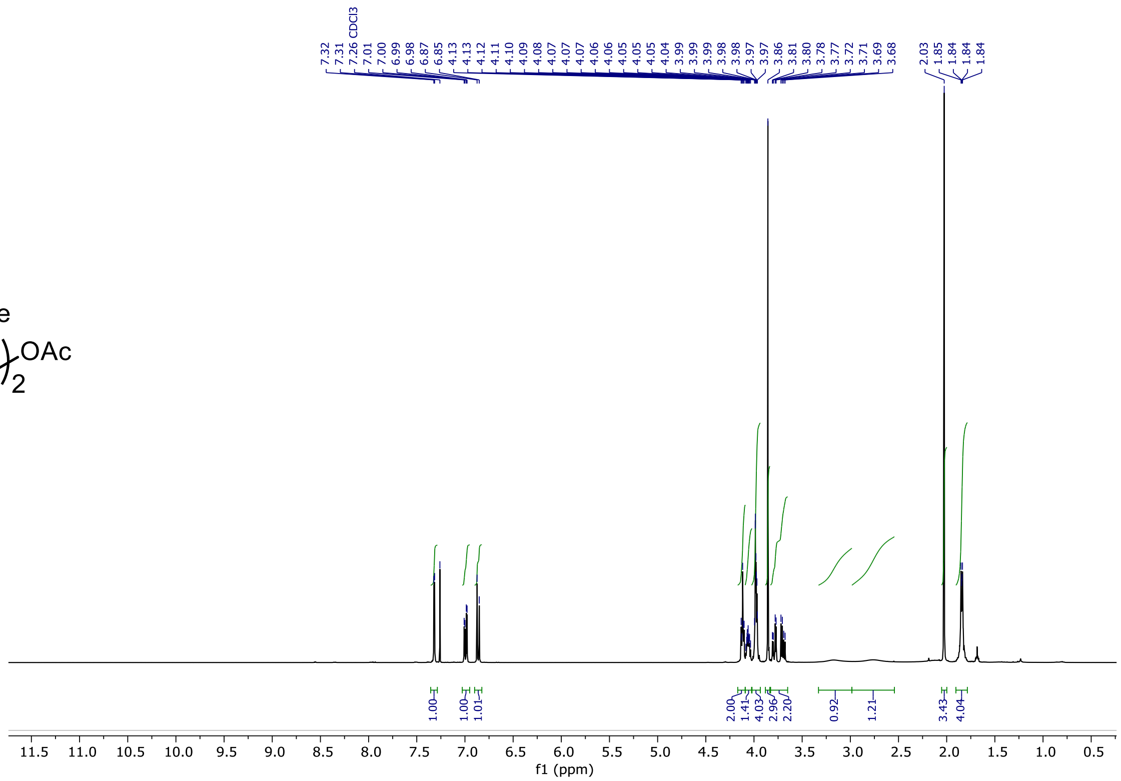

^{13}C NMR spectrum of *S*-**8** ($CDCl_3$, 101 MHz)

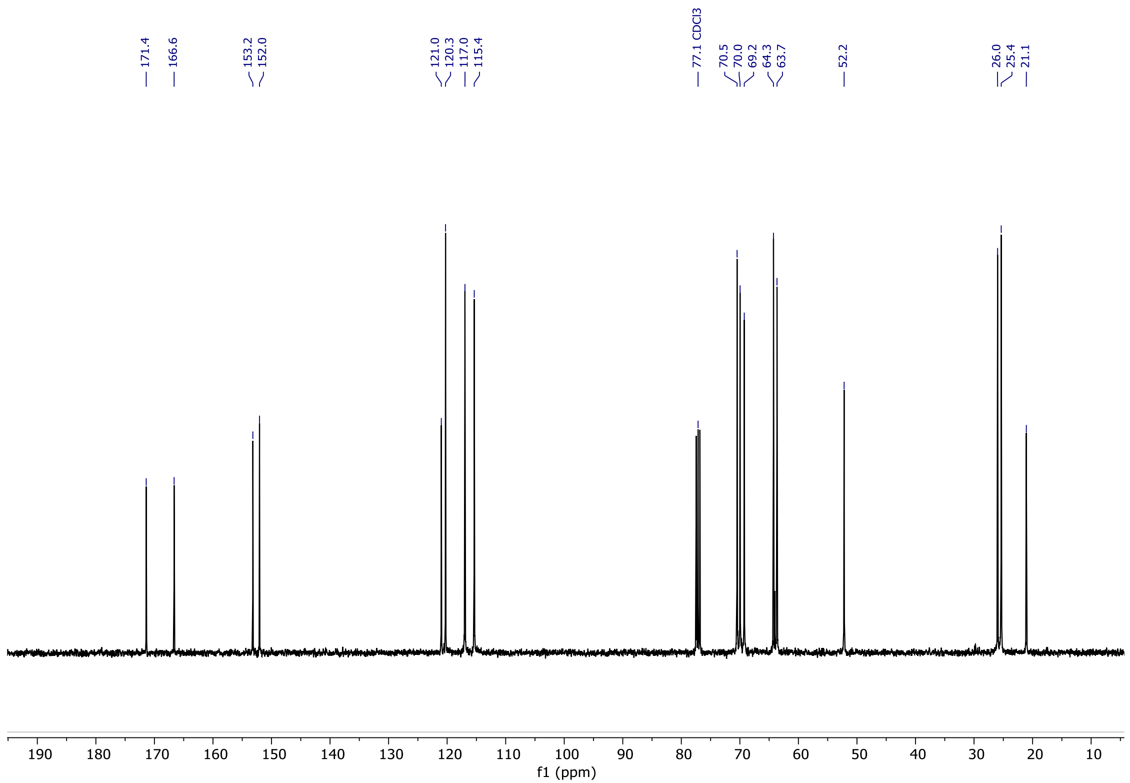

^{1}H NMR spectrum of *R*-**9** (CD_3OD, 400 MHz)

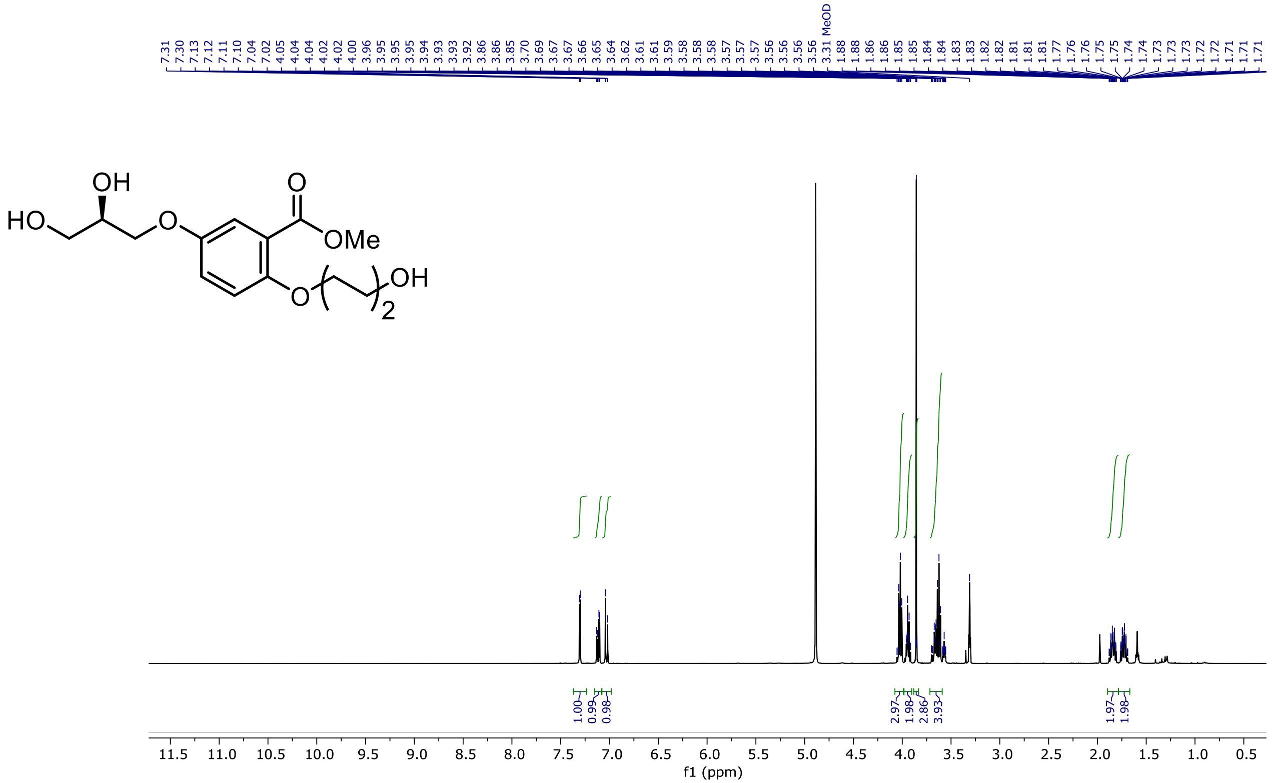

^{13}C NMR spectrum of *R*-**9** (CD_3OD, 101 MHz)

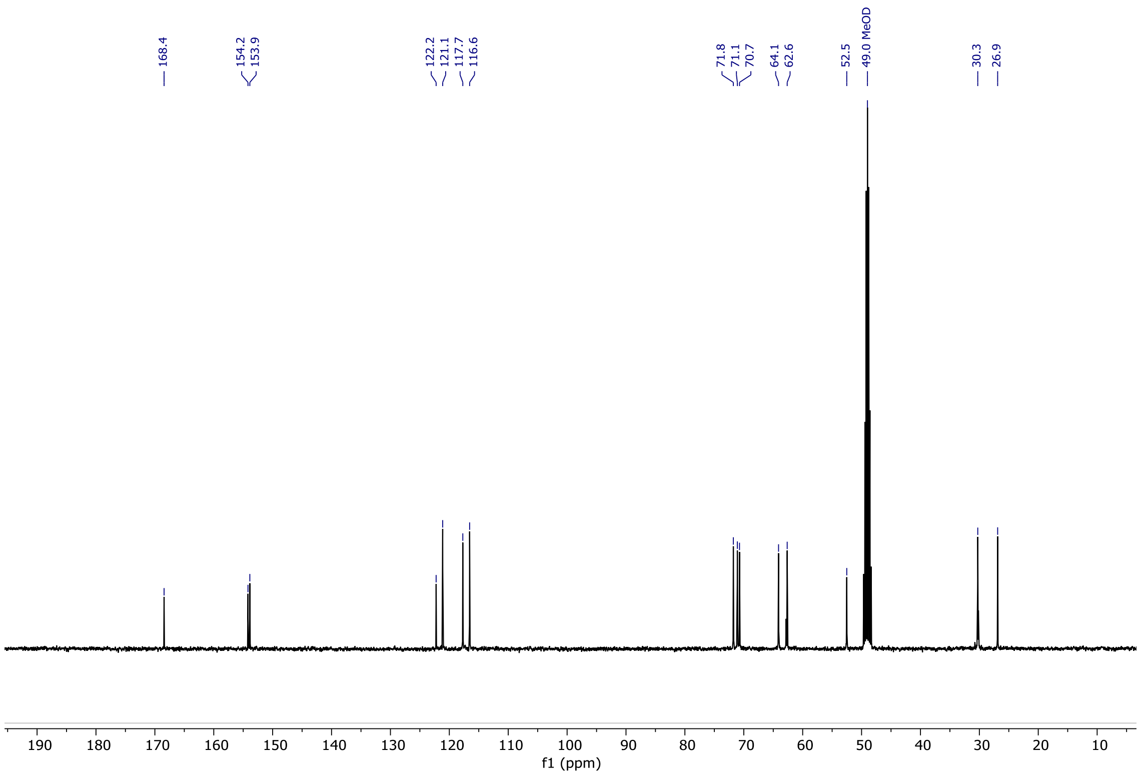

^{1}H NMR spectrum of *S*-**9** (CD_3OD, 400 MHz)

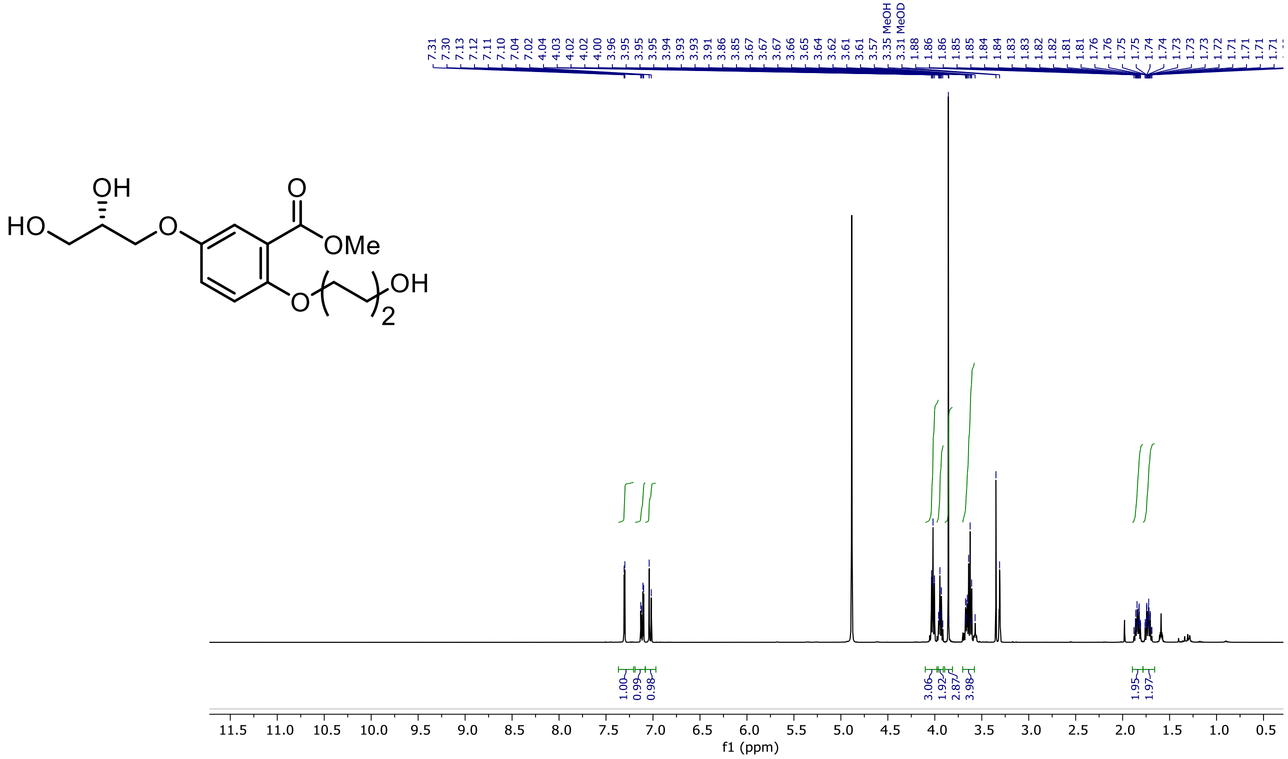

^{13}C NMR spectrum of *S*-**9** (CD_3OD, 101 MHz)

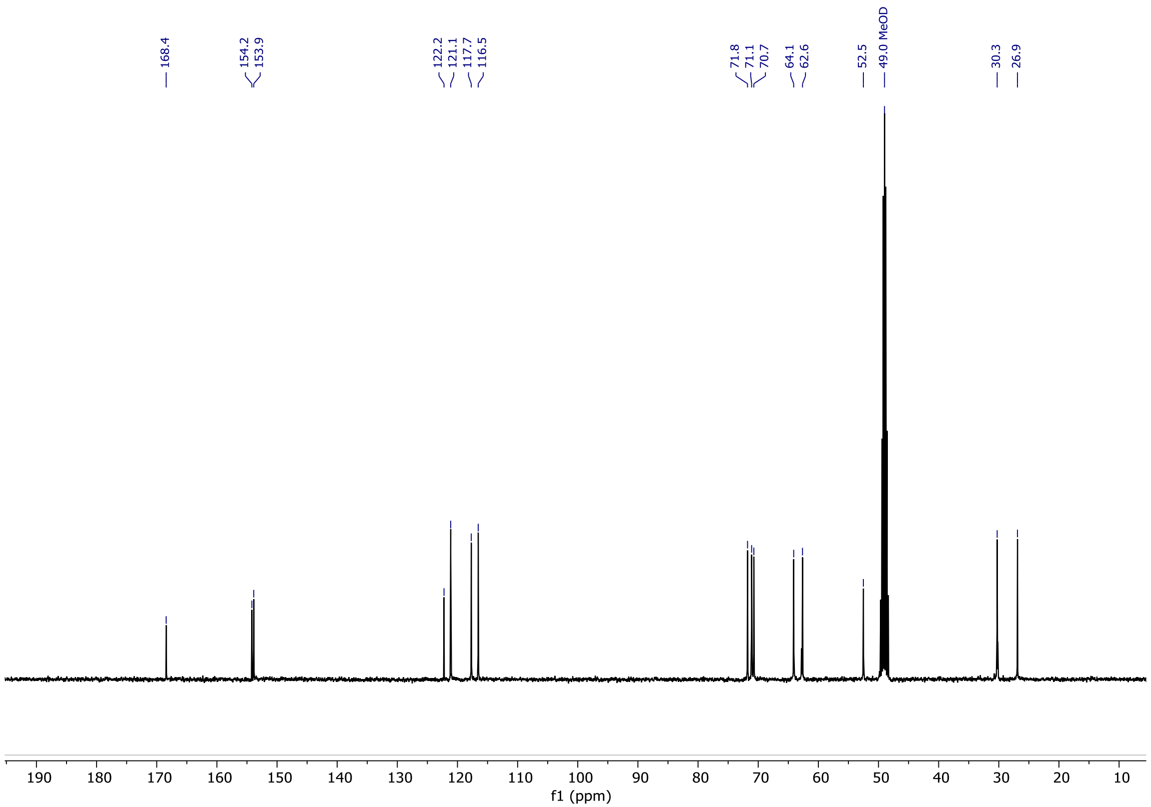

^{1}H NMR spectrum of *R*-**10** (D_2O, 400 MHz)

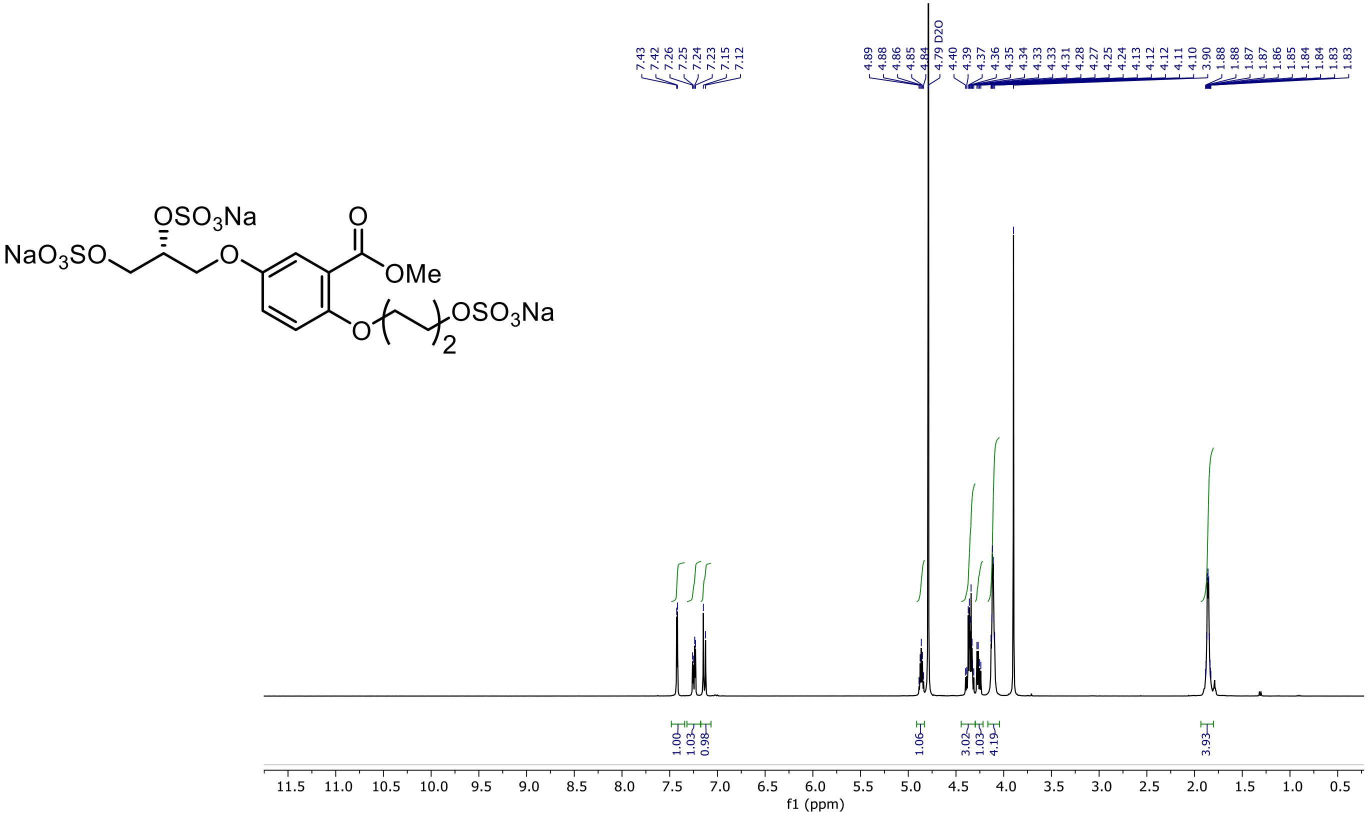

^{13}C NMR spectrum of *R*-**10** (D_2O, 101 MHz)

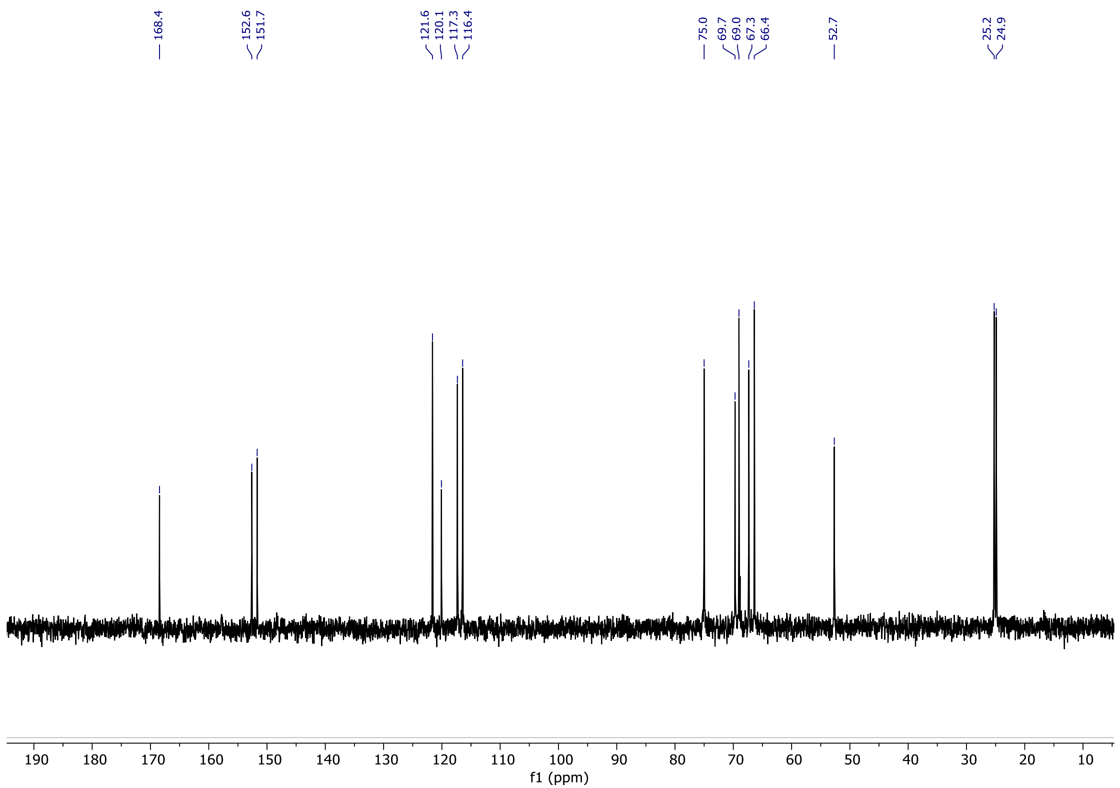

^{1}H NMR spectrum of *S*-**10** (D_2O, 400 MHz)

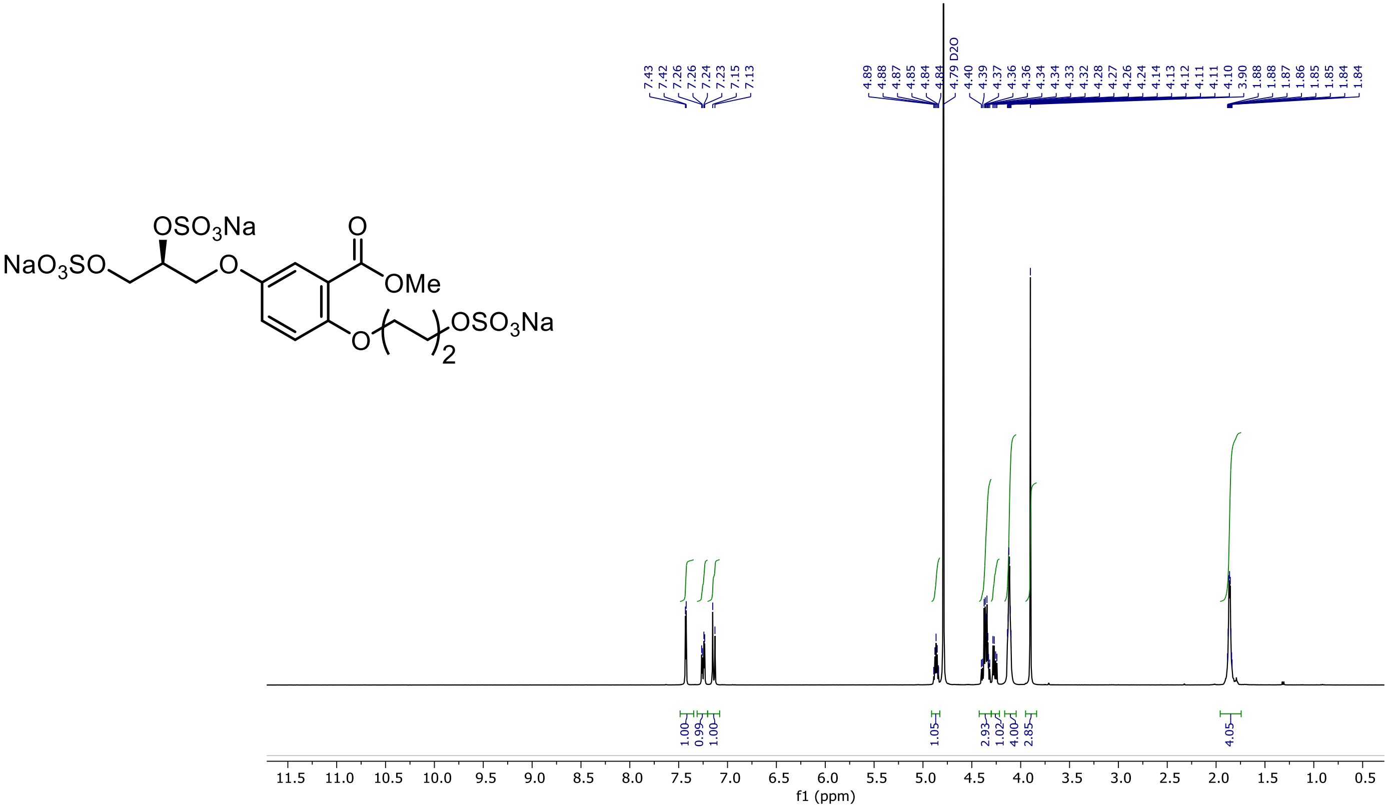

^{13}C NMR spectrum of *S*-**10** (D_2O, 101 MHz)

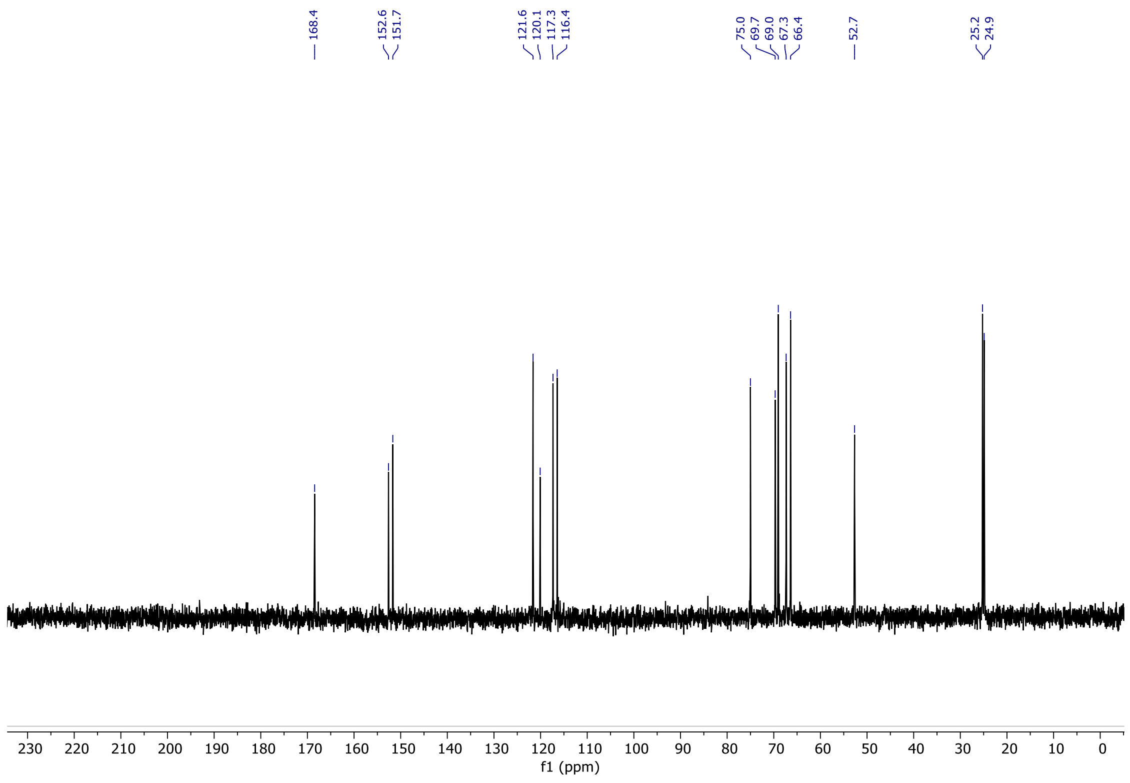

^{1}H-NMR spectrum of **14a** (400 MHz, $CDCl_3$)

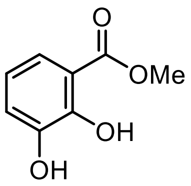

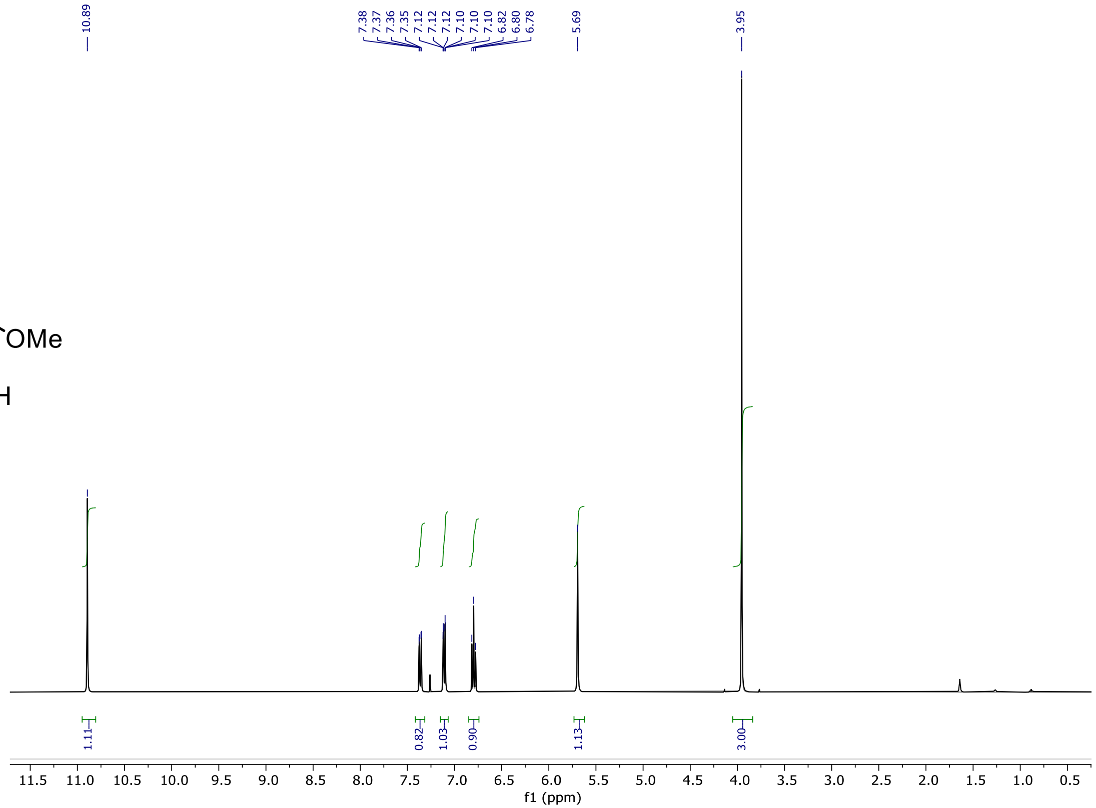

^{13}C-NMR spectrum of **14a** (101 MHz, $CDCl_3$)

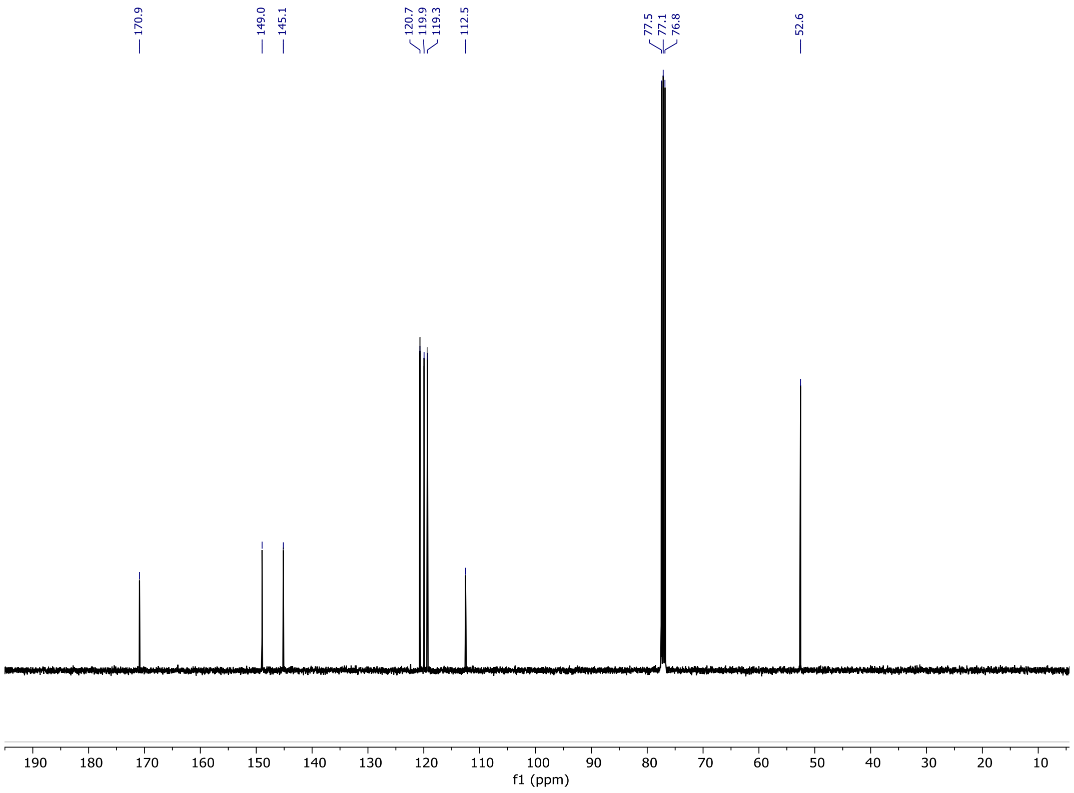

^{1}H-NMR spectrum of **14b** (400 MHz, $CDCl_3$)

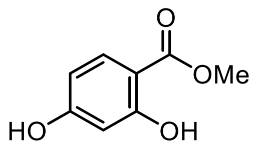

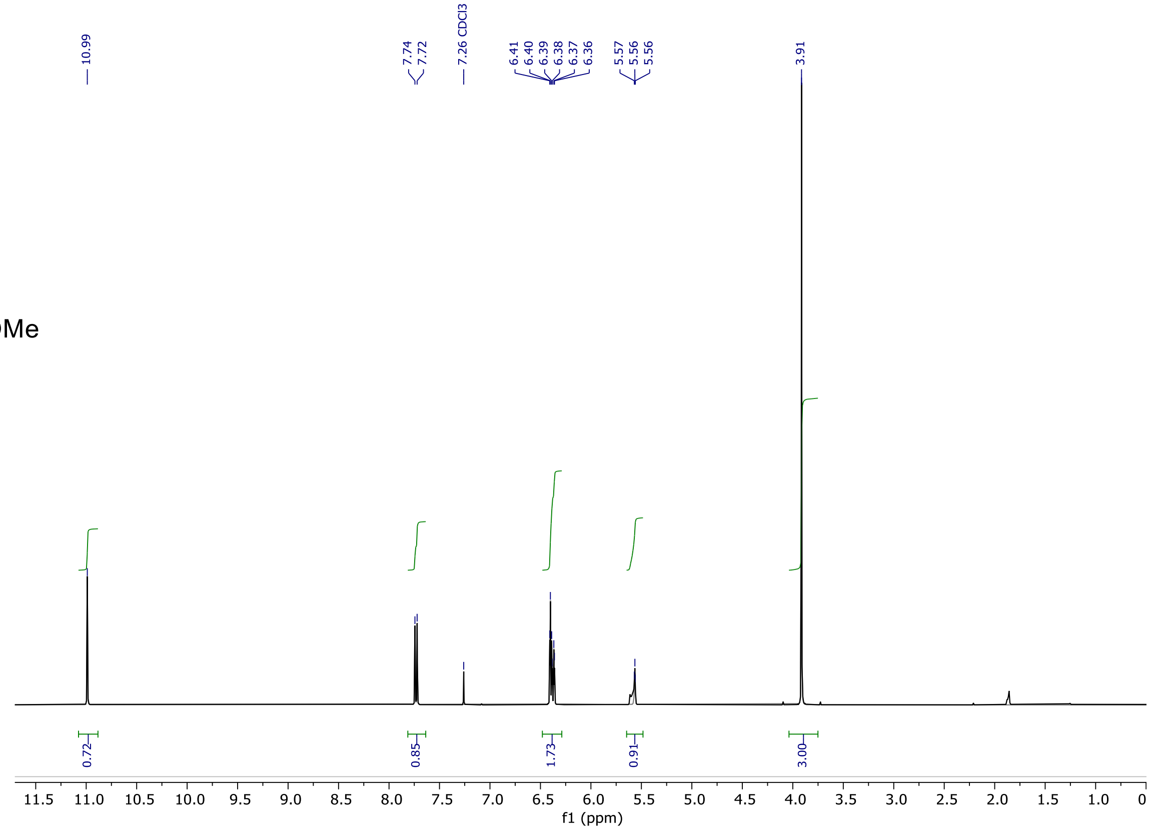

^{13}C-NMR spectrum of **14b** (101 MHz, $CDCl_3$

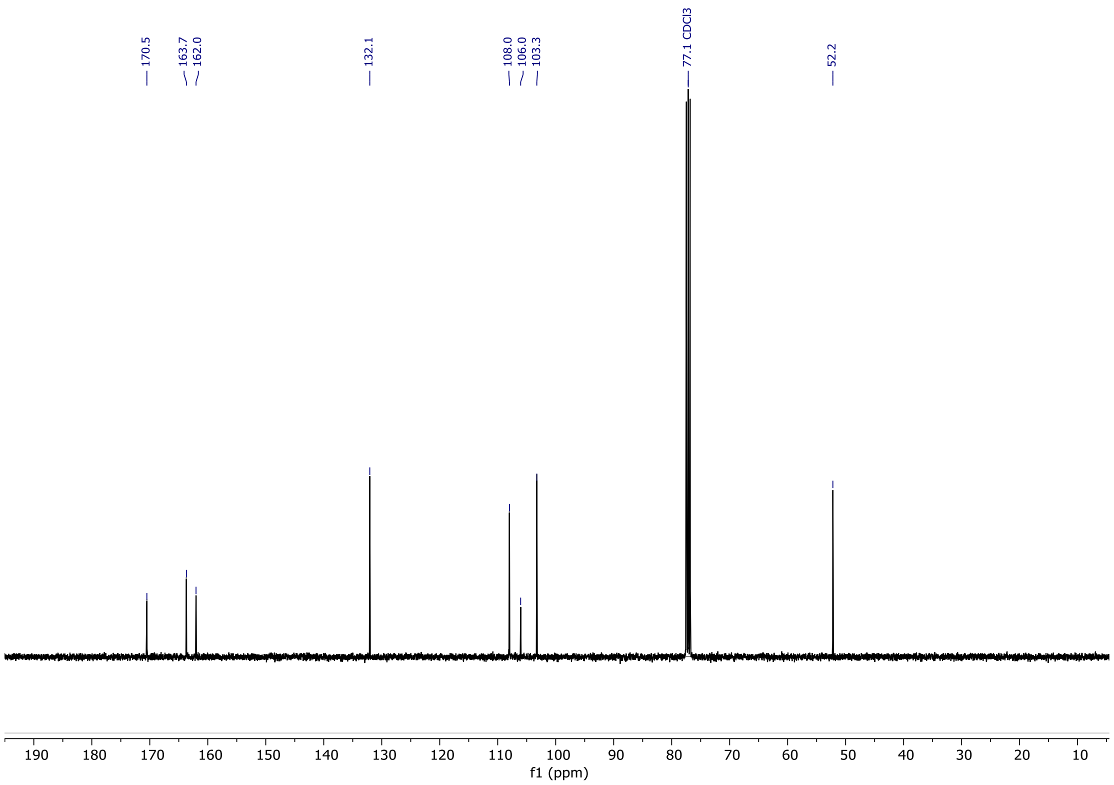

^{1}H-NMR spectrum of **14c** (400 MHz, $(CD_3)_2SO$)

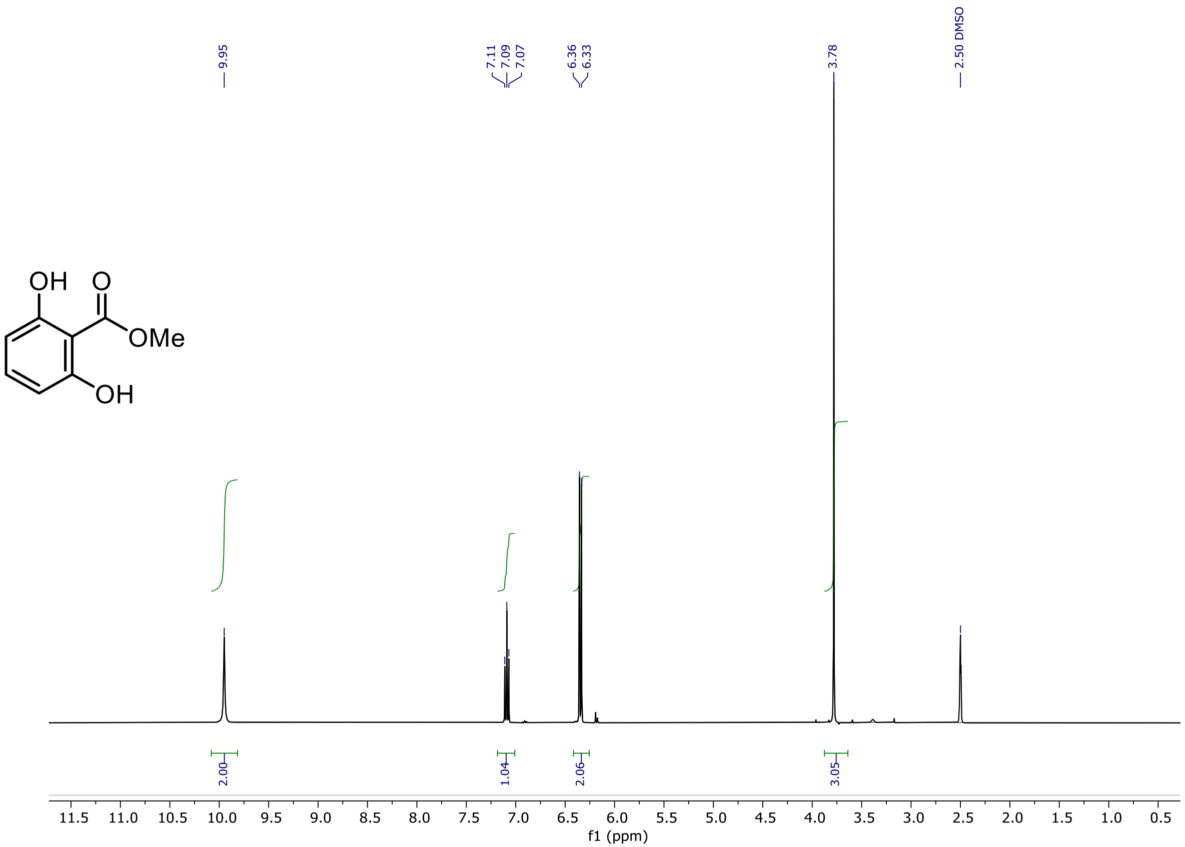

^{13}C-NMR spectrum of **14c** (101 MHz, $(CD_3)_2SO$)

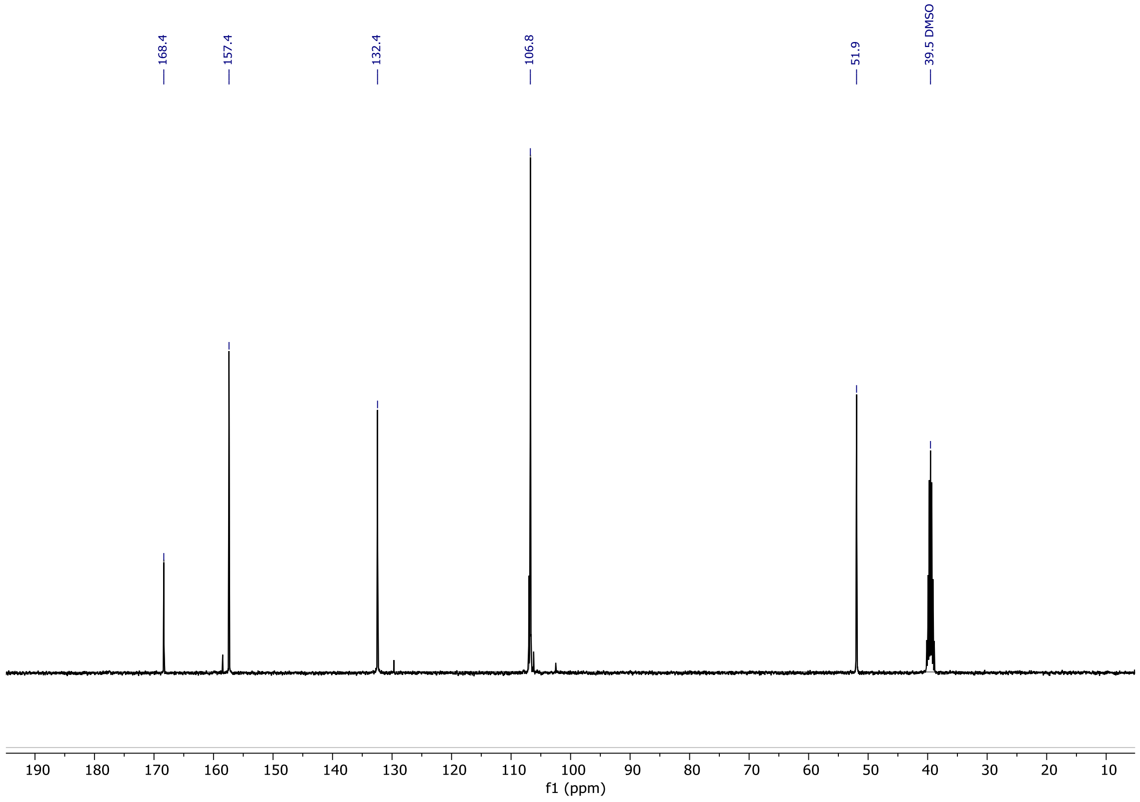

^{1}H-NMR spectrum of **14d** (400 MHz, $(CD_3)_2SO$)

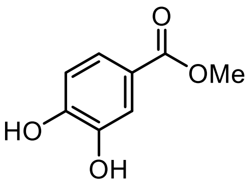

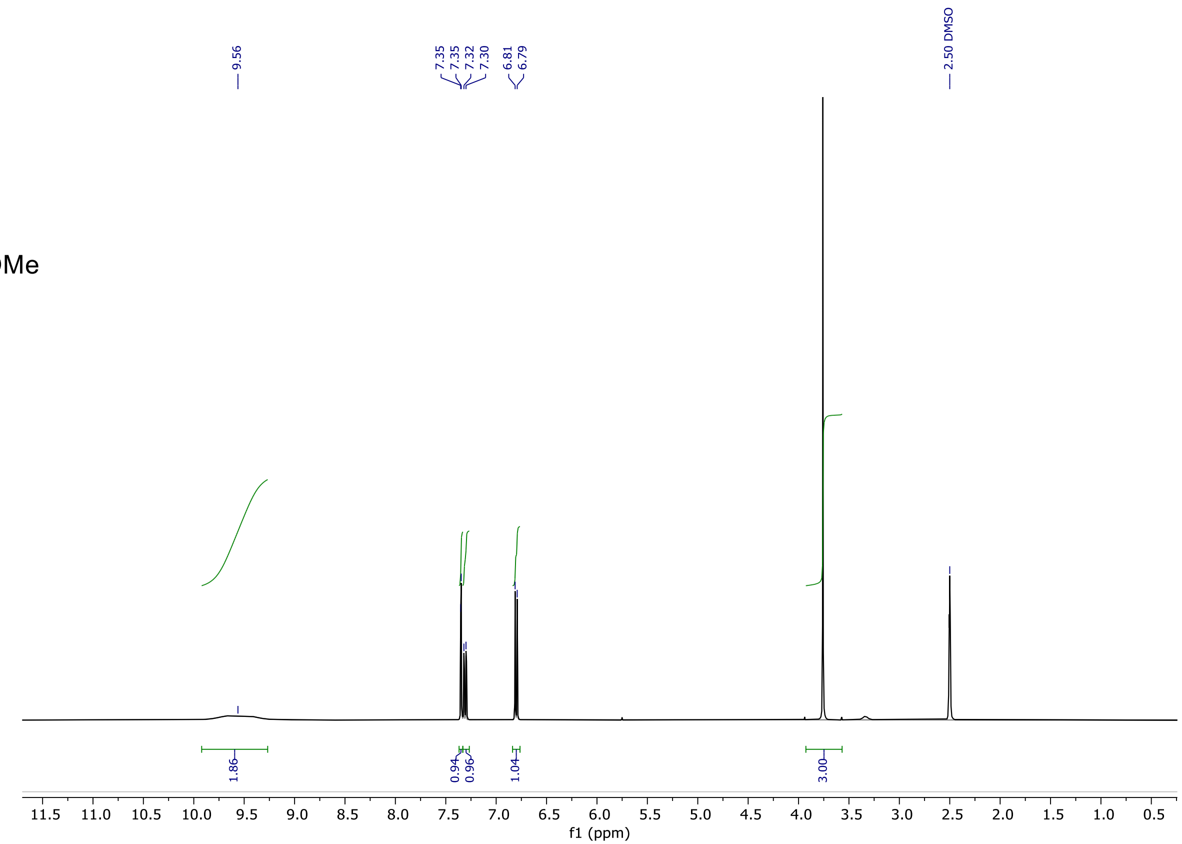

^{13}C-NMR spectrum of **14d** (101 MHz, $(CD_3)_2SO$)

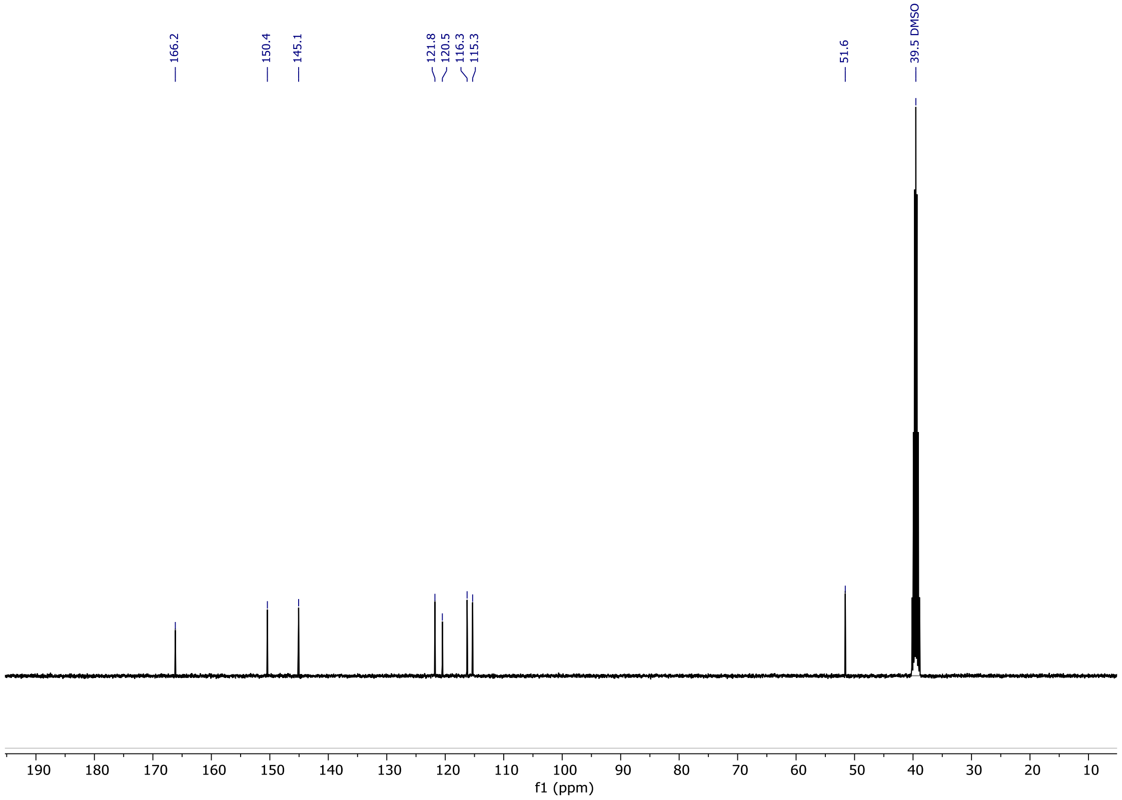

^{1}H-NMR spectrum of **14e** (400 MHz, $(CD_3)_2SO$)

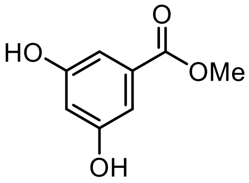

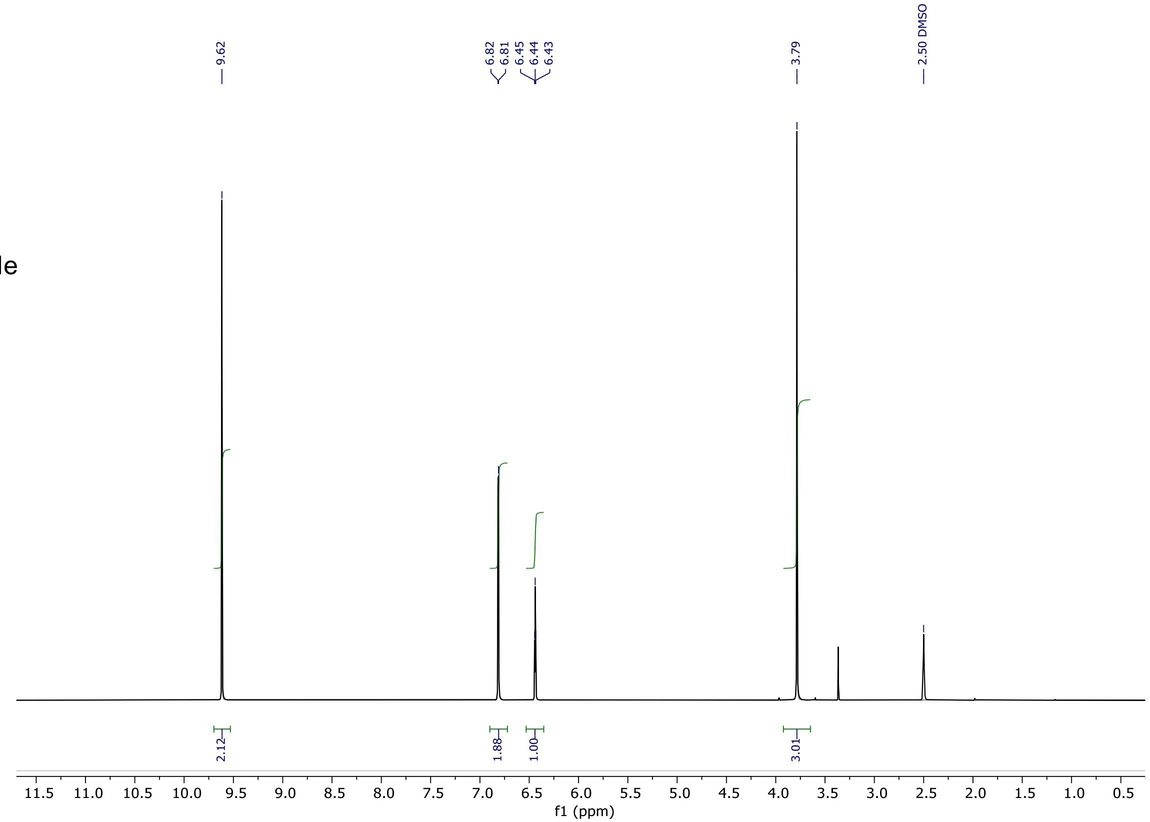

^{13}C-NMR spectrum of **14e** (101 MHz, $(CD_3)_2SO$)

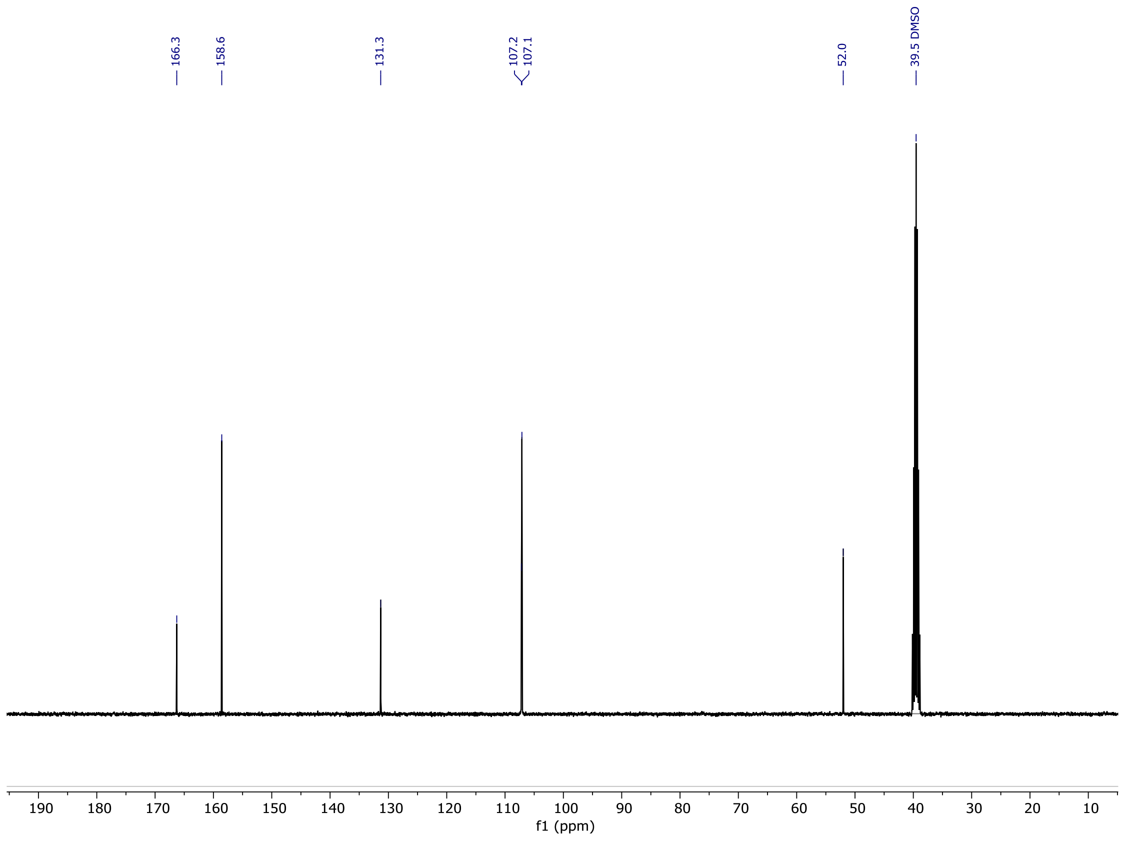

^{1}H-NMR spectrum **14g** ($CDCl_3$, 400 MHz)

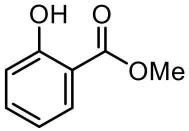

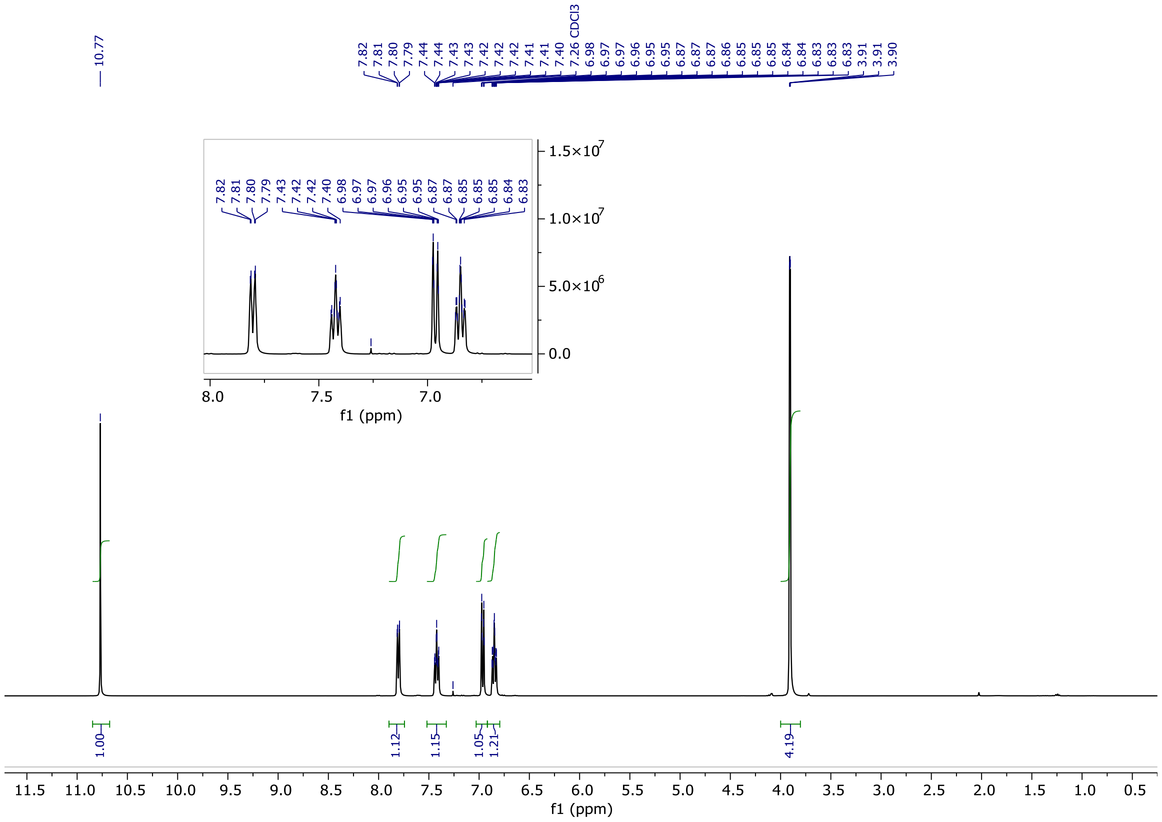

^{13}C-NMR spectrum **14g** ($CDCl_3$, 101 MHz)

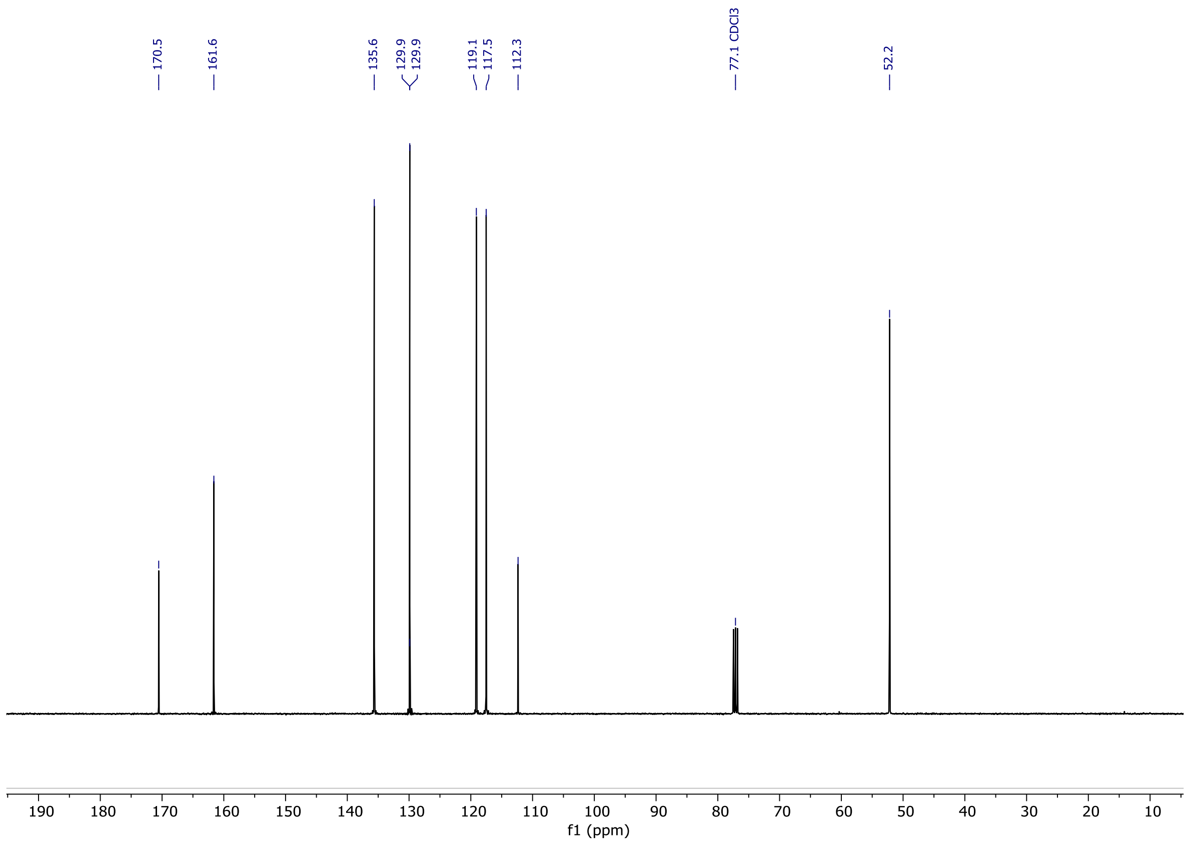

^{1}H-NMR spectrum **14h** ($(CD_3)_2CO$, 400 MHz)

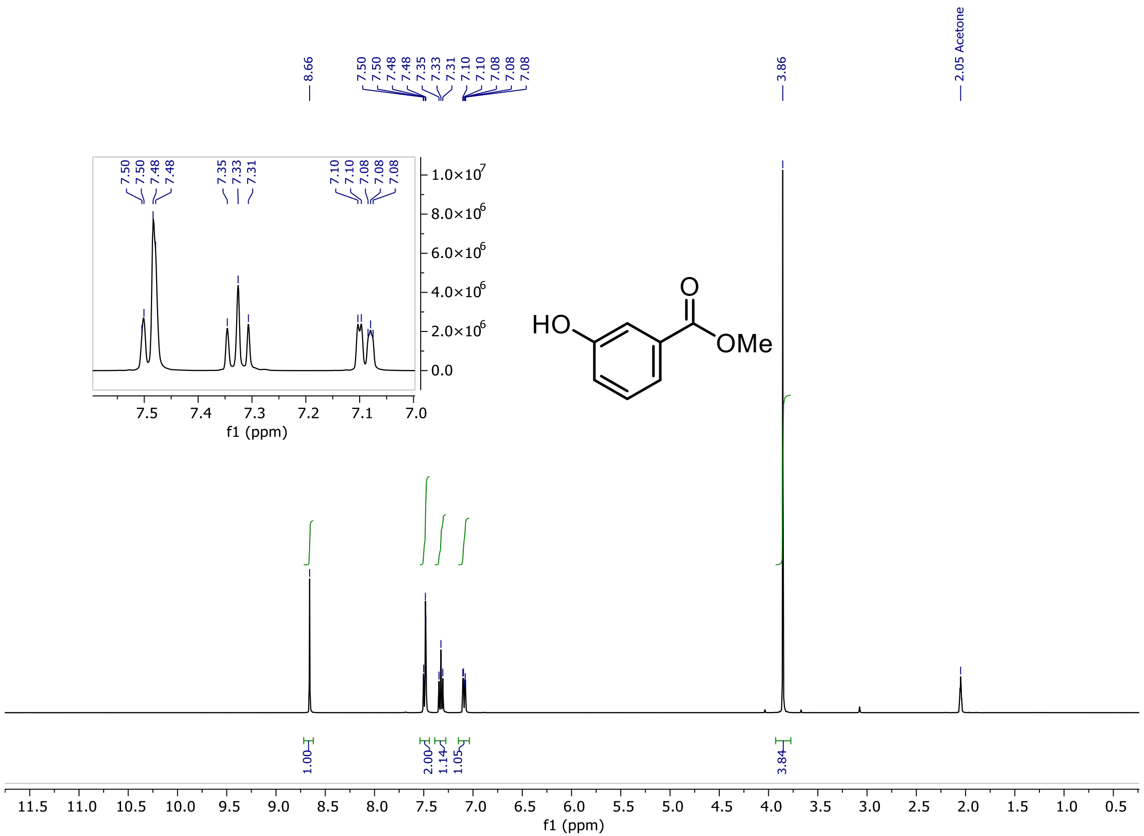

^{13}C-NMR spectrum **14h** ($(CD_3)_2CO$, 101 MHz)

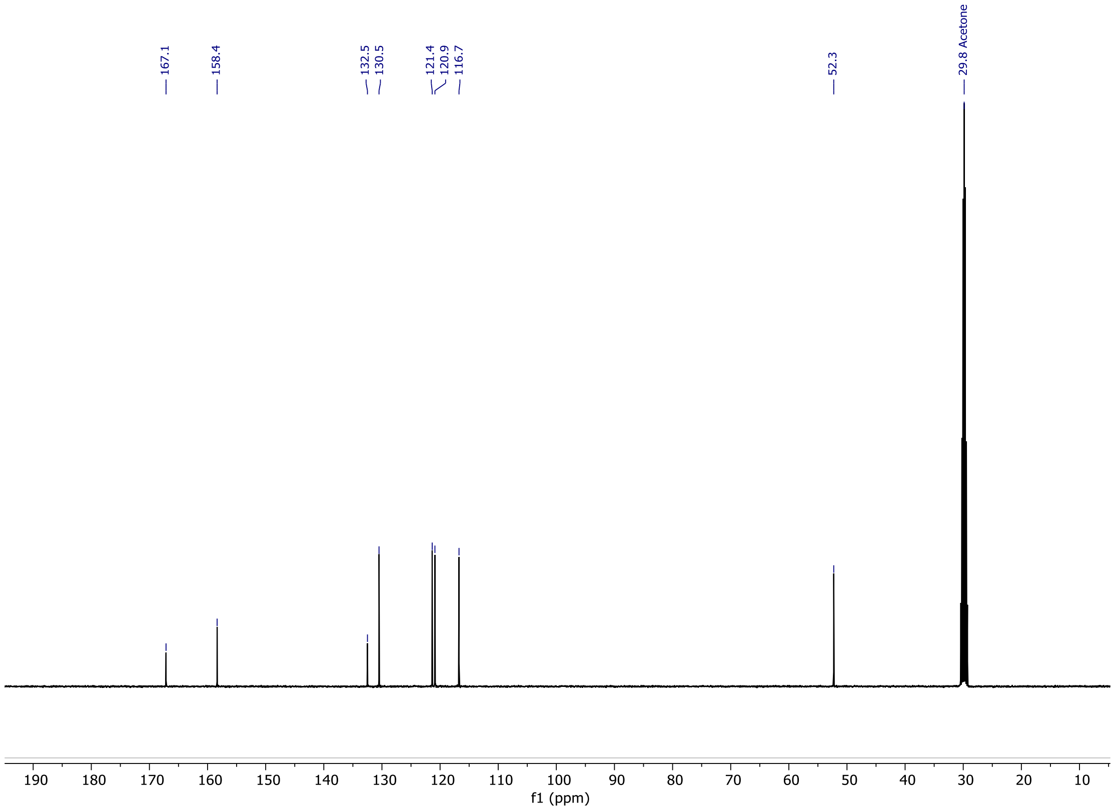

^{1}H-NMR spectrum **14i** ($CDCl_3$, 400 MHz)

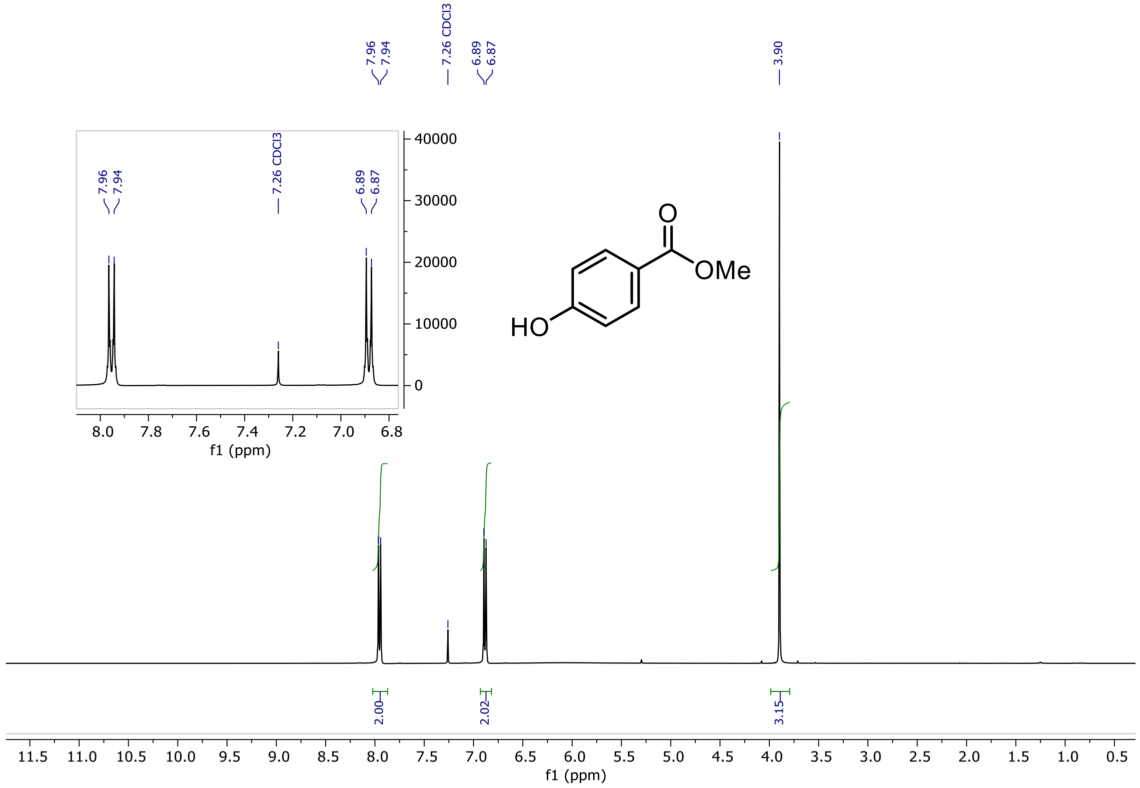

^{13}C-NMR spectrum **14i** ($CDCl_3$, 101 MHz)

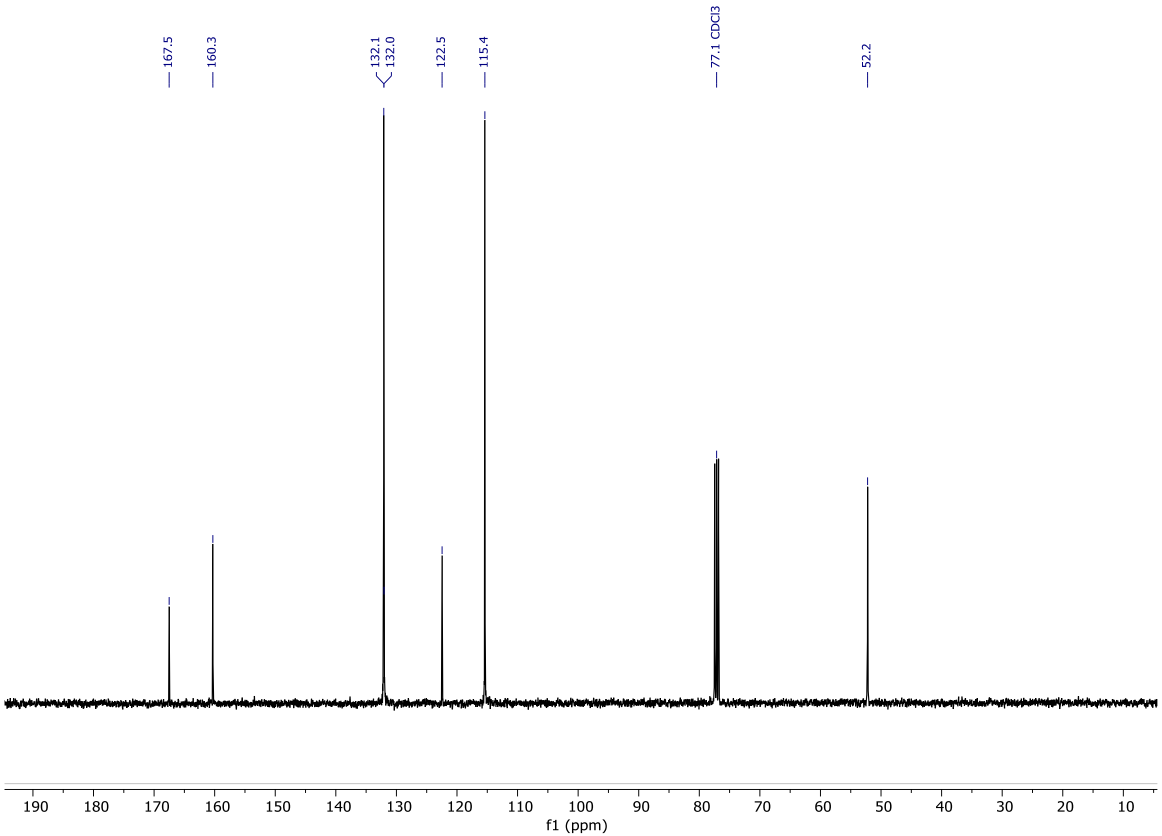

^{1}H-NMR spectrum of **15a** (400 MHz, $CDCl_3$)

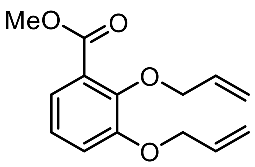

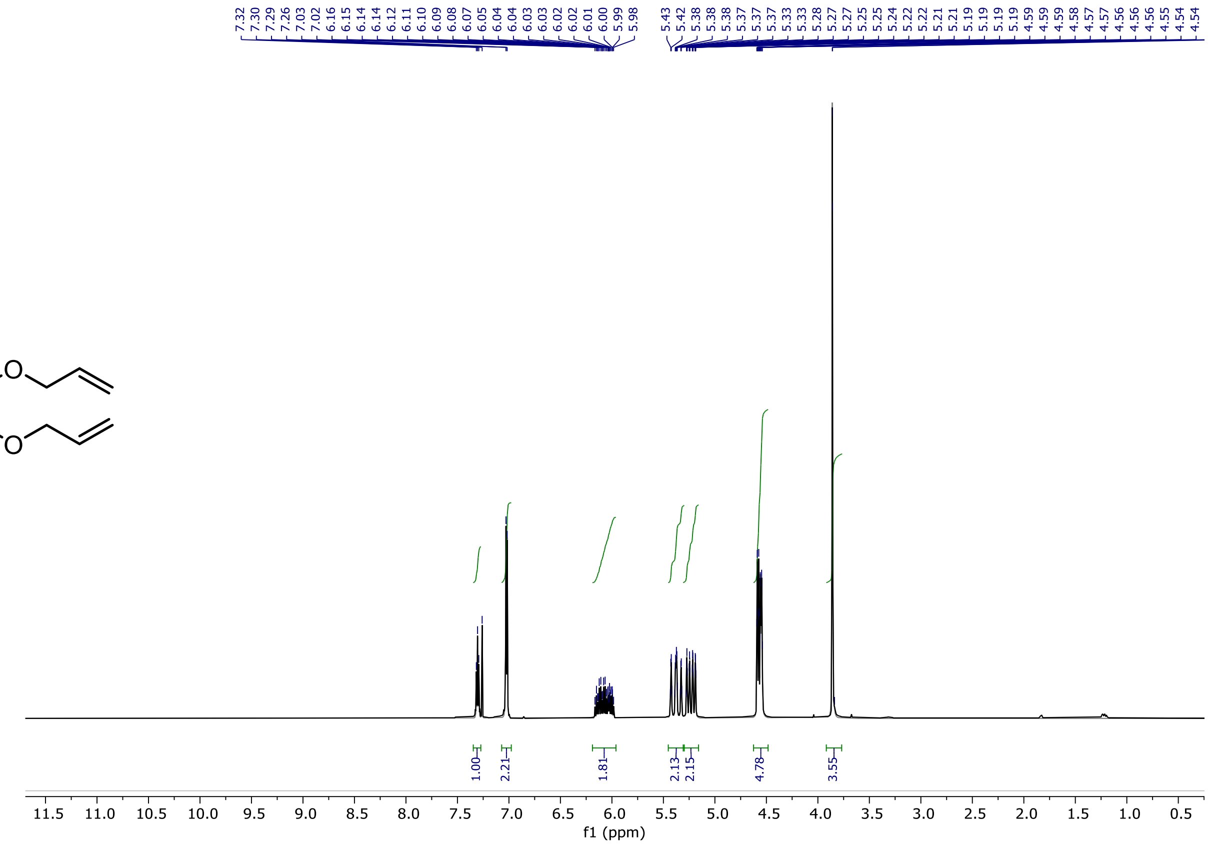

^{13}C-NMR spectrum of **15a** (101 MHz, $CDCl_3$)

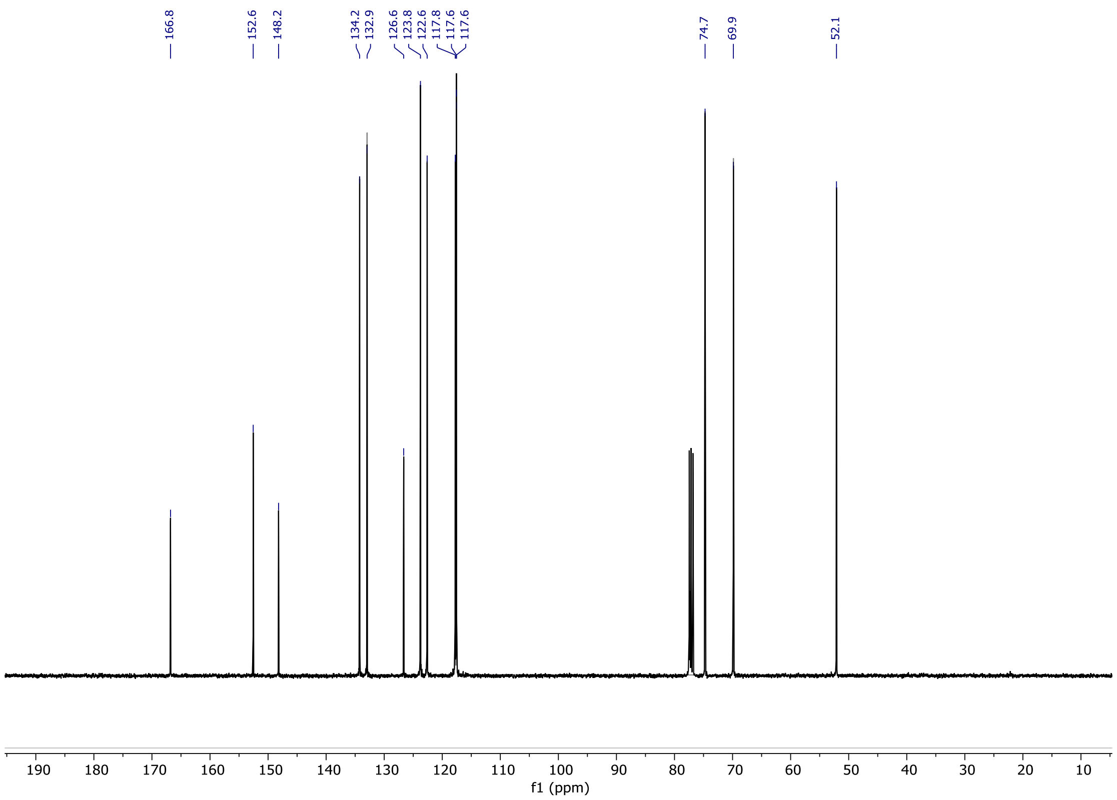

^{1}H-NMR spectrum of **15b** (400 MHz, $CDCl_3$)

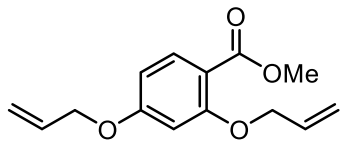

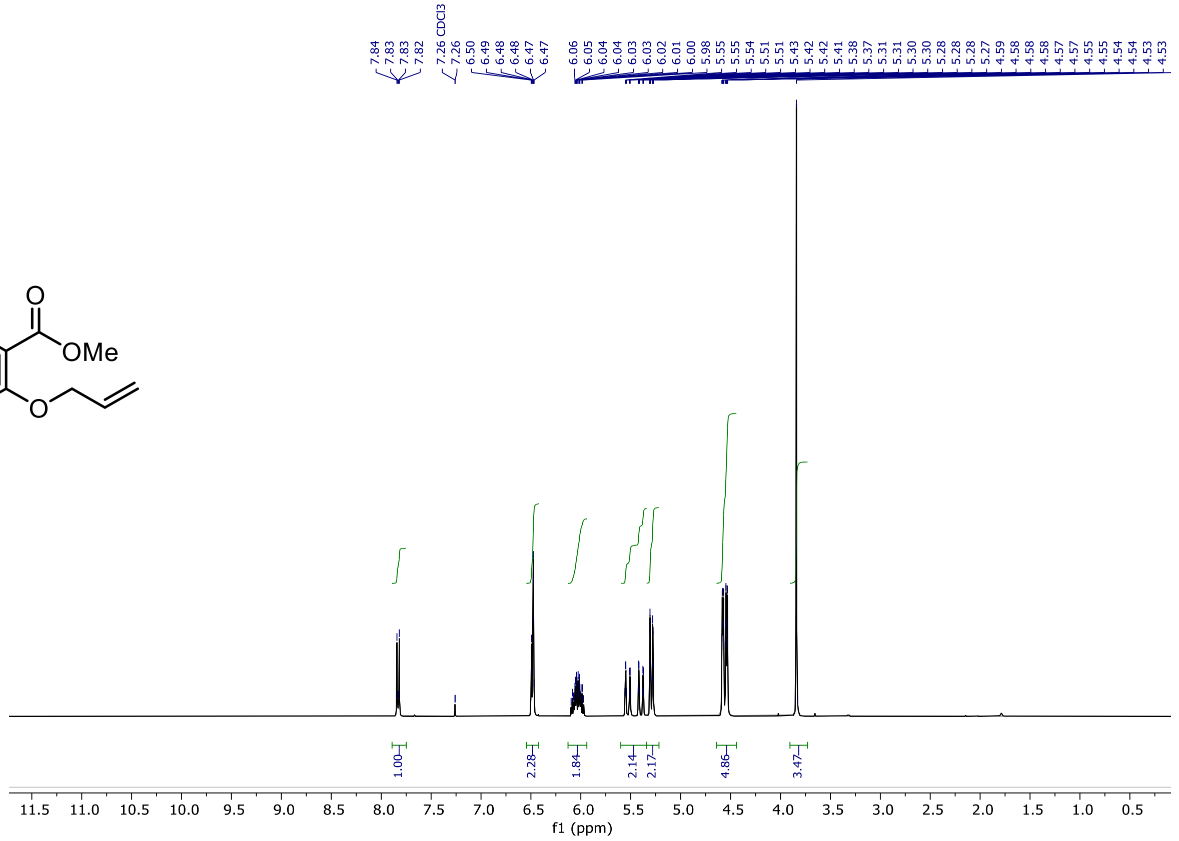

^{13}C-NMR spectrum of **15b** (101 MHz, $CDCl_3$)

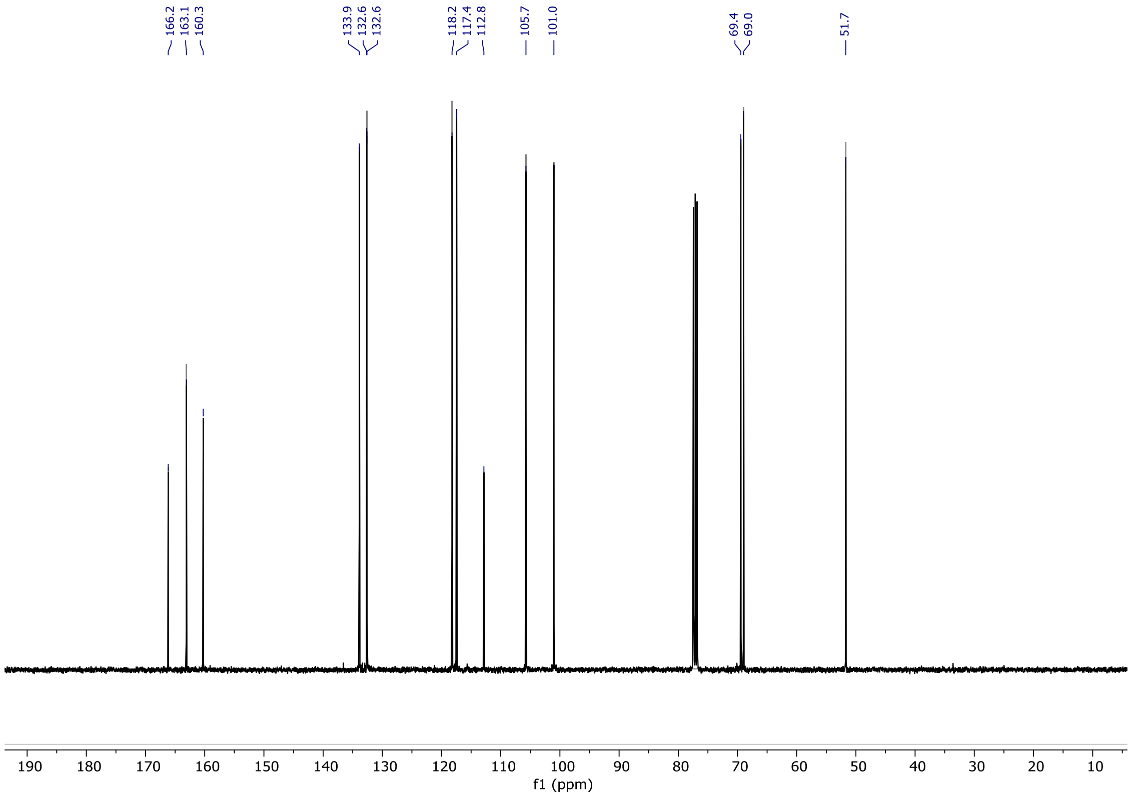

^{1}H-NMR spectrum of **15c** (400 MHz, $CDCl_3$)

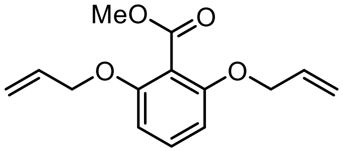

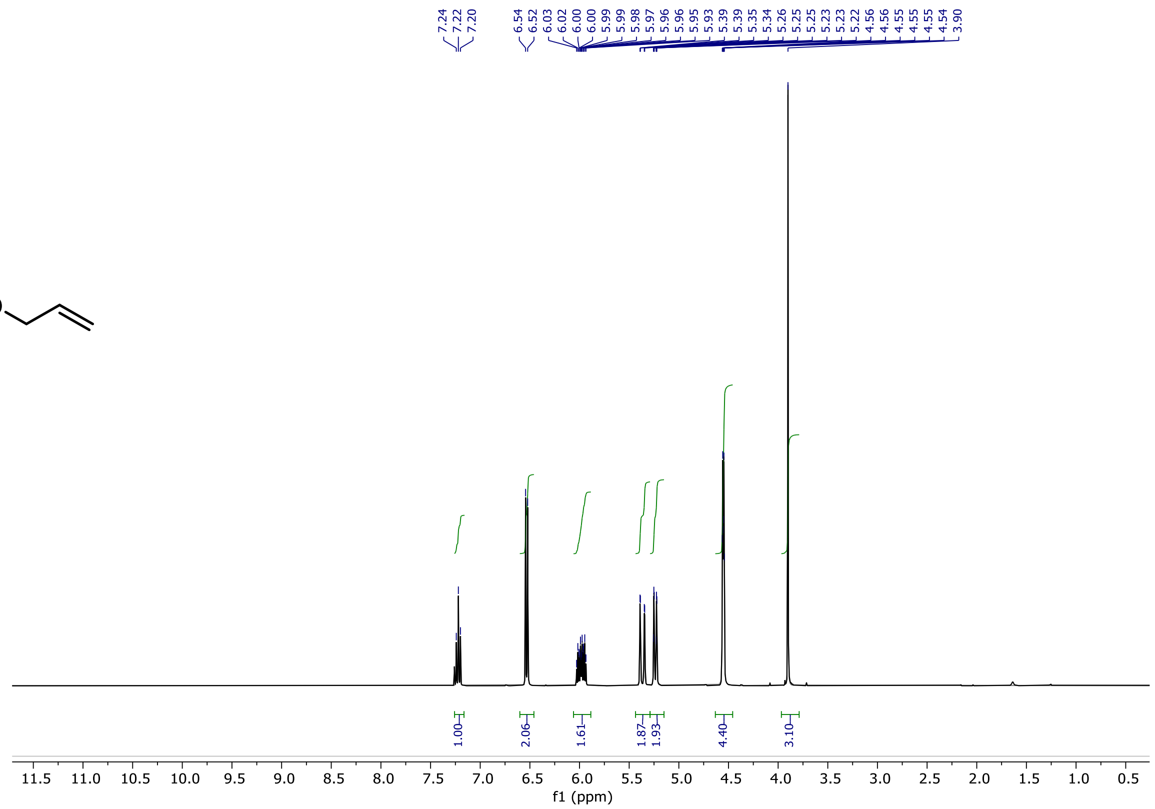

^{13}C-NMR spectrum of **15c** (101 MHz, $CDCl_3$)

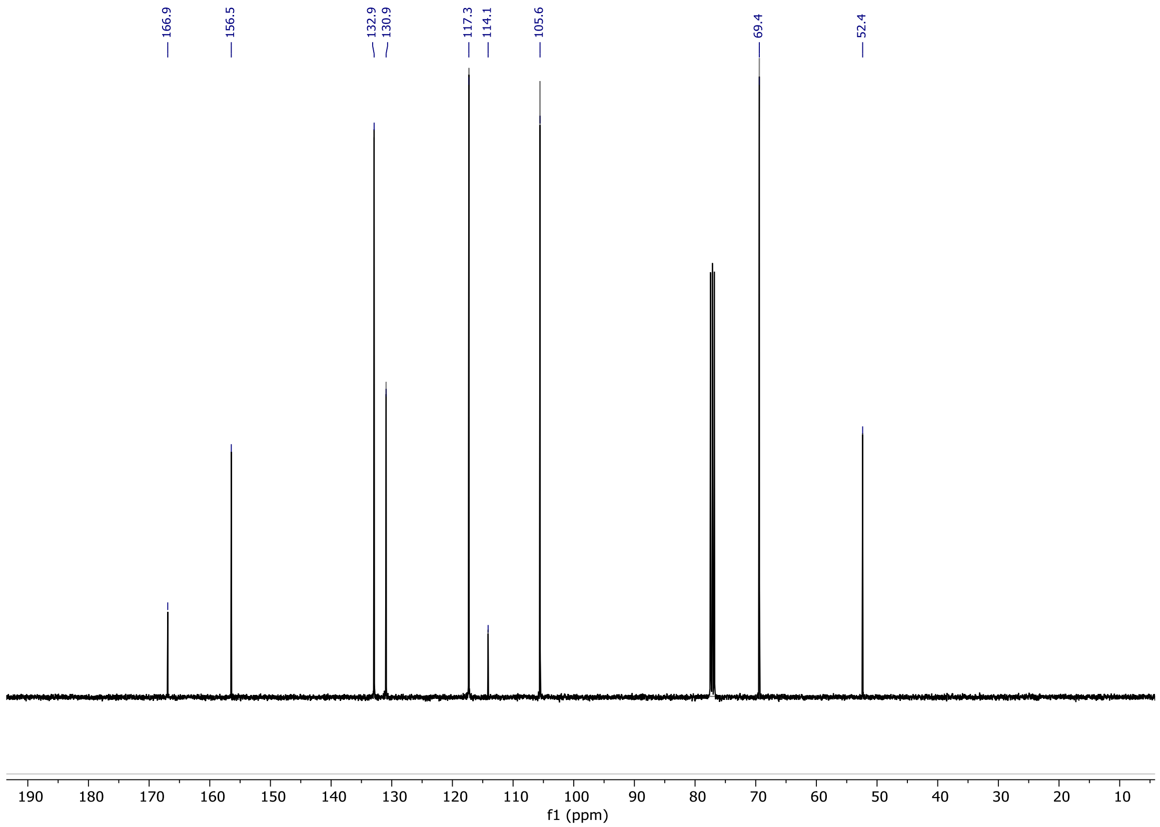

^{1}H-NMR spectrum of **15d** (400 MHz, $CDCl_3$)

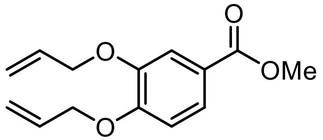

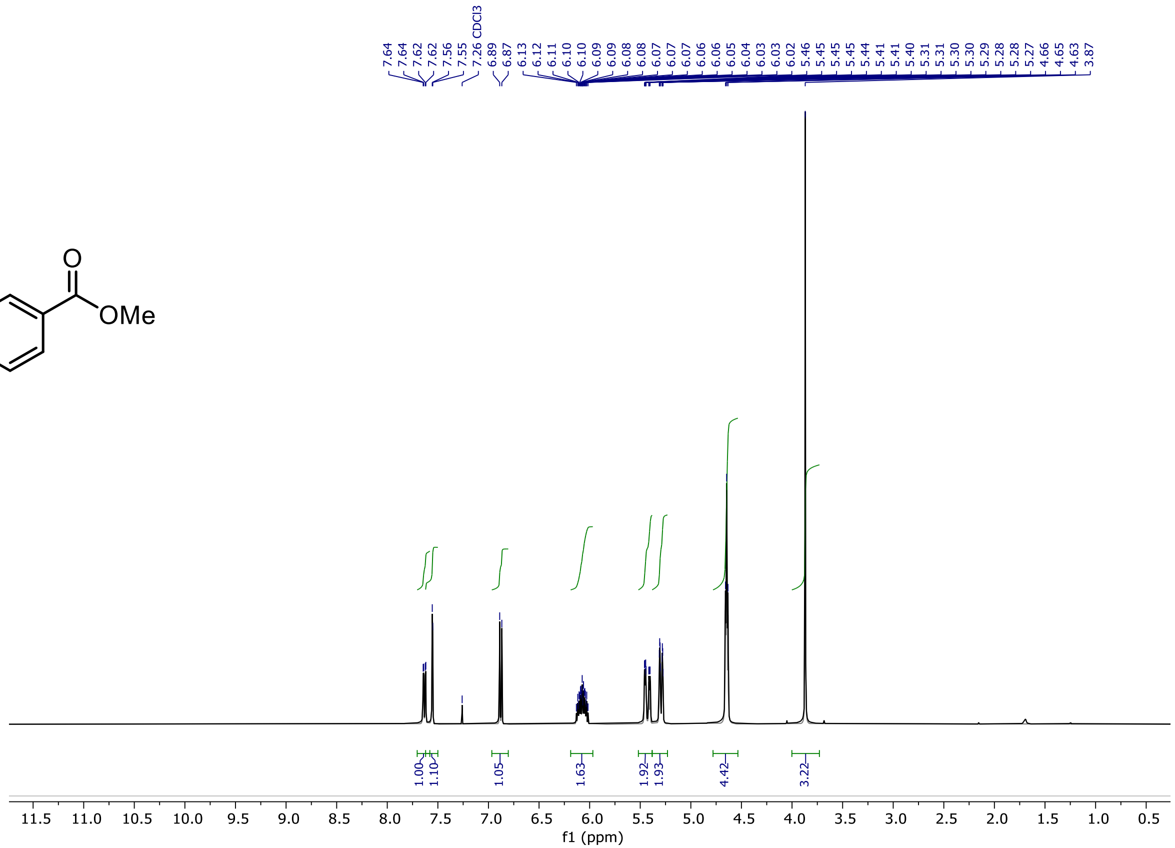

^{13}C-NMR spectrum of **15d** (101 MHz, $CDCl_3$)

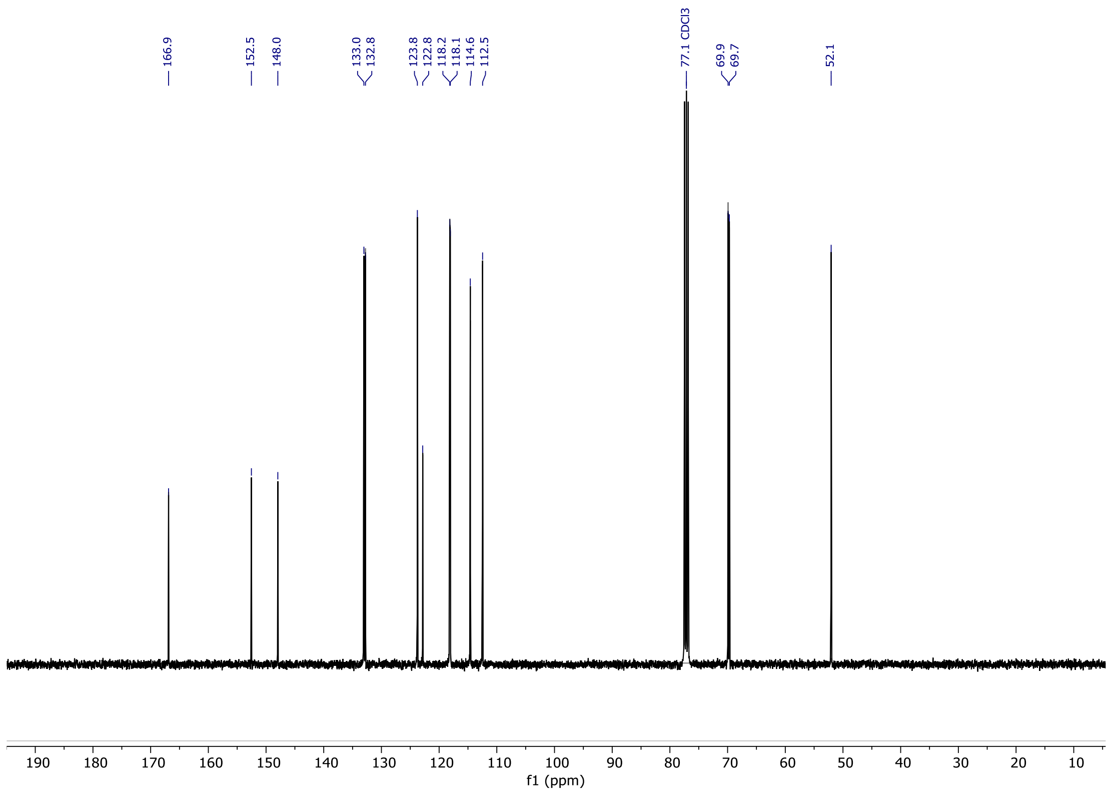

^{1}H-NMR spectrum of **15e** (400 MHz, $(CD_3)_2CO$)

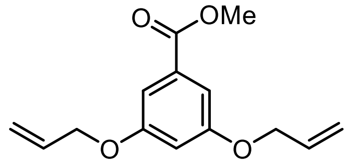

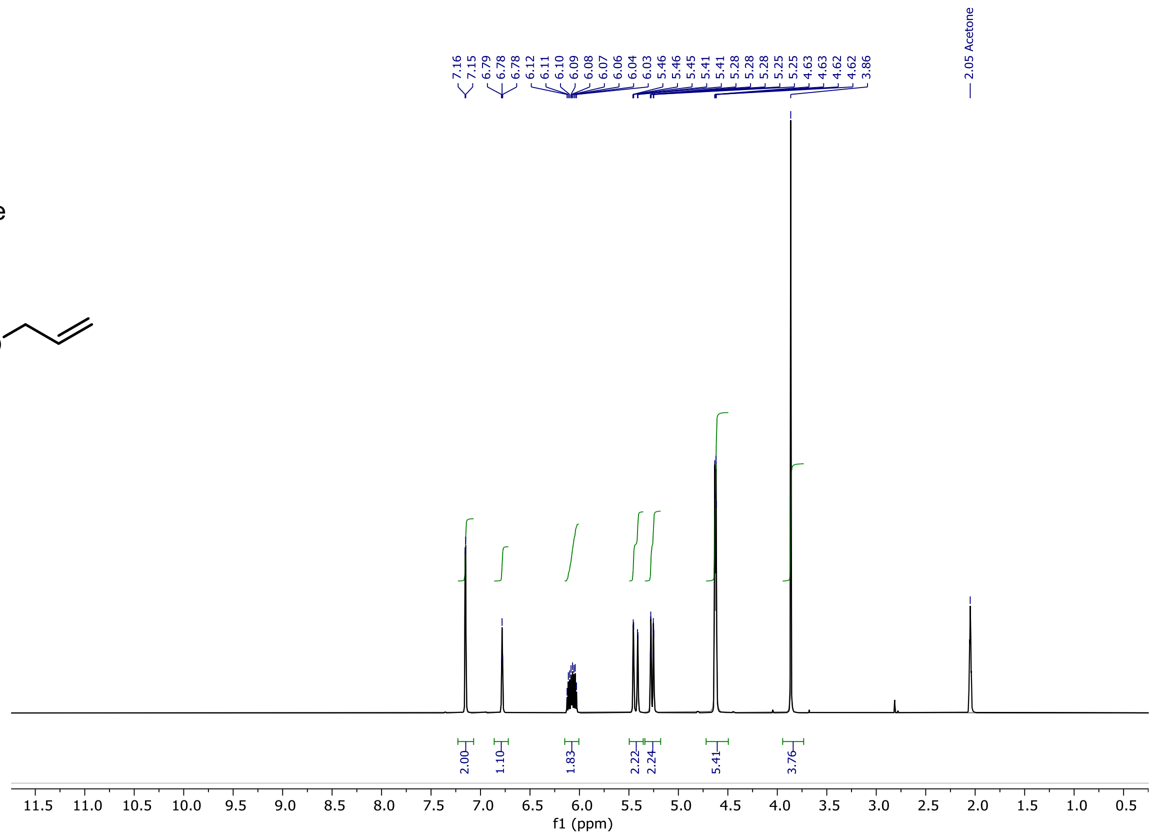

^{13}C-NMR spectrum of **15e** (101 MHz, $(CD_3)_2CO$)

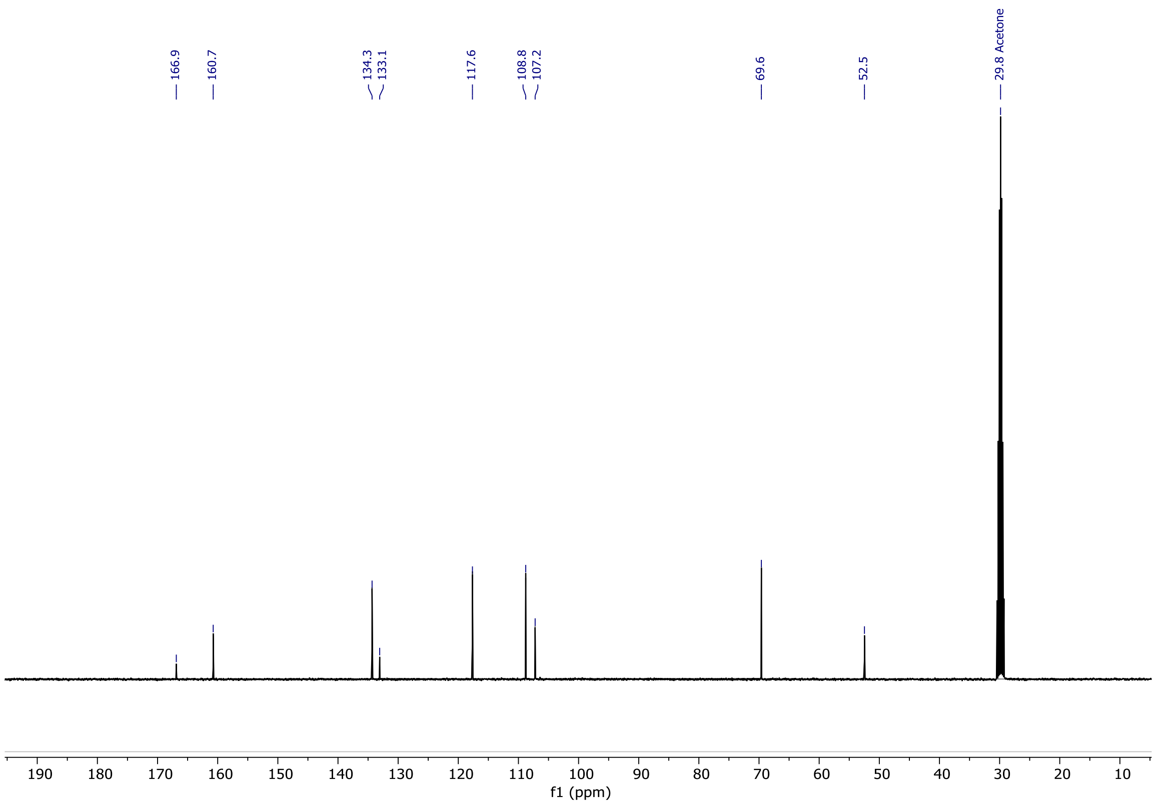

^{1}H-NMR spectrum of **15f** (400 MHz, $CDCl_3$)

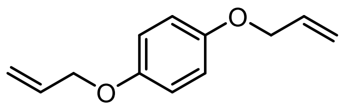

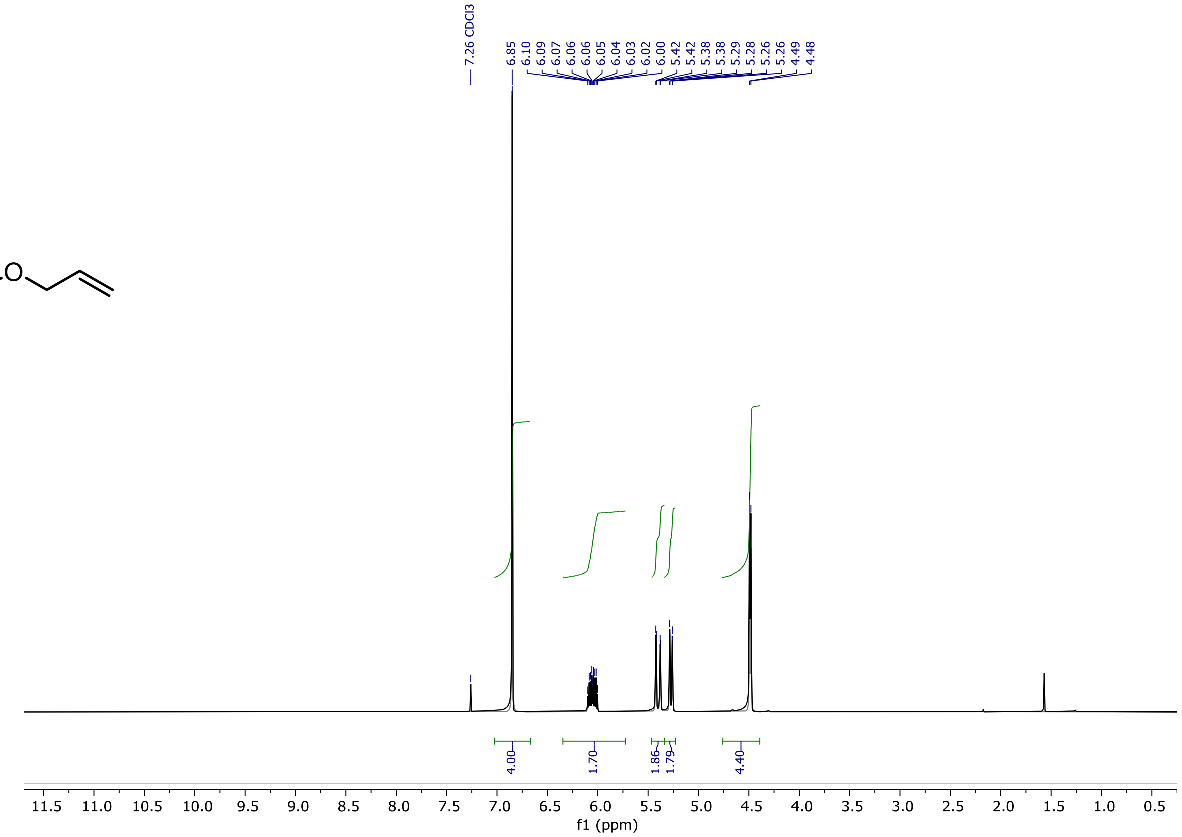

^{13}C-NMR spectrum of **15f** (101 MHz, $CDCl_3$)

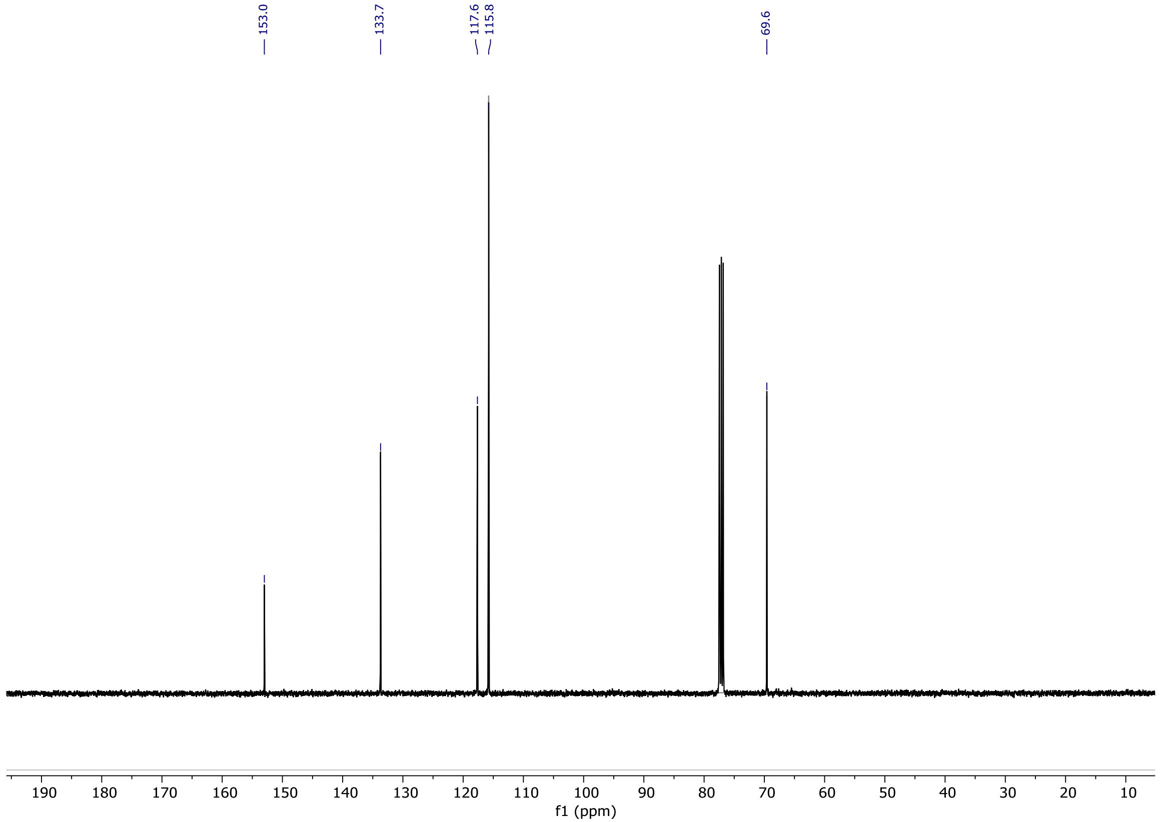

^{1}H-NMR spectrum of **15g** ($CDCl_3$, 400 MHz)

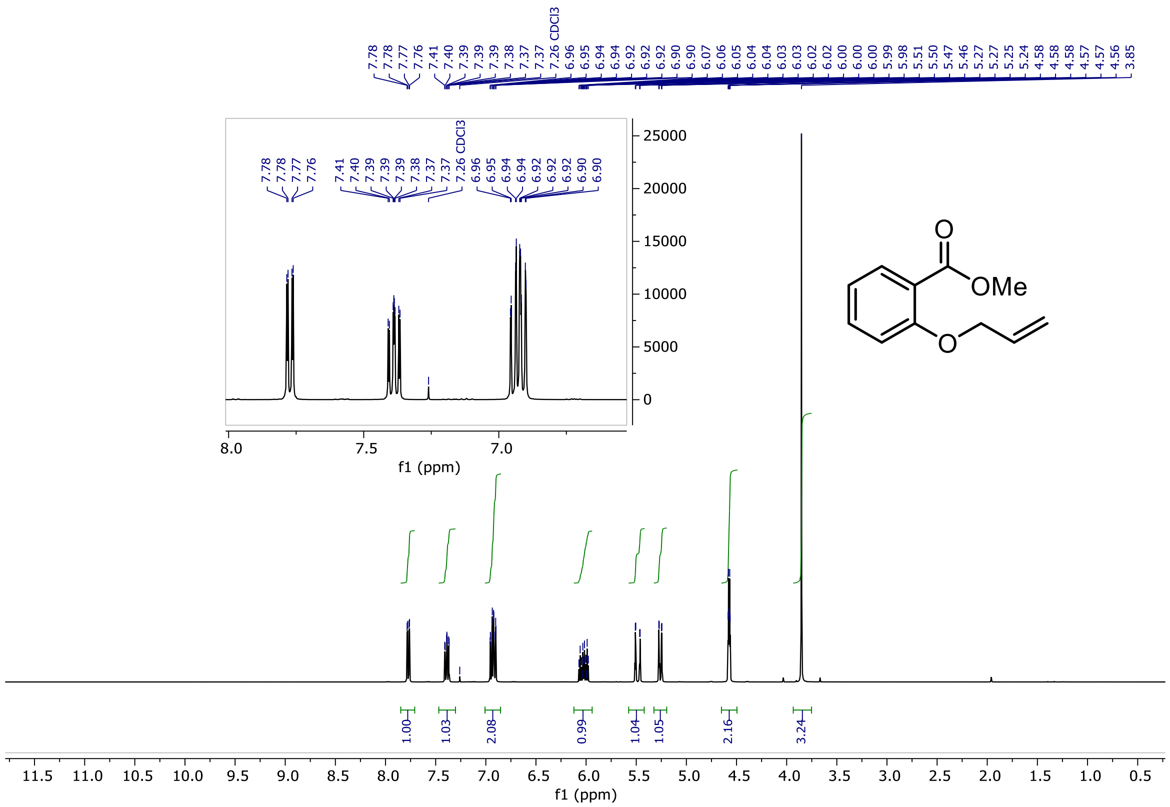

^{13}C-NMR spectrum of **15g** ($CDCl_3$, 101 MHz)

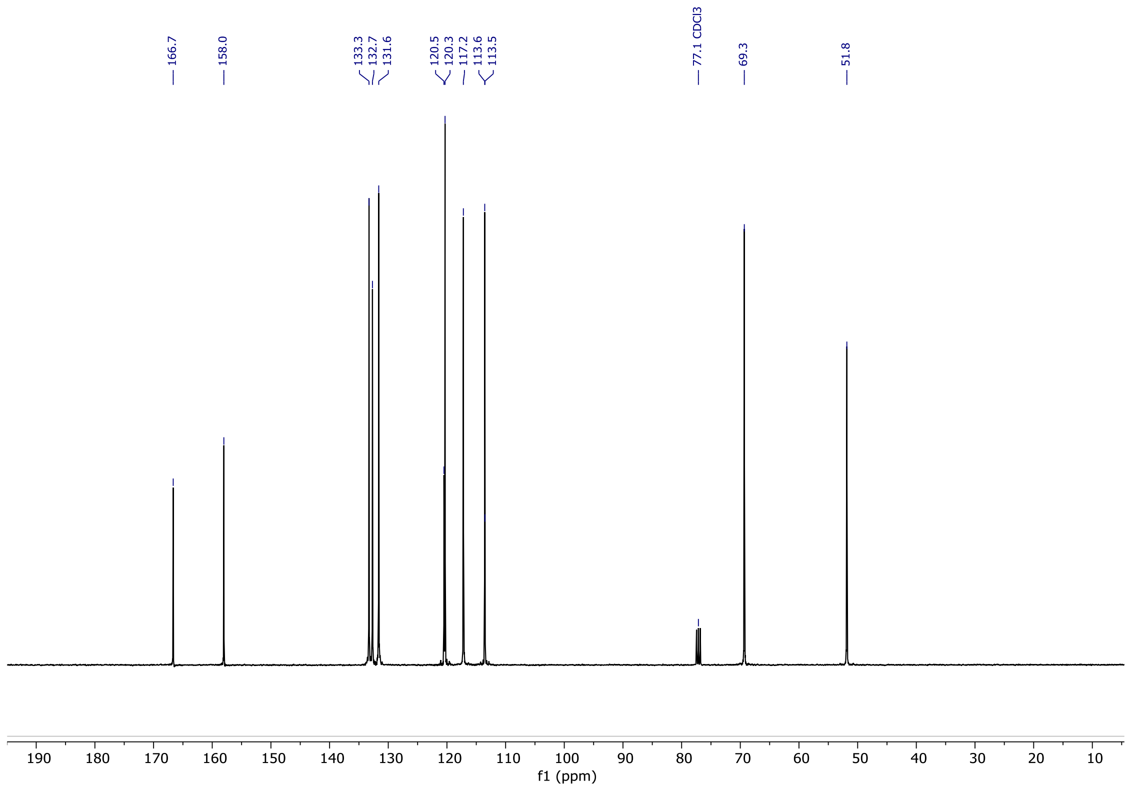

^{1}H-NMR spectrum of **15h** ($CDCl_3$, 400 MHz)

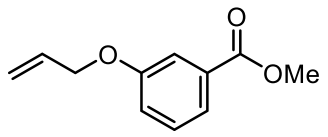

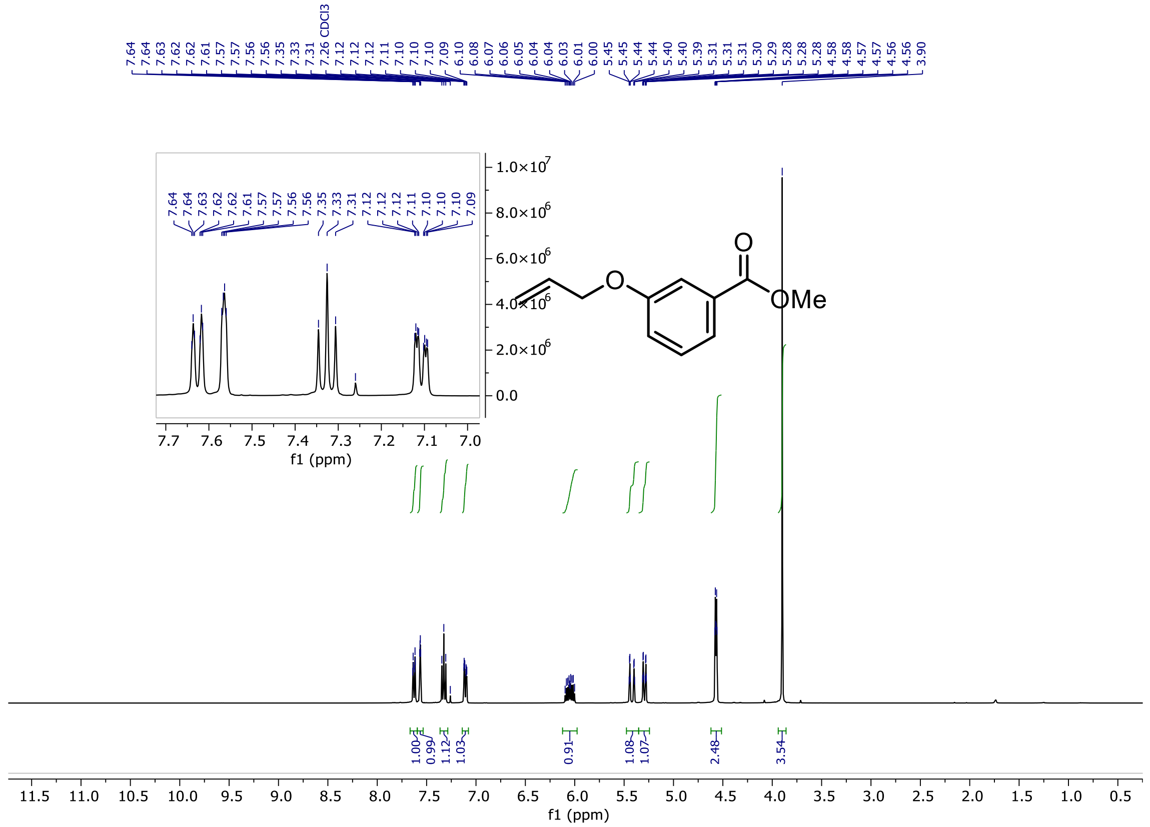

^{13}C-NMR spectrum of **15h** ($CDCl_3$, 101 MHz)

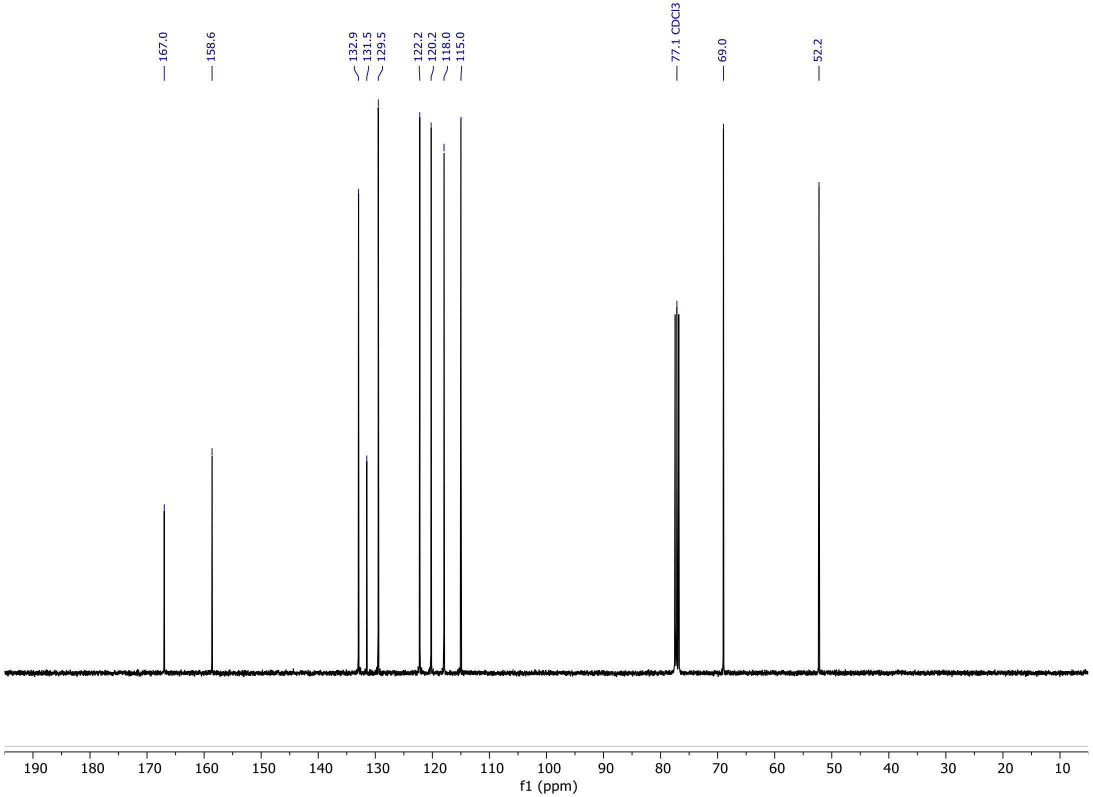

^{1}H-NMR spectrum of **15i** ($CDCl_3$, 400 MHz)

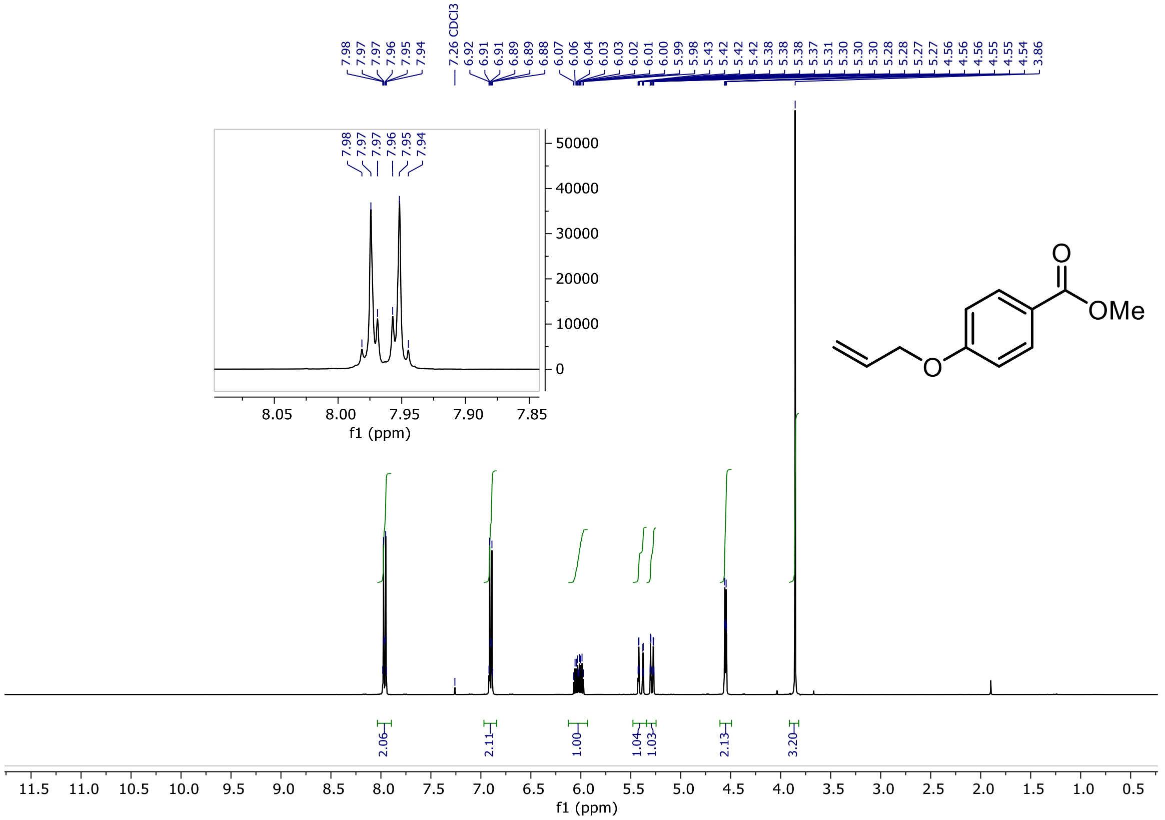

^{13}C-NMR spectrum of **15i** ($CDCl_3$, 101 MHz)

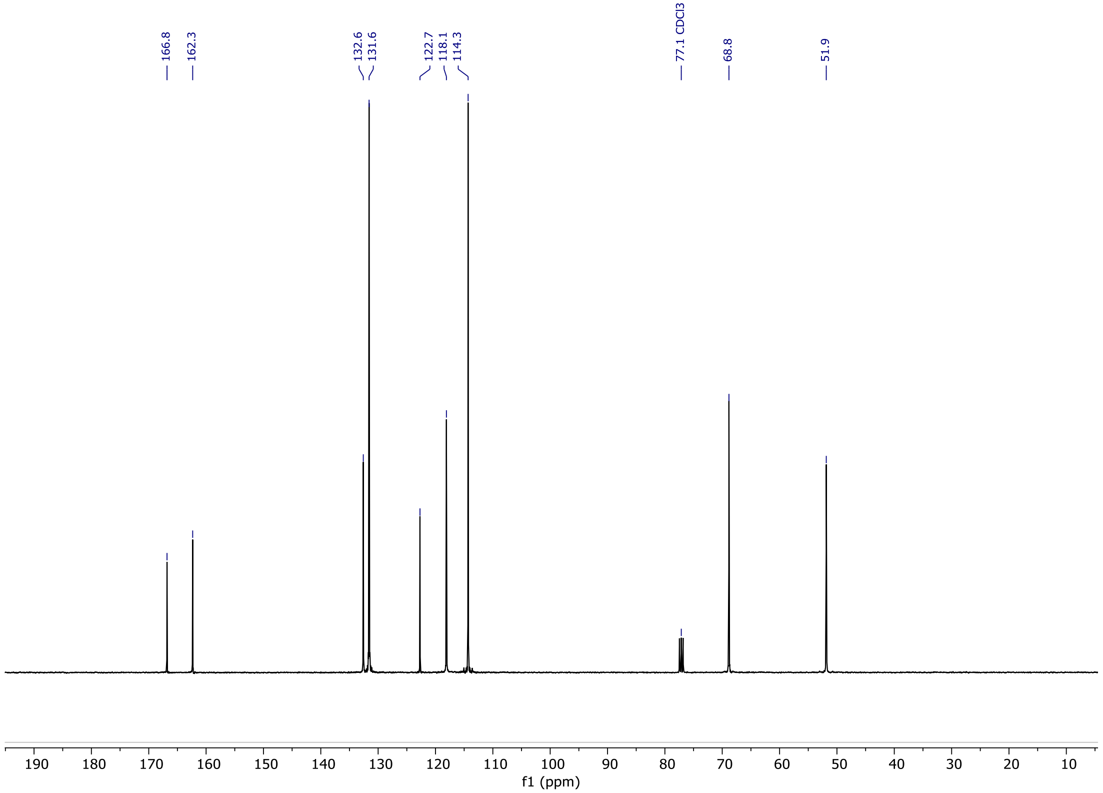

^{1}H-NMR spectrum of **16a** (400 MHz, CD_3OD)

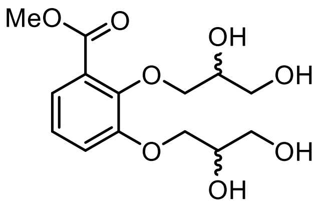

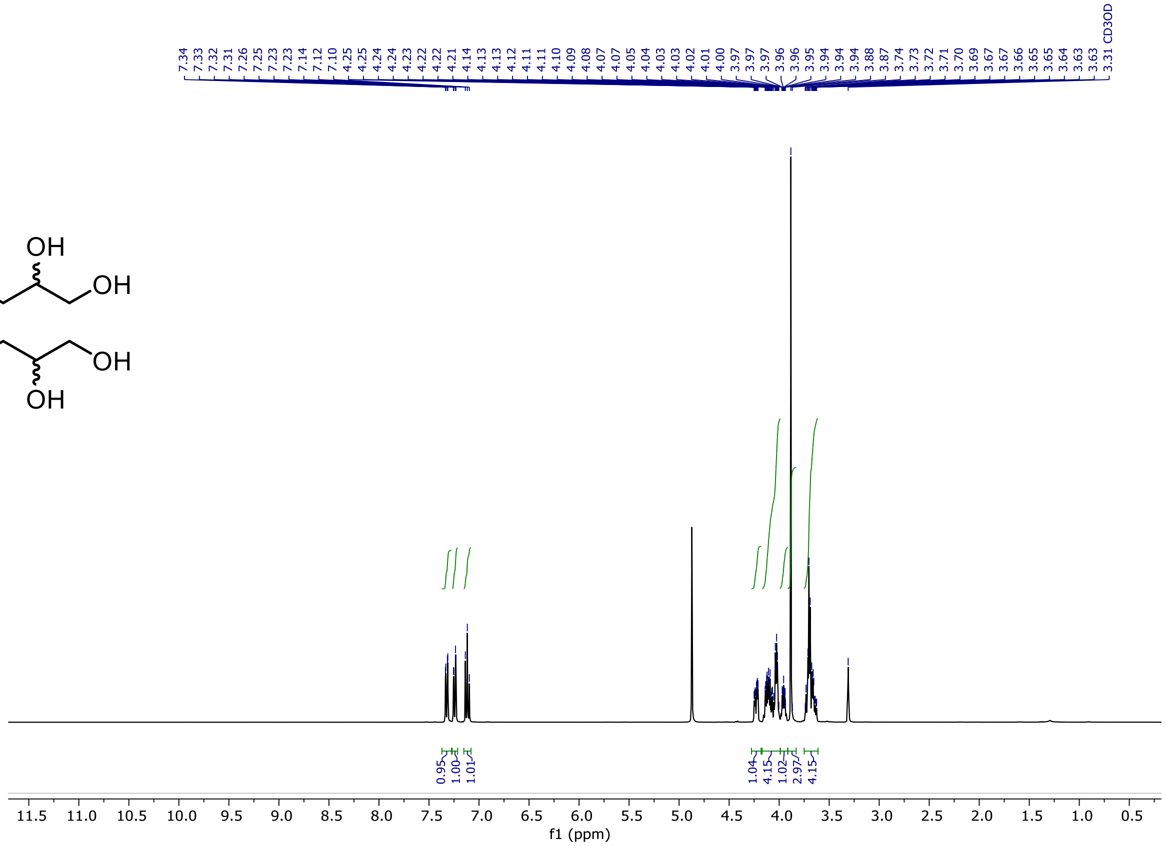

^{13}C-NMR spectrum of **16a** (101 MHz, CD_3OD)

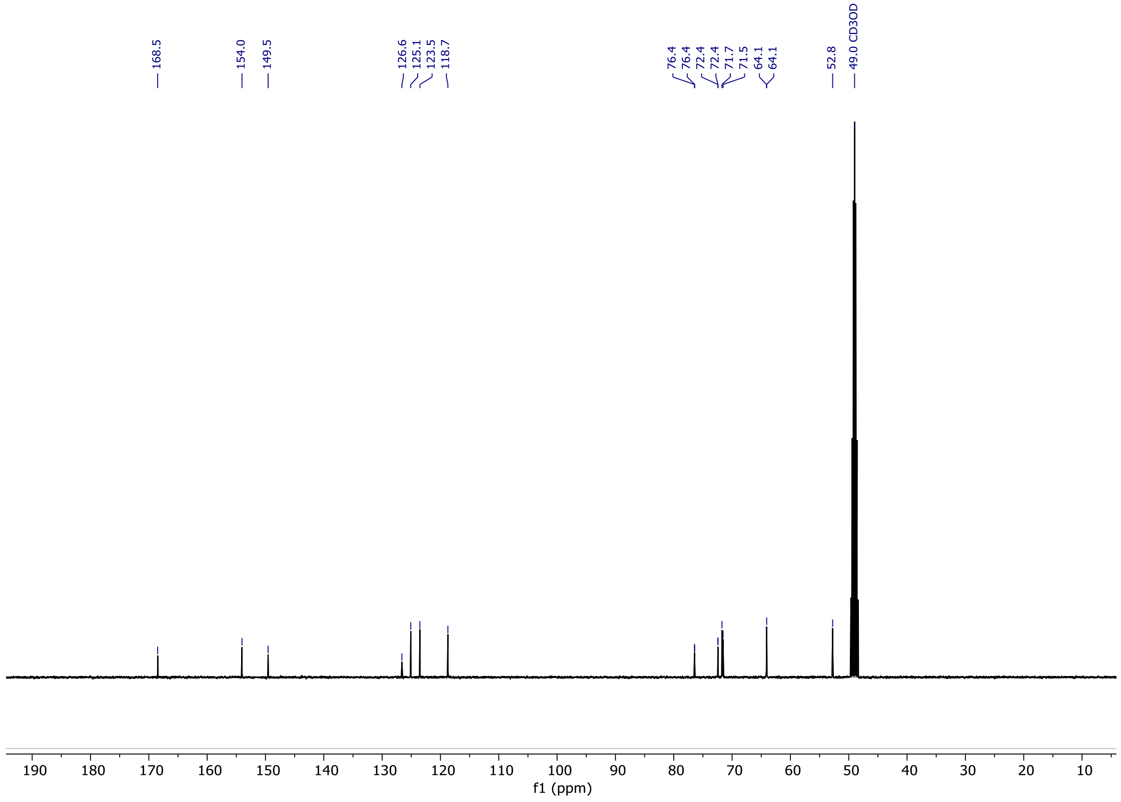

^{1}H-NMR spectrum of **16b** (400 MHz, CD_3OD)

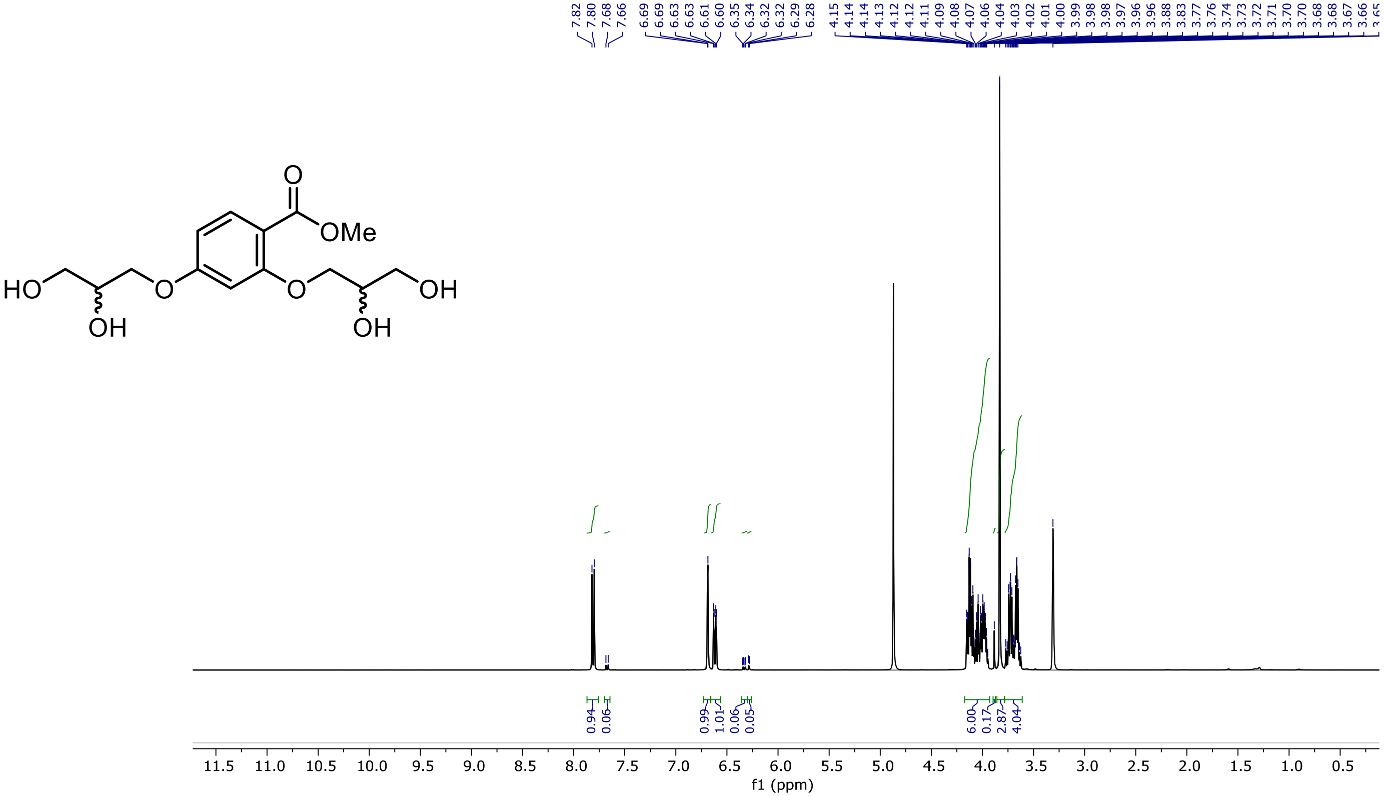

^{13}C-NMR spectrum of **16b** (101 MHz, CD_3OD)

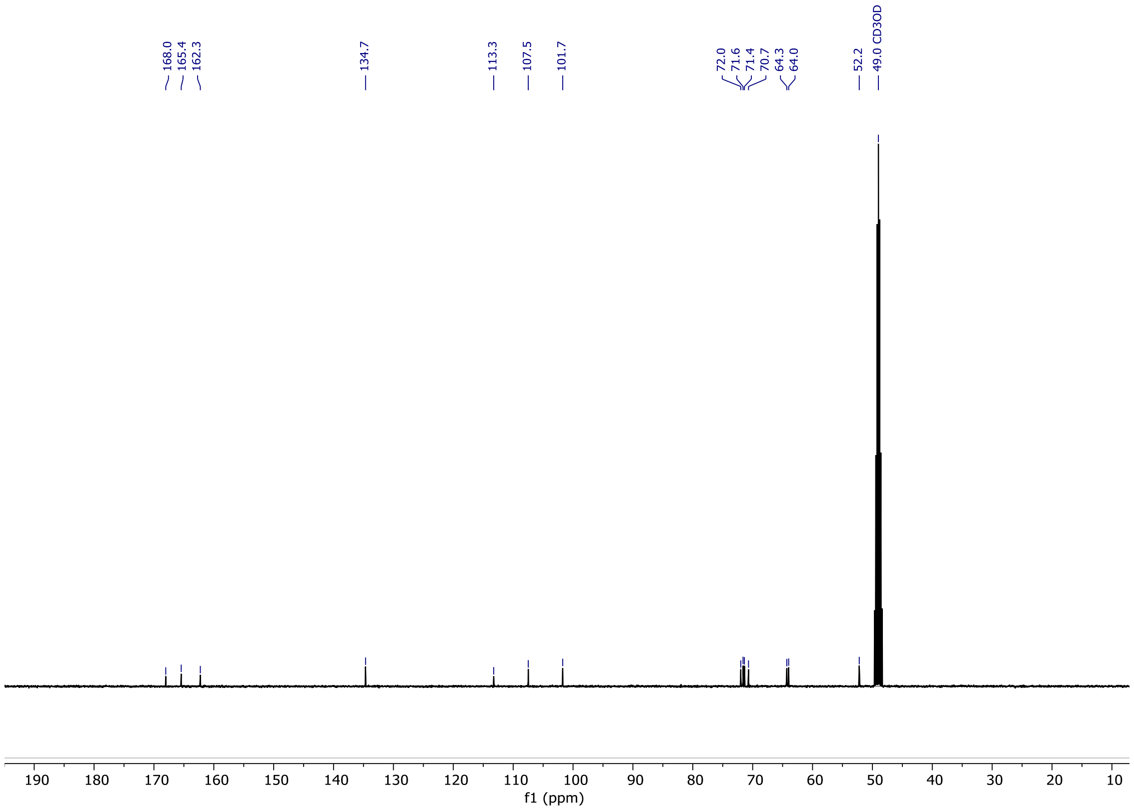

^{1}H-NMR spectrum of **16c** (400 MHz, CD_3OD)

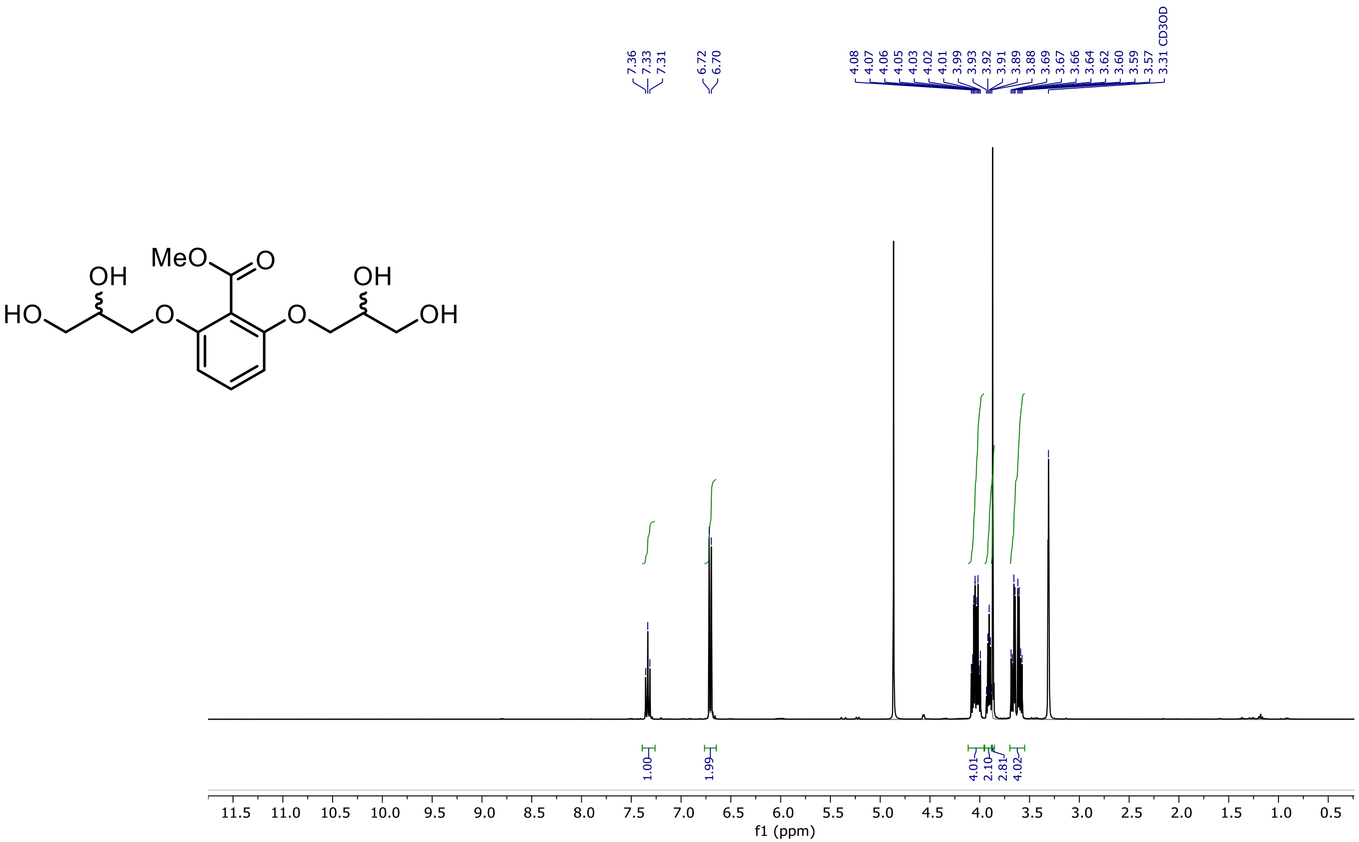

^{13}C-NMR spectrum of **16c** (101 MHz, CD_3OD)

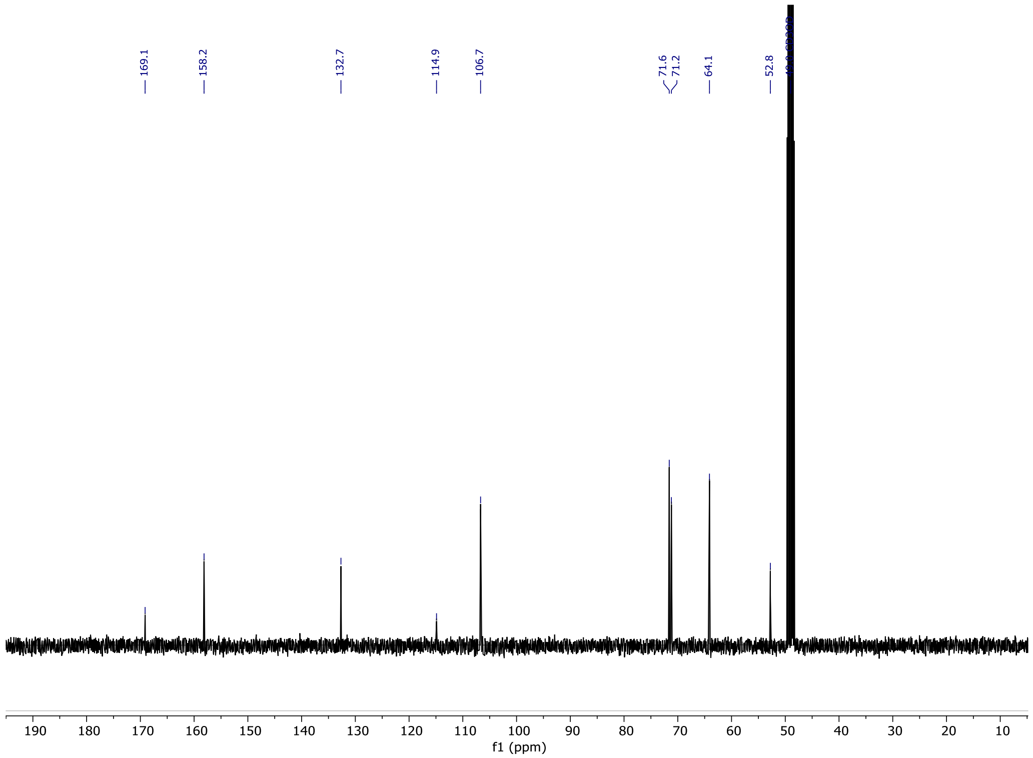

^{1}H-NMR spectrum of **16d** (400 MHz, CD_3OD)

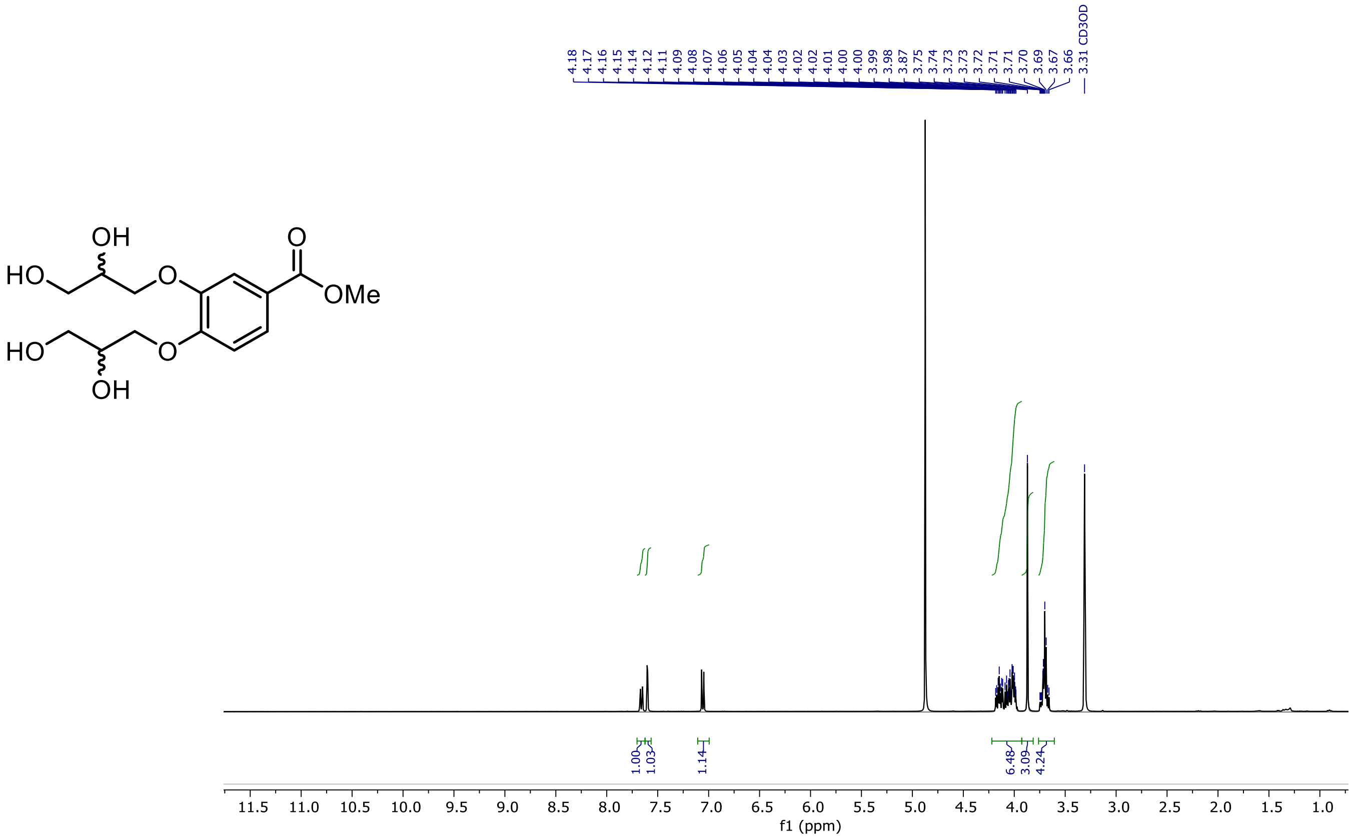

^{13}C-NMR spectrum of **16d** (101 MHz, CD_3OD)

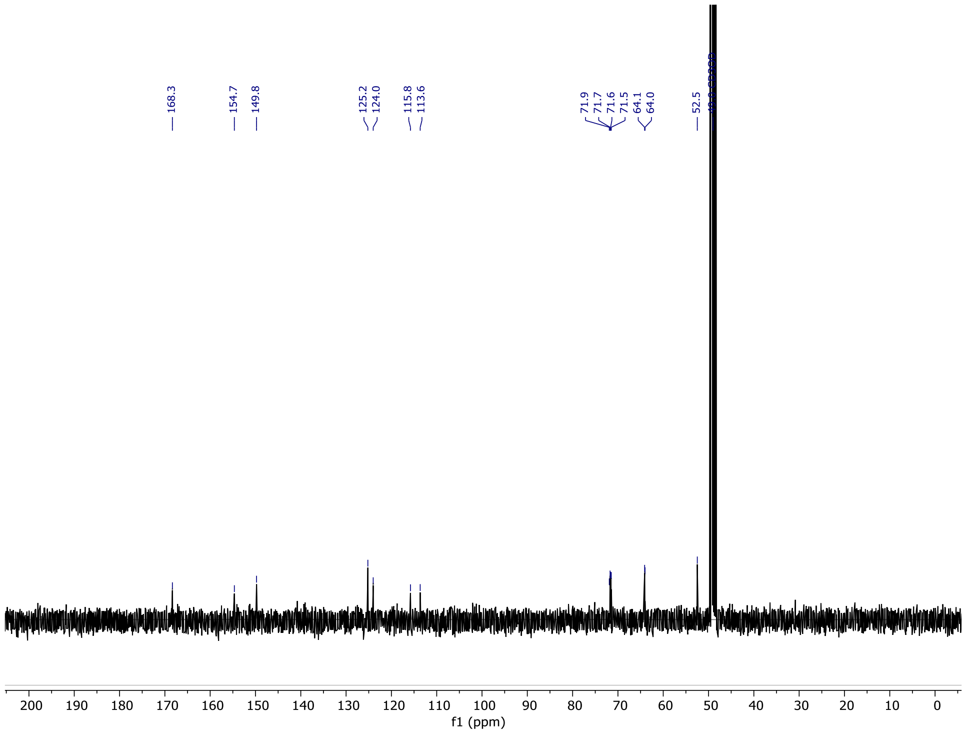

^{1}H-NMR spectrum of **16e** (400 MHz, CD_3OD)

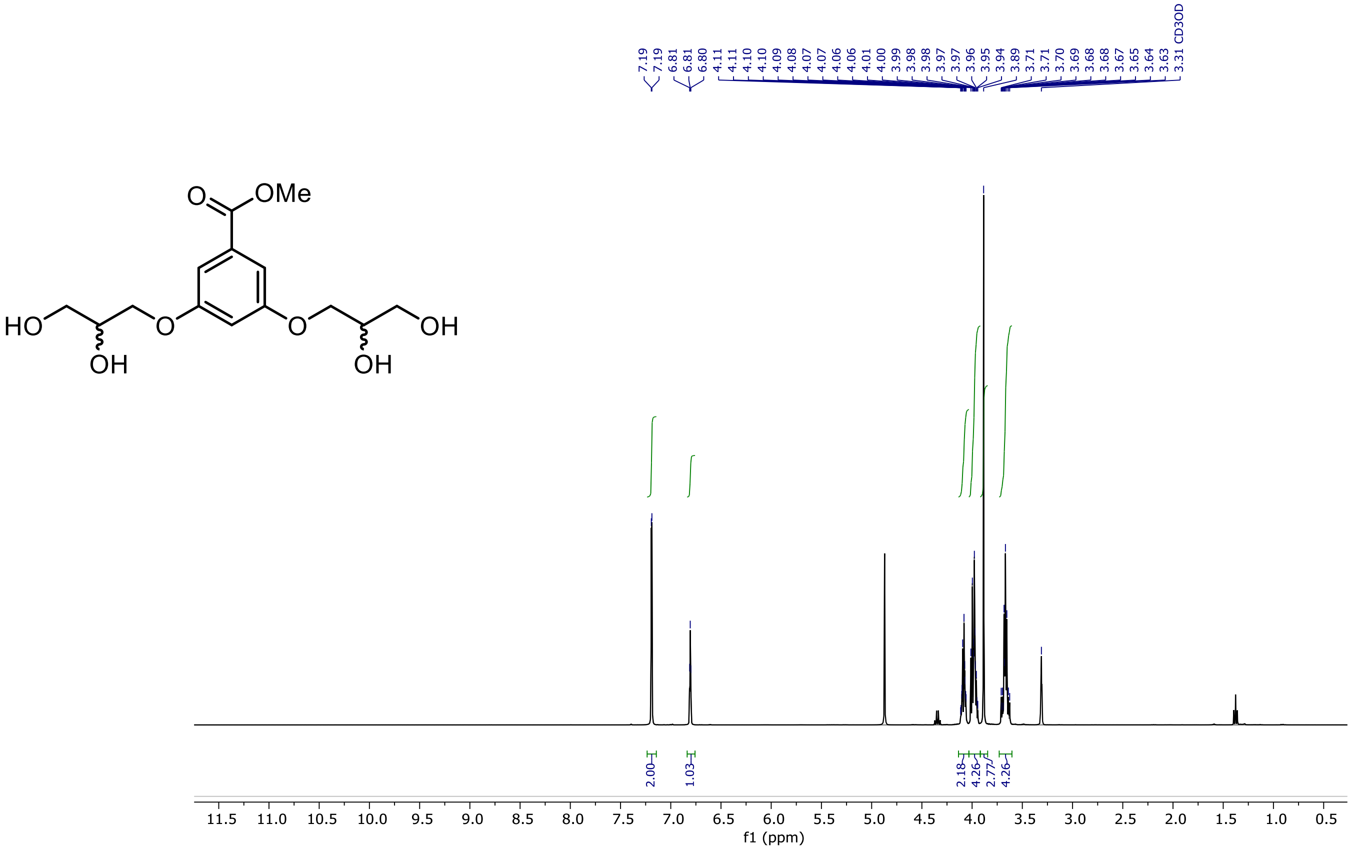

^{13}C-NMR spectrum of **16e** (101 MHz, CD_3OD)

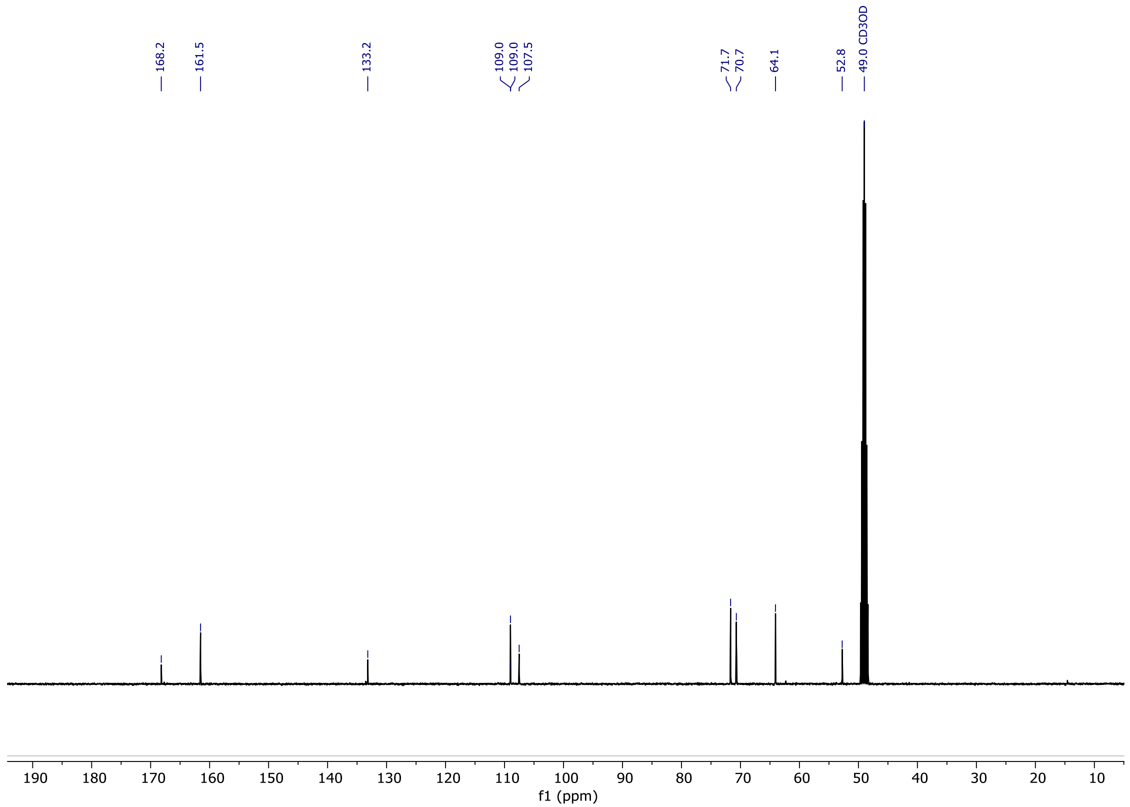

^{1}H-NMR spectrum of **16f** (400 MHz, CD_3OD)

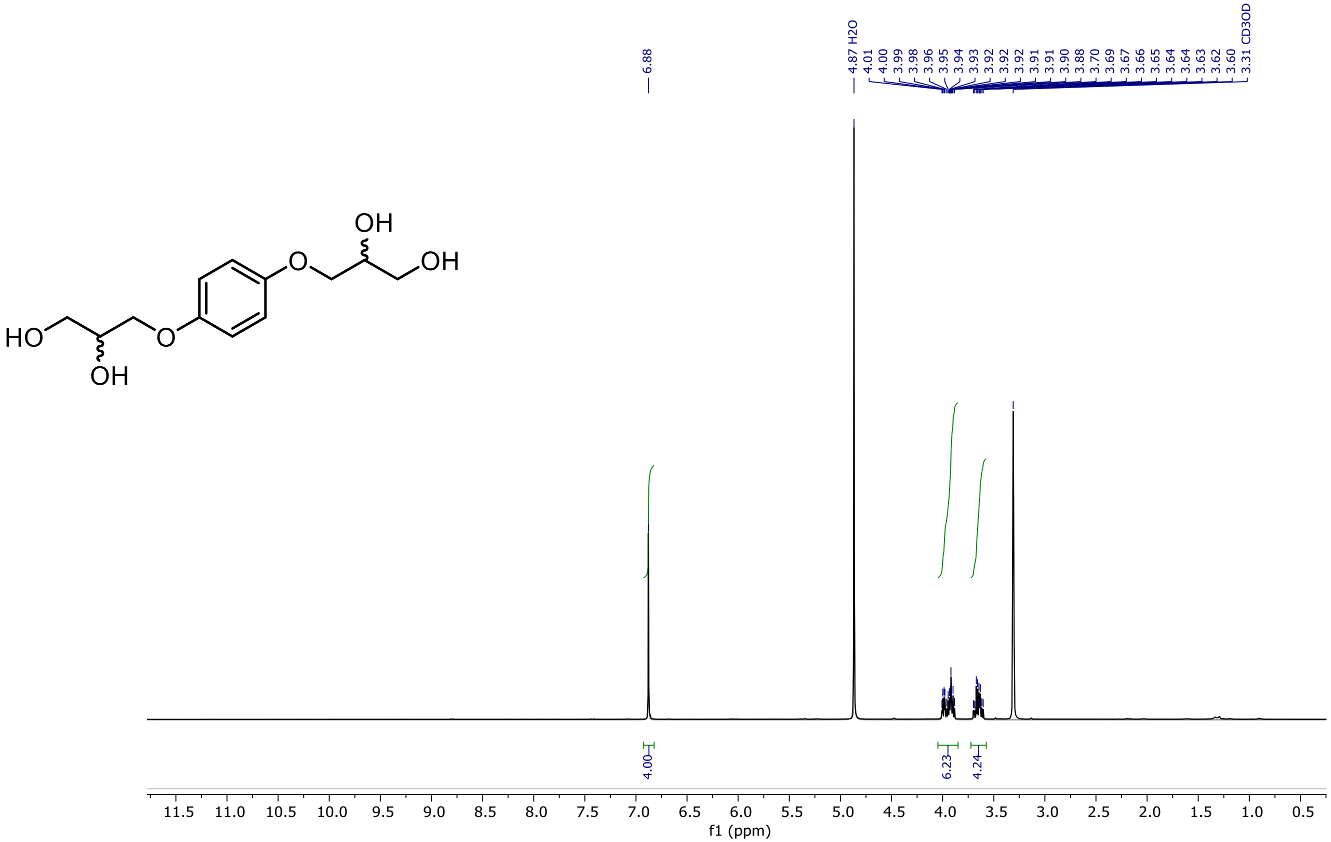

^{13}C-NMR spectrum of **16f** (101 MHz, CD_3OD)

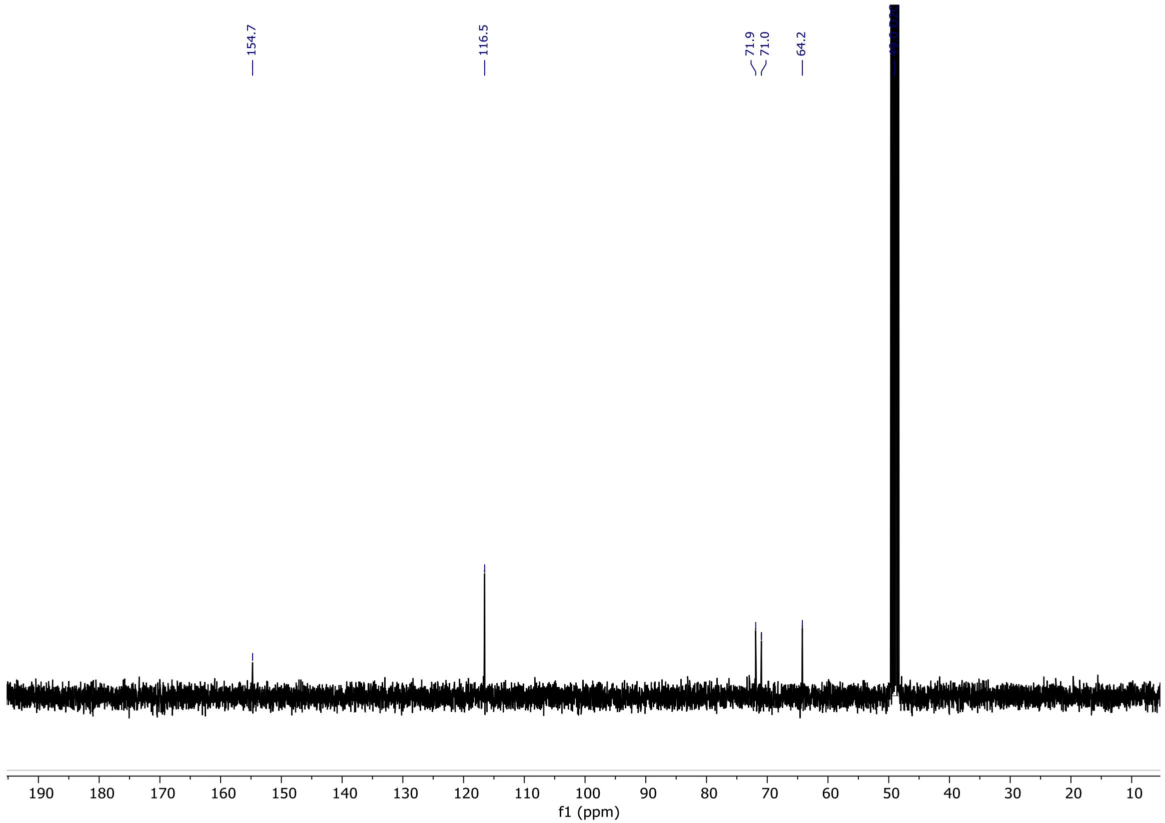

^{1}H-NMR spectrum of **16g** ($(CD_3)_2SO$, 400 MHz)

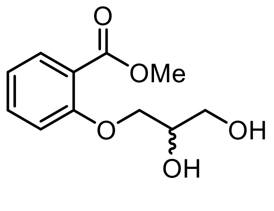

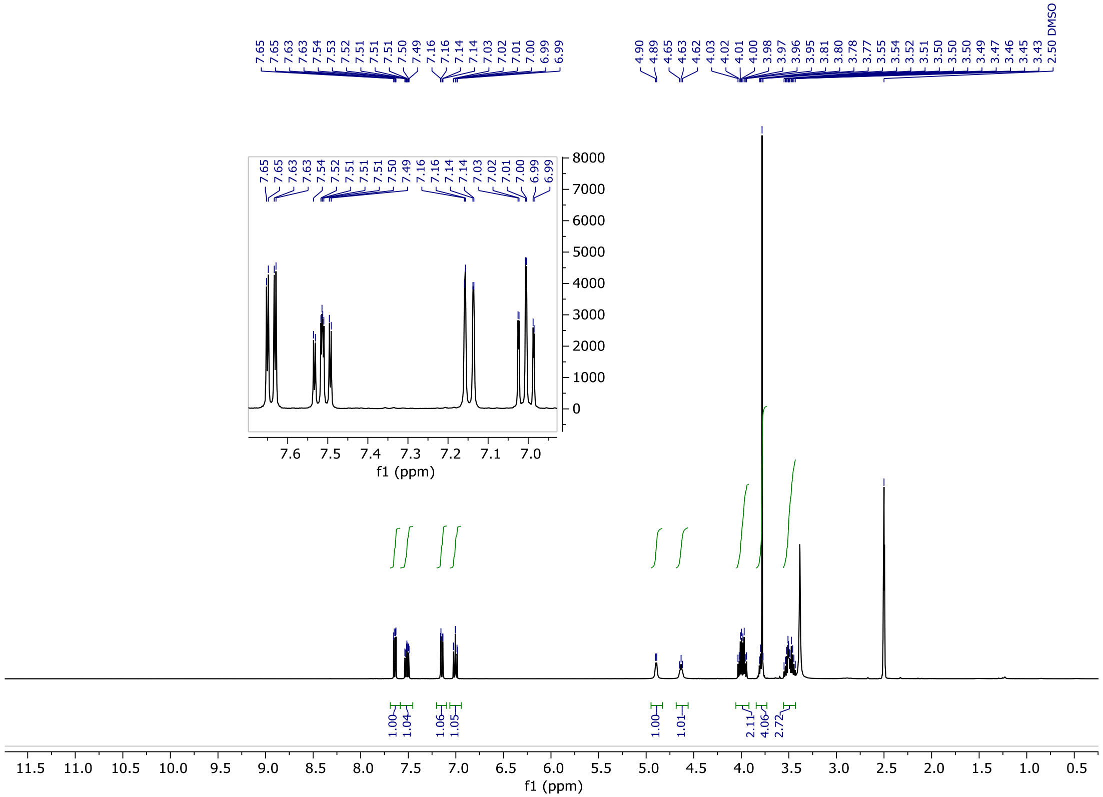

^{13}C-NMR spectrum of **16g** ($(CD_3)_2SO$, 101 MHz)

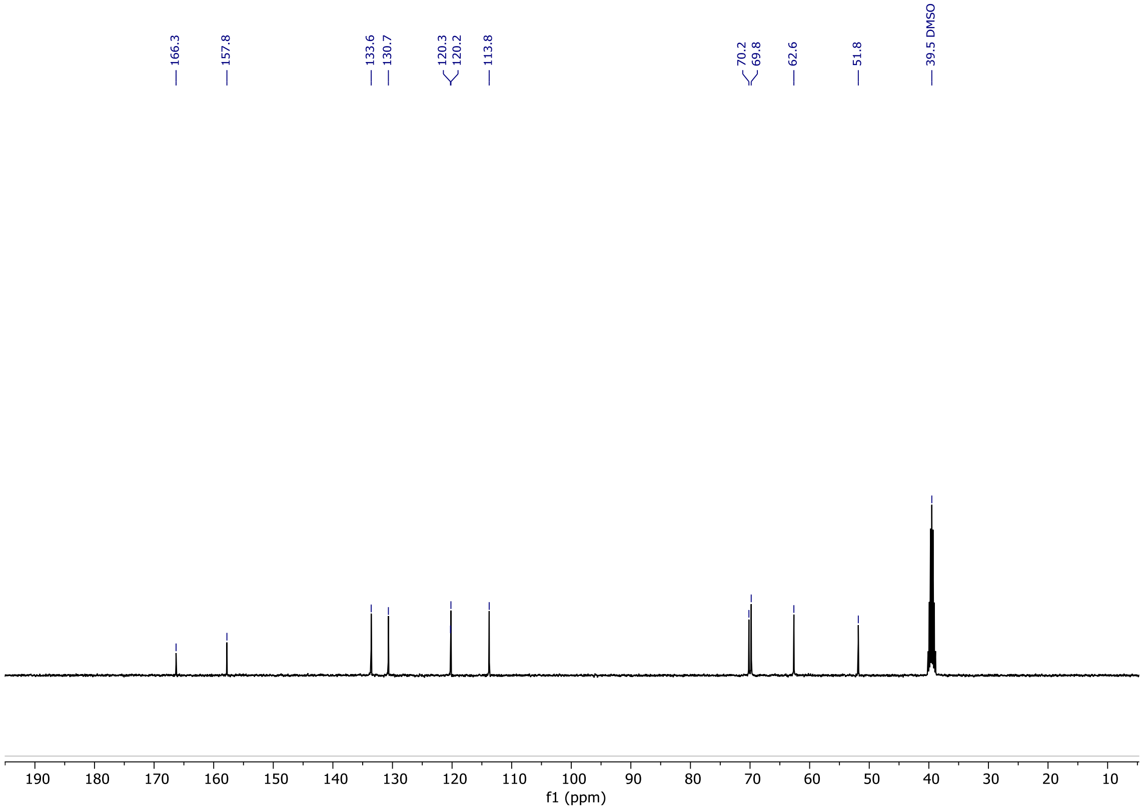

^{1}H-NMR spectrum of **16h** ($(CD_3)_2SO$, 400 MHz)

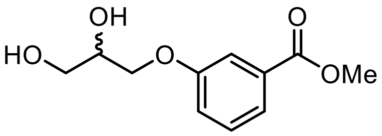

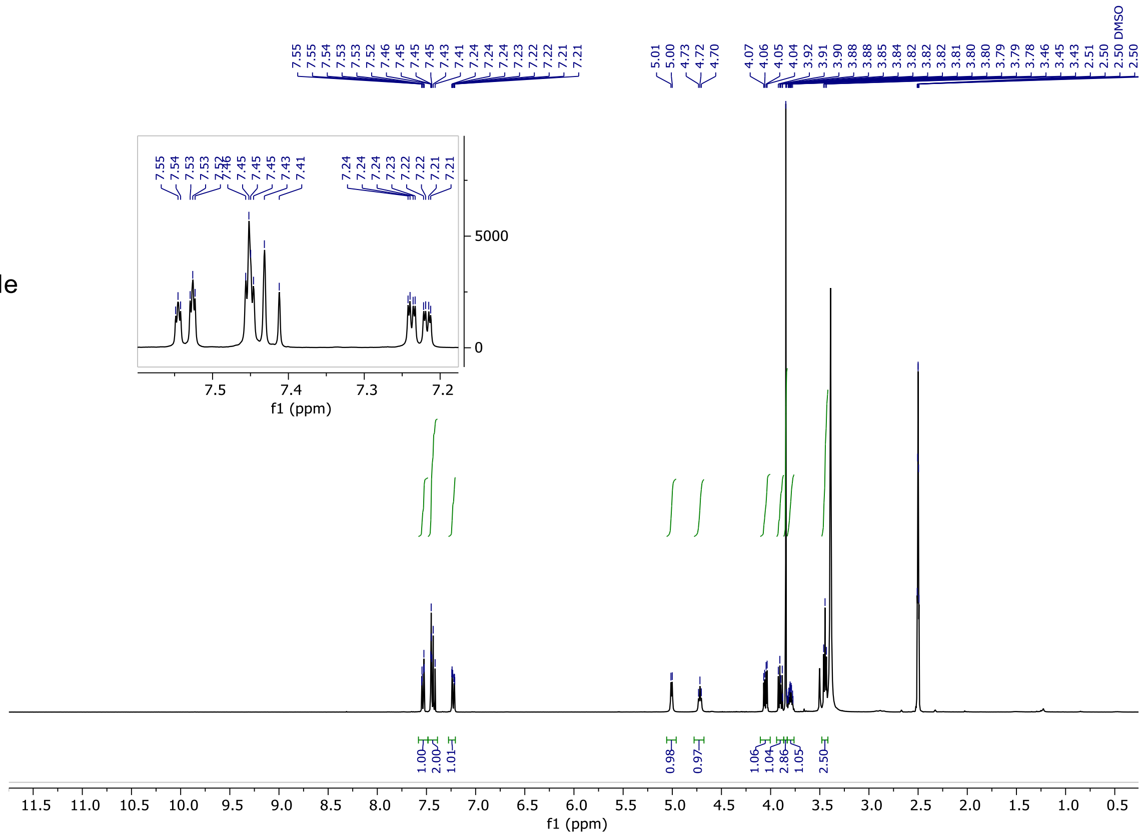

^{13}C-NMR spectrum of **16h** $(CD_3)_2SO$, 101 MHz)

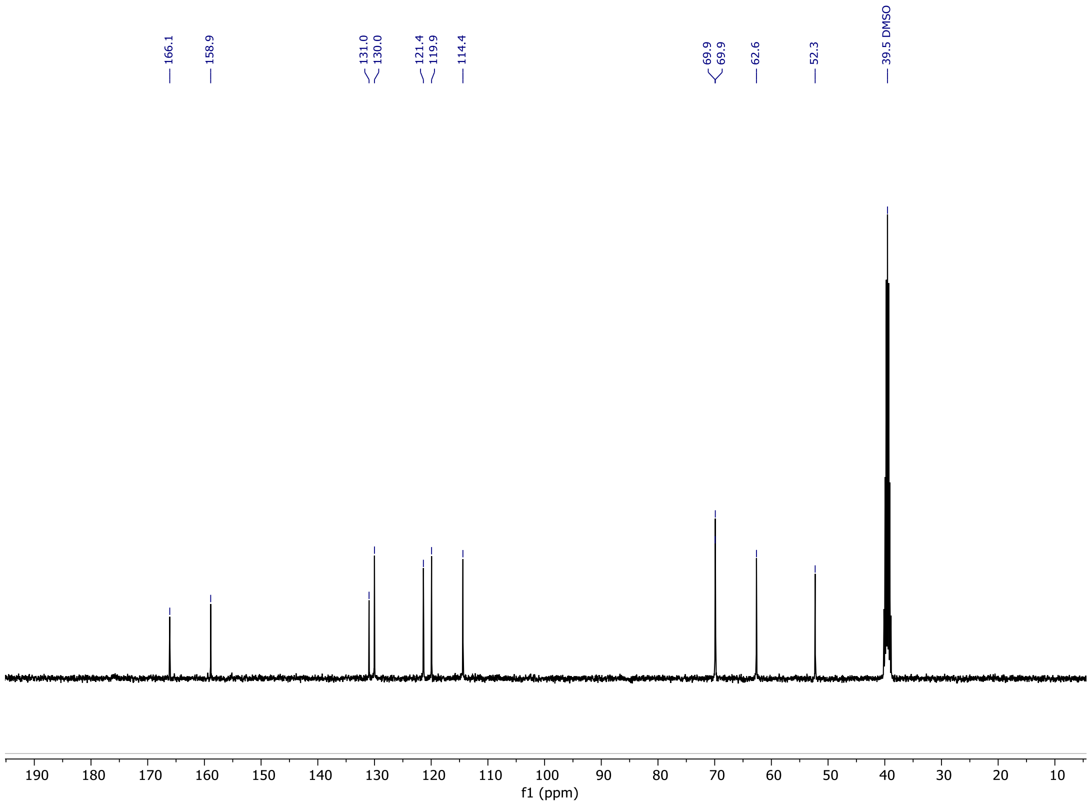

^{1}H-NMR spectrum of **16i** ($(CD_3)_2CO$, 400 MHz)

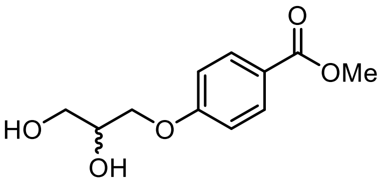

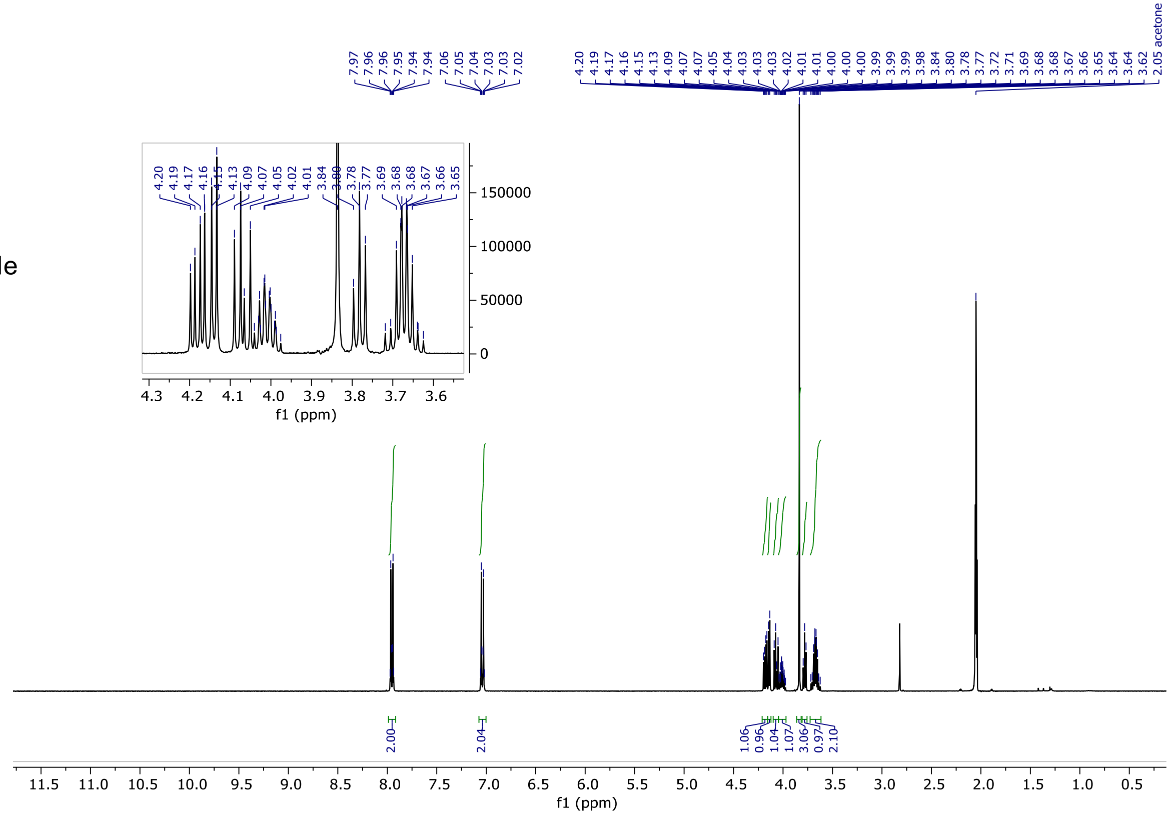

^{13}C-NMR spectrum of **16i** ($(CD_3)_2CO$, 101 MHz)

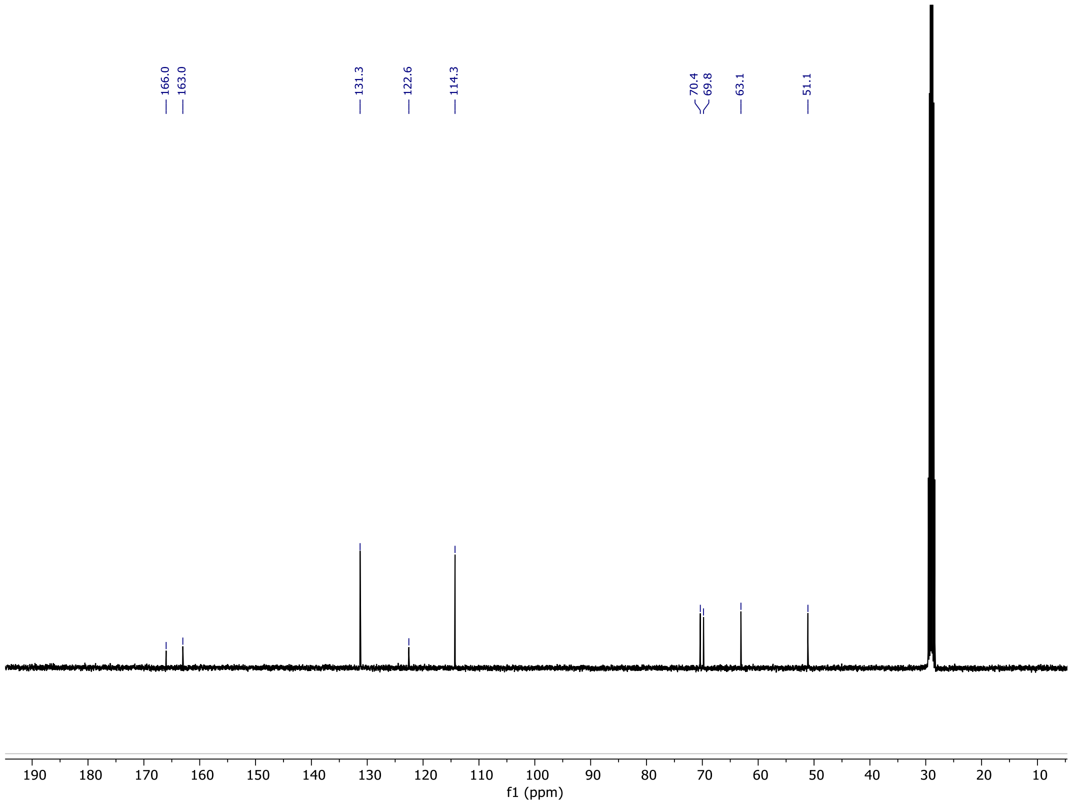

^{1}H-NMR spectrum of **17a** (400 MHz, D_2O)

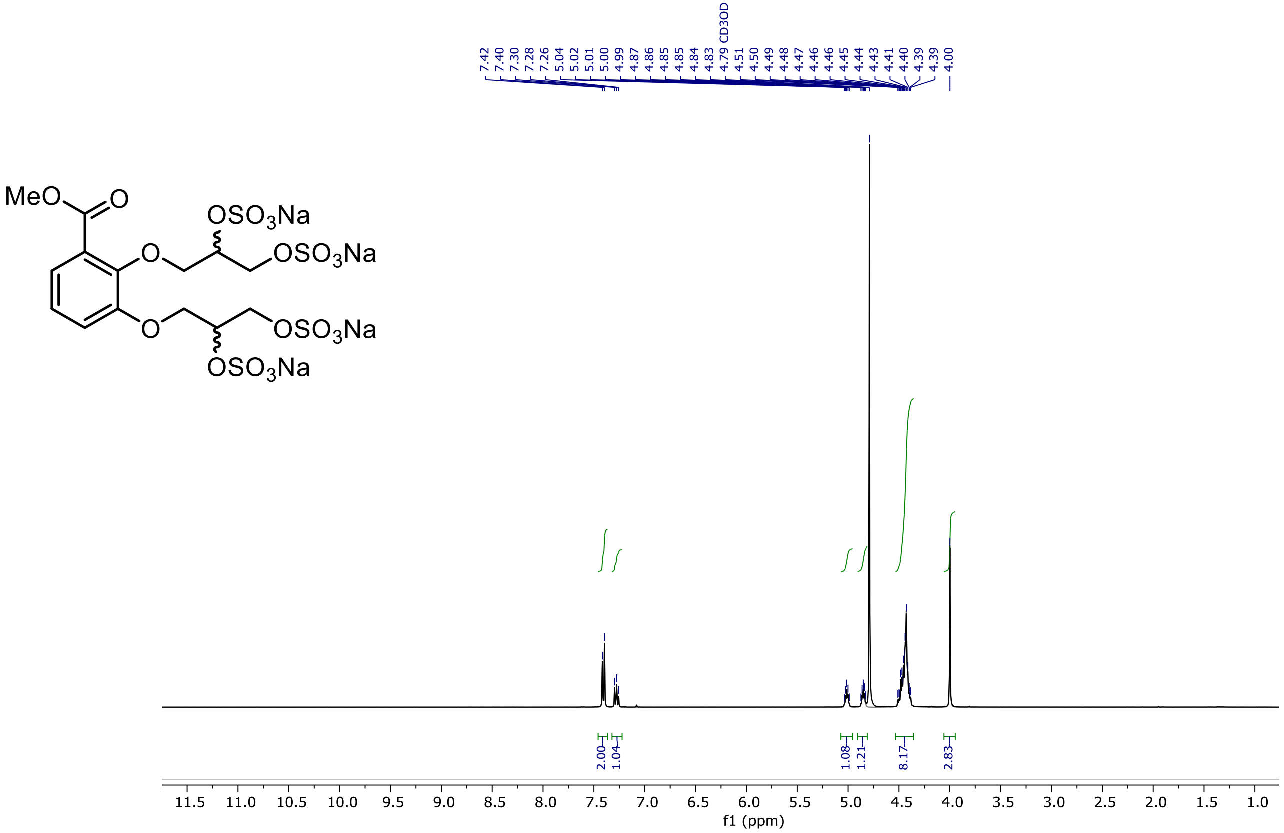

^{13}C-NMR spectrum of **17a** (101 MHz, D_2O)

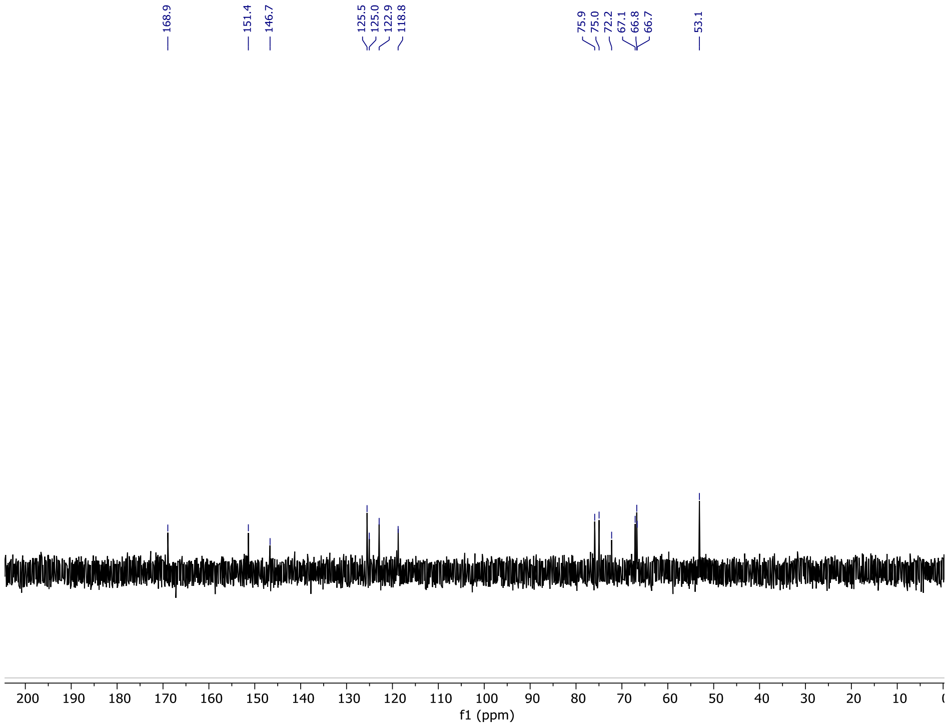

^{1}H-NMR spectrum of **17b** (400 MHz, $(CD_3)_2SO$)

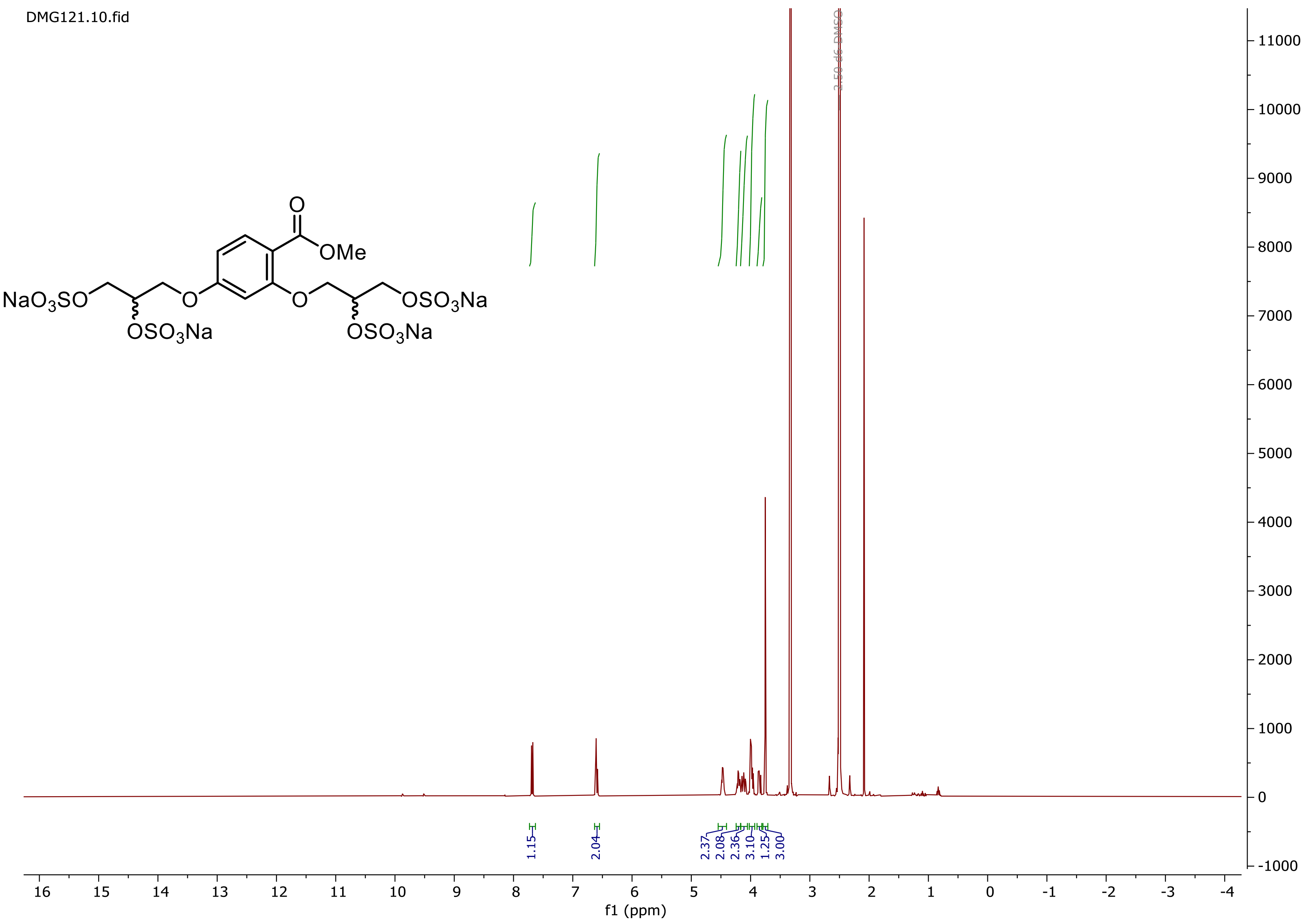

^{1}H-NMR spectrum of **17c** (400 MHz, $(CD_3)_2SO$)

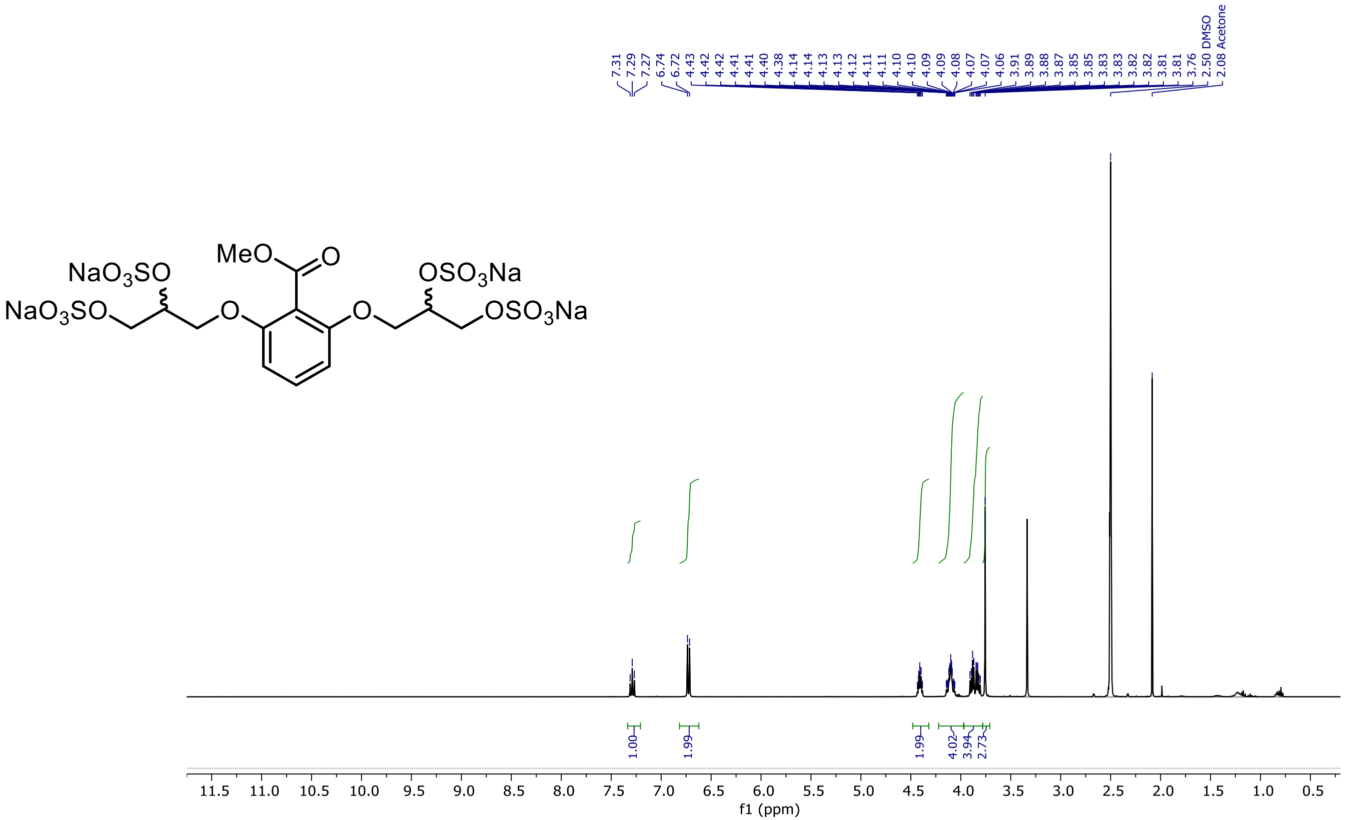

^{13}C-NMR spectrum of **17c** (101 MHz, $(CD_3)_2SO$)

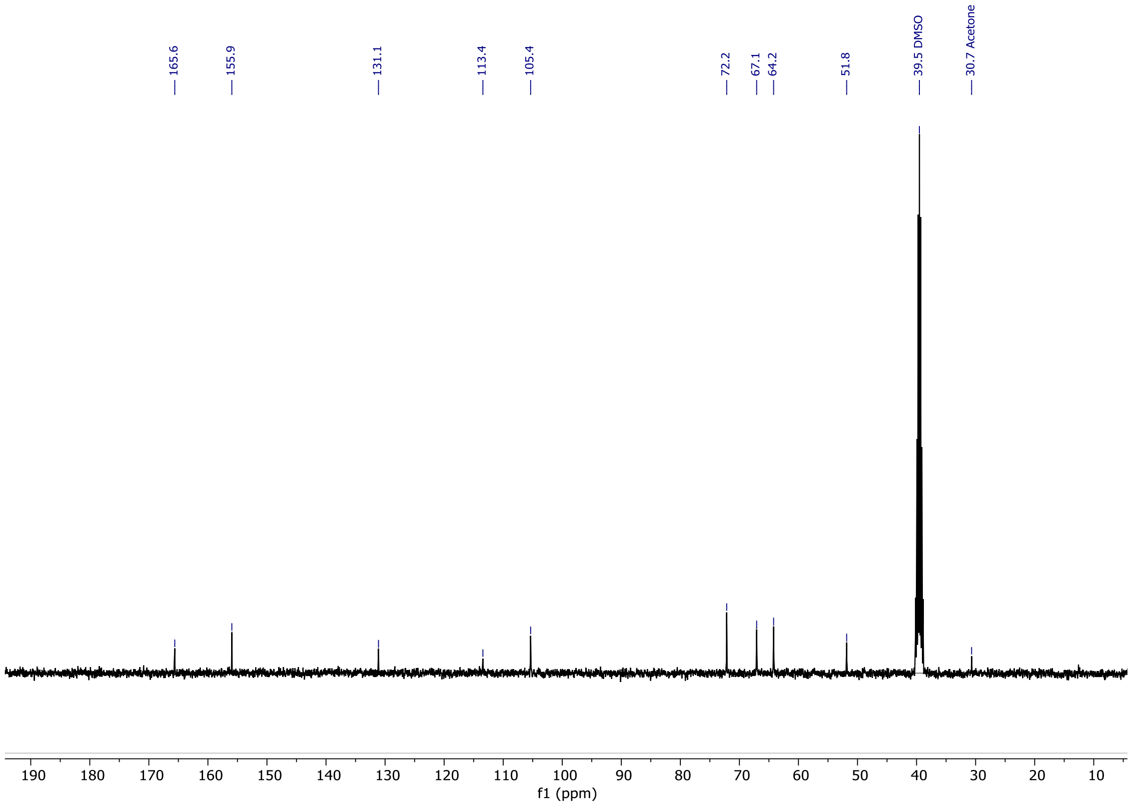

^{1}H-NMR spectrum of **17d** (400 MHz, D_2O)

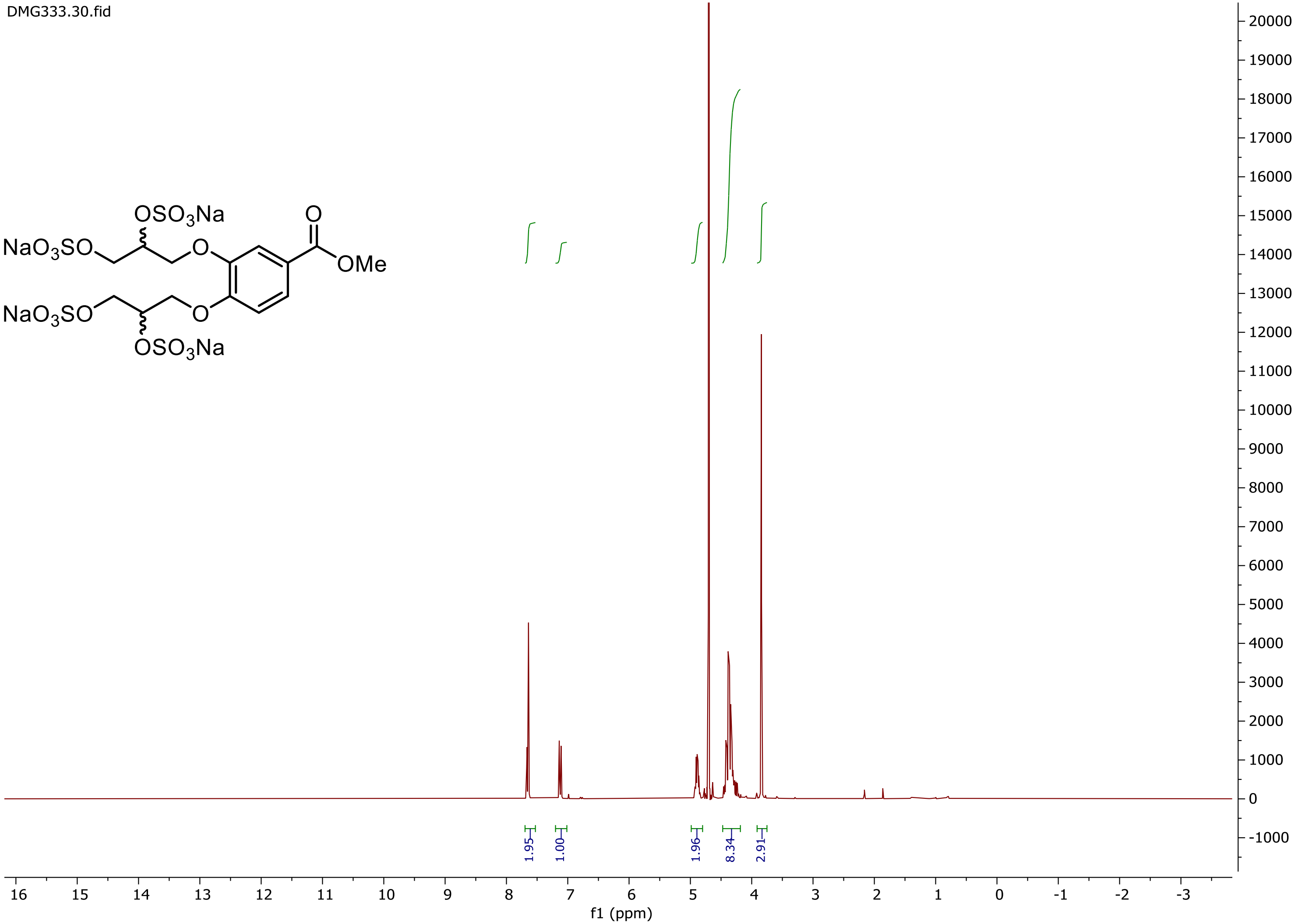

^{13}C-NMR spectrum of **17d** (101 MHz, D_2O)

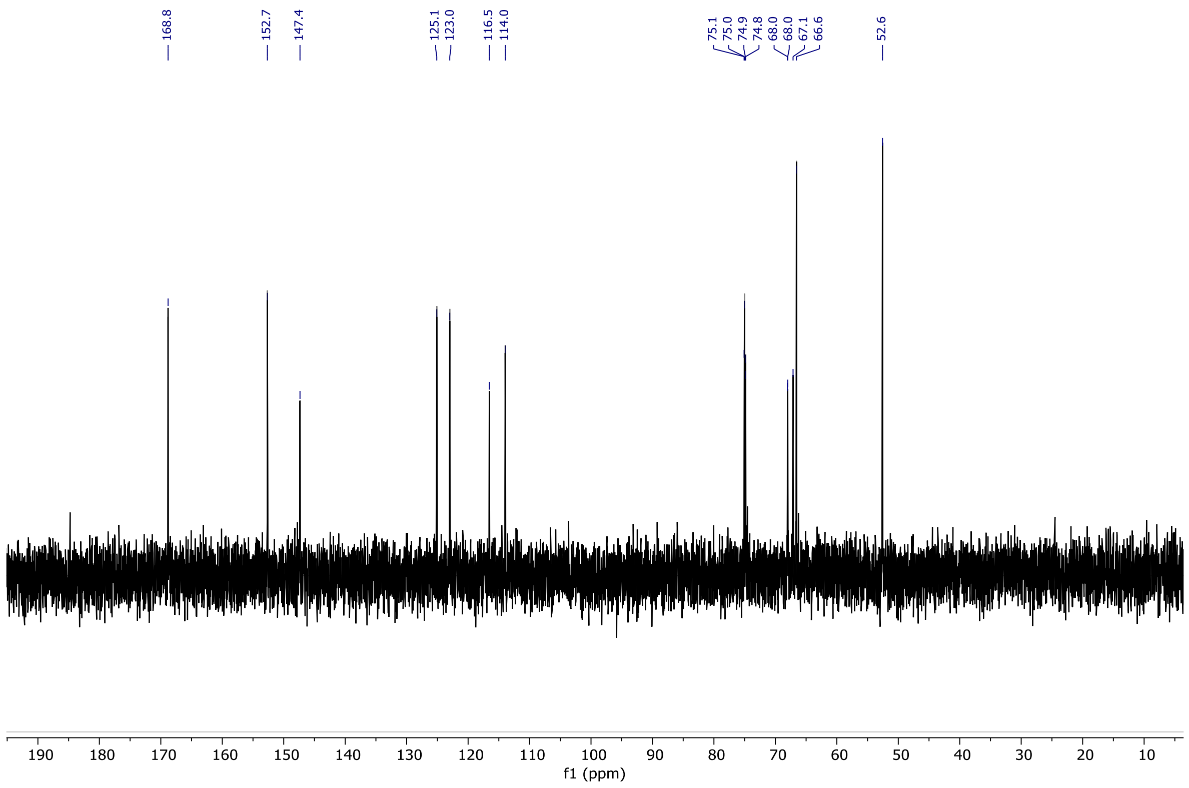

^{1}H-NMR spectrum of **17e** (400 MHz, D_2O)

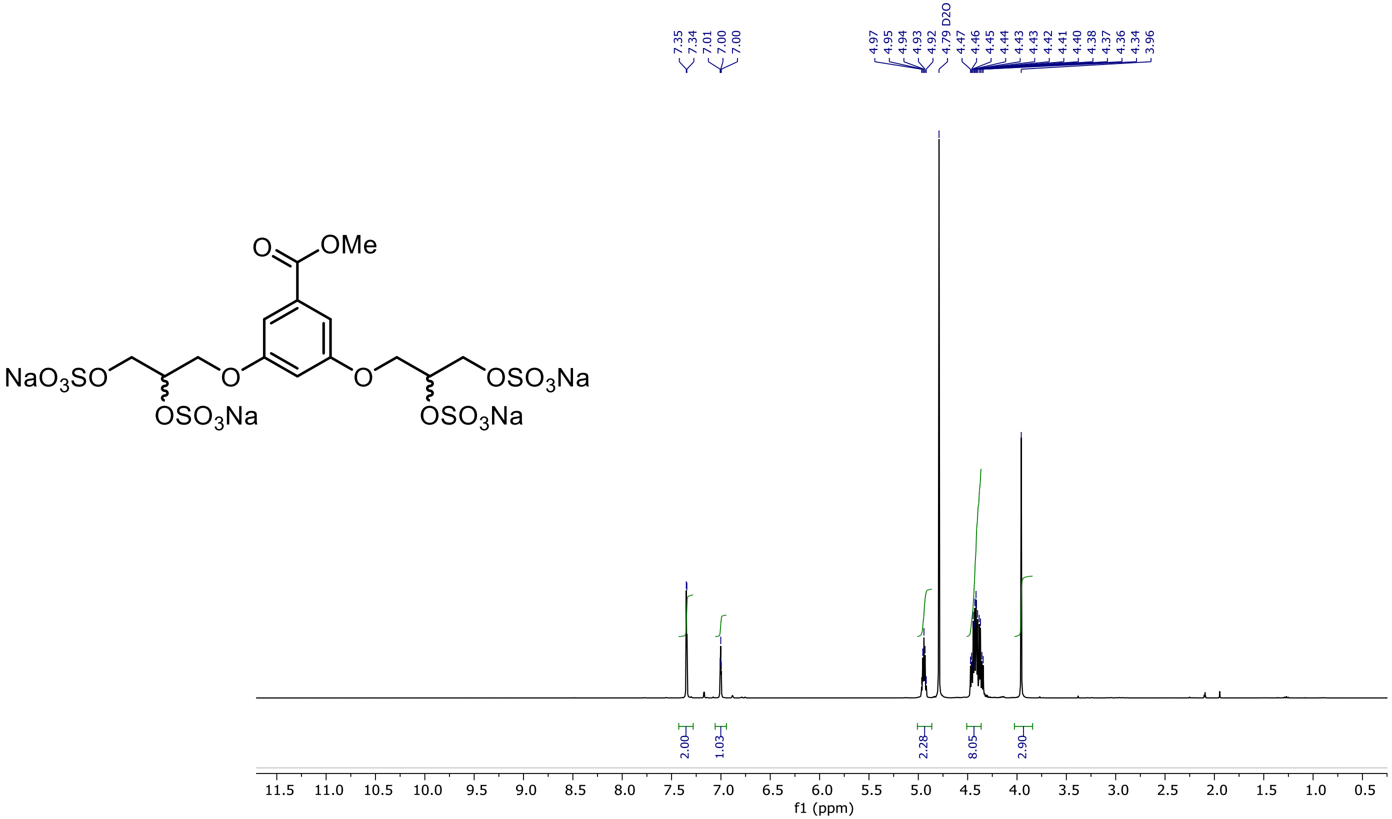

^{13}C-NMR spectrum of **17e** (101 MHz, D_2O)

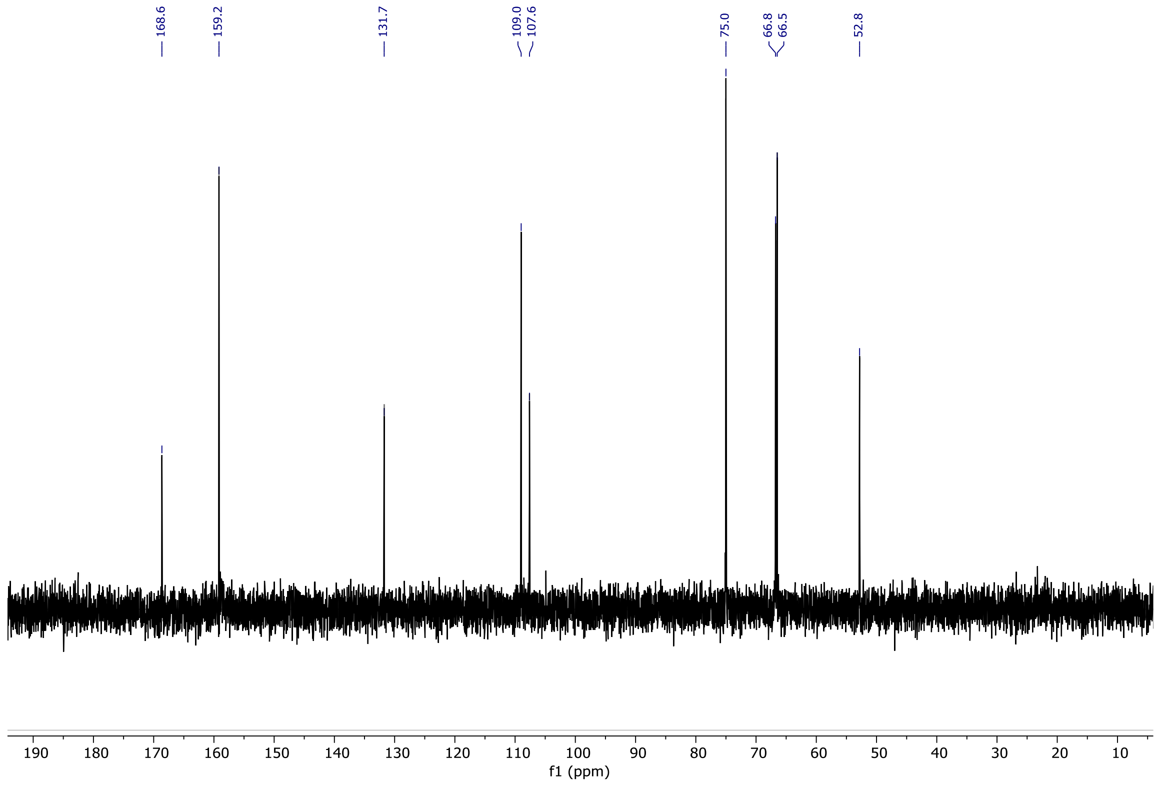

^{1}H-NMR spectrum of **17f** (400 MHz, D_2O)

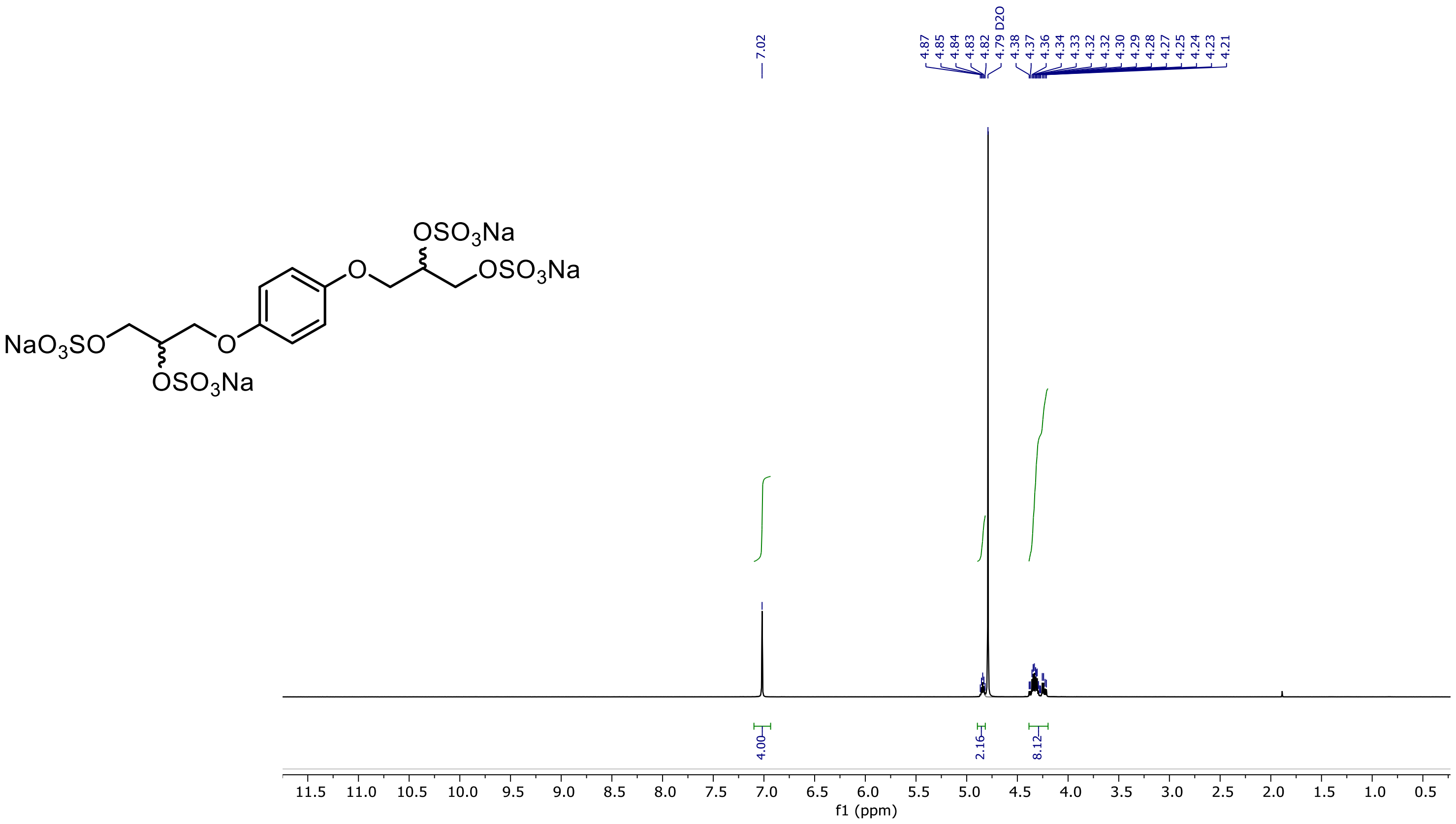

^{13}C-NMR spectrum of **17f** (101 MHz, D_2O)

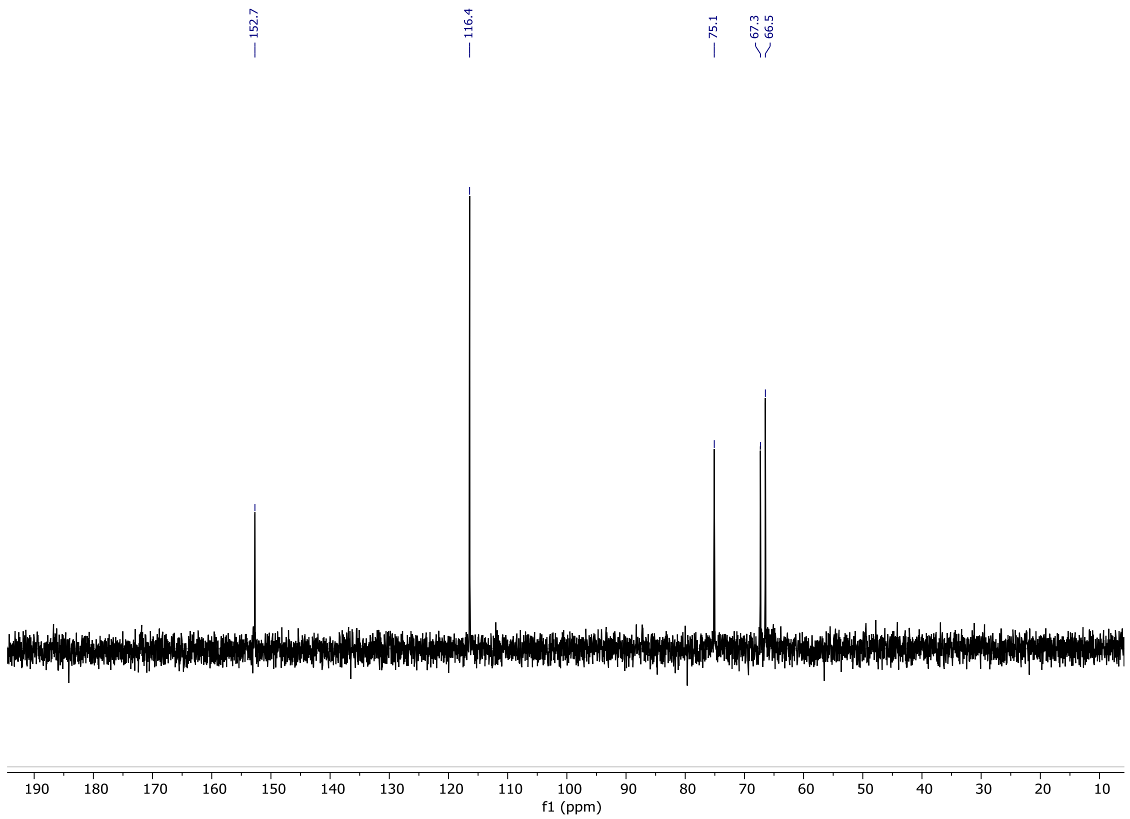

^{1}H-NMR spectrum of **17g** (400 MHz, $(CD_3)_2SO$)

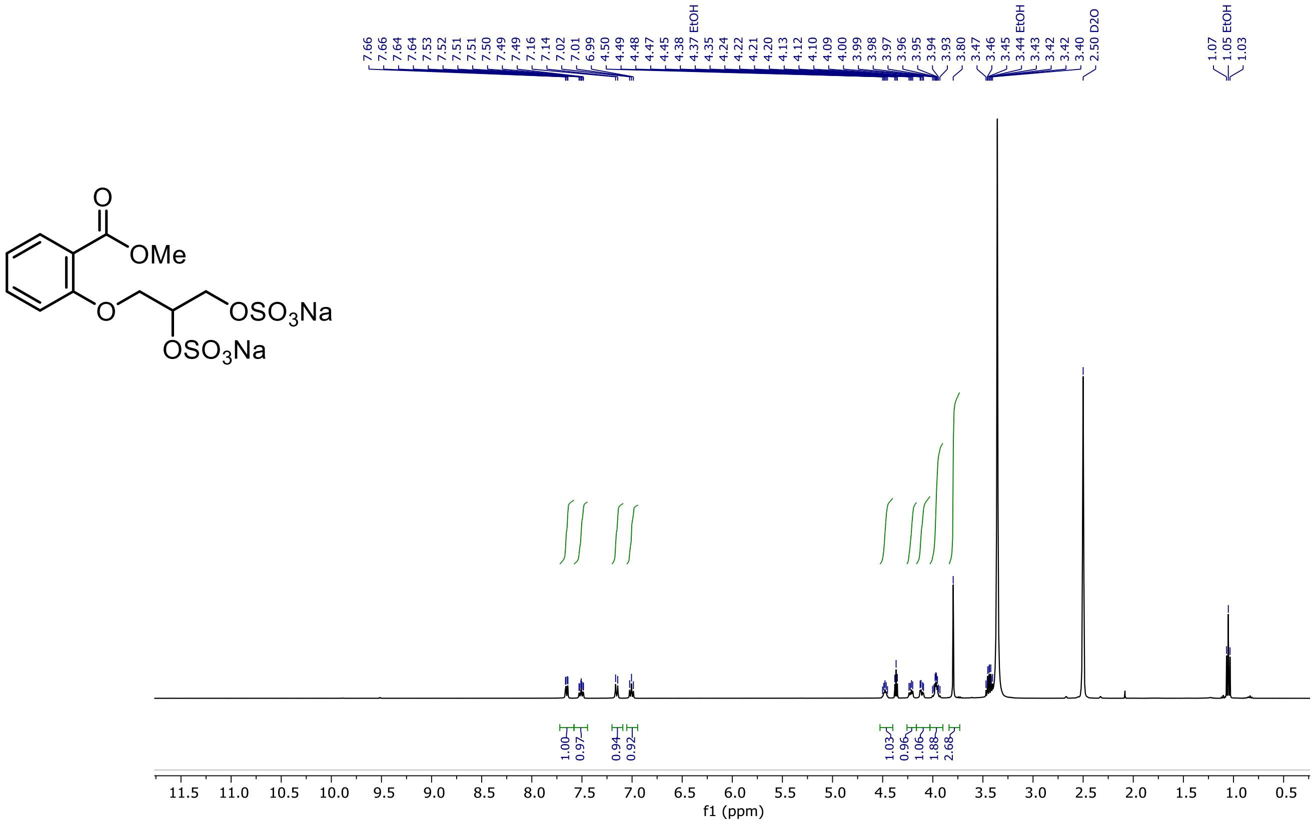

^{13}C-NMR spectrum of **17g** (101 MHz, $(CD_3)_2SO$)

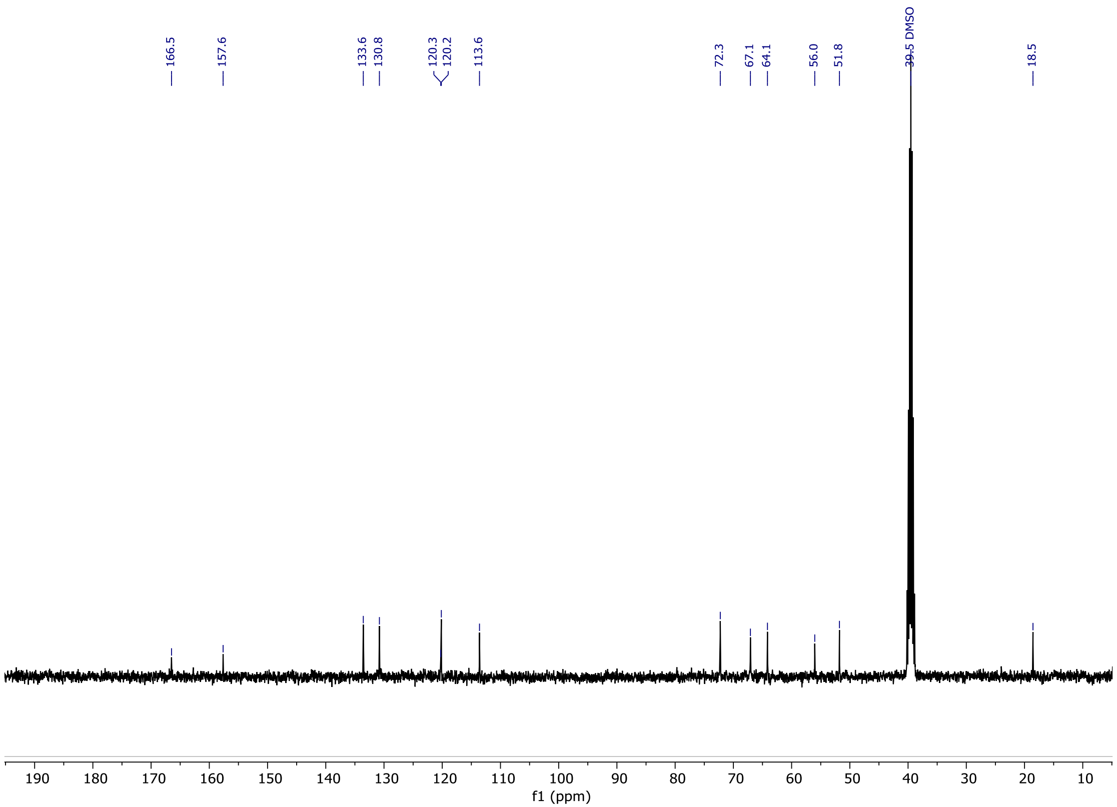

^{1}H-NMR spectrum of **17h** (400 MHz, $(CD_3)_2SO$)

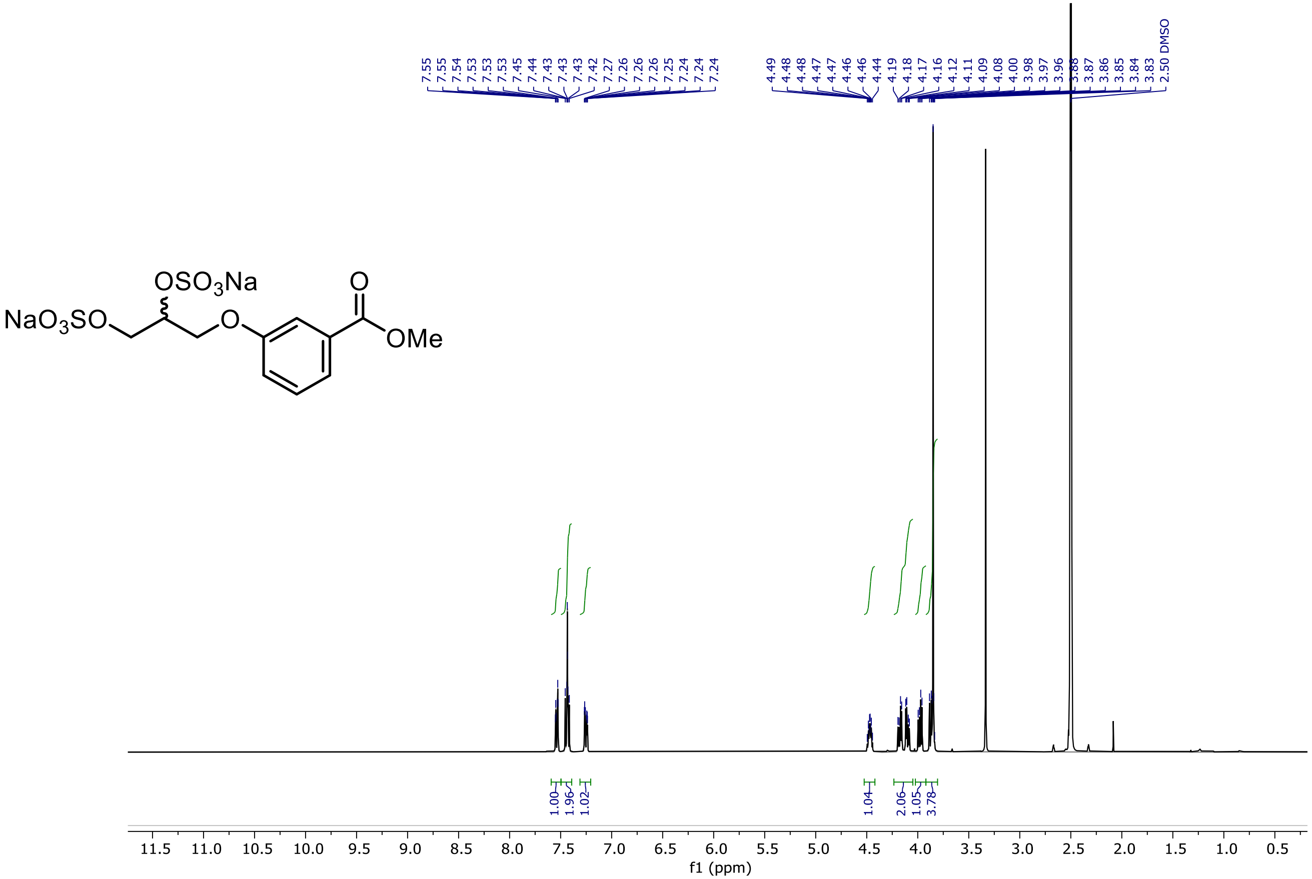

^{13}C-NMR spectrum of **17h** (101 MHz, $(CD_3)_2SO$)

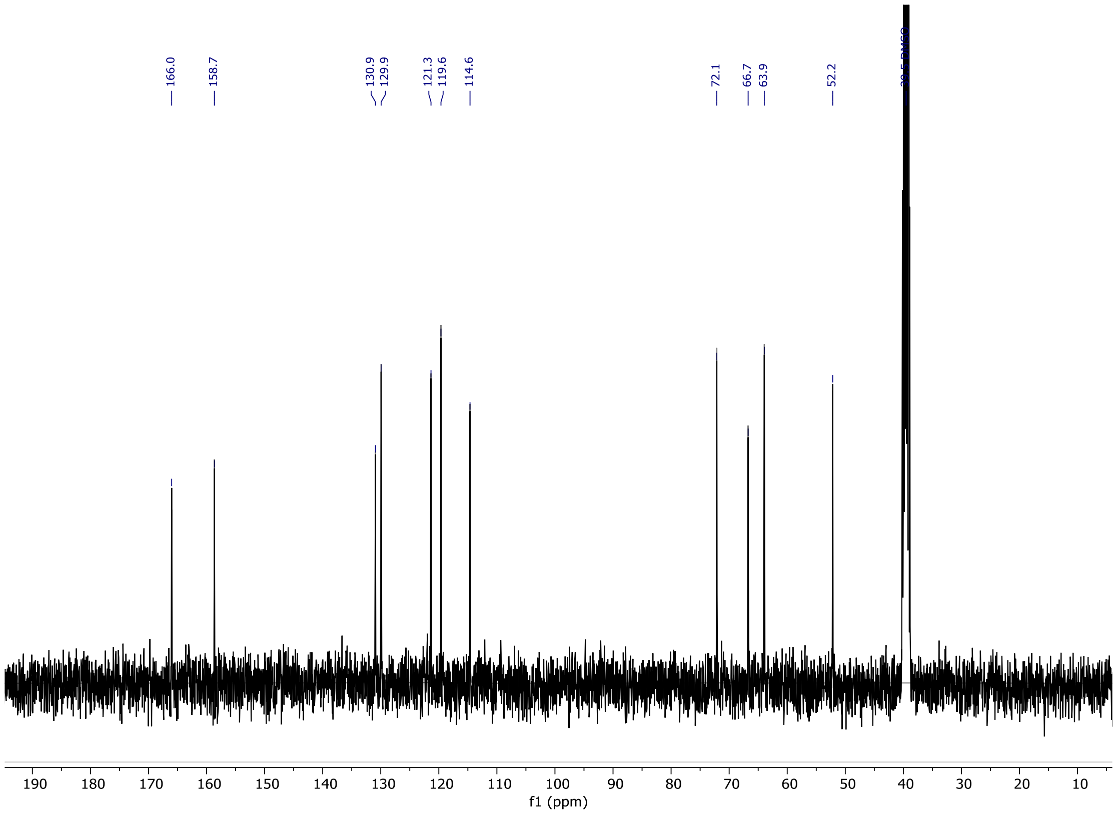

^{1}H-NMR spectrum of **17i** (400 MHz, D_2O)

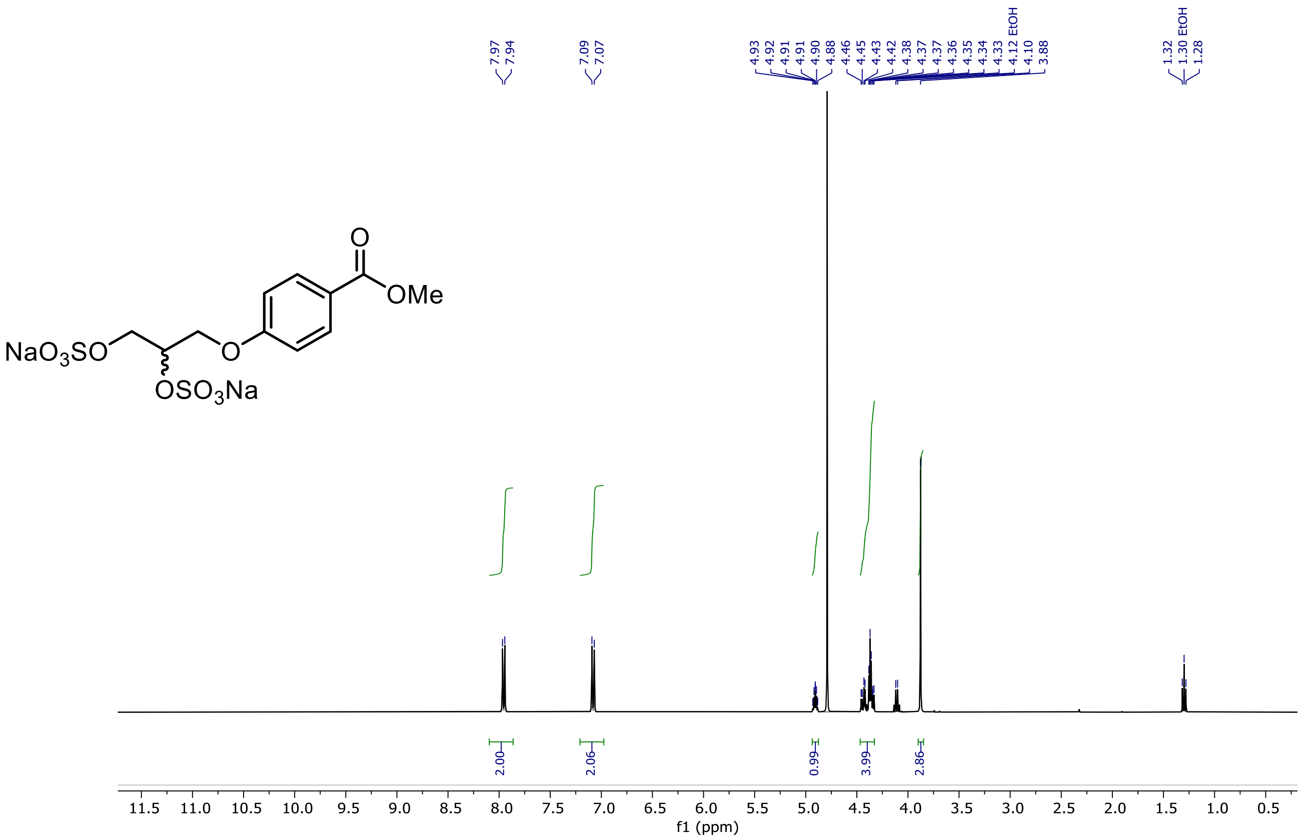

^{13}C-NMR spectrum of **17i** (101 MHz, D_2O)

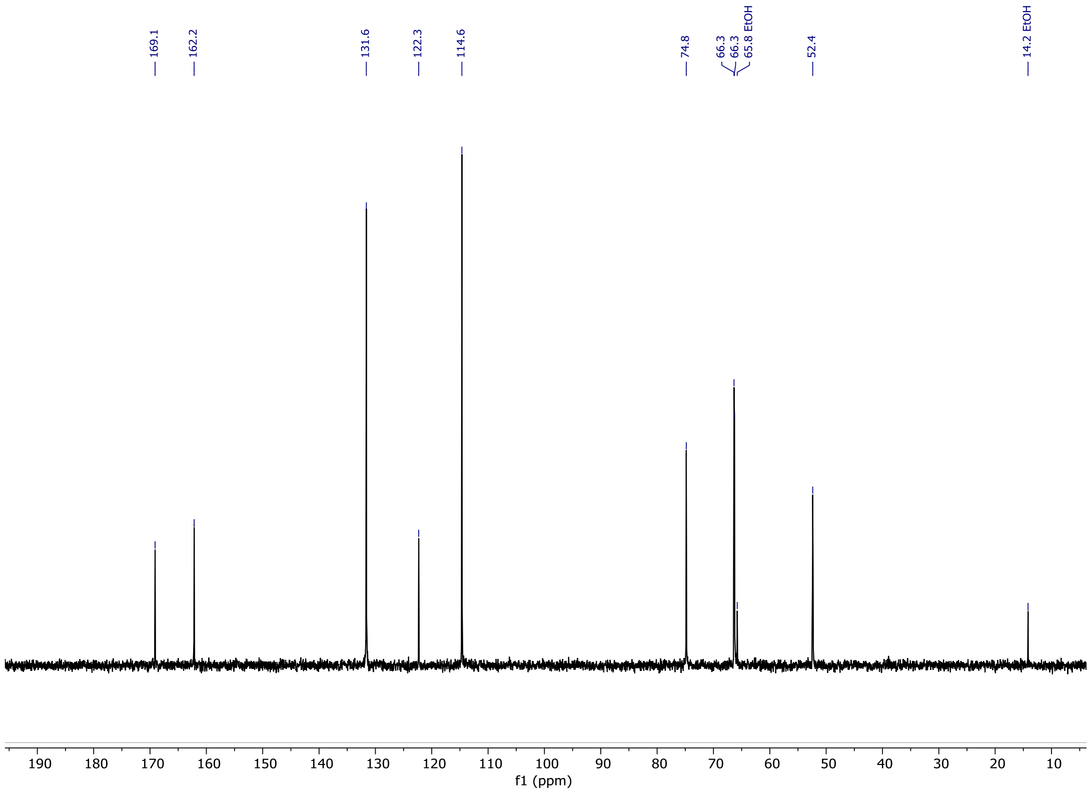

^{1}H NMR spectrum of **20** ($CDCl_3$, 400 MHz)

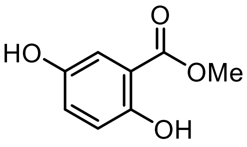

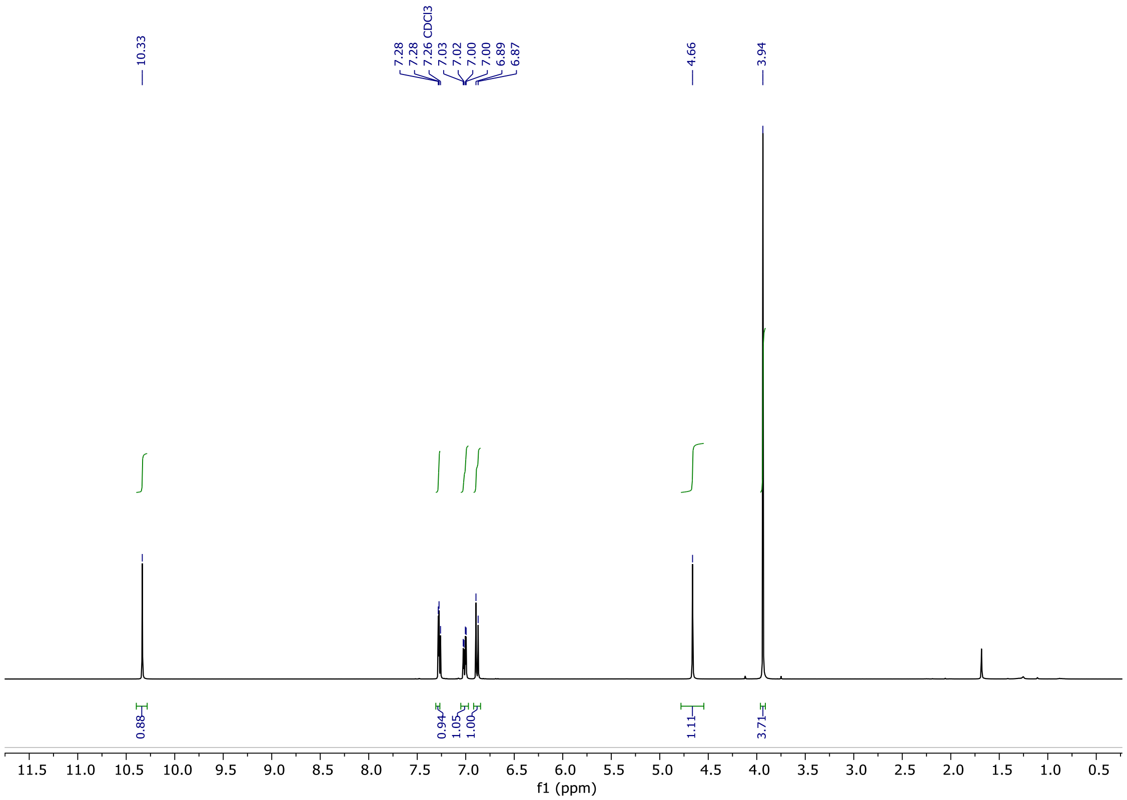

^{13}C NMR spectrum of **20** ($CDCl_3$, 101 MHz)

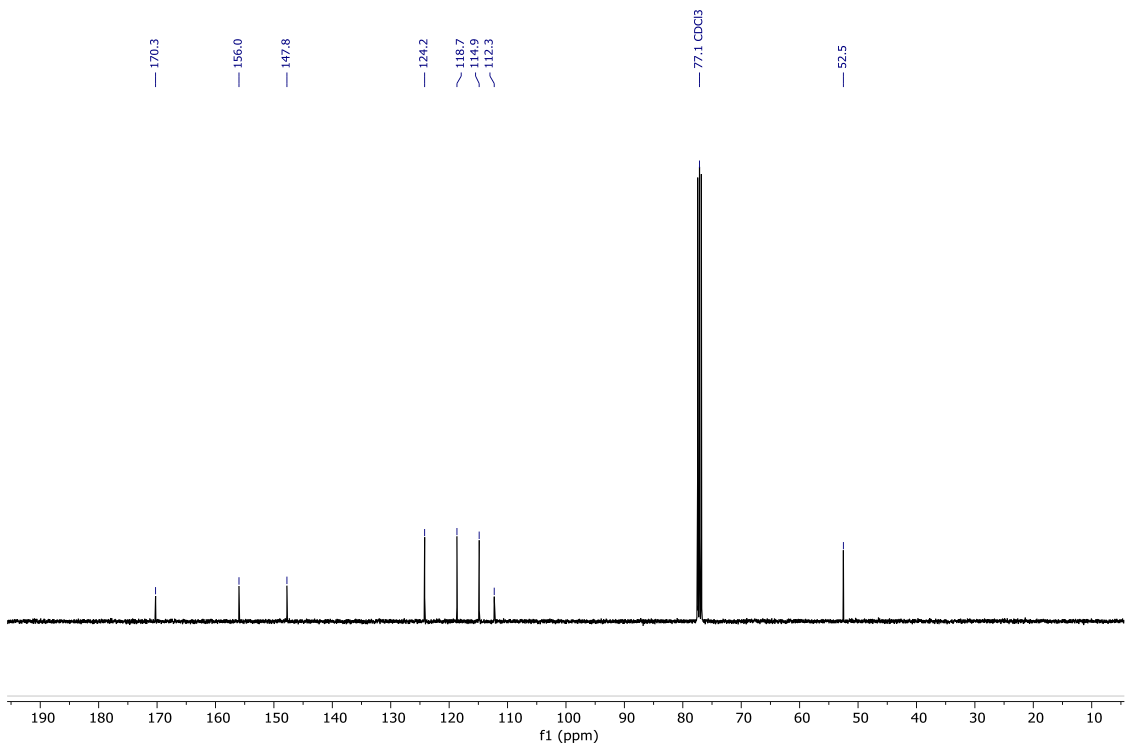

^{1}H-NMR spectrum of **22** (400 MHz, $CDCl_3$)

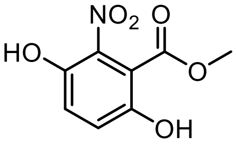

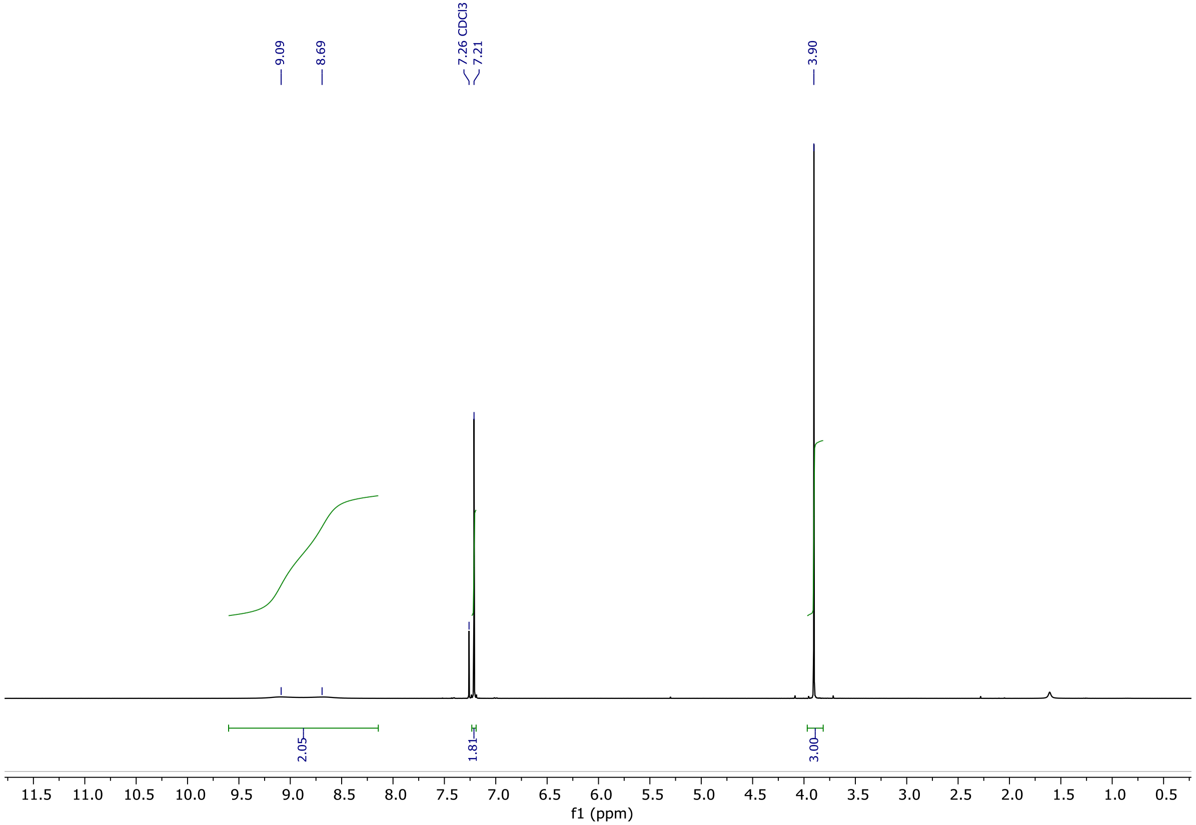

^{13}C-NMR spectrum of **22** (101 MHz, $CDCl_3$)

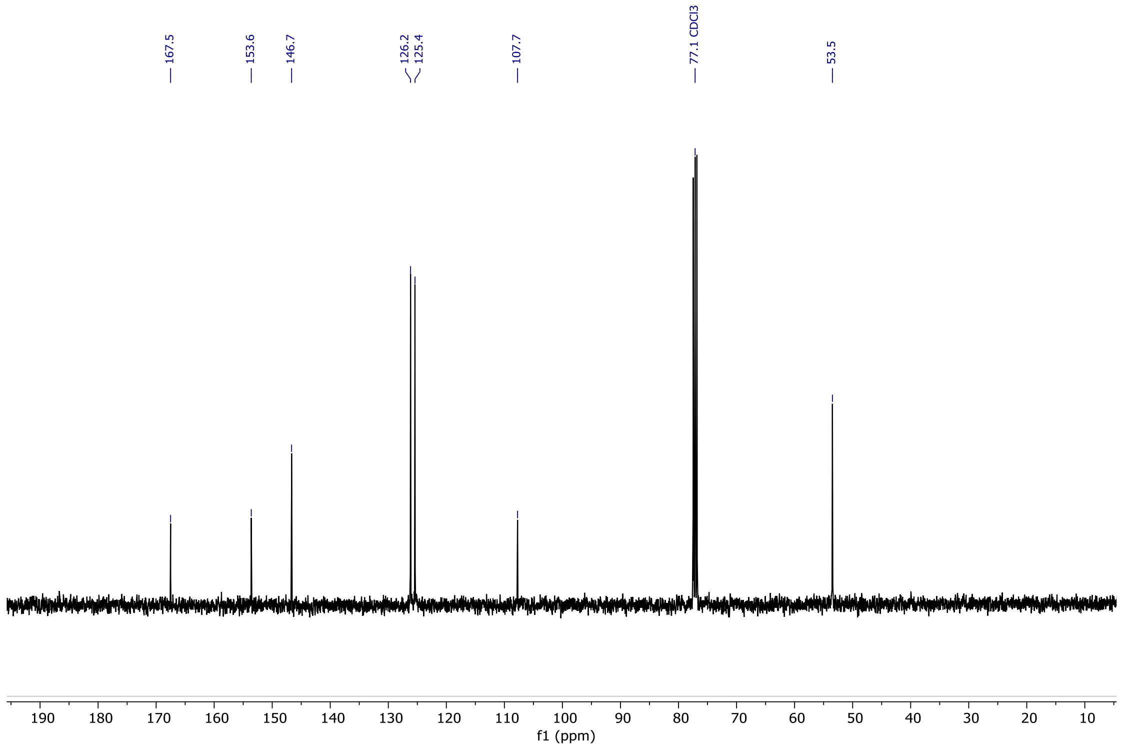

^{1}H-NMR spectrum of **23** (400 MHz, $CDCl_3$)

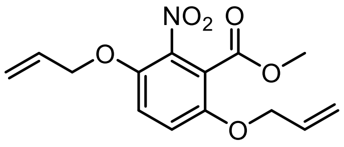

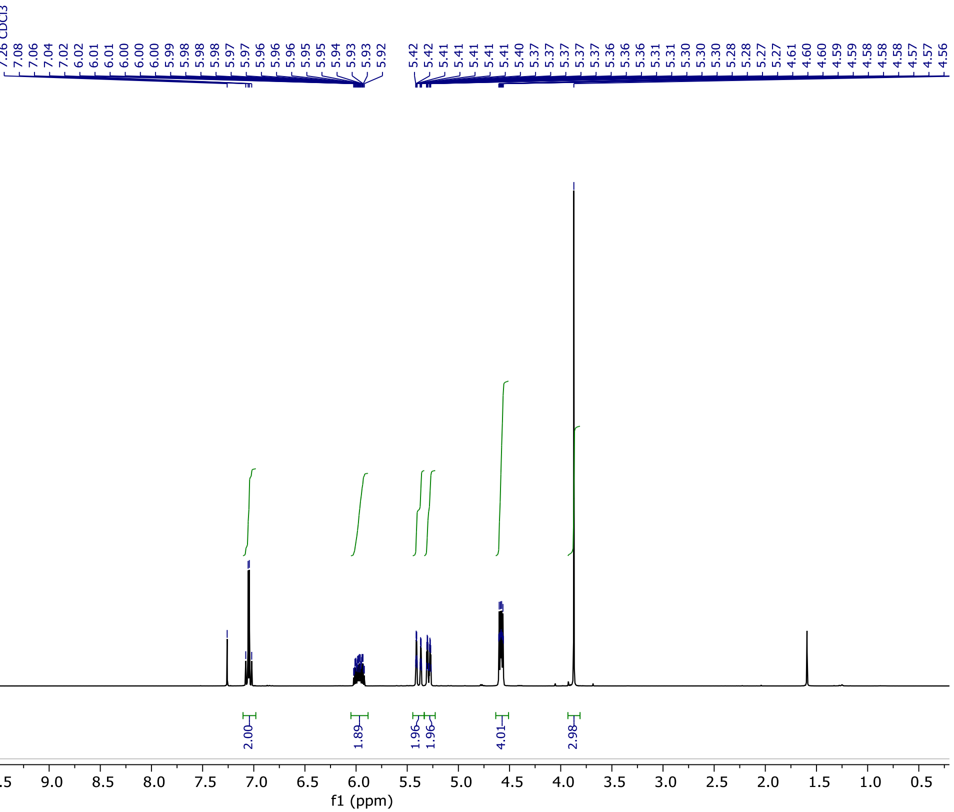

^{13}C-NMR spectrum of **23** (101 MHz, $CDCl_3$)

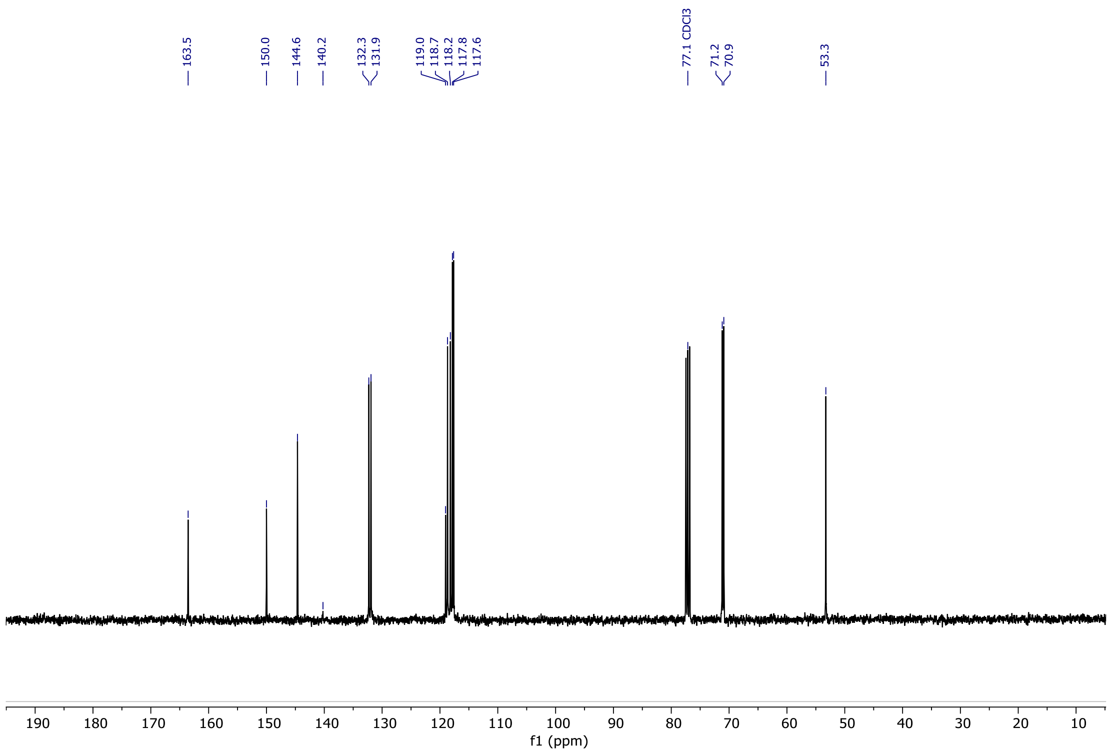

^{1}H-NMR spectrum of **24** (400 MHz, $(CD_3)_2SO$)

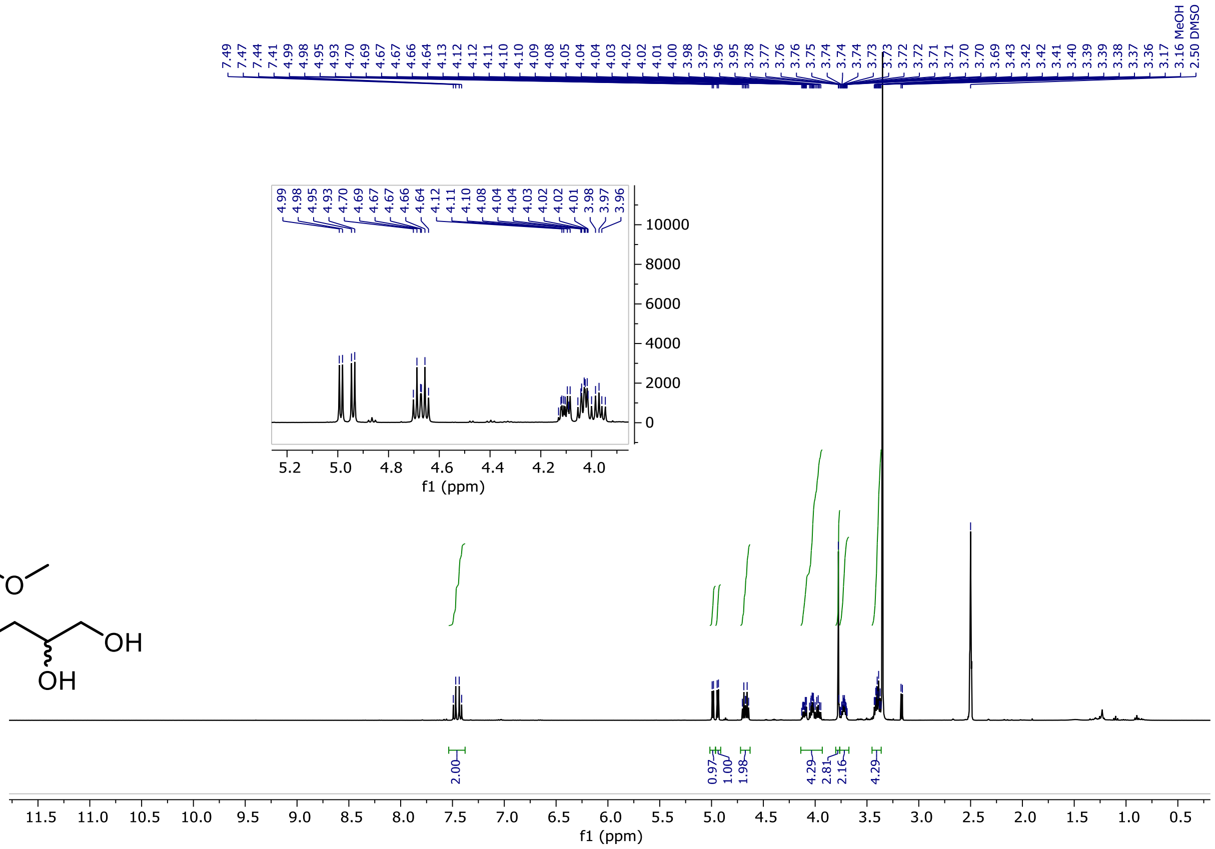

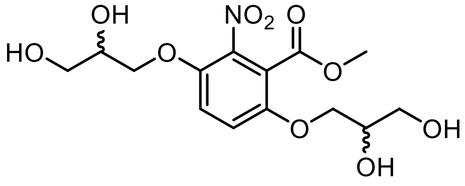

^{13}C-NMR spectrum of **24** (101 MHz, $(CD_3)_2SO$)

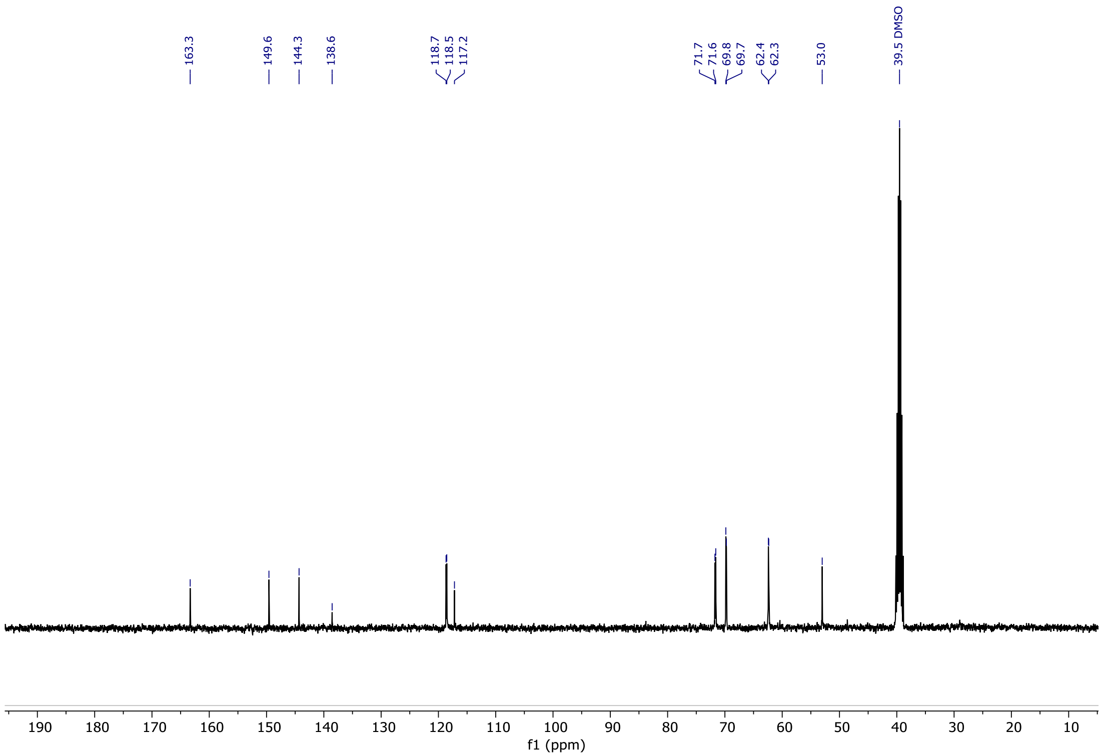

^{1}H-NMR spectrum of **25** (400 MHz, $(CD_3)_2SO$)

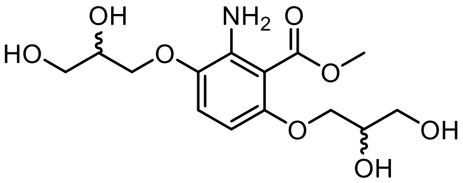

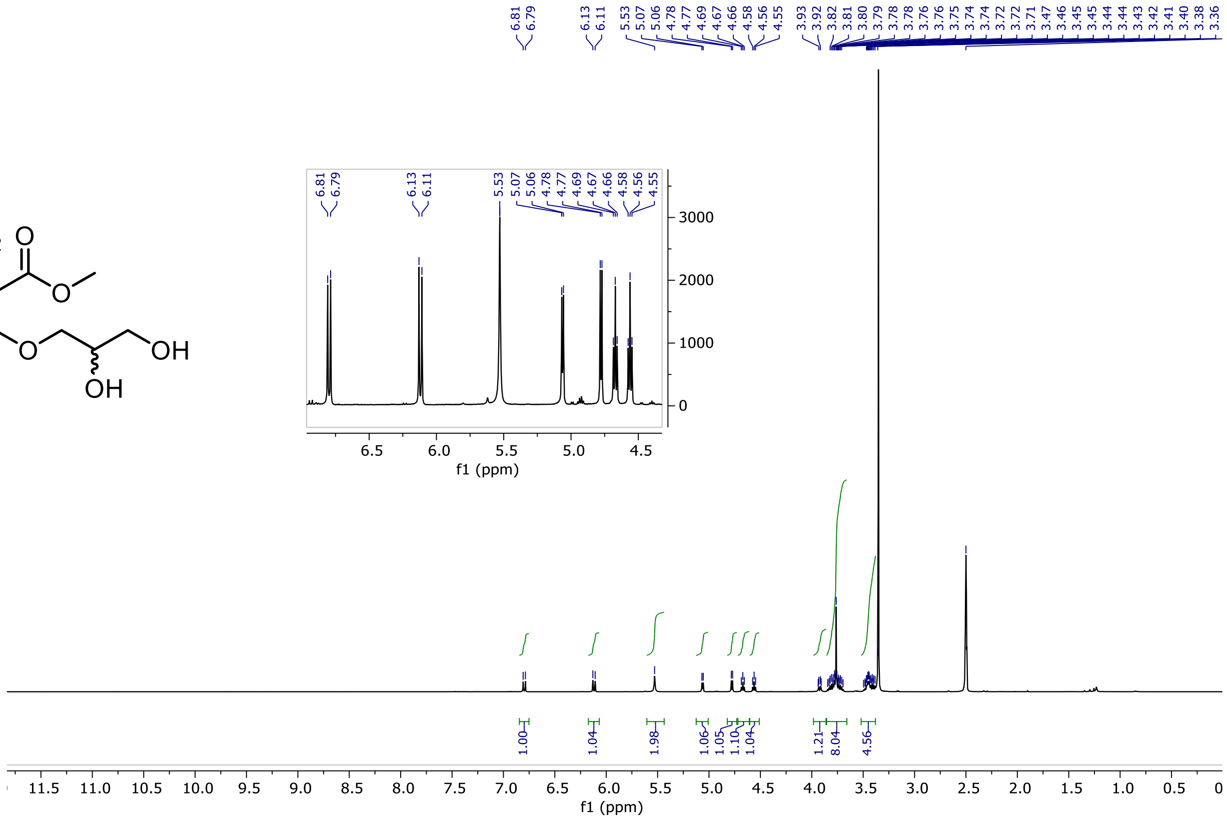

^{13}C-NMR spectrum of **25** (101 MHz, $(CD_3)_2SO$)

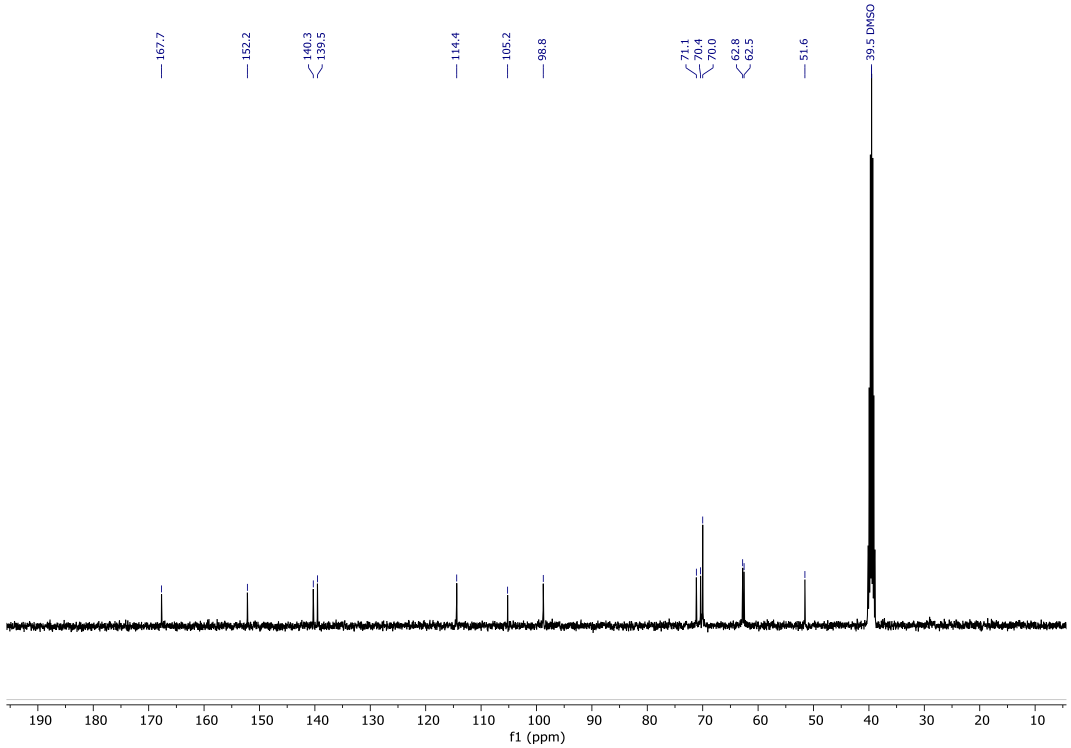

^{1}H-NMR spectrum of **26** (400 MHz, D_2O)

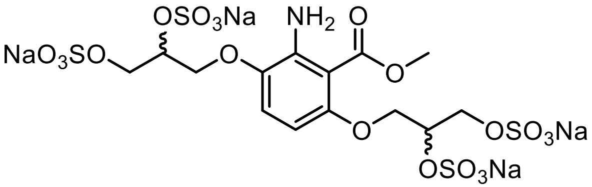

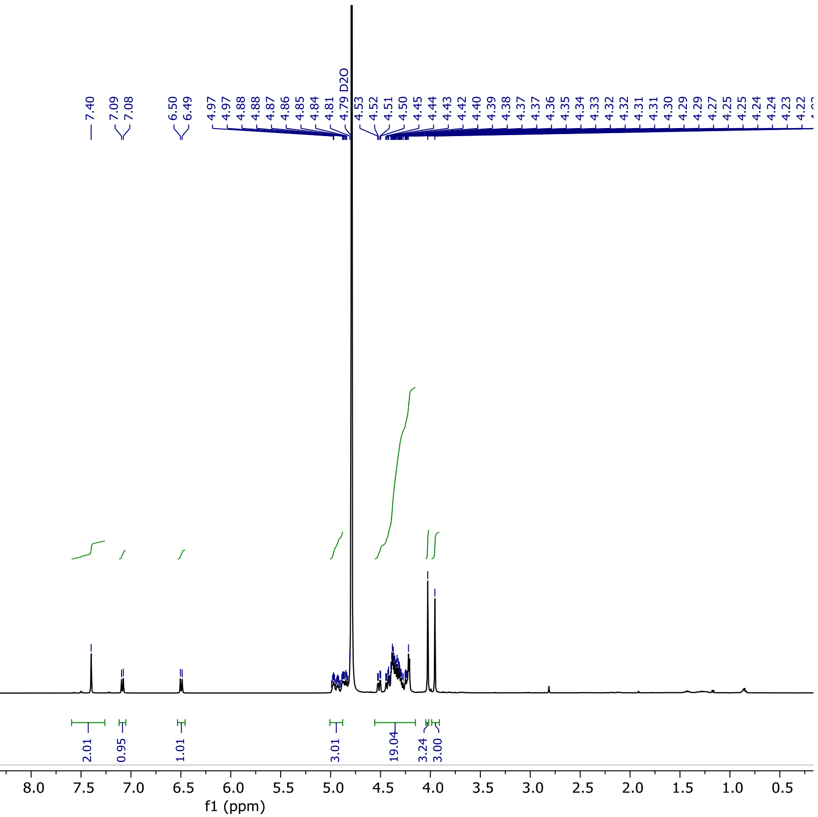

^{13}C-NMR spectrum of **26** (101 MHz, D_2O)

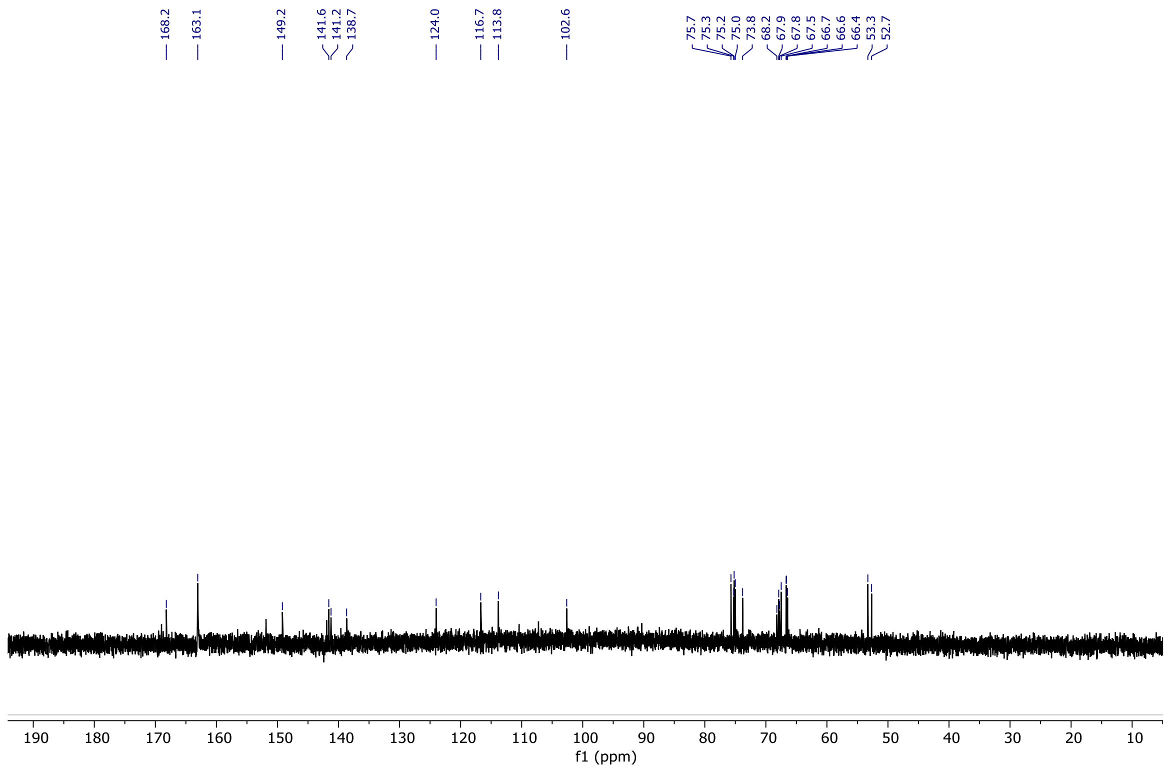

Crystal structure determination of 22

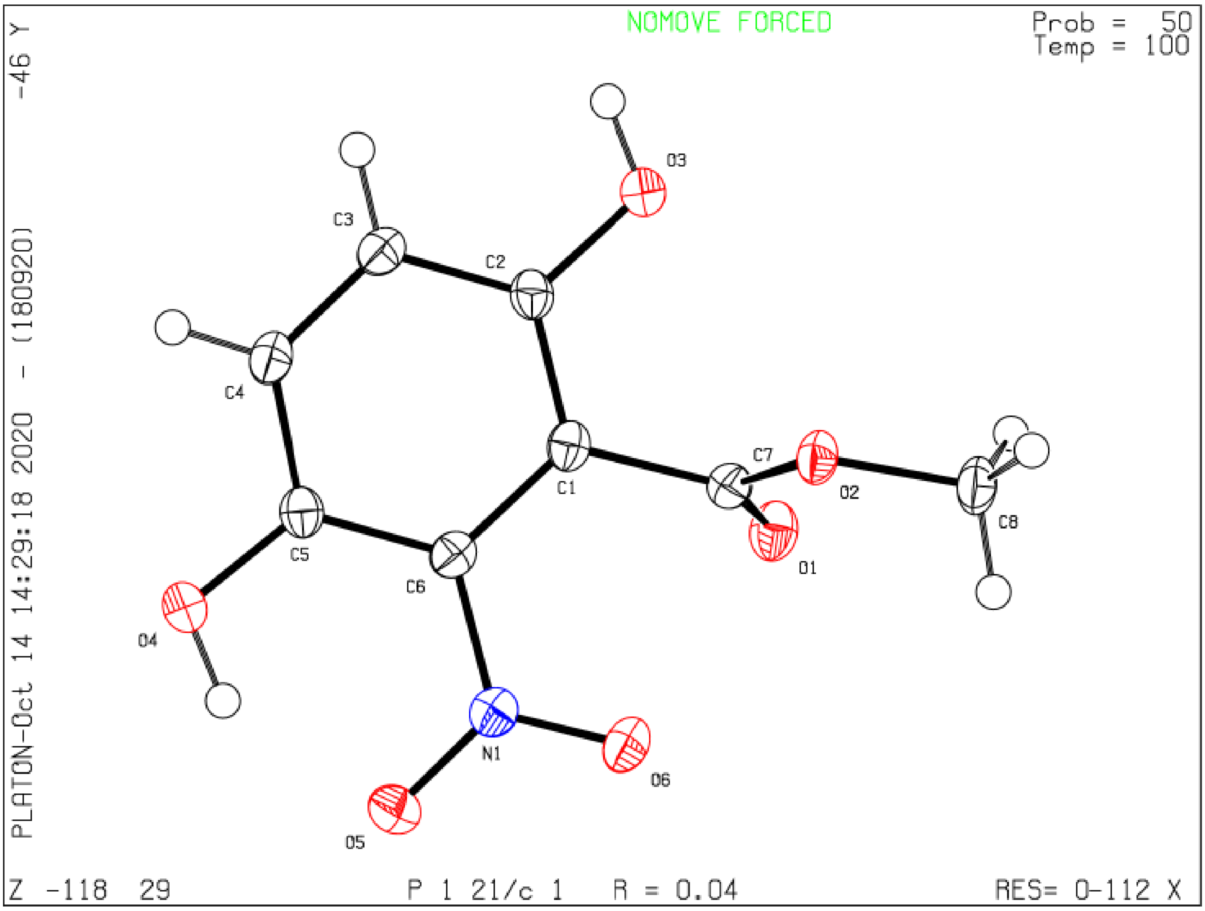

$C_8H_7NO_6$ (M =213.15 g/mol): monoclinic, space group $P2_1/c$ (no. 14), a = 8.9555(7) Å, b = 10.1597(6) Å, c = 9.9607(8) Å, β = 110.056(9)°, V = 851.32(12) Å^3, Z = 4, T = 100.00(10) K, μ(CuKα) = 1.278 mm^{-1}, $Dcalc$ = 1.663 g/cm^3, 3177 reflections measured (10.516° ≤ 2Θ ≤ 149.958°), 1687 unique (R_{int} = 0.0197, R_{sigma} = 0.0263) which were used in all calculations. The final R_1 was 0.0356 (I > 2σ(I)) and wR_2 was 0.0944 (all data).

A suitable crystal was selected and a dataset for **22** was measured on an Agilent SuperNova diffractometer using an Atlas detector. The data collection was driven and processed and an absorption correction was applied using CrysAlisPro.[S26] The structure was solved using ShelXT[S27] and was refined by a full-matrix least-squares procedure on F^2 in ShelXL.[S28] Figures and reports were produced using OLEX2.[S29] All non-hydrogen atoms were refined with anisotropic displacement parameters. The hydroxyl hydrogen atoms bonded to O(3) and O(4) were located in the electron density and freely refined. All other hydrogen atoms were fixed as riding models and the isotropic thermal parameters (U_{iso}) were based on the U_{eq} of the parent atoms.

CCDC 2038360 contains the supplementary crystallographic data for this paper. These data can be obtained free of charge from The Cambridge Crystallographic Data Centre via www.ccdc.cam.ac.uk/data_request/cif.

**Table S1**: Crystal data and structure refinement for **22**	
Identification code	DMG 621
Empirical formula	$C_8H_7NO_6$
Formula weight	213.15
Temperature/K	100.00(10)
Crystal system	monoclinic
Space group	$P2_1/c$
a/Å	8.9555(7)
b/Å	10.1597(6)
c/Å	9.9607(8)
α/°	90
β/°	110.056(9)
γ/°	90
Volume/Å^3	851.32(12)
Z	4
ρ_{calc}g/cm^3	1.663
μ/mm^{-1}	1.278
F(000)	440.0
Crystal size/mm^3	0.149 × 0.146 × 0.119
Radiation	CuKα (λ = 1.54184)
2Θ range for data collection/°	10.516 to 149.958
Index ranges	-10 ≤ h ≤ 7, -11 ≤ k ≤ 12, -12 ≤ l ≤ 12
Reflections collected	3177
Independent reflections	1687 [R_{int} = 0.0197, R_{sigma} = 0.0263]
Data/restraints/parameters	1687/0/145
Goodness-of-fit on F^2	1.045
Final R indexes [I>=2σ (I)]	R_1 = 0.0356, wR_2 = 0.0884
Final R indexes [all data]	R_1 = 0.0427, wR_2 = 0.0944
Largest diff. peak/hole / e Å^{-3}	0.30/-0.23

Table S2: Fractional Atomic Coordinates (×10^4) and Equivalent Isotropic Displacement Parameters (Å^2×10^3) for **22**. U_{eq} is defined as 1/3 of of the trace of the orthogonalised U_{IJ} tensor.

Atom	*x*	*y*	*z*	U(eq)
C1	2905.8(17)	5107.3(15)	6228.1(15)	16.8(3)
C2	3874.7(18)	6144.2(15)	6151.9(15)	17.7(3)
C3	3936.8(18)	6536.9(15)	4817.5(16)	18.9(3)
C4	3096.0(18)	5863.7(16)	3600.0(16)	19.7(3)
C5	2162.8(18)	4766.4(15)	3636.4(16)	18.3(3)
C6	2048.4(17)	4421.7(15)	4966.0(15)	17.2(3)
C7	2926.9(17)	4697.7(15)	7686.9(15)	17.4(3)
C8	2214(2)	5295.7(17)	9672.1(16)	22.7(4)
N1	927.2(15)	3420.8(13)	5029.9(13)	18.5(3)
O1	3606.4(13)	3723.0(11)	8308.4(11)	22.6(3)
O2	2200.2(13)	5565.8(11)	8231.1(11)	19.4(3)
O3	4771.4(14)	6730.9(12)	7396.1(11)	22.0(3)
O4	1409.1(14)	4165.0(12)	2385.6(11)	23.1(3)
O5	370.3(13)	2663.3(12)	4003.0(11)	24.0(3)
O6	536.6(13)	3374.3(11)	6103.0(11)	22.6(3)

__Supplementary Figures and Tables__

Table S3: Energy scores obtained from docking and from PBSA. Van der Waals, Electrostatic, polar solvation and SASA were obtained from the PBSA calculation.

	Docking Energy Score (kJ/mol)	ΔG from PBSA (kJ/mol)	Van der Waal (kJ/mol)	Electrostatic (kJ/mol)	Polar solvation (kJ/mol)	SASA (kJ/mol)
**α-4**	-47.4	-197.2 ± 10.4	-31.8 ± 2.2	-1139.7 ± 5.6	985.1 ± 13.5	-10.7 ± 0.3
**β-5**	-51.4	-262.8 ± 7.6	-91.7 ± 2.9	-1487.7 ± 8.5	1331.9 ± 14.9	-15.4 ± 0.2
***R*-10**	-41.0	-53.9 ± 29.7	-64.9 ± 5.1	-749.6 ± 8.6	771.1 ± 61.1	-11.4 ± 0.7
***S*-10**	-42.0	-199.2 ± 6.3	-99.2 ± 3.1	-872.9 ± 4.6	789.84 ± 8.5	-16.9 ± 0.2
**α-2**	-9.2	-4.9 ± 4.1	-70.0 ± 2.4	-45.4 ± 2.2	122.8 ± 5.3	-12.3 ± 2.1
**α-3**	-15.8	-64.2 ± 3.1	-62.2 ± 2.1	-333.6 ± 2.9	343.8 ± 4.8	-12.2 ± 0.1
**β-3**	-19.2	-79.9 ± 4.2	-61.3 ± 1.9	-271.4 ± 2.3	263.2 ± 7.3	-10.5 ± 0.3

**17a**	-51.2	-180.9 ± 7.2	-77.1 ± 2.0	-1224.2 ± 5.6	1136.0 ± 9.7	-12.7 ± 0.2
**17b**	-47.3	-157.1 ± 12.4	-16.4 ± 4.7	-1202.7 ± 9.2	1073.6 ± 15.9	-11.6 ± 0.3
**17c**	-42.8	-302.1 ± 14.4	-96.7 ± 3.3	-1102.1 ± 8.2	912.9 ± 21	-16.3 ± 0.4
**17d**	-47.4	-134.6 ± 9.4	-41.5 ± 4.0	-1169.7 ± 6.2	1090.4 ± 10.8	-13.8 ± 0.4
**17e**	-45.4	-165.2 ± 9.1	-70.2 ± 3.5	-1145.2 ± 5.9	1064.9 ± 11.7	-14.8 ± 0.3
**17f**	-45.8	-229.1 ± 13.4	-112.6 ± 3.9	-1193.2 ± 10.9	1082.9 ± 23.2	-16.3 ± 0.4
**17g**	-36.9	-209.3 ± 4.9	-67.3 ± 1.8	-545.3 ± 4.8	414.0 ± 8.6	-10.8 ± 0.2
**17h**	-43.6	-94.0 ± 8.5	-68.9 ± 4.3	-628.9 ± 5.2	614.7 ± 12.4	-10.9 ± 0.5
**17i**	-38.3	-87.9 ± 4.4	-104.5 ± 4.9	-583.1 ± 21.2	613.5 ± 24.9	-13.7 ± 0.6
**26**	-47.7	-416.7 ± 12.3	-68.4 ± 2.5	-1255.5 ± 9.1	919.6 ± 21.2	-12.5 ± 0.3

Ligplot diagrams

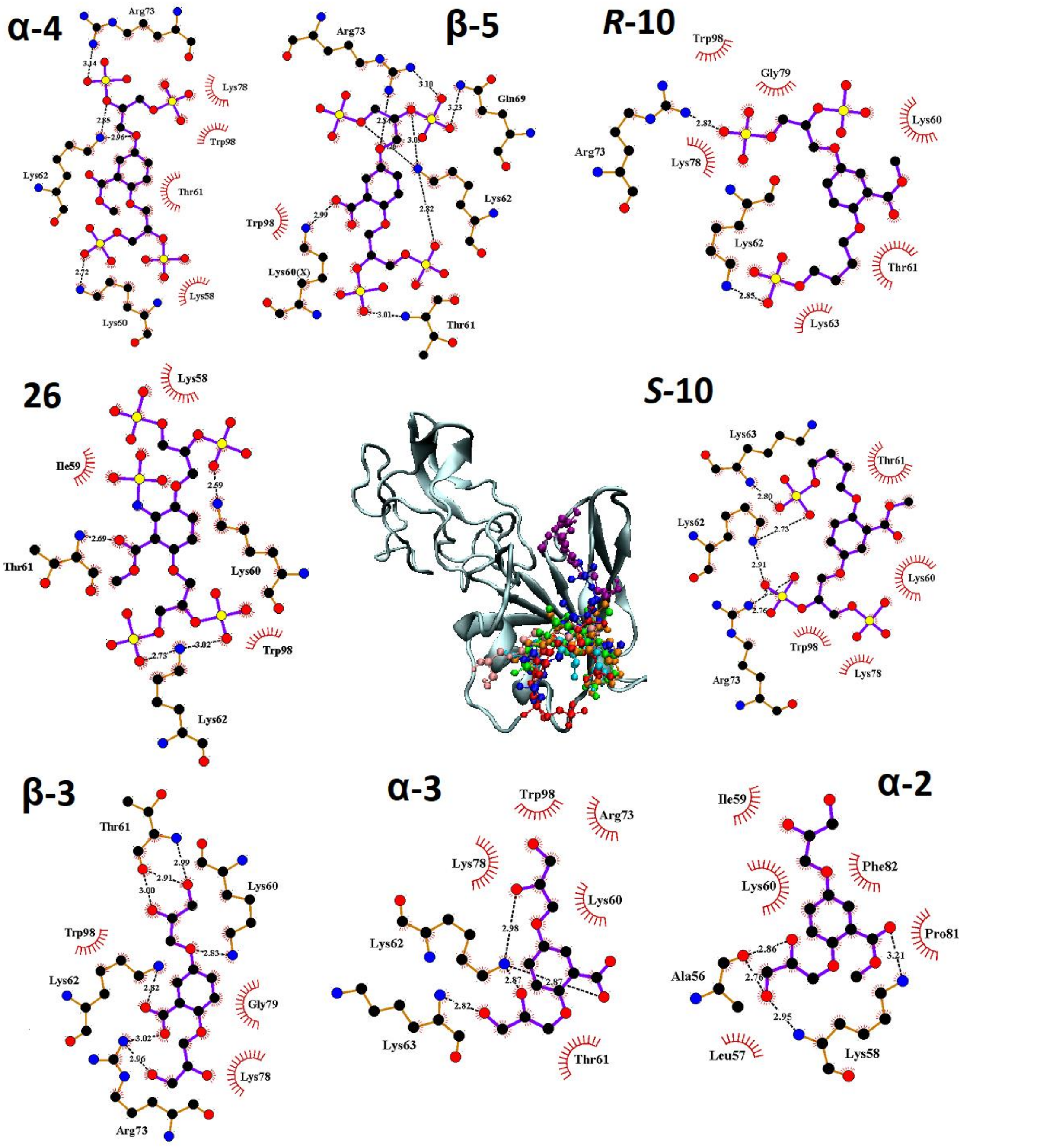

Figure S1: Microenvironments of interaction occupied by glycomimetic molecules (α-**4**-blue**,** β-**5**-red, *R*-**10**-orange**,** *S*-**10**-green**,** α-**2**-purple, α-**3**-cyan, β-**3**-pink, **26,** 1BHT protein is presented in ice blue.

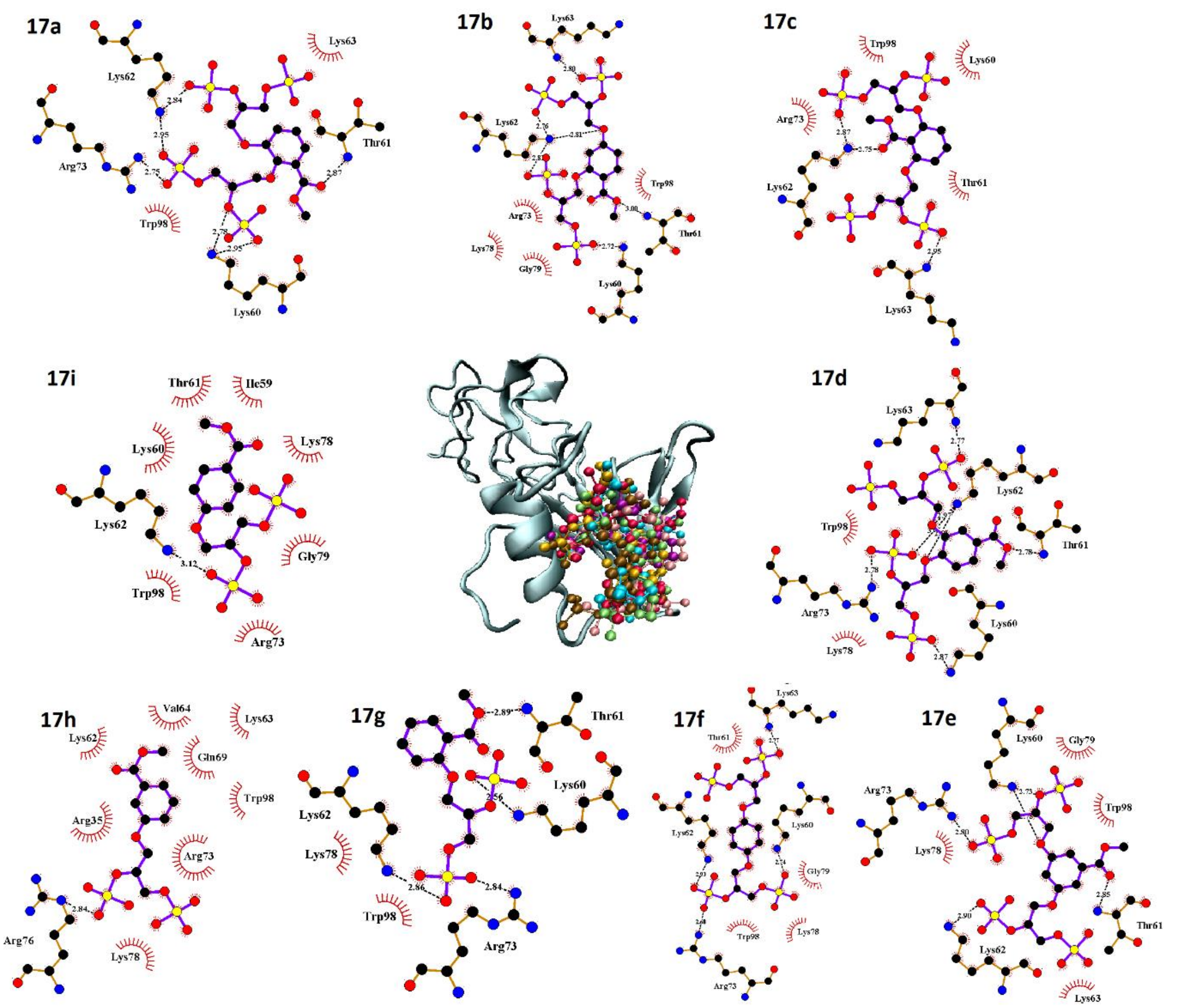

Figure S2: Binding microenvironments of sulfate molecule binding site for modified glycomimetics molecules (**17a**-pink, **17b**-lime, **17c**-ochre, **17d**-cyan2, **17e**-red2, **17f**-orange3, **17g**-purpl, **17h**-blue, **17i**, the 1BHT protein relaxed by molecular dynamics is represented in iceblue).

Additional Supporting Synthetic Schemes

The chemical synthesis of a series of second-generation HS-glycomimetics were achieved directly in 3 to 4 synthetic steps (Scheme S1). The phenolic positions were efficiently substituted with allyl groups *via* reaction with allyl bromide. The allyloxy intermediates were subsequently dihydroxylated using the Upjohn methodology,[S30] affording a variety of diols and tetraol intermediate precursors. Final sulfation of the dihydroxylated intermediates with TBSAB afforded a library of novel persulfated HS-glycomimetics based around the core structure of α-**4** (Scheme S1).

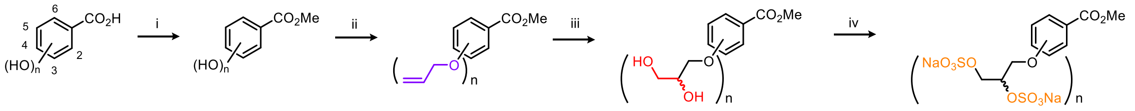

13	14	15	16	17
**a** (2-OH, 3-OH)	**a** (2-OH, 3-OH) 88%	**a** (2,3) 73%	**a** (2,3) 72%	**a** (2,3) 85%
**b** (2-OH, 4-OH)	**b** (2-OH, 4-OH) 88%	**b** (2,4) 78%	**b** (2,4) 60%	**b** (2,4) 23%
**c** (2-OH, 6-OH)	**c** (2-OH, 6-OH) 60%	**c** (2,6) 56%	**c** (2,6) 90%	**c** (2,6) 80%
**d** (3-OH, 4-OH)	**d** (3-OH, 4-OH) 80%	**d** (3,4) 74%	**d** (3,4) 54%	**d** (3,4) 68%
**e** (3-OH, 5-OH)	**e** (3-OH, 5-OH) 90%	**e** (3,5) 82%	**e** (3,5) 87%	**e** (3,5) 63%
	**f** (1,4-hydroxyquinone)	**f** 90%	**f** 94%	**f** 83%
**g** (2-OH)	**g** (2-OH) 99%	**g** (2) 91%	**g** (2) 97%	**g** (2) 70%
**h** (3-OH)	**h** (3-OH) 92%	**h** (3) 94%	**h** (3) 97%	**h** (3) 46%
**i** (4-OH)	**i** (4-OH) 97%	**i** (4) 80%	**i** (4) 97%	**i** (4) 72%

Scheme S1: The synthetic strategy and rational design for the second generation HS-glycomimetics, utilising previously demonstrated synthetic methods. Conditions: **i**) H_2SO_4, MeOH, reflux; **ii**) allyl bromide, K_2CO_3, TBAI, acetone, reflux; **iii**) $K_2OsO_2(OH)_4$, NMO, acetone/H_2O (9:1); **iv**) TBSAB, MeCN, 90 ˚C.

For all examples, each intermediate was achieved in good yield (esters **14**: 60 – 90%; for alkenes/dienes **15**: 56 – 90%; for polyols **16**: 54 – 94% and for persulfates **17**: 23 – 85%) following standard procedures. For the synthesis of **17c** both Fischer esterification and alkylation steps afforded the desired intermediates in moderate yields (60 & 56% for **14c** & **15c**, respectively).

Notably, for unsymmetrical tetraols **16a**, **16b** and **16d**, no *d.r* could be confirmed by analysis of the crude ^{1}H-NMR and ^{13}C-NMR spectra data. This is in accordance with the observed resonance signals for tetraol **2**, however, due to operating under non-asymmetric reaction conditions, the *e.r*/*d.r* of the polyols and corresponding persulfate are 1:1:1:1 (2*R*5*R*, 2*S*5*S, 2R*5*S, 2S*5*R*) in each case.

Finally, persulfation of the diol and tetraol intermediates **16** with TBSAB afforded nine novel organosulfates (**17a-i**) designed around the structure of HS-glycomimetic α-**4**.

Continuing from the original structure of HS-glycomimetic α-**4**, it was proposed that the installation of a *para*- sulfamate functionality, relative to the carbonyl, would be an interesting structure to synthesise due to a similar group being present in the structures of H and HS (**Scheme S2**).

H/HS-disacharide unit, **18** Target HS-glycomimetic **19**

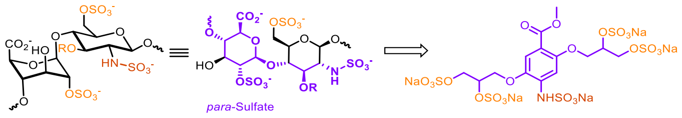

Scheme S2: The rational design of a glycomimetic containing a p-sulfamate functionality.

Attempts to synthesise this target structure **19** by nitration of ester **20** afforded **22** in 14% yield (**Scheme S3**). Which was conclusively confirmed by small molecule single crystal X-ray crystallography of ester, **22**.[S31] Nonetheless, the synthesis was carried forward, and the nitro intermediate was reduced to afford amine **25** in 96% yield. Sulfation of the hydroxyl and amine groups with TBSAB afforded the penta-sulfate **26** in 33% yield on a 12 mg (0.036 mmol) scale (**Scheme S3**).

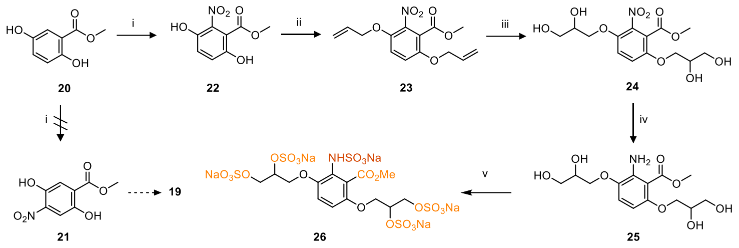

Scheme S3: The synthesis of HS-glycomimetic **26**. Conditions: **i**) HNO_3, AcOH, Ac_2O, **15**%; **ii**) Allyl-Br, K_2CO_3, TBAI, acetone, reflux, **48**%; **iii**) $K_2OsO_2(OH)_4$, NMO, acetone/H_2O, 9:1, 40 ˚C, **61**%; **iv**) H_2, Pd/C, MeOH (H-Cube), **94**%; **v**) 1) $Bu_3N{\bullet}SO_3$, MeCN, 90 ˚C, 2) NEH, ^{t}BuOMe/^{i}PrOH, **33**%.

References

S1. D. M. Gill, L. Male, A. M. Jones, *Chem Commun.* 2019, **55**, 4319 – 4322.

S2. J. A. Chavez, S. A. Summers, *Arch. Biochem. Biophys.,* 2003, **419**, 101 – 109

S3. A. M. Quitela, R. Jimenez, L. Piqueras, M. Gomex-Guzman, J. Haro, M. J. Zarzuelo, A. Cogolludo, M. J. Sanz, M. Toral, M. Romero, F. Perez-Vizcaino, J. Duarte, *Br. J. Pharmacol*., 2014, **171**, 3089 – 3102

S4. M .Romero, R. Jimenez, M. Sanchez, R. Lopez-Sepulveda, M. J. Zarluelo, F. O'Valle, A. Zarzuelo, F. Perez-Viscaino, J. Duarte, *Atherosclerosis*, 2010, **212**, 58 – 67

S5. A. M. Mahmoud, F. L. Wilkinson, A. M. Jones, J. A. Wilkinson, M. Romero, J. Duarte, M. Y. Alexander, *Biochimica et Biophysica Acta (BBA) - General Subjects*, 2017, **1861**, 3311 – 3322.

S6. M. Romero, R. Jimenez, M. Sanchez, R. Lopez-Sepulveda, A. Zarluelo, J. Tarmargo, F. Perez-Viscaino, J. Duarte, *Atherosclerosis*, 2010, **212**, 78 – 85

S7. Adapted from the procedure found in reference S6.

S8. A. K. Malde, L. Zuo, M. Breeze, M. Stroet, D. Poger, P. C. Nair, C. Oostenbrink, and A. E. Mark, *J. Chem. Theory Comput.* 2011, **7**, 4026-4037.

S9. G. M. Morris, R. Huey, W. Lindstrom, M. F. Sanner, R. K. Belew, D. S. Goodsell, and A. J. Olson, *J. Comput. Chem.* 2009, **30**, 2785-2791.

S10. W. Humphrey, A. Dalke, and K. Schulten, *J. Mol. Graph.* 1996, **14**, 33-38.

S11. N. Schmid, A. P. Eichenberger, A. Choutko, S. Riniker, M. Winger, A. E. Mark, and W. F. van Gunsteren, *Eur. Biophys. J.* 2011, **40**, 843-856.

S12. G. Bussi, D. Donaio, and M. Parrinello, *J. Chem. Phys.* 2007, **126**, 014101.

S13. M. Parrinello, and A. Rahman, *J. Appl. Phys.* 1981, **52**, 7182-7190.

S14. D. Van Der Spoel, E. Lindahl, B. Hess, G. Groenhof, A. E. Mark, H. J. C. Berendsen, *J. Comput. Chem.* 2005, **26**, 1701-1718.

S15. R. Kumari, R. Kumar, Open Source Drug Disovery Consortium, and A. Lynn, *J. Chem. Inf. Model.* 2014, **54**, 1951-1962.

S16. E. Baco, F. Hoegy, I. J. Schalk, G. L. A. Mislin, *Org. Biomol. Chem*., 2014, **12**, 749 – 757

S17. K. Schuenemannm, D. P. Furkert, E. C. Choi, S. Connelly, J. D. Fraser, J. Sperry, M. A. Brimble, *Org. Biomol. Chem*., 2014, **12**, 905 – 912

S18. F. Carta, D. Vullo, A. Maresca, A. Scozzafava, C. T. Suparan, *Bioorg. Med. Chem*., 2013, **35**, 1650 – 1662

S19. R. K. Pandey, G. G. Jarvis, P. S. Low, *Tetrahedron Lett*., 2012, **53**, 1627 – 1629

S20. C. C. Nawrat, L. I. Palmer, A. J. Blake, C. J. Moody, *J. Org. Chem*., 2013, **78**, 5587 – 5603

S21. Y. Zhang, J. Bao, X.-X. Deng, W. He, J.-J. Fan, F.-Q. Jiang, L. Fu, *Bioorg. Med. Chem. Lett.,* 2016, **26**, 4081 – 4085

S22. H.-L. C. Diaz, E. C. Polo, *J. Org. Chem.,* 2017, **82**, 4072 – 4112

S15. R. Epple, M. Azimioara, R. Russo, Y. Xie, X. Wang, C. Cow, J. Wityak, D. Karanewsky, B. Bursulaya, A. Kreusch, T. Tuntland, A. Gerken, M. Iskandar, E. Saez, H. M. Seidel, S.-S. Tian, *Bioorg. Med. Chem. Lett.,* 2006, **16**, 5488 – 5492

S24. Z.-M. Wang, M. Shen, *J. Org. Chem*., 1998, **63**, 1414 – 1418

S25. M. H. Prudhomme, G. Dauphin, G. Jeminet, *J. Antibiotics*, 1986, **39**, 922 – 933

S26. CrysAlisPro, Rigaku Oxford Diffraction, 2015.

S27. G. M. Sheldrick, *Acta Cryst.* 2015, **A71**, 3-8.

S28. G. M. Sheldrick, *Acta Cryst.* 2015, **C71**, 3-8.

S29. O. V. Dolomanov, L. J. Bourhis, R. J. Gildea, J. A. K. Howard, H. Puschmann, *J. Appl. Crystallogr.* 2009, **42**, 339-341.

S30. V. VanRheenen, R. C. Kelly, D. Y. Cha, *Tetrahedron Lett.* 1976, **17**, 1973.

S31. CCDC 2038360 (for **22**) contains the supplementary crystallographic data for this paper. These can be obtained free of charge from The Cambridge Crystallographic Data Centre.
